# On the sustainability of a family planning program in Nigeria when funding ends

**DOI:** 10.1371/journal.pone.0222790

**Published:** 2019-09-26

**Authors:** Ilene S. Speizer, David K. Guilkey, Veronica Escamilla, Peter M. Lance, Lisa M. Calhoun, Osifo T. Ojogun, David Fasiku

**Affiliations:** 1 Carolina Population Center, University of North Carolina at Chapel Hill, Chapel Hill, NC, United States of America; 2 Department of Maternal and Child Health, Gillings School of Global Public Health, University of North Carolina at Chapel Hill, Chapel Hill, NC, United States of America; 3 Department of Economics, University of North Carolina at Chapel Hill, Chapel Hill, NC, United States of America; 4 Data Research and Mapping Corporation, Abuja, Nigeria; Johns Hopkins Bloomberg School of Public Health, UNITED STATES

## Abstract

Few studies have examined the sustainability of family planning program outcomes in the post-program period. This article presents the results of a natural experiment where the Nigerian Urban Reproductive Health Initiative Phase I programming ended in early 2015 and Phase II activities continued in a subset of cities. Using data collected in 2015 and 2017, we compare contraceptive ideation and modern family planning use in two cities: Ilorin where program activities concluded in 2015 and Kaduna where program activities continued. The results demonstrate that exposure to program activities decreased in Ilorin but for those individuals reporting continuing exposure, the effect size of exposure on modern family planning use remained the same and was not significantly different from Kaduna. Modern family planning use continued to increase in both cites but at a lower rate than during Phase I. The results are useful for designing family planning programs that sustain beyond the life of the program.

## Introduction

There has been a growing emphasis on understanding the meaning and measurement of program sustainability in public health interventions [1–4]. The definition of sustainability varies across studies, but the focus is often on program components and outcomes that were developed or achieved in an earlier phase of a project and are maintained after the initial or focused funding period ends[5,6]. Sustainability is also called institutionalization, maintenance, durability, and continuation [5]and a number of studies have developed conceptual frameworks to inform the measurement of sustainability and/or scale-up of programs [1,3,7–9].

While much of the literature on the sustainability of health programs comes from developed countries[4,5], a growing body of work considers the sustainability of public health interventions in sub-Saharan Africa [1]and Asia [10–14]. A recent systematic review of the sustainability of health programs from sub-Saharan Africa found 41 studies that met the inclusion criteria that included: a) examining the status of the intervention during or after the initial funding ended; and b) examining continuation of the intervention. Many focused on communicable diseases with HIV and AIDS and malaria the most common; none examined the sustainability of a family planning (FP) program [1]. Studies of sustainability often use qualitative data and focus on systems-level outcomes and processes including whether program implementation continues at the school, health facility, or organizational level where it was initially introduced, and whether the program was implemented with fidelity [4,6,10,12,15,16]. A smaller number of studies examine whether individual-level behavioral outcomes are sustained and what factors lead to sustained behaviors.

Interestingly, Scheier’s systematic review on the sustainability of health-related programs in developed nations [5] indicated that about 80% of interventions that measured continuation demonstrated sustainability of at least one component in at least 60% of their sites after the program officially ended. Sustained interventions tended to have local ownership, were flexible to the program setting, and had visible benefits to the beneficiaries at all levels [5]. Reviews from developing countries have identified similar factors associated with successful scale-up and sustained delivery of programs at scale including factors relevant to ownership or leadership at the political, community, and organizational levels; plans for long-term financing; the program’s flexibility, adaptability and fit within the target organization; and the extent of service delivery capacity created for long-term implementation [1,7,17].

In the field of FP, one of the few examples of a rigorous examination of sustainability involved the Promoting Change in Reproductive Behavior (PRACHAR) program in Bihar, India that sought to delay marriage, delay first birth, and encourage contraceptive use within three months of a birth [18]. Evaluations of the PRACHAR program showed large impacts on key outcomes [18,19]. In 2013, Jeejebhoy and colleagues [20] used cross-sectional data to examine the sustainability of impacts in intervention and matched comparison sites four-years post-intervention. Because of differing study areas over time, the sustainability study used a post-test only design. It found significant differences between the intervention and comparison areas on awareness and use of FP, but no long-term effects were found on fertility or marriage outcomes. Interestingly, long-term effects on FP awareness and use were also found among women only indirectly exposed in the intervention communities [20], suggesting enduring spillover or diffusion effects to non-participants.

Another analysis of PRACHAR program sustainability examined whether young people who participated in a three-day training from intervention communities differed from matched young people from comparison communities four years after the training [21]. The authors demonstrated significantly higher awareness of sexual and reproductive health matters as well as better contraceptive practice among the intervention group exposed to the training as compared to the comparison group, however, this might relate to self-selection into the program as indicated by the authors [21]. Notably the primary outcomes of the program, including timing of marriage and timing of childbearing, did not differ across groups [21].

Another example of a FP program that had a follow-up evaluation of program sustainability is the Better Life Options (BLO) Program in India that examined intervention and comparison areas with another post-test only design [22]. The BLO program found that 1–4 years after program implementation, young women (ages 15–26) in the intervention community had more awareness of HIV, an older age at marriage, better school and employment outcomes, and higher contraceptive use, among other outcomes, than young women in comparison areas [22]. It is notable that the few sustainability studies of FP programs found were from India and tend to use post-test only and cross-sectional designs. There is a lack of rigorous evidence on FP program sustainability for sub-Saharan Africa.

This paper begins to fill this gap by examining the sustainability of the Nigerian Urban Reproductive Health Initiative (NURHI) program on FP attitudes and behaviors two-years after the initial program end date. It takes advantage of the fact that the program was completely terminated in one site and continued in a modified form in the other site. This study is unique not only because it contributes to the knowledge base about sustainability of FP programs but also because it uses novel data sources and methods to inform the key outcomes of ideation toward FP and modern contraceptive use. The results provide crucial evidence regarding the sustainability of the NURHI program on FP attitudes and behaviors as well as whether there are diminishing returns on the continuation of the same activities over the longer-term in the two settings: where the program continued and where the program terminated. The hope is that this study will be important for informing strategies to spread and scale-up FP programming in urban Nigeria and elsewhere in sub-Saharan Africa.

## Methods

### Data

The Nigerian Urban Reproductive Health Initiative (NURHI), funded by the Bill & Melinda Gates Foundation (BMGF) and implemented by the Johns Hopkins Center for Communications Programs, aimed to improve FP access and modern contraceptive use in select urban areas through comprehensive demand and supply side programming. The NURHI program utilized demand generation activities to encourage interpersonal discussion about FP, reduce barriers, myths, and social stigma, and increase approval of FP methods. Strategies included community-level outreach events, distribution of information, education, and communication (IEC) materials at public and private health facilities and in the communities, and through mass media, including television and radio programs. In addition, the NURHI program worked to improve the supply of FP services by training providers on provision of quality care and new methods; ensuring commodity supplies; and improving the overall clinic environment in target, high volume facilities. Phase I (2009–2014) of the NURHI FP program was implemented in six cities: Abuja, Benin City, Ibadan, Ilorin, Kaduna, and Zaria. For details of the NURHI program see Krenn et al. [23]. Following completion of Phase I activities, a subsequent Phase II of the NURHI project began in 2015 and continued in Oyo state (incorporating Ibadan) and Kaduna state (incorporating Kaduna city and Zaria) and expanded to Lagos state; program activities were ended in the other cities. Of note, Phase II of the NURHI program was less intense at the city level given lower funding levels and the focus on state-level programming rather than only cities.

The Measurement, Learning & Evaluation (MLE) project (also funded by BMGF), conducted the evaluation of the NURHI Phase I program with Data, Research and Mapping Consult (DRMC) and the National Population Commission as the local implementing partners [24]. The MLE project applied a hybrid design, collecting both longitudinal and cross-sectional survey data to evaluate the direct impact of NURHI on contraceptive use and behavioral and attitudinal norms in the six study cities. At endline (2015), a two-stage sampling design was used to identify a representative sample of women from each city. For the first stage selection of enumeration areas that could serve as primary sampling units, we used a sampling frame based on the 2006 Nigeria census that provided enumeration area (EA) population estimates that were the most accurate possible and thus allowed for the most efficient selection of representative samples of households. Census EAs were grouped into primary sampling units (PSUs) for random selection in the first stage of sampling. In the second stage of sampling, a household listing was undertaken and once complete, 41 households were randomly selected from selected PSUs. All women of reproductive age (15–49 years) living in the selected households were eligible to participate in the survey following informed consent. All women were asked to provide verbal consent to participate; study interviewers signed the consent forms following receipt of verbal consent. In 2015, we surveyed a total of 2,801 women from Kaduna, and 940 women from Ilorin.

In 2017, another cross-sectional representative survey was conducted by DRMC in both Kaduna and Ilorin to examine the sustainability of the NURHI program two-years after program operations terminated. We compare two cities: one where program activities concluded at the end of Phase I (Ilorin) and one where activities continued in Phase II of the NURHI program (Kaduna). All clusters surveyed in Kaduna and Ilorin in 2015 were included in the 2017 survey. As part of the 2017 data collection, we undertook a census of all households located in study clusters in Ilorin and Kaduna. All women ages 15–49 were eligible for interview after providing informed verbal consent. A total of 4,673 women from Kaduna and 2,209 women from Ilorin were surveyed in 2017. The study protocol and all consent procedures and consent forms for both the 2015 and 2017 surveys were approved by the Institutional Review Board at the University of North Carolina at Chapel Hill and by the National Health Research Ethics Committee of Nigeria (NHREC) in Nigeria.

The census of all households in study clusters in 2017 allowed us to match households and women from 2015 (that included a sample of households) to create a longitudinal sample of women from the two cities. During 2017 fieldwork, households that were interviewed in 2015 were identified and flagged for matching using information on the name of the household head and the household composition. In addition, a small number of households (n = 22) were matched after data collection using stringent criteria for matching (contact first author for details of these additional matches). Matching of women within matched households rested primarily on age and consistency of reported parity as of the 2015 round. A total of 1,535 (1,097 from Kaduna, 438 from Ilorin) women were matched across the two time periods for longitudinal analyses. It is worth noting that data are also available from the larger cross-sectional samples in 2015 and 2017 for the two cities (contact lead author for additional information). Multivariate analyses of the longitudinal sample are used to estimate NURHI’s continuing effects after Phase I activities formally concluded in 2015.

### Variables

At both time periods, all surveyed women were asked questions on fertility and family planning behaviors as well as information on exposure to the NURHI program, among other things. Questionnaires used came from earlier rounds of the study (baseline and midterm) and most of the variables included in this paper were validated as part of the impact evaluation of the NURHI program [24]. The first outcome of interest is modern contraceptive use. For this study, modern use includes use of injectables, intrauterine devices (IUD), implants, pills (emergency and daily), condoms (female and male), and sterilization (female and male). We excluded lactational amenorrhea method (LAM) from our measure of modern use because it was not one of the methods promoted by NURHI, and it is often mis-reported on surveys [25].

The second outcome of interest is women’s FP ideation, a representation of knowledge, values, beliefs, and communication related to FP and modern contraceptive use. Because of the large number of factors that relate to ideation, principal components analysis (PCA) was used to construct a single, continuous ideation index. The index is based on the same set of underlying factors used by NURHI to examine ideation in an earlier analysis [26]. The NURHI program theory of change identifies changes in a person’s beliefs, ideas, and feelings (ideational factors) as a key intermediate step to influencing contraceptive use over time [23]. Therefore, ideation is used as an outcome as well as an explanatory variable in the analyses that follow. The following binary variables were used for the ideation PCA: knowledge of more than six (median) modern contraceptive methods; reject more than six (median) myths regarding risks associated with modern contraception; discussed family size with partner in the last six months; discussed FP with partner in the last six months; discussed FP with significant other besides partner in the last year; perceived at least one non-spouse significant other supports her use of FP; perceived that all or most women in her community use FP; self-efficacy score above 4 out of 6 (median) that captures a woman’s comfort with obtaining or discussing FP with her partner and others; reportedly does not need permission to use FP; would recommend FP to someone. To make the comparison of ideation over time meaningful, we first estimated the PCA for the full 2017 cross-sectional sample from Kaduna and Ilorin. We used the full cross-sectional sample because it is representative of the population. The factor loadings from the first principal component were then applied to the full 2015 cross-sectional data to generate the continuous ideation index values for Kaduna and Ilorin in 2015. The same factor loadings were used over time to standardize the measure of ideation (see S1 Table for the factor loadings). Besides being an outcome variable, the ideation index was also treated as an endogenous explanatory variable in the modern contraceptive use model.

The key explanatory variables for these analyses are exposure to NURHI program related factors. The NURHI program had many demand side activities and our analyses focus on indicators for eight of them. In earlier longitudinal impact evaluation analyses, we estimated the causal impact of each of these activities separately (i.e., at the end of the NURHI Phase I program [24]). However, the sample size was much larger in that analysis and was spread across six cities. In the current work, we have data from two cities, Kaduna and Ilorin, and a smaller sample size. In addition, we wanted to test whether exposure effects were different for the two cities and different across the two time periods. To do this, we would have needed to create many interaction terms which would make interpretation of the results quite difficult. Therefore, for our first set of multivariate analyses, we use principal components analysis to construct a single, continuous exposure index. The index represented reported exposure to the following: NURHI television dramas and advertisements promoting FP; NURHI radio dramas and commercials promoting FP; community outreach presentations on FP at familial events including naming ceremonies and weddings; received SMS promoting FP; and exposure to any of the following media with NURHI FP or general FP slogans including provider badge with NURHI messages, NURHI logo, NURHI message cards, and billboards. The same approach used for standardizing the PCA of ideation over time (described above) was used for standardizing the PCA of the exposure variables. In a second set of analyses presented below, we examine grouped exposures to determine the marginal effects for various program components separately on modern contraceptive use.

In the NURHI impact evaluation, supply-side variables were included [24]. In this analysis, the supply-side variables including distance to the nearest facility, as well as characteristics of facilities within 1km of a woman’s residence such as whether the facility had recent FP stockouts, was recently renovated, or participated in NURHI training, were tested and were not significant and therefore were dropped from the final models. This insignificance of supply side factors is a common problem when trying to measure supply side effects in urban environments where services are densely packed, and one does not know the actual facility that the respondent may choose to visit. In a companion facility level analysis, we found that NURHI facilities had better quality in 2017 than non-NURHI facilities and quality was related to the number of family planning clients that visited a facility [27].

A migration related variable was also included as an explanatory variable in the models. We had no a priori belief about the direction of the effect of this regressor—whether a woman travelled to another city or town in the last year could increase or reduce NURHI exposure and contraceptive use. Control variables included age, education, union status (married or living with partner), religion, primary language spoken at home, and a household asset score (see Table 1 for classifications of these variables). The household asset score represented the sum of reported household materials and assets including cement walls, electricity, piped water in the home/yard, flush toilet to septic/sewer system, refrigerator, vehicle, and own home structure.

**Table 1:** Descriptive characteristics of longitudinal sample of women living in the Nigerian cities of Kaduna and Ilorin surveyed in both 2015 and 2017.

	Kaduna			Ilorin		
	2015	2017	P-value	2015	2017	P-value
Age (years)						
15–19	20.28	16.46	<0.001	16.37	14.39	0.042
20–24	19.63	16.50		17.45	11.97	
25–29	19.46	18.92		16.64	16.63	
30–34	17.63	16.56		18.74	16.99	
35–39	12.41	14.49		17.29	20.96	
40–44	6.53	10.12		8.95	11.06	
45–49	4.05	6.94		4.56	8.01	
Mean age (median)	27.53 (27)	29.62 (29)		29.03 (29)	31.02 (31)	
Marital status						
In union	65.66	64.74	0.306	72.74	71.25	0.330
Divorced/widowed	3.77	4.74		1.94	3.17	
Never married	30.57	30.52		25.32	25.59	
Education						
No education	11.60	8.67	<0.001	11.53	8.60	0.003
Primary	15.29	13.58		12.98	15.09	
Junior secondary	15.29	11.46		5.91	3.17	
Senior secondary	37.48	40.63		36.66	34.11	
Higher	20.33	25.66		32.91	39.04	
Religion						
Muslim Christian, Catholic, other	66.72 33.28	64.73 35.27	0.344	69.09 30.91	67.82 32.18	0.278
Primary language spoken at home						
Hausa	69.28	66.49	0.585	1.75	0.77	0.510
Yoruba	4.54	5.27		85.94	85.77	
English/Pigeon English	11.01	11.14		3.29	4.13	
Other	15.17	17.11		9.02	9.34	
Average asset score*	3.72	3.77	0.386	3.58	3.76	.025
Travel to another city in Nigeria in past year	42.42	51.05	0.002	40.04	47.09	0.007
Number of observations	1,097	1,097	--	438	438	--

Note: Significance based on F-tests. All descriptive percentages are weighted using weights from appropriate time period (2015 or 2017). Number of observations unweighted.

*Sum of reported household materials and assets including cement walls, electricity, piped water in the home/yard, flush toilet to septic/sewer system, refrigerator, vehicle, and own home structure; significance based on Wald test.

### Conceptual framework

**Fig 1:**
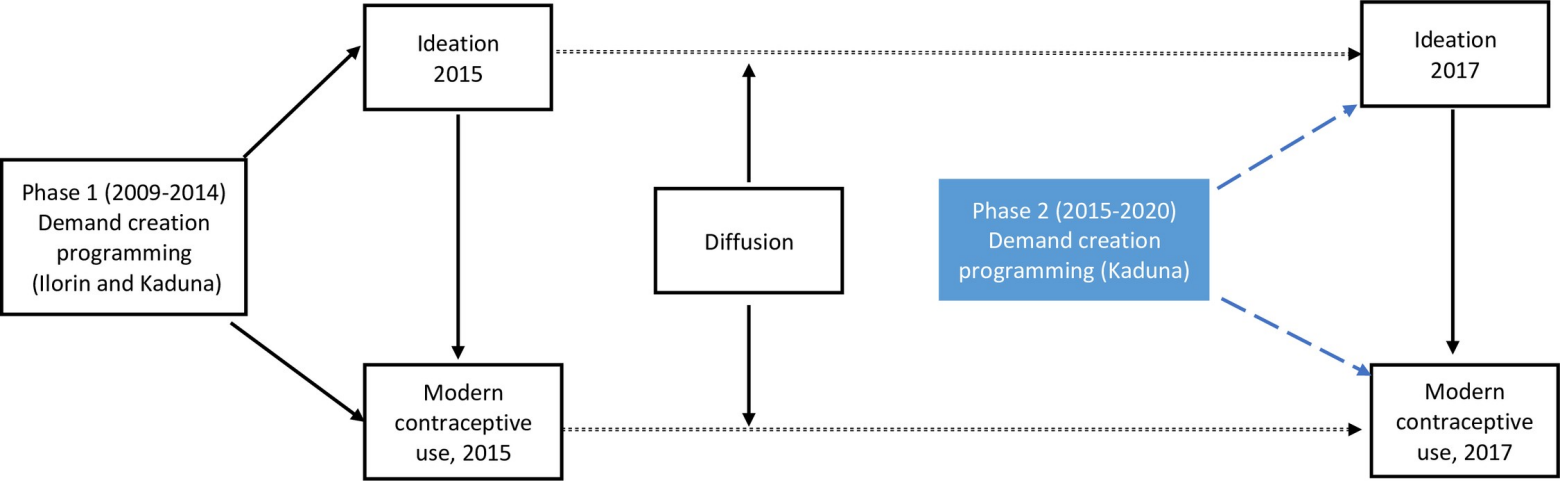
Conceptual framework for sustainability of family planning programming beyond the program cycle.

Fig 1 provides the conceptual framework for how FP programming can be sustained beyond program termination. Prior research has demonstrated that Phase 1 of NURHI programming was related to improved ideation and modern contraceptive use [24,26]; this is demonstrated on the left side of the framework. Moreover, literature on the diffusion of family planning programming has demonstrated that program effects can spread beyond initial adopters to other individuals in the city through the process of social learning [28]; these diffusion effects are shown with dotted lines. Also shown on the figure is the Phase 2 programming that takes place only in Kaduna. In this case, ideation and use may be higher in Kaduna than Ilorin because of this additional program exposure. Our analyses can measure the levels and trends in ideation and modern contraceptive use under these scenarios–Ilorin, where the program stopped in 2015 but diffusion effects may result in continued increases in ideation and contraceptive use in 2017, and Kaduna, where diffusion and continued programming both influence these outcomes.

### Statistical methods

We exploit the longitudinal sub-sample to estimate models that control for the potential endogeneity of exposure or ideation caused by correlation with unobservables at both the community and individual levels. We use two dependent variables: modern contraceptive use estimated using a logit framework and the ideation index using an extension of the linear regression model. We illustrate our methods using the more complicated, nonlinear logit model:

$$\ln\left[\frac{P(C_{ijt}=1)}{P(C_{ijt}=0)}\right]=\alpha_{1}+\alpha_{2}City_{ij}+\alpha_{3}t_{t}+\alpha_{4}E_{ijt}+\alpha_{5}X_{ijt}+\mu_{ij}$$ (1)

where the dependent variable is the log odds that woman i (i = 1,2,…,N_j_) from community j (j = 1,2,…,M) at time t (t = 1,2) is a current user of modern contraception at the time of the survey. *City_ij_* is a dummy variable indicating city of residence for woman i from community j, *E_ijt_* represents program exposure or the ideation index, *X_ijt_* represents individual level control variables such as age and education that are assumed to be exogenous, and *μ_ij_* represents time invariant unobservable individual and community level variables such as an individual’s level of motivation to limit family size or attitudes of community leaders towards modern contraceptive use. We do not specify separate time invariant errors for community and individual since, by design, we only have individuals from the 2015 survey who were found to still be living in the same community in 2017 and our estimation method is a variation of fixed effects because we suspect that program exposure is potentially correlated with this time invariant error.

The specific variant of fixed effects that we use is the correlated random effects model [29–31] which involves modeling the time invariant error as follows:

$$\mu_{ij}=\lambda_{1}{\bar{E}}_{ij}+\lambda_{2}{\bar{X}}_{ij}+\upsilon_{ij}$$ (2)

where ${\bar{X}}_{ij}$ and ${\bar{E}}_{ij}$ denote averages at the individual level of the two observations for the X and E variables respectively. We then substitute (2) into (1) to get:

$$\ln\left[\frac{P(C_{ijt}=1)}{P(C_{ijt}=0)}\right]=\alpha_{1}+\alpha_{2}City_{ij}+\alpha_{3}t_{t}+\alpha_{4}E_{ijt}+\alpha_{5}X_{ijt}+\lambda_{1}{\bar{E}}_{ij}+\lambda_{2}{\bar{X}}_{ij}+\upsilon_{ij}.$$ (3)

In the linear case, it is well known that if we estimate (3) using random effects or feasible generalized least squares (GLS) that the estimates of *α*_4_ and *α*_5_ will be identical to the fixed effects estimates or within estimates [32] and a joint test that the *λ*'*s* are equal to zero is a robust version of the Hausman test of the null hypothesis of no correlation between program exposure and the time invariant error term. In this logit form, we estimate Eq (3) by random effects logit. Note, unlike standard fixed effects methods, this hybrid approach to modeling that combines random and fixed effects permits us to obtain random effect estimates for regressors that do not vary with time such as the city dummy.

## Results

### Descriptive statistics

Table 1 provides the descriptive characteristics for the longitudinal data from 2015 and 2017. As a reminder, the city of Kaduna is where program activities continued after 2015 while program activities in Ilorin ceased in March of 2015. Table 1 demonstrates that, as expected, the women were somewhat older in 2017 than 2015 (i.e., mean age is two years higher in 2017). Further, in 2017, women from both cities were more likely to have traveled to another city than two years earlier, and the women were slightly more educated at the later date. Comparing across the cities, a slightly greater percentage of women in the sample from Ilorin were in union than in Kaduna. Further, women from Ilorin were more educated than women from Kaduna. Women from Kaduna were predominately Hausa speaking while women from Ilorin were predominately Yoruba speaking.

Table 2 presents descriptive statistics for the outcome variables. In Kaduna, modern contraceptive use significantly increased from 14.86% to 21.69% (p<0.001). In Ilorin, modern contraceptive use also increased from 22.89% to 27.07% (p = 0.049). As a percentage of modern method use, there was no significant change in method mix (long-acting methods vs. short acting methods) between 2015 and 2017.

**Table 2:** Modern contraceptive use, program exposure, and ideation characteristics for the longitudinal sample of women surveyed in 2015 and 2017, stratified by city.

	Kaduna			Ilorin		
	2015	2017	P-value	2015	2017	P-value
Total population	1,097	1,097	--	438	438	--
Contraceptive use						
% modern contraceptive use^†^	14.86	21.69	<0.001	22.89	27.07	0.049
Method mix among modern method users (n)	155	218		103	119	
% of users using long-acting methods^¥^	28.63	28.95	0.953	18.50	18.82	0.929
% of users using short-acting methods^¥^	71.37	71.05		81.50	81.18	
Ideation factors representing women’s knowledge, values, and beliefs toward FP						
Know about more than 6 methods out of 10*	59.03	65.25	0.015	65.88	61.42	0.210
Reject more than 6 myths out of 10*	49.95	54.97	0.222	54.88	66.11	0.018
Discuss family size with partner in last 6 months	20.51	80.45	<0.001	34.36	63.99	<0.001
Discuss FP with partner in last 6 months	41.92	26.97	<0.001		44.36	22.98	<0.001
Discuss FP with significant other besides partner in last year	39.09	52.91	<0.001	36.20	34.57	0.675
Perceive at least one non-spouse significant other supports her use of FP	56.90	80.67	<0.001	60.63	49.72	0.029
Perceive that all or most women in community use FP	26.69	25.43	0.703	15.37	17.35	0.353
Self-efficacy score >4 out of 6*	64.82	67.26	0.563	54.84	58.53	0.242
Does not need permission to use FP	11.30	16.03	0.071	7.98	12.63	0.012
Recommend FP to someone	20.02	30.53	<0.001	11.93	20.14	0.005
Mean ideation index value^£^	0.13	0.46	0.001	-0.09	-0.19	0.355
Exposure to individual NURHI demand generating activities						
Television programs	42.64	76.77	<0.001	81.94	59.21	<0.001
Radio programs	48.45	73.59	<0.001	89.26	76.16	0.016
Community outreach	23.56	38.25	<0.001	15.85	11.28	0.085
Provider badge	11.33	13.41	0.380	31.97	25.46	0.105
SMS	5.45	9.37	0.036	21.56	6.15	<0.001
Billboard	6.58	4.63	0.311	24.83	2.70	<0.001
Cards	6.48	18.31	<0.001	31.26	14.08	<0.001
Logo	49.21	76.08	<0.001	79.29	77.36	0.592
Mean exposure index value^£^	-0.34	0.27	<0.001	1.25	-0.03	<0.001
Exposure to grouped NURHI demand generating activities						
Mass media–Television and/or radio programs	58.87	88.44	<0.001	93.26	81.20	0.049
Print media–Billboard, provider badge, cards	19.22	29.07	0.003	49.40	35.05	0.002
Community outreach and SMS	26.95	43.00	<0.001	30.92	16.77	<0.001

Note: All values are weighted using weights from the appropriate time period (2015 or 2017).

† Modern methods include: injectables, intrauterine device (IUD), implants, pills (emergency and daily), condoms, and sterilization.

^¥^Denominator is women using modern contraceptive methods, excluding LAM; long-acting methods include sterilization, implants, and IUDs; short-acting methods include daily and emergency pills, injectables, and condoms.

*Binary variable generated using median value as cutoff.

^£^Principle components analysis used to generate continuous ideation index that combines the ten binary ideation factors, using data from Kaduna and Ilorin in 2017. We then applied the 2017 loadings from the first principal component to the 2015 ideation factors to manually generate continuous ideation index values for Kaduna and Ilorin in 2015. The same approach was used to generate a continuous exposure index, using the eight individual exposures.

Table 2 also includes descriptive statistics for ideation and exposure. In Kaduna, there was a significant increase in the ideation index from 0.13 to 0.46 (p<0.001) between 2015 and 2017. Certain ideation factors significantly increased including knowledge on FP; discussion of family size with the respondent’s partner in last 6 months; discussion of FP with a significant other besides the partner in last year; perceived support for FP; does not need permission to use FP; and would recommend FP to someone. The exception was for discussion of family planning in the last 6 months which significantly declined between 2015 and 2017 (p<0.001). The results for Ilorin show a different pattern. Overall, the ideation index in Ilorin did not change significantly between 2015 and 2017; however, a few of the items significantly improved including reject myths; discussion of family size with partner; does not need permission to use FP; and would recommend FP. Other items significantly declined over the period including discussion of FP in the last six months and perceived support for family planning. Notably, ideation was higher in Kaduna than in Ilorin in 2015.

The key independent variable of interest is exposure to NURHI demand generation activities. In Kaduna where the program continued, women generally reported significantly greater exposure to the NURHI activities in 2017 relative to 2015 (see Table 2). Conversely, while Ilorin had higher exposure in 2015 than Kaduna, over the follow-up period, exposure in Ilorin declined significantly; this is not surprising since the program ended in 2015. Overall, the mean exposure value in Kaduna goes up over time (from -0.34 to 0.27) while the mean value goes down in Ilorin (from 1.25 to -0.03). Finally, the bottom of Table 2 provides grouped exposure measures. We see that for Kaduna, the mass media (radio and television), print media (billboards, provider badge, and cards), and community (outreach and SMS) measures all significantly increased between 2015 and 2017 whereas in Ilorin, they all significantly declined in the same period.

### Multivariate results

Table 3 presents results for three sets of models. For all three regression models, a joint test of the null hypothesis that the *λ*'*s* for Eq (3) are equal to zero was strongly rejected at all standard significance levels. This means that the use of correlated random effects to control for the possibility of correlation between individual and community level unobservables and NURHI program related variables was necessary and standard methods would yield biased estimates for program exposure. This also justifies our use of the longitudinal sub-sample rather than the larger cross-sectional samples.

**Table 3:** Correlated random effects results for the 2015 and 2017 longitudinal sample–three sets of results defined by the outcome and main predictors.

	Coefficient	SE	P-value
**(a) Ideation and exposure index**			
Kaduna	0.8000	0.1004	<0.001
2017 dummy	0.1851	0.0442	<0.001
Exposure index	0.2453	0.0204	<0.001
Travel to another city in Nigeria in past year	0.0095	0.0602	0.874
**(b) Modern contraceptive use and ideation index**			
Kaduna	-0.5155	0.2882	0.074
2017 dummy	0.3417	0.1318	0.010
Ideation index	0.6185	0.0735	<0.001
Travel to another city in Nigeria in past year	-0.3876	0.1742	0.026
(c) Modern contraceptive use and exposure index*			
Kaduna	0.1688	0.2811	0.548
2017 dummy	0.4172	0.1265	0.001
Exposure index	0.2025	0.0563	<0.001
Travel to another city in Nigeria in past year	-0.3602	0.1667	0.031

Note: Models adjusted for age, marital status, religion, education, language, and household assets.

*Results for model (c) controls presented in Table 4; Similar control variable results obtained for models (a) and (b), not shown.

Model (a) examines the relationship between exposure to NURHI program components and ideation, controlling for demographic factors; model (b) examines how ideation is related to modern method use; and model (c) examines how NURHI exposure relates to modern method use. Table 4 provides the control variables from Model (c) which are similar across all three models. In preliminary models (S2 and S3 Tables), we used interaction terms to test for differing effects of exposure and ideation by city, by year, and by whether the respondent was Muslim. We also used three and four way interactions to capture differing effects with all possible combinations. Overall, the interaction terms were not significant, which was unexpected. We expected to see significant interactions between the 2017 dummy and living in Kaduna reflecting continued exposure to the NURHI program in this site. Based on the preliminary results that were generally not significant, we present below the results without the interactions.

**Table 4:** Controls for correlated random effects model [Table 3, model (c)] of modern contraceptive use and exposure index for the 2015 and 2017 longitudinal sample.

	Coefficient	SE	P-value
Age			
15–19 years	Ref		
20–24 years	1.7821	0.6742	0.008
25–29 years	1.7250	0.7448	0.021
30–34 years	2.3288	0.8054	0.004
35–39 years	2.5186	0.8599	0.003
40–44 years	2.3499	0.9225	0.011
45–49 years	2.6055	1.0403	0.012
Marital status			
Never married	Ref		
In union	0.0957	0.5155	0.853
Divorced/widowed	0.1035	0.7582	0.891
Education			
None	Ref		
Primary	-0.4363	0.4130	0.291
Junior secondary	0.4551	0.5143	0.376
Senior secondary	0.2979	0.4789	0.534
Higher	0.7286	0.5779	0.207
Religion			
Christian, Catholic, other Muslim	Ref -1.1411	0.5468	0.037
Primary language spoken at home (%)			
Hausa	Ref		
Yoruba	-0.6868	0.6223	0.270
English/Pigeon English	-0.0542	0.4193	0.897
Other	-0.0327	0.4620	0.943
Household asset score	-0.0263	0.0970	0.786

The multivariate results (Table 3) for model (a) show a strongly positive and significant effect for the Kaduna dummy suggesting that all else being equal, ideation is higher in Kaduna, the city where the program continued. We also see that ideation continued to increase in 2017 and exposure to NURHI program variables had a strong positive effect on ideation.

In model (b) with modern contraceptive use as the dependent variable, we see that the sign of the Kaduna dummy is negative (p<0.10). Further, the effect of the ideation index is strongly significant and positive. From model (a) we know that the Kaduna dummy has a strong positive effect on ideation and thus the ideation effect is likely capturing some of this higher ideation in Kaduna. Modern contraceptive use continued to increase in 2017, and recent travel had a significantly negative effect on modern use. We have no a priori expectations for the sign of the travel variable since women who move between cities may be more likely to use contraception if they have greater access to methods from their travel or may have less need for contraception if they are moving around and are less likely to be having regular sex.

Finally, model (c) shows the direct effects of exposure on modern contraceptive use. The effects that are positive and significant are the time dummy and the exposure index where over time, use is higher and women who have greater exposure are more likely to use. The Kaduna dummy has a positive point estimate; however, it is far from significant. The effect of recent travel is basically the same as model (b).

Results for the control variables from the three sets of models are presented in Table 4. We present control variable results from model (c) which examines the relationship between NURHI exposure and modern contraceptive use since this is the primary model of interest. Generally, the control variables had the expected effect such that older women were more likely to use FP and Muslim women were less likely to use FP.

The major conclusion one can draw to this point is that exposure to NURHI programs has continued to have strong positive effects on both ideation and modern contraceptive use and that ideation has strong positive effects on modern contraceptive use and so it is a major pathway through which exposure has its effect. However, the exposure index, while it was quite useful when we tested for many interaction effects, does not allow us to examine each program component separately. Since we found almost no significant interaction effects and a joint test of the interaction terms failed to reject the null that the effects were jointly zero (see results including interactions in S2 and S3 Tables model (c)), we now examine the effects for groups of program exposure components as well as a measure of the effect of a small set of them simultaneously.

### Marginal effects for program components

To examine specific marginal effects for each of the program components, we replaced the exposure index with seven exposure measures and then re-estimated model (c) (results not shown). Note that the logo exposure measure was dropped because it tended to be found as part of many of the other exposure measures. We then calculated marginal effects for each program component. Since all exposures are binary, the marginal effect is the difference between predicted modern contraceptive use with the exposure set to zero and then set to one and all other variables kept at their actual empirical values. The marginal effects for individual exposures were not significant probably due to collinearity. However, when we examine the effect of groups of similar exposures (mass media; print media; and outreach), we see some significant effects (Table 5). Print media, which captures exposure to the NURHI logo on one or more of the following: billboards, cards, or provider badge, has a positive effect on modern contraceptive use. Exposure to mass media which includes NURHI television and/or radio programs also has a positive effect on modern contraceptive use. Outreach, which includes receiving FP messages at life events or SMS promoting FP, was not significant. Of note, mass media and print media activities may be sustained after the program leaves by remaining available (e.g., billboards not taken down) or through television programming that spreads beyond the state-level setting (e.g., through national campaigns or national advertisements). We also calculated a marginal effect for exposure to both mass media (TV and/or radio) and print media (billboard and/or cards and/or badge) at once and we see a substantial impact on modern contraceptive use. However, it should be noted that in 2015, 78% of all respondents were exposed to either mass media or print media, while 33% were exposed to both. In 2017, the numbers were 87% and 30% respectively.

**Table 5:** Marginal effects for the grouped NURHI exposure variables* on modern contraceptive use in 2015 and 2017 longitudinal sample.

	Margin	SE	P-value
NURHI exposure			
Mass media	0.0443	0.0227	0.05
Print media	0.0443	0.0214	0.04
Outreach	0.0184	0.0204	0.37
Joint exposure: Media + Print media	0.0882	0.0293	<0.01
2017 (vs. 2015)	0.0356	0.0138	0.01

Note: Media represents exposure to NURHI TV and/or NURHI radio programs or advertisements; Outreach includes presentations promoting FP at life events including naming ceremonies, and/or receiving SMS promoting FP; Print media includes recall of viewing the NURHI logo on billboards, NURHI message cards, or provider badges.

*Model is similar to Model 3c above, however, instead of the exposure index the grouped exposure measures are included; models also are adjusted for age, marital status, religion, education, language, and household assets.

Finally, the marginal effect for the 2017 dummy variable is also presented in Table 5 and we see that it has a significant and positive effect on modern contraceptive use. This is after controlling for exposures and demographic characteristics. One possible explanation is diffusion, as hypothesized in the conceptual framework. An analysis of Nigeria data found significant effects of social learning on contraceptive use as measured by community level contraceptive use [33]. A similar, mechanism could be at work here, but we do not explicitly try to model social learning in this work.

## Discussion

This paper takes advantage of a natural experiment that occurred in two cities in Nigeria where the NURHI FP program received funding to continue operations in one city and its surrounding state (Kaduna) and not in another (Ilorin). We use data gathered in 2015, which coincided with the termination of the program in Ilorin, and in 2017, two years after termination, to see how the two cities fared.

As we point out in the introduction, there have been very few studies that have examined the sustainability of FP programs anywhere in the world and none that we are aware of from sub-Saharan Africa that had data at the time of program termination and data from a later point in time. We not only have this type of data for two repeated cross-sections, but we also have a subset of the sample where we have the same individuals at two points in time and so we are able to use the powerful tools associated with longitudinal data in this analysis.

An interesting descriptive result is that respondents in Ilorin reported continued exposure to the different aspects of the program even though it was terminated but at a level that was reduced from the peak exposure experienced in 2015. In contrast, exposure continued to rise in Kaduna. Because of the large number of exposures, we used principal components analysis to develop an exposure index that then could be used to test for interactions of exposure with city, time, and religion. Surprisingly, we found little evidence of interaction effects. Thus, the effect size of exposure in Ilorin was not statistically different from the effect size in Kaduna. The time and religion interactions also turned up little of substance. It is also notable that ideation had a similar pattern to exposure such that it decreased over time in Ilorin while it increased in Kaduna. However, the effect of ideation on contraceptive use remained positive for both cities and exposure to NURHI program activities was significantly related to ideation for both cities as well.

The descriptive statistics provide some interesting perspectives on observed changes in the sample. Notably, in both Kaduna and Ilorin, modern contraceptive use increased significantly. Further, as mentioned above, there was significantly more exposure in Kaduna in 2017 relative to 2015 while exposure significantly dropped in Ilorin. The demographic factors worked in favor of increasing use in this longitudinal sample with more respondents “aging into” ages where we tend to see higher use and marital status having little change over time. This demonstrates the importance of undertaking the multivariate analyses.

The results of the analyses that teased apart the exposure items demonstrated that a number of the NURHI program activities, when grouped by similar exposure methods, were related to continued contraceptive use in the follow-up period. Mass media and print media had significant effects in the final analyses. Notably, mass media increased significantly in Kaduna but decreased in Ilorin over the 2015 to 2017 period. Similarly, exposure to print media significantly increased in Kaduna but significantly decreased in Ilorin. These types of activities may continue to have an effect in Ilorin through sustained effects or diffusion if, for example, the outreach workers continue to use messages learned earlier under the NURHI program or if the television shows or advertisements are seen through the national media or through local cable channels. In an analysis that used the 2015 Nigeria data, authors [33] demonstrated diffusion of program effects beyond the study cities into rural parts of the study states; this may be what is taking place in Ilorin to sustain the activities, but at a lower level.

Our results can be compared to the earlier studies on sustainability of FP programs in India and for other health outcomes in sub-Saharan Africa [1,21]. Like the PRACHAR program, we demonstrate that the intermediate outcome of ideation sustains over time; however, ideation is significantly higher in the city that continued compared to the city where the program was terminated [21]. That said, we do find that both ideation and exposure continue to be related to modern method use in both cities. Our study differs from many of the sub-Saharan African studies by including quantitative data and focusing on outcomes (ideation and contraceptive use) whereas many of the studies of sustainability of other health programs focus on the sustainability of program components (e.g., quality of care or program implementation) and are undertaken using qualitative methods [1,4,6,10,12,15,16]. Future studies examining family planning outcomes post-program termination are important for understanding the circumstances of program sustainability beyond the initial funding cycle.

This study is not without limitations. The first limitation is that it was difficult to examine the exposure effects with the granularity that we would have liked since there were a large number of program components and there was correlation across the NURHI program activities. With that in mind, we created an exposure index which demonstrated the importance of NURHI exposure in both Kaduna that continued activities as well as in Ilorin where activities ended; overall exposure declined in Ilorin over time. Second, the longitudinal sample used for the multivariate analysis, while providing a more rigorous analysis approach, was not large enough to allow us to stratify by city which would have been helpful for understanding city-level differences. Third, women are self-reporting their exposure to the program activities and while they were asked about exposure in the last year for many of the components, the radio and television measures were broader and may capture remembered exposure from earlier in the NURHI Phase I programming. With the data available, it is not possible to control for this potential retrospective reporting bias. Finally, discussions with the NURHI team have indicated that in NURHI Phase II, a focus of the program was to spread to the state-level. This meant that efforts were less intensive in the city of Kaduna in the two-year follow-up period. This may have attenuated the effect of program sustainability in Kaduna from what might have been expected if Kaduna city had remained the primary target area of the program.

This study also has important strengths. Data were collected to examine the sustainability of program activities after the program ended; these types of data are rarely available post-program funding. The results of this study demonstrate important results to inform future FP programming. In particular, the program effects sustained two-years out such that exposure to the program continued to be related to ideation, a key intermediate factor for the NURHI program, and modern contraceptive use. Further, there may be some diffusion effects such that women continue to discuss program-related factors (e.g., discuss family size; reject myths; etc.) and this discussion may help to spread the effects of the program post-implementation. Finally, this study showed that in Kaduna, the city where the program continued, there was less increase in modern method use than in the earlier phase. This may reflect diminishing returns of program activities, or alternatively may reflect the increased focus of program activities on other parts of the state.

To conclude, the effect of the NURHI Phase I program sustained and was associated with continued increases in modern contraceptive use, even in the city where the program ended. It is notable that the NURHI Phase I program activities did not have an explicit plan for sustainability and therefore, sustained exposure, whether through diffusion or remembering earlier messages on the radio and television, is promising. That said, exposure in Ilorin did decline significantly, indicating a risk that with more time, the effect of NURHI programming on ideation and modern contraceptive use may fade. As part of NURHI Phase II, greater attention is focused on identifying strategies that are sustainable and engaging the government for long-term support. That said, in this analysis, the exposure effects in Kaduna were no greater than those in Ilorin; this might reflect the program focus in NURHI Phase II on spreading to rural areas and thus not continued attention to urban areas. Funders and program planners need to consider undertaking activities that can be sustained from the launch; this likely involves engaging the government and public sector throughout program design and implementation, as done under NURHI Phase I and more strongly done under NURHI Phase II. That said, there is also a need to identify strategies to insert new energy into FP programming to help affect greater increases in modern method use in a place like Kaduna that has had FP programming for a number of years. This study demonstrated sustainability of program activities; however, questions remain on what will happen over time given observed declines in exposure in Ilorin. Future studies are needed to examine the long-term impact of these declines on modern family planning use.

## Supporting information

S1 Table Principal components analysis factor loadings used to generate ideation and exposure variables. Factor loadings obtained using full representative cross-sectional sample for Kaduna and Ilorin in 2017 were used to calculate 2015 values.
(DOCX)

S2 Table Correlated random effects results with interactions for the 2015 and 2017 longitudinal sample–three sets of results defined by the outcome and main predictors. (DOCX)

S3 Table Controls for correlated random effects model [S2 Table, model (c)] Modern contraceptive use and exposure index for the 2015 and 2017 longitudinal sample. (DOCX)

S1 File Nigeria 2015 Endline Woman Questionnaire.pdf–woman’s questionnaire used in 2015 survey. (PDF)

S2 File Nigeria 2017 Sustainability Woman Questionnaire–woman’s questionnaire used in 2017 survey. (PDF)
