# Supplementary material for: On the sustainability of a family planning program in Nigeria when funding ends
Source: PLoS One. 2019 Sep 26;14(9):e0222790. doi: 10.1371/journal.pone.0222790 (PMC6762171; doi:10.1371/journal.pone.0222790)
Supplement: S1 Table — Factor loadings obtained using full representative cross-sectional sample for Kaduna and Ilorin in 2017 were used to calculate 2015 values. (DOCX) [file pone.0222790.s001.docx]

**S1 Table. Principal components analysis factor loadings used to generate ideation and exposure variables. Factor loadings obtained using full representative cross-sectional sample for Kaduna and Ilorin in 2017 were used to calculate 2015 values**

|  |  | **2017 cross-sectional sample PCA factor loadings** |
| --- | --- | --- |
| **Ideation factors representing women’s knowledge, values, and beliefs toward FP** |  |  |
| Know about more than 6 methods out of 10* |  | 0.3723 |
| Reject more than 6 myths out of 10* |  | 0.0285 |
| Discuss family size with partner in last 6  months |  | -0.1309 |
| Discuss FP with partner in last 6 months |  | 0.3443 |
| Discuss FP with significant other besides  partner in last year |  | 0.4440 |
| Perceive at least one non-spouse significant  other supports her use of FP |  | 0.3840 |
| Perceive that all or most women in community  use FP |  | 0.3372 |
| Self-efficacy score >4 out of 6* |  | 0.2812 |
| Does not need permission to use FP |  | 0.0024 |
| Recommend FP to someone |  | 0.4331 |
|  |  |  |
| **Exposure to individual NURHI demand generation activities** |  |  |
| Television programs |  | 0.4530 |
| Radio programs |  | 0.3993 |
| Community outreach |  | 0.3289 |
| Provider badge |  | 0.3542 |
| SMS |  | 0.2761 |
| Billboard |  | 0.0979 |
| Cards |  | 0.3902 |
| Logo |  | 0.4045 |
