# Supplementary material for: On the sustainability of a family planning program in Nigeria when funding ends
Source: PLoS One. 2019 Sep 26;14(9):e0222790. doi: 10.1371/journal.pone.0222790 (PMC6762171; doi:10.1371/journal.pone.0222790)
Supplement: S2 Table — (DOCX) [file pone.0222790.s002.docx]

**S2 Table. Correlated random effects results with interactions for the 2015 and 2017 longitudinal sample – three sets of results defined by the outcome and main predictors**

|  | **Coefficient** | **SE** | **P-value** |
| --- | --- | --- | --- |
| **(a) Ideation and exposure index** | | | |
| Kaduna | 1.0568 | 0.1313 | <0.001 |
| 2017 dummy | 0.0767 | 0.1660 | 0.644 |
| Exposure index | 0.1713 | 0.0633 | 0.007 |
| Travel to another city in Nigeria in past year | -0.0042 | 0.0605 | 0.944 |
| Interaction terms |  |  |  |
| Exposure index and 2017 dummy | -0.0959 | 0.1064 | 0.367 |
| Exposure index, 2017 dummy, and Kaduna | -0.0744 | 0.1226 | 0.544 |
| Kaduna and 2017 dummy | -0.1175 | 0.1889 | 0.534 |
| Kaduna and exposure index | 0.0910 | 0.0600 | 0.129 |
| Muslim and 2017 dummy | 0.1181 | 0.1875 | 0.529 |
| Muslim and Kaduna | 0.8811 | 0.5110 | 0.085 |
| Muslim and exposure index | 0.0623 | 0.0613 | 0.310 |
| Muslim, 2017 dummy, and Kaduna | 0.1554 | 0.2230 | 0.486 |
| Muslim, 2017 dummy, and exposure index | 0.1423 | 0.1221 | 0.244 |
| Muslim, 2017 dummy, Kaduna, and exposure index | -0.0764 | 0.1371 | 0.577 |
| **(b) Modern contraceptive use and ideation index** | | | |
| Kaduna | -0.2333 | 0.3468 | 0.501 |
| 2017 dummy | 0.4814 | 0.3656 | 0.188 |
| Ideation index | 0.3850 | 0.1915 | 0.044 |
| Travel to another city in Nigeria in past year | -0.3468 | 0.1758 | 0.049 |
| Interaction terms |  |  |  |
| Ideation index and 2017 dummy | 0.0550 | 0.2689 | 0.838 |
| Ideation index, 2017 dummy, and Kaduna | 0.3147 | 0.3134 | 0.315 |
| Kaduna and 2017 dummy | -0.8658 | 0.4766 | 0.069 |
| Kaduna and ideation index | 0.1128 | 0.1819 | 0.535 |
| Muslim and 2017 dummy | -0.2706 | 0.4425 | 0.541 |
| Muslim and Kaduna | -0.1844 | 1.3843 | 0.894 |
| Muslim and ideation index | 0.0671 | 0.1828 | 0.714 |
| Muslim, 2017 dummy, and Kaduna | 0.8437 | 0.6032 | 0.162 |
| Muslim, 2017 dummy, and ideation index | -0.1431 | 0.3106 | 0.645 |
| Muslim, 2017 dummy, Kaduna, and ideation index | 0.0838 | 0.3581 | 0.815 |
| **(c) Modern contraceptive use and exposure index*** | | | |
| Kaduna | 0.8342 | 0.3600 | 0.020 |
| 2017 dummy | 0.4600 | 0.4471 | 0.304 |
| Exposure index | 0.0466 | 0.1625 | 0.774 |
| Travel to another city in Nigeria in past year | -0.3377 | 0.1683 | 0.045 |
| Interaction terms |  |  |  |
| Exposure index and 2017 dummy | 0.3178 | 0.2722 | 0.243 |
| Exposure index, 2017 dummy, and Kaduna | -0.2976 | 0.3100 | 0.337 |
| Kaduna and 2017 dummy | -0.5212 | 0.4914 | 0.289 |
| Kaduna and exposure index | 0.1536 | 0.1599 | 0.337 |
| Muslim and 2017 dummy | -0.2651 | 0.5015 | 0.597 |
| Muslim and Kaduna | 0.2202 | 1.4284 | 0.877 |
| Muslim and exposure index | 0.0647 | 0.1601 | 0.686 |
| Muslim, 2017 dummy, and Kaduna | 1.0123 | 0.5879 | 0.085 |
| Muslim, 2017 dummy, and exposure index | -0.0862 | 0.3222 | 0.789 |
| Muslim, 2017 dummy, Kaduna, and exposure index | -0.0340 | 0.3595 | 0.925 |

Note: Models adjusted for age, marital status, religion, education, language, and household assets.

*Results for model (c) controls presented in Table 4; Similar results obtained for models (a) and (b), controls not shown.
