# Supplementary material for: On the sustainability of a family planning program in Nigeria when funding ends
Source: PLoS One. 2019 Sep 26;14(9):e0222790. doi: 10.1371/journal.pone.0222790 (PMC6762171; doi:10.1371/journal.pone.0222790)
Supplement: S3 Table — (DOCX) [file pone.0222790.s003.docx]

**S3 Table. Controls for correlated random effects model [S2 Table, model (c)] Modern contraceptive use and exposure index for the 2015 and 2017 longitudinal sample**

|  | **Coefficient** | **SE** | **P-value** |
| --- | --- | --- | --- |
| Age (%) |  |  |  |
| 15-19 years | Ref | Ref | Ref |
| 20-24 years | 1.7034 | 0.6681 | 0.011 |
| 25-29 years | 1.6105 | 0.7415 | 0.030 |
| 30-34 years | 2.2237 | 0.8045 | 0.006 |
| 35-39 years | 2.3791 | 0.8585 | 0.006 |
| 40-44 years | 2.3004 | 0.9216 | 0.013 |
| 45-49 years | 2.5927 | 1.0436 | 0.013 |
| Marital status (%) |  |  |  |
| Never married | Ref | Ref | Ref |
| In union | 0.2169 | 0.5164 | 0.675 |
| Divorced/widowed | 0.1291 | 0.7614 | 0.865 |
| Education (%) |  |  |  |
| None | Ref | Ref | Ref |
| Primary | -0.5028 | 0.4182 | 0.229 |
| Junior secondary | 0.4232 | 0.5206 | 0.416 |
| Senior secondary | 0.2472 | 0.4873 | 0.612 |
| Higher | -0.5028 | 0.4182 | 0.246 |
| Religion (%) |  |  |  |
| Muslim | -1.2361 | 0.7142 | 0.084 |
| Primary language spoken at home (%) |  |  |  |
| Hausa | Ref | Ref | Ref |
| Yoruba | -0.7247 | 0.6356 | 0.254 |
| English/Pigeon English | -0.0215 | 0.4197 | 0.959 |
| Other | 0.0997 | 0.4660 | 0.831 |
| Household asset score | 0.0001 | 0.0985 | 0.999 |
