# Supplementary material for: On the sustainability of a family planning program in Nigeria when funding ends
Source: PLoS One. 2019 Sep 26;14(9):e0222790. doi: 10.1371/journal.pone.0222790 (PMC6762171; doi:10.1371/journal.pone.0222790)
Supplement: S1 File — (PDF) [file pone.0222790.s004.pdf]

\+OFFICE USE ONLY: QUESTIONNAIRE NO: [__|__|__|__|__|__|__|__|__||__|__]
(Baseline cluster code 4 digits + HH number 3 digits + household division number 2 digits + Respondent line number 2 digits)

MLE URBAN NIGERIAN SURVEY 2014
WOMAN QUESTIONNAIRE: HAUSA

BASELINE STATE NAME & CODE ____________________

BASELINE CITY NAME & CODE____________________
(Abuja=1, Benin=2, Ibadan=3, Ilorin=4, Kaduna=5, Zaria=6)

BASELINE LGA NAME & CODE ____________________

BASELINE CLUSTER NAME & CODE ____________________

BASELINE HOUSEHOLD NUMBER ……………………………………………

RESPONDENT MOVED FROM PREVIOUS (LAST KNOWN) LOCATION?…………………
(1=Yes, 2=No)

END LINE CITY NAME & CODE ____________________
(Abuja=1, Benin=2, Ibadan=3, Ilorin=4, Kaduna=5, Zaria=6)

END LINE LGA NAME & CODE ____________________

HOUSEHOLD DIVISION NUMBER……………………………………………
(*OFFICE USE ONLY*)

BASELINE LINE NUMBER OF WOMAN……………………………………………

NAME OF RESPONDANT____________________
[SURNAME, GIVEN NAME]

ADDRESS OF HOUSEHOLD

INTERVIEWER VISITS

**VISIT No.**	**1**	**2**	**3**	**FINAL VISIT**
DATE	DAY/ MONTH/ YEAR [____/_____/_14_]	DAY/ MONTH/ YEAR [____/_____/__14_]	DAY/ MONTH/ YEAR [____/_____/__14_]	DAY [____|____] MONTH [____|____] YEAR [_2_|_0_|_1_|_4_]
INTERVIEWER'S NAME	____________	____________	____________	____________
INTERVIEWER CODE				
RESULT*				
**NEXT VISIT: DATE**	[____/_____/14_]	[____/_____/14_]		TOTAL NO. OF VISITS
**TIME**	H H M M	H H M M		

***RESULT CODES:**
1. COMPLETED
2. NOT AT HOME
3. POSTPONED
4. REFUSED
5. PARTLY COMPLETED
6. INCAPACITATED
7. OTHER ______________________(Specify)

LANGUAGE

MAIN LANGUAGE OF INTERVIEW TRANSLATOR USED? YES = 1 NO = 2
LANGUAGE CODES: HAUSA =1 YORUBA =2 ENGLISH = 3 PIDGIN =4 OTHERS = 6 (Specify______________)

SUPERVISOR	FIELD EDITOR	OFFICE EDITOR	KEYED BY
NAME……………………………… CODE: [| |] DATE [__ / ___/ 14_] DD MM YY	NAME………………………… CODE: [| |] DATE [__ / __/ 14_] DD MM YY	NAME………………………………. CODE: [|] DATE [__ / ___/ 14_] DD MM YY	NAME…………………………………………… CODE: [|] DATE [__/ __/ 14_] DD MM YY

Individual Consent: Woman's Survey on Family Planning

Introduction

Hello! My name is _________________________________; I am part of a research team working for the National Population Commission (NPC) under the Measurement, Learning and Evaluation (MLE) Project for the Nigerian Urban Reproductive Health Initiative (NURHI). In the last four years, we visited this household and spoke to you about this study. We may have also spoken to other women that live in this household or were visiting at that time. We will be asking questions to all women who participated in this study in the last 4 years. Your participation in this study will help to improve family planning services in this city.

Barka da war haka! Sunana ---------------------------------------Ina daya daga cikin mutane masu yiwa Hukumar Kidaya ta Kasa bincike Muna gudanar da bincike kan hanyar tsarin iyali a biranen Najeriya.A shekaru 4 da suka shige,mun ziyarci wannan gidan kuma mun yi maku magana gameda wannan binciken kuma maiyiwa mun yi ma wasu mata masu zama nan gidan magana tareda wadanda suka kawo maku ziyara a wannan gidan. Zamu sake yi wa dukkan matan da muka yi wa tambayoyi tambaya a wannan binciken. Kasancewarki cikin wannan bincike zai taimaka a inganta harkokin tsarin iyali a wannan garin.

Explanation of Procedure

The interview will take place in or around your home, somewhere private. The interview will take about 60 minutes. I will ask you questions about your home, child birth spacing, health-care seeking, and family size decisions. You may choose not to give the interview, or not to answer a question for any reason. You can stop the interview at any time by telling me that you want to stop it. If you decide not to give the interview or not to answer a question, no harm will come to you, and there will be no effect on your access to health services in the future.

Tambayoyin zasu kasance a gidanki ko kusa da gidanki ko wani wuri kebabbe.Tambayoyin zasu dauki kusan minti 60.Zan yi miki tambayoyi game da gidanki,hanyar tsarin iyali, neman kiwon lafiya da kuma yanke hukunci kan yawan iyali.
Ki na damar kin amsa tambayoyi, ko ki ki amsa wata tambaya saboda wani dalili. Za ki iya tsaida tambayoyi a kowanne lokaci ki ka fada mini ba ki son ki cigaba.Idan ba ki son shiga ko ba ki son amsa wata tambaya, babu wani abu da zai same ki, kuma ba zai shafi damar gidanki na samun harkokin kiwon lafiya nan gaba ba.

Confidentiality

Your answers will not be shared with anyone outside this research project. Your name will not appear on the survey. We will not share answers with community members, health providers, family or anyone else. At the end of the study, we will put all the answers together and make a report.

Your contact information will be kept in a locked cabinet, and kept apart from your answers to the questions. Once the study is finished, the list of names with your contact information, and the completed surveys will be destroyed.

Ba za'a fadawa wani wanda ba ya cikin wannan binciken amsoshinki ba.Duk tambayoyi za'a yisu a sirri. Kuma sunanki ba zai fito a binciken ba.Amsoshinki ba za mu fadawa jama'ar al'ummarki ba, ko masu bada kiwon lafiya, ko iyalanki ko kuma wani daban. A karshen wannan binciken, za mu sa duk amsoshinku a wuri daya kuma mu rubuta rahoto.
Zamu adana dukkan hanyoyin da za'a iya samunki,kuma zamu adana dukkan amsoshinki. A lokacin da muka kammala wannan bincike, sunayen da muka rubuta da hanyoyin da za'a iya samunki da takardun da aka yi maki tambayoyi duk za'a lalata su.

Benefits

Research helps society by providing new knowledge. You may not benefit directly from this survey. However, your answers will be important for planning better programs to make sure women can access the health care they need.

Bincike na taimaka wa al'umma da sanin sababbin abubuwa, Ba lalle ne ki amfana kai tsaye da wannan binciken ba. Koda yake amsoshinki za su kasance masu muhimmanci wajen zana kyawawan shirye-shiryen da za su baiwa mata damar samun inganta kiwon lafiyarsu.

Risks and Discomfort

There is the possibility you may feel uncomfortable about a question I ask. If you feel uncomfortable about any of the questions, you do not have to answer them. I can skip those questions and go on to the next section. You can end the interview at any time. There is also the possibility that someone may approach us during the interview to find out what we are discussing. We intend to do this interview in private, if someone approaches us, we will stop the interview until we can continue in private. Some questions may not apply to you, but the interviewer must ask the same questions of everyone.

Babu mamaki ki ji babu dadi game da tambayoyin da na ke yi, idan ba ki ji dadin ko wacce tambaya ba, ba lalle sai kin amsa ba. Zan iya tsallakewa zuwa tambayoyi na gaba.Kina da damar ki tsaida ni daga yin tambayoyi duk lokacin da ki ke so. Kuma babu mamaki wani yazo wurinmu lokacin amsa tambayoyi don ya san abinda muke tattaunawa.Munyi niyyar muyi wadannan tambayoyin a sirrance saboda haka idan wani yazo zami iya tsaida tambayoyin har sai mun kebe mu biyu. Wasu tambayoyin ba su shafe ki ba,amma kuma mai tambaya dole ya tambayi kowa tambaya iri daya.

Costs and Payment Individual Consent:

There are no costs for being in this study. You will not receive any compensation for taking part in this study.

Babu wani biyan kudi don an shiga wannan binciken.Ba za ki karbi wani kudi ba domin kin shiga wannan binciken.

Questions /Your rights as Participants

This study has been approved by the National Health Research Ethics Committee of Nigeria (NHREC) (assigned no. NHREC/01/01/2007, approved duration from 30/04/2014 to 29/04/2015) and the University of North Carolina (USA). If you have any questions about this study or the results, you can contact the following: The study coordinator, Mr. Bolaji Akinsulie at the National Population Commission at 08055515054 or the Institutional Review Board at the University of North Carolina at +1 919-966-3113. You have the right to ask, and have answers, to any

questions you may have about this research. If you have questions or concerns, you should contact the researchers listed above, or ask me before or after the interview. Do you have any questions now?

An sami amincewar wannan binciken daga Ma'aikatar Kiwon Lafiya da kula da binciken ladubba ta Najerya da Jami'ar Arewacin Carolina (Amurka). Idan ki na da tambaya game da wannan binciken ko sakamakonsa za ki iya tuntubar wadannan: Kodinetan binciken , Mr Bolaji Babatunde Akinsulie a Hukumar Kidaya ta Kasa (08055515054) ko Hukumar Makarantar Nazari ta Jami'ar Arewacin Carolina a +1919-966-3113.
Ki na da 'yancin yin tambaya kuma ki samu amsar tambayarki, duk tambayar da ki ke so a wannan binciken.Idan ki na da tambayoyi ko damuwa kina iya tumtubar masu binciken da a ka lissafa a sama, ko ki tambaye ni kafin ko bayan na gama miki tambayoyi.Yanzu ki na da wasu tambayoyi da za kiyi mini?

Consent

Now, can you tell me if you agree to participate in this research? If you say yes, it means that you have agreed to be part of the study.

Yanzu, kina iya fadi mani idan kin amince ki kasance a wannan binciken? Idan kika ce kin yadda, shine ke nufin cewa kin amince ki kasance a binciken.

May I begin the interview now?

Zan iya fara tambayarki yanzu?

Yes ………………………1 → **CONTINUE**

No …………………………2 → **THANK RESPONENT AND END INTERVIEW**

Would you like a copy of this document?

Interviewer's signature... **DATE [____/_____/2014]**

SECTION 1: BACKGROUND CHARACTERISTICS			
**Qno**	**Questions and filters**	**Coding categories**	**Skip to**
	RECORD START TIME:	HOUR……………………………….[__|__]<br>MINUTES…………………………..[__|__]	
Thank you for agreeing to participate in this survey. As I mentioned in asking for your consent, we are going to looking to assess your health and information needs. To begin, we are going to ask you some background questions about yourself.<br><br>***Nagode da amincewar ki a wannan binciken. Kamar yadda na fada miki wajen neman izininki, muna neman bayanai akan lafiyar ki. Da farko, zamu tambaye ki wasu mahimman abubuwa game da ke kanki.***			
Q101	In what month and year were you born?<br><br>***A wanne wata da shekara aka haife ki?***	MONTH . . . .……[__|__]<br>DON'T KNOW MONTH . . . . 98<br>YEAR . . . .…..[__|__][__|__]<br>DON'T KNOW YEAR 9998	
Q102	How old were you on your last birthday?<br><br>***Shekarunki nawa cikakku?***<br><br>COMPARE AND CORRECT Q101 AND/OR Q102 IF INCONSISTENT	AGE IN COMPLETED YEARS….….[__|__]	
Q103	Have you ever attended school formal or Qur'anic?<br><br>***Kin ta ba shiga makarantar boko ko ta Alo (Qur'ani)?***	YES……………………………….......................1<br>NO …………………………………………….…...2 →	 **Q107**
Q104	What is the highest level of school you **attended:** Quranic only, primary, junior secondary, senior secondary, or higher?<br><br>***Mene ne zurfin ilmin ki: Makarantar Alo (Al qur'ani) kadai, firamare, karamar sakandare, babbar sakandare ko gaba da sakandare?***	QURANIC ONLY………………………...............0 →<br>PRIMARY……………………………….……………1<br>JUNIOR SECONDARY (JSS)…….................... ….2<br>SENIOR SECONDARY (SSS)…….........................3<br>HIGHER……………………….…..…....................4 →	**Q107**<br><br><br><br>**Q109**
Q105	What is the highest (class/year) you completed at that level?<br><br>***Aji nawa ne mafi zurfi da ki ka kammala a wannan matsayin?***<br><br>IF NO YEAR COMPLETED, WRITE "00"	CLASS/YEAR……………………[__|__]	
Q106	CHECK 104:<br><br>PRIMARY (CODE 1) [ ] ↓	JSS OR SSS (CODE 2 OR 3) [ ] →	**Q109**
Q107	Now I would like you to read a sentence to me, but please tell me, what language you are most comfortable with?<br><br>**Yanzu ina son ki karanta mani wannan sharaddar, amma kafin ki karanta mani wanne harshe kika fi kwarancewa da shi?**<br><br>CIRCLE CODE FOR LANGUAGE SELECTED AND HAND THE RESPONDENT A LITERACY CARD IN THE SELECTED LANGUAGE	HAUSA……………………………………….. …01<br>YORUBA…………………………................ …02<br>IGBO………………………………………………03<br>ENGLISH…………………………………………04<br>PIDGIN ENGLISH……………………………….05<br>RESPONDENT CANNOT<br>READ…………………………………………..... 11 →<br>BLIND/VISUALLY IMPAIRED……………….. ..21 →<br>OTHER______________________________ 96<br>(SPECIFY)	 <br><br><br><br><br>**Q109**<br>**Q109**
Q108	Now I would like you to read this sentence to me.<br><br>**Yanzu ina son ki karanta mani wannan sharaddar?**<br><br>SHOW A SENTENCE FROM THE LITERACY CARD TO THE RESPONDENT<br>IF RESPONDENT CANNOT READ THE WHOLE SENTENCE, PROBE:<br>Can you read any part of the sentence to me?	CANNOT READ AT ALL……………………….. 1<br>ABLE TO READ ONLY PARTS OF<br>SENTENCE . . . .…………... 2<br>ABLE TO READ WHOLE<br>SENTENCE………………………...………….…. 3<br>NO CARD WITH REQUIRED<br>LANGUAGE………………………………………. 4	
Q109	What is your religion?<br><br>***Menene addinin ki?***	CATHOLIC………………………………………. 1<br>PROTESTANT/OTHER<br>CHRISTIAN……………………...……………… 2<br>MUSLIM…………….……………...…………..… 3<br>NO RELIGION …………………..….…………… 5 →<br>OTHER________________________________ 6<br>(SPECIFY)	 <br><br><br>**Q112**

Q110	How religious do you consider yourself? Do you consider yourself …<br><br>READ OUT RESPONSES<br><br>***Yaya ki ka dauki kanki a addinance? Kin dauki kan ki mai yin addini sosai, mai yin addini daidai gwargwado ko ba mai yin addini ba…..***	STRONGLY RELIGIOUS ……….……………… 1<br>SOMEWHAT RELIGIOUS ……….…………….. 2<br>NOT AT ALL<br>RELIGIOUS…….…………….…...………………. 3	
Q111	To what degree does your religion influence the decisions you make about family planning? Would you say that your beliefs: never, somewhat, often, or always influence the decisions you make about family planning<br><br>***A wanne matsayi ne addiniki yake shafar kudurin da kike yi game da tsarin iyali? Zaki iya cewa addininki baya shafar kudurin da ki ke yi game da tsarin iyali,yana shafar kudurin ki ko da yaushe/ ko sa' i sa' i .***	NEVER……………………………..…1<br>SOMEWHAT……..…………………..…2<br>OFTEN/FREQUENTLY…………..…3<br>ALWAYS…………………………...…4<br>DON'T KNOW (ABOUT FP)……..…8	
Q112	What language do you usually speak at home?<br><br>***Wanne harshe/yarei ki ka fi amfani dashi a gida?***	HAUSA……………………….………1<br>YORUBA……………….……………2<br>IGBO…………………….….………3<br>ENGLISH…………………….…….. 4<br>PIDGIN ENGLISH………………….5<br>OTHER_____________________ 6<br>(SPECIFY)	
Q113	Outside your home, what language do you speak the most?<br><br>***Idan kin fita daga gida, wanne harshe/yarei kika fi magana da shi ko da yaushe?***	HAUSA…………………………….…1<br>YORUBA……………….……..……2<br>IGBO……………………………..……3<br>ENGLISH……………………………..4<br>PIDGIN ENGLISH…………………..…5<br>OTHER_____________________6<br>(SPECIFY)	

SECTION 2: REPRODUCTION

QNo.	Questions and filters	Coding categories	Skip to
	Now I would like to ask you about all the births you have had during your life. Please be as honest as possible and know that your answers will not be shared with anyone else.<br><br>***Yanzu ina so nayi miki tambayoyi game da dukka haife- haife da ki ka yi a rayuwarki. Ki yi iyakar kokari ki gaya mini gaskiya, kuma ki sani cewa duk amsoshin ki za'a barsu a sirance ba za a gayawa kowa ba.***		
Q201	Have you ever had any live birth?<br><br>***Kin taba haihuwar dan/yar da ya/ta yazo da rai?***	YES……………………………………1<br>NO……………………………………..2 →	Q206
Q202	Do you have any sons or daughters to whom you have given birth who are now living with you?<br><br>***A yanzu haka kina da 'ya'ya maza ko mata da ki ka haifa wadanda kuke tare?***	YES…………………………..……1<br>NO…………………………….………2 →	Q204
Q203	How many of these sons live with you?<br><br>And how many daughters live with you?<br><br>***'Ya'yanki maza nawa ne kuke tare?***<br><br>***Da kuma 'ya'ya mata nawa ne kuke tare?***	SONS AT HOME……………… [__|__]<br><br>DAUGHTERS AT HOME………[__|__]	
Q204	Do you have any sons or daughters to whom you have given birth who are alive but do **not** live with you?<br><br>***Kina da 'ya'ya maza ko mata wadanda ki ka haifa, suna raye amma ba kwa tare dasu?***	YES……………………………………..1<br>NO…………………………..……………2 →	Q206
Q205	How many sons are alive but not living with you?<br><br>And how many daughters are alive but not living with you?<br><br>***'Ya'yan ki maza nawa ne ke raye amma ba kwa tare?***<br>***Da kuma 'ya'ya mata nawa ne ke raye amma ba kwa tare?***	SONS ELSEWHERE………………….[__|__]<br><br>DAUGHTERS ELSEWHERE………. [__|__]	
Q206	Sometimes it happens that children die. It may be painful to talk about and I am sorry to ask you about painful memories, but it is important to get correct information. Have you given birth to a boy or a girl who was born alive but later died?<br><br>***Wani lokaci yana faruwa cewa yara na mutuwa. Kiyi hakuri inna tuna miki da abun da yariga ya wuce, amma ya kamata mu samu ainihin bayanai.Kin taba haihuwan 'da namiji ko'ya mace wanda ya/ta zo da rai amma daga baya ya/ta rasu?***<br><br>IF NO, PROBE: Any baby who cried or showed signs of life but did not survive?<br>***Akwai dan da yayi kuka, ko ya nuna alamar rai amma daga baya ya rasu?***	YES……………………………………..1<br><br>NO…………………………..……………2 →	Q208
Q207	How many boys have died?<br><br>And how many girls have died?<br><br>**'Ya'ya maza nawa ne suka rasu?**<br><br>***Da kuma ya'ya mata nawa ne suka rasu?***	BOYS DEAD…………….……………. [__|__]<br><br>GIRLS DEAD…………………….……. [__|__]	
Q208	SUM ANSWERS TO 203, 205, AND 207, AND ENTER TOTAL.<br><br>If none, record 00.	TOTAL……………………………..………[__|__]	
Q209	CHECK 208:<br><br>Just to make sure that I have this right: you have had in TOTAL _______ birth. Is that correct?<br><br>***Dan tabbatar da ganin banyi kuskure ba, jimilar (dukka) 'ya'yan da ki ka haifa ……Hakane?***<br><br>YES ☐ ↓ NO ☐ → PROBE AND CORRECT 201-208 as necessary.		

Q210	CHECK Q208:<br><br>YES, HAS ONE OR MORE BIRTHS ☐<br>↓<br>Q211	NO BIRTHS ☐ →	**Q233**

BIRTH HISTORY

Now I would like to record the names of all your births whether still alive or not, starting with the first one you had.
Yanzu Ina son in rubuta sunayen dukan ;ya'yan da kika haifa da wadanda ke raye hadi da wadanda suka mutu. Mu fara da haihuwar ki ta farko,

RECORD NAMES OF **ALL** THE **LIVE** BIRTHS in Q212. RECORD TWINS AND TRIPLETS ON SEPARATE LINES. IF THERE ARE MORE THAN 18 BIRTHS, USE AN ADDITIONAL QUESTIONNAIRE. STARTING WITH THE SECOND ROW. CIRCLE LINE NUMBER AND NAME OF ALL CHILDREN BORN IN JANUARY 2012 OR LATER.

(Q211)<br>Line no.	(Q212)<br>What name was given to your baby?<br><br>***Wanne suna a ka ba jaririn?***	(Q213)<br>Were any of these births twins/triplets?<br><br>***Wannan haihuwar tagwaye ne ko 'yan uku?***	(Q214)<br>Is (NAME) a boy or a girl?<br><br>***Shin (Suna) namiji ne ko mace?***	(Q215)<br>In what month and year was (NAME) born? PROBE: What is her/his birthday? (other probes: what season was it)<br><br>***Wanne wata ne da shekara ki ka haifi (SUNA)?***	(Q216)<br>Is (NAME) still alive?<br><br>***Shin (SUNA) ya/ta na raye?***	(Q217)<br>IF ALIVE:<br>How old is (NAME) currently?<br><br>RECORD AGE IN COMPLETED YEARS<br><br>IF LESS THAN 1 YEAR, RECORD "00".<br><br>***Idan 'dan/'yar ta/ya na raye, Shekarun (SUNA) nawa ne yanzu?***	(Q218)<br>IF ALIVE:<br>Is (Name) living with you?<br><br>**SUNA) ya/ta na zama tare da ke?**	(Q219)<br>**IF DEAD:**<br>How old was (NAME) when she/he died?<br>IF LESS THAN 2 YEARS, PROBE:<br>***Shekarun (SUNA) nawa ne kafin rasuwar sa/ta?***<br><br>How many months old was (NAME)?<br>***Watannin (SUNA) nawa ne kafin rasuwar sa/ta?***<br><br>IF LESS THAN 1 MONTH OLD, RECORD DAYS; MONTHS IF LESS THAN TWO YEARS; OR YEARS IF 2 OR MORE YEARS OLD	(Q220)<br>Were there any other live births between (NAME OF PREVIOUS BIRTH) and (NAME), including any children who died after birth?<br><br>***Akwai wata haihuwa mai rai a tsakanin (NAME OF PREVIOUS BIRTH) da (NAME), Harda 'ya'yan da suka rasu bayan haihuwa?***
01	NAME:<br>______________________	SING……1<br>MULT…..2	BOY…1<br>GIRL...2	MONTH [__|__]<br>YEAR [__|__][__|__]	YES….1<br>NO……2 → **Q219**	AGE IN YEARS<br>[__|__]	YES….1<br>NO……2<br>**ALL SKIP TO (02)**	DAYS……..1 [__|__]<br>MONTHS…2 [__|__]<br>YEARS……3 [__|__]<br>DK………..…….998	
02	NAME:<br>______________________	SING……1<br>MULT…..2	BOY…1<br>GIRL...2	MONTH [__|__]<br>YEAR [__|__][__|__]	YES….1<br>NO……2 → **Q219**	AGE IN YEARS<br>[__|__]	YES….1<br>NO……2<br>**ALL SKIP TO Q220**	DAYS……..1 [__|__]<br>MONTHS…2 [__|__]<br>YEARS……3 [__|__]<br>DK…………..….998	YES…1 → ADD BIRTH<br>NO....2 → NEXT BIRTH
03	NAME:<br>______________________	SING……1<br>MULT…..2	BOY…1<br>GIRL...2	MONTH [__|__]<br>YEAR [__|__][__|__]	YES….1<br>NO……2 → **Q219**	AGE IN YEARS<br>[__|__]	YES….1<br>NO……2<br>**ALL SKIP TO Q220**	DAYS……..1 [__|__]<br>MONTHS…2 [__|__]<br>YEARS……3 [__|__]<br>DK…………..….998	YES…1 → ADD BIRTH<br>NO....2 → NEXT BIRTH
04	NAME:<br>______________________	SING……1<br>MULT…..2	BOY…1<br>GIRL...2	MONTH [__|__]<br>YEAR [__|__][__|__]	YES….1<br>NO……2 → **Q219**	AGE IN YEARS<br>[__|__]	YES….1<br>NO……2<br>**ALL SKIP TO Q220**	DAYS……..1 [__|__]<br>MONTHS…2 [__|__]<br>YEARS……3 [__|__]<br>DK…………..….998	YES…1 → ADD BIRTH<br>NO....2 → NEXT BIRTH

(Q211)<br>Line no.	(Q212)<br>What name was given to your baby?<br>***Wanne suna a ka ba jaririn?***	(Q213)<br>Were any of these births twins/triplets?<br>***Wannan haihuwar tagwaye ne ko 'yan uku?***<br>CIRCLE	(Q214)<br>Is (NAME) a boy or a girl?<br>***Shin (Suna) namiji ne ko mace?***<br>**CIRCLE**	(Q215)<br>In what month and year was (NAME) born?<br>PROB***E: Wanne wata ne da shekara ki ka haifi (SUNA)?***<br>PROBE: What is her/his birthday?<br>(other probes: what season was it)<br>***PROBE: Yaushe ne ranar haihuwar sa/ta?***<br>(***Other probes: A wane lokaci (damina ko rani)?***	(Q216)<br>Is (NAME) still alive?<br>***Shin (SUNA) ya/ta na raye?***	(Q217)<br>IF ALIVE:<br>How old is (NAME) currently?<br>RECORD AGE IN COMPLETED YEARS<br>IF LESS THAN 1 YEAR, RECORD "00".<br>***Idan 'dan/'yar ta/ya na raye, Shekarun (SUNA) nawa ne yanzu?***	(Q218)<br>IF ALIVE:<br>Is (Name) living with you?<br>**SUNA) ya/ta na zama tare da ke?**	(Q219)<br>**<u>IF DEAD:</u>**<br>How old was (NAME) when she/he died?<br>IF LESS THAN 2 YEARS, PROBE:<br>***Shekarun (SUNA) nawa ne kafin rasuwar sa/ta?***<br>How many months old was (NAME)?<br>***Watannin (SUNA) nawa ne kafin rasuwar sa/ta?***<br>IF LESS THAN 1 MONTH OLD, RECORD DAYS; MONTHS IF LESS THAN TWO YEARS; OR YEARS IF 2 OR MORE YEARS OLD	(Q220)<br>Were there any other live births between (NAME OF PREVIOUS BIRTH) and (NAME), including any children who died after birth?<br>***Akwai wata haihuwa mai rai a tsakanin (NAME OF PREVIOUS BIRTH) and (NAME), harda 'ya'yan da suka rasu bayan haihuwa?***
05	NAME:<br>______________________________	SING……1<br>MULT…..2	BOY…1<br>GIRL...2	MONTH [__|__]<br>YEAR [__|__][__|__]	YES….1<br>NO……2<br>↓<br>**Q219**	AGE IN YEARS<br>[__|__]	YES….1<br>NO……2<br>**ALL SKIP TO Q220**	DAYS……..1 [__|__]<br>MONTHS…2 [__|__]<br>YEARS……3 [__|__]<br>DK…………...….998	YES…1→ ADD BIRTH<br>NO....2→ NEXT BIRTH
06	NAME:<br>______________________________	SING……1<br>MULT…..2	BOY…1<br>GIRL...2	MONTH [__|__]<br>YEAR [__|__][__|__]	YES….1<br>NO……2<br>↓<br>**Q219**	AGE IN YEARS<br>[__|__]	YES….1<br>NO……2<br>**ALL SKIP TO Q220**	DAYS……..1 [__|__]<br>MONTHS…2 [__|__]<br>YEARS……3 [__|__]<br>DK……….…….998	YES…1→ ADD BIRTH<br>NO....2→ NEXT BIRTH
07	NAME:<br>___________________________	SING……1<br>MULT…..2	BOY…1<br>GIRL...2	MONTH [__|__]<br>YEAR [__|__][__|__]	YES….1<br>NO……2<br>↓<br>**Q219**	AGE IN YEARS<br>[__|__]	YES….1<br>NO……2<br>**ALL SKIP TO Q220**	DAYS……..1 [__|__]<br>MONTHS…2 [__|__]<br>YEARS……3 [__|__]<br>DK…………...….998	YES…1→ ADD BIRTH<br>NO....2→ NEXT BIRTH
08	NAME:<br>______________________________	SING……1<br>MULT…..2	BOY…1<br>GIRL...2	MONTH [__|__]<br>YEAR [__|__][__|__]	YES….1<br>NO……2<br>↓<br>**Q219**	AGE IN YEARS<br>[__|__]	YES….1<br>NO……2<br>**ALL SKIP TO Q220**	DAYS……..1 [__|__]<br>MONTHS…2 [__|__]<br>YEARS……3 [__|__]<br>DK…………...….998	YES…1→ ADD BIRTH<br>NO....2→ NEXT BIRTH
10	NAME:<br>______________________________	SING……1<br>MULT…..2	BOY…1<br>GIRL...2	MONTH [__|__]<br>YEAR [__|__][__|__]	YES….1<br>NO……2<br>↓<br>**Q219**	AGE IN YEARS<br>[__|__]	YES….1<br>NO……2<br>**ALL SKIP TO Q220**	DAYS……..1 [__|__]<br>MONTHS…2 [__|__]<br>YEARS……3 [__|__]<br>DK…………...….998	YES…1→ ADD BIRTH<br>NO....2→ NEXT BIRTH

(Q211) Line no.	(Q212) What name was given to your baby? ***Wanne suna a ka ba jaririn?***	(Q213) Were any of these births twins/triplets? ***Wannan haihuwar tagwaye ne ko 'yan uku?*** CIRCLE	(Q214) Is (NAME) a boy or a girl? ***Shin (Suna) namiji ne ko mace?*** **CIRCLE**	(Q215) In what month and year was (NAME) born? PROBE: What is her/his birthday? (other probes: what season was it) ***Wanne wata ne da shekara ki ka haifi (SUNA)? PROBE: Yaushe ne ranar haihuwar sa/ta? (Other probes: A wane lokaci (damina ko rani)?***	(Q216) Is (NAME) still alive? ***Shin (SUNA) ya/ta na raye?***	(Q217) IF ALIVE: How old is (NAME) currently? RECORD AGE IN COMPLETED YEARS IF LESS THAN 1 YEAR, RECORD "00". ***Idan 'dan/'yar ta/ya na raye, Shekarun (SUNA) nawa ne yanzu?***	(Q218) IF ALIVE: Is (Name) living with you? **SUNA) ya/ta na zama tare da ke?**	(Q219) **IF DEAD:** How old was (NAME) when she/he died? IF LESS THAN 2 YEARS, PROBE: ***Shekarun (SUNA) nawa ne kafin rasuwar sa/ta?*** How many months old was (NAME)? ***Watannin (SUNA) nawa ne kafin rasuwar sa/ta?*** IF LESS THAN 1 MONTH OLD, RECORD DAYS; MONTHS IF LESS THAN TWO YEARS; OR YEARS IF 2 OR MORE YEARS OLD	(Q220) Were there any other live births between (NAME OF PREVIOUS BIRTH) and (NAME), including any children who died after birth? ***Akwai wata haihuwa mai rai a tsakanin (NAME OF PREVIOUS BIRTH) and (NAME), Harda 'ya'yan da suka rasu bayan haihuwa?***
11	NAME: ______________	SING……1<br>MULT…..2	BOY…1<br>GIRL...2	MONTH [__|__]<br>YEAR [__|__][__|__]	YES…..1<br>NO……2 → **Q219**	AGE IN YEARS<br>[__|__]	YES….1<br>NO……2<br>**ALL SKIP TO Q220**	DAYS……..1 [__|__]<br>MONTHS…2 [__|__]<br>YEARS …..3 [__|__]<br>DK……………..…998	YES…1 → ADD BIRTH<br>NO....2 → NEXT BIRTH
12	NAME: ______________	SING……1<br>MULT…..2	BOY…1<br>GIRL...2	MONTH [__|__]<br>YEAR [__|__][__|__]	YES…...1<br>NO…….2 → **Q219**	AGE IN YEARS<br>[__|__]	YES….1<br>NO……2<br>**ALL SKIP TO Q220**	DAYS……..1 [__|__]<br>MONTHS…2 [__|__]<br>YEARS …..3 [__|__]<br>DK……………..…998	YES…1 → ADD BIRTH<br>NO....2 → NEXT BIRTH
13	NAME: ______________	SING……1<br>MULT…..2	BOY…1<br>GIRL...2	MONTH [__|__]<br>YEAR [__|__][__|__]	YES…...1<br>NO…….2 → **Q219**	AGE IN YEARS<br>[__|__]	YES….1<br>NO……2<br>**ALL SKIP TO Q220**	DAYS……..1 [__|__]<br>MONTHS…2 [__|__]<br>YEARS …..3 [__|__]<br>DK……………..…998	YES…1 → ADD BIRTH<br>NO....2 → NEXT BIRTH
14	NAME: ______________	SING……1<br>MULT…..2	BOY…1<br>GIRL...2	MONTH [__|__]<br>YEAR [__|__][__|__]	YES…...1<br>NO…….2 → **Q219**	AGE IN YEARS<br>[__|__]	YES….1<br>NO……2<br>**ALL SKIP TO Q220**	DAYS……..1 [__|__]<br>MONTHS…2 [__|__]<br>YEARS …..3 [__|__]<br>DK……………..…998	YES…1 → ADD BIRTH<br>NO....2 → NEXT BIRTH
15	NAME: ______________	SING……1<br>MULT…..2	BOY…1<br>GIRL...2	MONTH [__|__]<br>YEAR [__|__][__|__]	YES…...1<br>NO…….2 → **Q219**	AGE IN YEARS<br>[__|__]	YES….1<br>NO……2<br>**ALL SKIP TO Q220**	DAYS……..1 [__|__]<br>MONTHS…2 [__|__]<br>YEARS …..3 [__|__]<br>DK……………..…998	YES…1 → ADD BIRTH<br>NO....2 → NEXT BIRTH

(Q211) Line no.	(Q212) What name was given to your baby? ***Wanne suna a ka ba jaririn?***	(Q213) Were any of these births twins/triplets? ***Wannan haihuwar tagwaye ne ko 'yan uku?*** CIRCLE	(Q214) Is (NAME) a boy or a girl? ***Shin (Suna) namiji ne ko mace?*** CIRCLE	(Q215) In what month and year was (NAME) born? PROBE: What is her/his birthday? (other probes: what season was it) ***Wanne wata ne da shekara ki ka haifi (SUNA)?*** ***PROBE: Yaushe ne ranar haihuwar sa/ta?*** (***Other probes: A wane lokaci (damina ko rani)?***	(Q216) Is (NAME) still alive? ***Shin (SUNA) ya/ta na raye?***	(Q217) IF ALIVE: How old is (NAME) currently? RECORD AGE IN COMPLETED YEARS IF LESS THAN 1 YEAR, RECORD "00". ***Idan 'dan/'yar ta/ya na raye, Shekarun (SUNA) nawa ne yanzu?***	(Q218) IF ALIVE: Is (Name) living with you? **SUNA) ya/ta na zama tare da ke?**	(Q219) IF DEAD: How old was (NAME) when she/he died? IF LESS THAN 2 YEARS, PROBE: ***Shekarun (SUNA) nawa ne kafin rasuwar sa/ta?*** How many months old was (NAME)? ***Watannin (SUNA) nawa ne kafin rasuwar sa/ta?*** IF LESS THAN 1 MONTH OLD, RECORD DAYS; MONTHS IF LESS THAN TWO YEARS; OR YEARS IF 2 OR MORE YEARS OLD	(Q220) Were there any other live births between (NAME OF PREVIOUS BIRTH) and (NAME), including any children who died after birth? ***Akwai wata haihuwa mai rai a tsakanin (NAME OF PREVIOUS BIRTH) and (NAME), harda 'ya'yan da suka rasu bayan haihuwa?***
16	NAME: ______________________	SING……1<br>MULT…..2	BOY…1<br>GIRL...2	MONTH [__|__]<br>YEAR [__|__][__|__]	YES…...1<br>NO…….2 → **Q219**	AGE IN YEARS<br>[__|__]	YES….1<br>NO……2<br>**ALL SKIP TO Q220**	DAYS……..1 [__|__]<br>MONTHS…2 [__|__]<br>YEARS …..3 [__|__]<br>DK…………..….998	YES…1 → ADD BIRTH<br>NO....2 → NEXT BIRTH
17	NAME: ______________________	SING……1<br>MULT…..2	BOY…1<br>GIRL...2	MONTH [__|__]<br>YEAR [__|__][__|__]	YES…...1<br>NO…….2 → **Q219**	AGE IN YEARS<br>[__|__]	YES….1<br>NO……2<br>**ALL SKIP TO Q220**	DAYS……..1 [__|__]<br>MONTHS…2 [__|__]<br>YEARS …..3 [__|__]<br>DK…………..….998	YES…1 → ADD BIRTH<br>NO....2 → NEXT BIRTH
18	NAME: ______________________	SING……1<br>MULT…..2	BOY…1<br>GIRL...2	MONTH [__|__]<br>YEAR [__|__][__|__]	YES…...1<br>NO…….2 → **Q219**	AGE IN YEARS<br>[__|__]	YES….1<br>NO……2<br>**ALL SKIP TO Q220**	DAYS……..1 [__|__]<br>MONTHS…2 [__|__]<br>YEARS …..3 [__|__]<br>DK…………..….998	YES…1 → ADD BIRTH<br>NO....2 → NEXT BIRTH

Q221	Have you had any live births since the birth of (NAME OF LAST BIRTH)?<br><br>***Kin sake samun haihuwa tun haihuwar (NAME OF LAST BIRTH)?***	YES……………………………….…1 →<br><br>NO…………………………….….…..2	RECORD BIRTH(S) IN BIRTH HISTORY TABLE
Q222	Before the birth of (NAME OF FIRST BIRTH), did you have any other live births?<br><br>***Kafin haihuwar (NAME OF FIRST BIRTH), kin kara samu wata haihuwa mai rai?***	YES…………………………………1 →<br><br>NO…………………………………...2	RECORD BIRTH(S) IN BIRTH HISTORY TABLE
Q223	COMPARE Q208 WITH NUMBER OF BIRTHS IN HISTORY ABOVE AND MARK:<br>NUMBERS ARE SAME ☐ ↓ NUMBERS ARE DIFFERENT ☐ → (PROBE AND RECONCILE)<br><br>CHECK: FOR EACH BIRTH: YEAR OF BIRTH IS RECORDED. ☐<br>FOR EACH LIVING CHILD: CURRENT AGE IS RECORDED. ☐<br>FOR EACH DEAD CHILD: AGE AT DEATH IS RECORDED. ☐<br>FOR AGE AT DEATH 12 MONTHS OR 1 YEAR: PROBE TO DETERMINE EXACT NUMBER OF MONTHS. ☐		
Q224<br>**C**	FOR EACH BIRTH **SINCE JANUARY 2009**, ENTER 'B' IN THE MONTH OF BIRTH IN COLUMN 1 OF THE CALENDAR. WRITE THE NAME OF THE CHILD TO THE LEFT OF THE 'B' CODE. FOR EACH BIRTH, ASK THE NUMBER OF MONTHS THE PREGNANCY LASTED AND RECORD 'P' IN EACH OF THE PRECEDING MONTHS ACCORDING TO THE DURATION OF PREGNANCY. (NOTE: THE NUMBER OF 'P's MUST EQUAL THE NUMBER OF COMPLETED MONTHS OF PREGNANCY.)		
Q225	CHECK Q215 AND ENTER THE NUMBER OF **BIRTHS SINCE 2012**. IF NONE, RECORD "0". ☐		
Q226	CHECK Q225:<br>YES, HAS ONE OR MORE BIRTHS SINCE **January 2012** ☐ ↓ NO BIRTHS SINCE **January 2012** ☐ →		Q233
Q227	ENTER NAME AND LINE NUMBER OF YOUNGEST CHILD BORN SINCE JANUARY 2012 FROM Q211 AND Q212:<br><br>________________________[___|___]<br>NAME LINE NUMBER<br><br>LIVING ☐ ↓ DEAD ☐ →		**Q231**
Q228	Are you currently breastfeeding (NAME OF LAST CHILD)?<br><br>**Kina shayar da (NAME OF LAST CHILD) nono ne yanzu?**	YES……………………………… 1<br>NO……………………………….. 2 →	**Q231**
Q229	How many times did you breastfeed (NAME) in the last 24 hours?<br>**Kamar sau nawa kika shayar da (NAME OF LAST CHILD) nono a cikin awa 24 da ta shige?**<br>IF NUMBER IS NOT NUMERIC, PROBE FOR APPROXIMATE NUMBER.	NUMBER OF FEEDINGS……………..…[__|__]	
Q230	In the last 24 hours, did (NAME) drink anything from a bottle with a nipple or eat any foods or liquids?<br><br>**A cikin awa 24 da suka wuce, Shin (NAME) ya/ta sha wani abu daga kwalba mai bulunboti ko ya/ta ci wani abinci ko abinci mai ruwa-ruwa**?	YES……………………………… 1<br>NO……………………………….. 2<br>DON'T KNOW………………….. 8	
Q231	At the time you became pregnant with [NAME ABOVE], did you want to become pregnant <u>then</u>, did you want to wait until <u>later</u>, or did you <u>not want</u> to have any more children at all?<br><br>***A lokacin da ki ka sami cikin (SUNA), kin so dauka ne, ko kin so ki jinkirta sai zuwa gaba, ko kuma ba ki da sha'awar sake haihuwar ko kadan?***	THEN……………………………1 →<br><br>LATER………………………..…2<br><br>NOT AT ALL……………………3 →	**Q233**<br><br><br>**Q233**

Q232	How much longer would you have liked to wait?<br><br>***Har tsawon wane lokaci ki ka so ki jira?***<br><br>IF RESPONSE IS A DECIMAL FOR YEARS (2.5YRS), THEN CONVERT INTO MONTHS AND FILL IN ONLY MONTHS.	MONTHS……………………………….1 [__|__]<br>OR<br>YEARS.………………………………2 [__|__]<br>OR<br>OTHER (SPECIFY)__________________996<br>OR<br>DON'T KNOW……………..……………..998	
Q233	Are you currently pregnant?<br><br>***A yanzu haka, kina da ciki ne?***	YES……………………………… 1<br>NO……………………………….. 2 →<br>UNSURE……….…………… . 8 →	 **Q241**<br>**Q241**
Q234<br><br>**C**	How many months pregnant are you?<br><br>**Cikin na ki wata nawa ne?**<br><br>RECORD NUMBER OF COMPLETED MONTHS.<br><br>ENTER 'P'S IN THE CALENDAR IN COLUMM 1, BEGINNING WITH THE MONTH OF INTERVIEW AND FOR THE TOTAL NUMBER OF COMPLETED MONTHS.	MONTHS………………………… [__|__]	
Q235	At any time during this pregnancy, have you gone for an antenatal check up?<br><br>**Shin a yayin da kike da wanan cikin, kin je awon ciki?**	YES………………………………….…1<br><br>NO……………………………….….…2 →	 <br>**Q239**
Q236	From where or whom did you obtain (A CHECK UP) last time? PROBE: What is the name of this place/person? And where is it/he/she located?<br><br>**Daga ina ko wajen wanene ki ka samu yin awon ciki na karshe?**<br><br>**PROBE: Menene sunan wurin ko ma'aikacin? Kuma a ina ne wurin kuma ma'akacin shi/ita a ina ya/ta ke?**<br><br>WRITE NAME OF PERSON ONLY IF PROVIDER WORKS AS AN INDIVIDUAL	NAME OF FACILITY /PERSON<br>________________________________<br>________________________________<br>*CODE BOXES: OFFICE ONLY*<br>[__|__|__|__|__]<br><br>STREET<br>NAME/ADDRESS__________________<br>______________________________<br><br>LAND MARK<br>DESCRIPTION___________________	

Q237	From which type of facility did you obtain (A CHECK UP) last time?<br><br>**Daga wacce cibiyar kiwon lafiya ki ka yi awon karshe?**	**PUBLIC SECTOR**<br>GOVT HOSPITAL…………………….……11<br>WOMEN AND CHILDREN'S HOSPITAL……………………………........12<br>CHILD WELFARE CLINIC…………………13<br>GOVT. HEALTH CENTER……………........14<br>GOVERNMENT POST/DISPENSARY.....15<br>MATERNITY HOME……………......……16<br>MOBILE CLINIC……….………………........17<br>OTHER PUBLIC____________________18<br>(SPECIFY)<br><br>**PRIVATESECTOR**<br>PRIVATE HOSPITAL/CLINIC....................21<br>PRIVATE DOCTOR'S OFFICE……..……..22<br>NURSING/MATERNITY HOME……..……23<br>PHARMACY…………………..……….……24<br>PMS/CHEMIST………….………….……….25<br>MOBILE CLINIC……….……………..........26<br>CHW/TBA……………………….……….........27<br>TRADITIONAL HEALER……………….........28<br>OTHER PRIVATE___________________29<br>(SPECIFY)<br><br>**FAITH-BASED SECTOR**<br>MISSION HOSPITAL…………..............31<br>FAITH-BASED, CHURCH CLINIC…..……32<br><br>**OTHER SOURCE**<br>OTHER NGO HOSPITAL/CLINIC…………41<br>WORKSITE CLINIC………….………….…42<br>OTHER __________________________96<br>(SPECIFY)<br>DON'T KNOW . . . .………... .98	
Q238	Was this place you received your most recent antenatal visit in this city, in another city or town, or in a rural area?<br><br>**Wanne wurin ki ka samu kulawar goyon ciki na karshe, a wannan birnin ne, ko wani birnin ko garin ko kuma a wani karkara ne?**	THIS CITY/TOWN ……………………….. 1<br>ANOTHER CITY/TOWN………………….. 2<br>A RURAL AREA ………………………… 3	
Q239	At the time you became pregnant, did you want to become pregnant <u>then</u>, did you want to wait until <u>later</u>, or did you <u>not want</u> to have any more children at all?<br><br>***A lokacin da ki ka sami ciki, kin so dauka ne, ko kin so ki jinkirta sai zuwa gaba, ko kuma ba kida sha'awar sake haihuwar ko kadan?***	THEN…………………………..…...……...1 →<br><br>LATER………………………….……………..… 2<br><br>NOT AT ALL……………………………..…3 →	**Q241**<br><br><br><br>**Q241**
Q240	How much longer would you have liked to wait?<br><br>***Har tsawon wane lokaci kika so ki jira?***<br><br>IF RESPONSE IS A DECIMAL FOR MONTHS & YEARS (2.5YRS), THEN CONVERT IN MONTH AND FILL IN ONLY MONTHS.	MONTHS…………………………….…..1 [__|__]<br>OR<br>YEARS..……………………………….2 [__|__]<br>OR<br>OTHER (SPECIFY)__________________996<br>OR<br>DON'T KNOW…………………………….…..998	
Q241	When did your last menstrual period start?<br><br>***Yaushe ki ka fara ganin hailar ki ta karshe?***<br><br>________________________________<br>(DATE, IF GIVEN)<br><br>IF LESS THAN ONE DAY, CIRCLE "1" AND WRITE "00" DAYS AGO.	DAYS AGO…………………………..……1 [__|__]<br>WEEKS AGO……………………….....……2 [__|__]<br>MONTHS AGO……..……………...…… 3 [__|__]<br>YEARS AGO…………………………...……4 [__|__]<br><br>IN MENOPAUSE/HAS HAD HYSTERECTOMY……………….……………994<br>BEFORE LAST BIRTH……………………......995<br>NEVER MENSTRUATED…………………..…996<br>CANT REMEMBER…………………….…..998	

Q242	From one menstrual period to the next, are there certain days when a woman is more likely to get pregnant if she has sex?<br><br>***Shin daga haila zuwa wata hailar da akwai ranakun da mace zata iya daukan ciki idan ta yi jima'i?***	YES……………………………………1<br>NO…………………………………………2 →<br>DON'T KNOW……………………………8 →	 **Q244**<br>**Q244**
Q243	Which days are these? Would you say that this time is just before her period begins, during her period, right after her period has ended, or halfway between two periods??<br><br>***Shin wannan lokacin yana iya faruwa dab da ta fara haila ne, lokacin da take haila ne, dab da ta gama haila ne ko kuma tsakanin haila da haila?***	JUST BEFORE HER PERIOD<br>BEGINS………………………………… 1<br>DURING HER PERIOD……………… 2<br>RIGHT AFTER IT ENDS……………. 3<br>HALFWAY BETWEEN TWO<br>PERIODS ………………………………. 4<br>OTHER______________________ 6<br>(SPECIFY)<br>DON'T KNOW………………………... 8	
Q244	Do you think that a woman who is breastfeeding her baby can become pregnant?<br>**Shin a tunanin ki matar da ke shayar da nono na iya daukar ciki?**	YES . . . . ……... 1<br>NO . . . . ……… 2<br>DEPENDS……………………………… 3<br>DON'T KNOW . . . . ……… 8 →	 <br><br>**Q246**
Q245	What are the criteria in which breastfeeding is effective as a family planning / birth spacing method?<br>Anything else?<br><br>MARK ALL THAT APPLY.<br>**Wadanne matakai ne masu kyau na shayar da nono da suka dace da kaiyade iyali/tazarar haihuwa?**<br><br>**Akwai wani abu kuma?**	EXCLUSIVE BREASTFEEDING<br>(NO SUPPLEMENTS)……………. ….. A<br>EXCLUSIVE BREASTFEEDING<br>FOR UP TO 6 MONTHS……………… B<br>MENSTRUAL PERIOD HASN'T<br>RETURNED……………………………… C<br>OTHERS ______________________ X<br>(SPECIFY)<br>NO CRITERIA/NOT A METHOD ……. Y<br>DON'T KNOW………………………….. Z	

**CHECK FOR PRESENCE OF OTHERS. BEFORE CONTINUING, MAKE EVERY EFFORT TO ENSURE PRIVACY.**			
Q246	Have you ever had a pregnancy that ended in a miscarriage, abortion or stillbirth?<br><br>***Kin taba yin cikin da ki kayi barin cikin, ya zube da kanshi, ko dan ya zo ba rai?***<br><br>By miscarriage I mean a pregnancy that ended just by itself.<br>***Abun nufi da bari ina nufin Cikin da ya zube da kanshi***<br><br>By abortion, I mean a pregnancy that ended, because you did something or used something.<br>***Abun nufi da zubar da ciki, ina nufin Cikin da ya zube domin kin yi wani abu ko amfani da wani abu, misali aka cire maki.***<br><br>By still birth, I mean a baby that was born at full term and did not show any sign of life.<br>***Abun nufi da Haihuwar da dan baizo da rai ba ina nufin haihuwar da/ya daidai wata tara amma bai nuna alamar rai ba.***	YES………………………………1<br><br>NO………………………………..2 →	 <br>**Q254**
Q247	How many miscarriages have you ever had?<br><br>**Sau nawa ki ka ta ba yin bari?**	NUMBEROF MISCARRAIGES………….[__]__]	
Q248	How many abortions have you ever had?<br><br>***Sau nawa ki ka ta ba zubda ciki?***	NUMBER OF ABORTIONS……………..[__]__]	
Q249	How many still births have you ever had?<br><br>**Sau nawa kika taba haihuwa ama dan/yar bai zo da rai ba?**	NUMBER OF STILL<br>BIRTHS…………………………………….[__]__]	

0

C	PREGNANCY OUTCOME SINCE JANUARY 2009	250. Since January 2009, have you had a pregnancy that ended in a (PREGNANCY OUTCOME)? ***Tun Janaury 2009, Kin ta ba daukar cikin da***	251. How many times has this occurred since January 2009?<br><br>***Sau nawa hakan ya faru tun daga Janaury 2009?***	252. When did the last such pregnancy end?<br><br>***Yaushe irin wannan ya faru na karshe?***	253. How many months pregnant were you when the last such pregnancy ended?<br><br>***Ki na da Cikin wata nawa lokacin da irin wannan ya faru na karshe?***<br><br>ENTER 'M' FOR MISCARRIAGE, 'A' FOR ABORTION, OR 'S' FOR STILLBIRTH IN COLUMN 1 OF THE CALENDAR IN THE MONTH IN WHICH THE PREGNANCY WAS TERMINATED. ENTER 'P's FOR THE NUMBER OF COMPLETED MONTHS. THE TOTAL NUMBER OF 'P's MUST BE ONE LESS THAN THE NUMBER OF MONTHS PREGNANT AT THE TIME OF THE TERMINATION. FOLLOW THIS PROCEDURE FOR THE NUMBER OF TIMES THE EVENT HAPPENED ACCORDING TO Q248.
(01)	Stillbirth (pregnancy was full term but the baby showed no signs of life)<br><br>***Dan/yar da baizo da rai ba***	YES…..………..1<br>NO……….……2 → (02)	NUMBER OF TIMES……..[__|__]	MONTH…….[__|__]<br><br>YEAR…..[__|__|__|__].	MONTHS…….[__|__]
(02)	Miscarriage (pregnancy ended just by itself)<br><br>***ya zube da kanshi***	YES…..………..1<br>NO……….……2 → (03)	NUMBER OF TIMES……..[__|__]	MONTH…………….[__|__]<br><br>YEAR………..[__|__|__|__]	MONTHS…….[__|__]
(03)	Abortion (pregnancy ended by something you did or used, e.g. manual vacuum aspiration)<br><br>***ya zube domin kin yi wani abu ko amfani da wani abu, misali aka cire maki.***	YES…………....1<br>NO…..…………2 → Q254	NUMBER OF TIMES……..[__|__]	MONTH…………….[__|__]<br><br>YEAR………..[__|__|__|__]	MONTHS…….[__|__]

Q254	CHECK Q208 AND Q246:<br><br>ONE OR MORE<br>BIRTHS, ABORTIONS, MISCARRIAGES, OR STILLBIRTHS<br>(Q208=1 OR MORE **OR** Q246=1)<br>☐↓<br><br>HAS HAD<br>NO BIRTHS ☐→<br>(Q208=0),<br>NO ABORTIONS,<br>NO MISCARRIAGE, **AND**<br>NO STILLBIRTHS (Q246=2)		**Q301**
Q255	How old were you when you got pregnant for the very **first time?**<br><br>**Shekarar ki nawa lokacin da ki ka dauki cikin farko?**	[___]___]<br>RECORD EXACT AGE	
Q256	At the time you became pregnant the first time, did you want to become pregnant **then**, did you want to wait until **later,** or did you **not want** to have any children at all?<br><br>***A lokacin da ki ka sami ciki, kin so dauka ne, ko kin so ki jinkirta sai zuwa gaba, ko kuma ba kida sha'awar sake haihuwar ko kadan?***	THEN……………………………….1 →<br>LATER………………………… ….. 2<br>NOT AT ALL………………………..3 →	**Q301**<br><br>**Q301**
Q257	How much longer would you have liked to wait?<br><br>***Har tsawon wane lokaci kika so ki jira?***<br><br>IF RESPONSE IS A DECIMAL FOR MONTHS & YEARS (2.5YRS), THEN CONVERT IN MONTH AND FILL IN ONLY MONTHS.	MONTHS……..1 [ ][ ]<br>OR<br>YEARS………..2 [ ][ ]<br>OR<br>OTHER (SPECIFY)_______________996<br>OR<br>DON'T KNOW…998	

SECTION 3: CONTRACEPTION

Qno	Questions and filters	Coding categories			Skip to
CHECK FOR PRESENCE OF OTHERS. BEFORE CONTINUING, MAKE EVERY EFFORT TO ENSURE PRIVACY.					
Now I would like to talk about family planning/child spacing/birth spacing, the various ways or methods that a couple/ partners can use to delay or avoid a pregnancy.<br><br>***Yanzu ina so in yi magana akan dabarun tsarin iyali, tazara tsakanin haihuwa/ tazara tsakanin yara daban daban da ma'aurata kebi don jinkirta daukar ciki ko guje wa daukan ciki.***					
INSTRUCTIONS: CIRCLE '1' FOR EACH METHOD MENTIONED SPONTANEOUSLY. THEN PROCEED DOWN COLUMN READING THE NAME AND DESCRIPTION OF EACH METHOD NOT MENTIONED SPONTANEOUSLY. CIRCLE '2' FOR EACH METHOD RECOGNIZED UPON LISTENING TO DESCRIPTION. CIRCLE '3' FOR EACH METHOD NOT MENTIONED NOR RECOGNIZED.					
Q301. Which ways or methods have you heard of? **Wadanne dabarun tsarin iyali daban-daban ki ka ta ba ji?**<br>PROBE: HAVE YOU HEARD OF ANY OTHER WAYS OR METHODS THAT WOMEN OR MEN CAN USE TO AVOID PREGNANCY?<br><br>FOR METHODS NOT MENTIONED SPONTANEOUSLY, ASK: Have you ever heard of (METHOD)?<br>**Kin taba jin (METHOD)?**					Q302. Have you ever used (METHOD)?<br><br>Kin taba amfani da? (METHOD)?
		Yes, MENTIONED SPONTANEOUSLY	Yes, WHEN PROBED	No	
01	FEMALE STERILIZATION/ TUBAL LIGATION: Women can have an operation to avoid having any more children.<br><br>***Ana iya yi wa mata aiki a mahaifa don kar su sake samun haihuwa.***	1	2	3→ **Next Method**	Have you ever had an operation to avoid having any more children?<br><br>An taba yi maki aiki a mahaifa don karki sake sake samun haihuwa?<br><br>YES………………. 1<br>NO………………… 2
02	MALE STERILIZATION/VASECTOMY: Men can have an operation to avoid having any more children.<br><br>***Ana iya yi wa maza dandaka don hana haihuwa***	1	2	3→ **Next Method**	Has your partner ever had an operation to avoid having any more children?<br><br>An taba yiwa Mijin ki/Abokin zaman ki aiki don kar ya sake samun haihuwa?<br>1<br>YES………………. 2<br>NO…………………
03	DAILY PILL: Women can take a pill every day to avoid becoming pregnant.<br><br>***Mata na iya shan kwayar magani a ko wacce rana don hana daukan ciki***	1	2	3→ **Next Method**	YES………………. 1<br>NO………………… 2
04	IUD: Women can have a loop or coil placed inside them by a health provider.<br><br>***Likitoci ko jami'an kula da kiwon lafiya kan sawa mata wata irin roba/ karfe a farjinsu don hana daukan cik***	1	2	3→ Next Method	YES………………. 1<br>NO………………… 2
05	INJECTABLES OR INJECTIONS: Women can have an injection by a health provider that stops them from becoming pregnant for one or more months.<br><br>*Jami'in kiwon lafiya na yi wa mata allura wadda za ta hana su samun ciki na wata daya ko fiye da haka.*	1	2	3→**Next Method**	YES………………. 1<br>NO………………… 2

06	IMPLANTS/IMPLANON/JADELLE: Women can have a small rod placed in their upper arm by a health provider, which can prevent pregnancy for one to three years.<br><br>***Likita ko jami'in kiwon lafiya kan sawa mata wasu kananan karafa a dantsen su don su hana daukan ciki na tsawon shekara daya zuwa shekera uku?***	1	2	3→**Next Method**	YES……………… 1<br>NO………………… 2

Q301. Which ways or methods have you heard of? **Wadanne dabarun tsarin iyali daban-daban ki ka ta ba ji?**<br><br>PROBE: HAVE YOU HEARD OF ANY OTHER WAYS OR METHODS THAT WOMEN OR MEN CAN USE TO AVOID PREGNANCY?<br><br>FOR METHODS NOT MENTIONED SPONTANEOUSLY, ASK: Have you ever heard of (METHOD)? **Kin taba jin (METHOD)?**					Q302. Have you ever used (METHOD)?<br><br>**Q.302 Kin taba amfani da ? (METHOD)?**
		Yes, MENTIONED SPONTANEOUSLY	Yes, WHEN PROBED	No	
07	MALE CONDOM: Men can put a rubber sheath on their penis before sexual intercourse.<br>**Maza na iya sa kororon roba a azzakarin su kafin suyi jima'i**	1	2	3→**Next Method**	YES……………… 1<br>NO………………… 2
08	FEMALE CONDOM: Women can place a sheath in their vagina before sexual intercourse.<br>**Mata na iya sanya irin na su kororon roba a farjin su kafin su yi jima'i**	1	2	3→**Next Method**	YES……………… 1<br>NO………………… 2
09	STANDARD DAYS/CYCLE BEADS: Every month that a woman is sexually active she can avoid pregnancy by not having sexual intercourse on the days of the month she is most likely to get pregnant. She keeps track of this using a color-coded string of beads that indicates fertile and non-fertile days of a menstrual cycle.<br><br>**Mace na iya amfani da zaren da aka daura ma duwatsun ado masu kala domin ta san ranar da za ta iya daukar ciki. A duk ranar da zata iya daukar ciki kuma zata iya amfani da kwaroron roba ko kuma ta fasa yin jima'i.**	1	2	3→**Next Method**	YES……………… 1<br>NO………………… 2
10	RHYTHM METHOD: Every month that a woman is sexually active she can avoid pregnancy by not having sexual intercourse on the days of the month she is most likely to get pregnant.<br>***Mace na iya gujewa yin jima'I daidai lokacin da take ganin tana iya daukan ciki.***	1	2	3→**Next Method**	YES……………… 1<br>NO………………… 2
11	WITHDRAWAL Men can be careful and pull out before climax.<br>***Namiji na iya zare azzakarin sa a yayin jima'I daidai lokacin da ya ji zai yi zuwan kai.***	1	2	3→**Next Method**	YES……………… 1<br>NO………………… 2
12	EMERGENCY CONTRACEPTION/MORNING AFTER PILL/ POSTINOR 2 Women can take pills up to 3 days after sexual intercourse to avoid becoming pregnant.<br>***Don gujewa daukan ciki mace na iya shan kwayoyin maganin hana daukan ciki har na tsawon kwanaki uku bayan jima'i***	1	2	3→**Next Method**	YES……………… 1<br>NO………………… 2
13	LACTATIONAL AMENORRHEA (LAM) Up to six (6) months after childbirth, a woman can use a method that requires that she feeds the baby with only breastmilk (no other formula, water or other food) and that her menstrual period has not returned.<br>***Mata kan yi amfani da hanyar shayar da nono (banda wani abinci /madara/ruwa) akai-akai dare da rana har na tsawon wata shida, wanda haka yakansa rashin ganin jinin hailar su***	1	2	3→**Next Method**	YES……………… 1<br>NO………………… 2

14	SPERMICIDE Women can place a suppository, jelly, or cream in their vagina before intercourse.<br>**Mace zata iya shafa wani irin mai ko basilin (mai wanda yak e da sinadarin) a al'aurar ta kafin ta yi jima'i.**	1	2	3→**Next Method**	YES………………. 1<br>NO………………… 2
15	DIAPHRAGM Women can place a thin flexible disk in their vagina before intercourse.<br>**Mace na iya sa wani irin faifai mara karfi a cikin farjin ta kafin ta yi jima'i.**	1	2	3→**Next Method**	YES………………. 1<br>NO………………… 2
16	Have you heard of any other ways or methods that women or men can use to avoid pregnancy?<br>***Kin ta ba jin wata dabara ko ta gargajiya da mata da maza ke amfani dasu don gujewa daukan ciki?***	YES______________ 1<br>(SPECIFY)<br>NO…………………… 3 →**Q303**			YES………………. 1<br>NO………………… 2
17	NOTE: IF RESPONDENT MENTIONS ABSTINENCE AS A METHOD OF FP, DO **NOT** RECORD AS A METHOD. PROBE FOR ANY OTHER METHOD KNOWN.	YES______________ 1<br>(SPECIFY)<br>NO………………… 3 →**Q303**			YES………………. 1<br>NO………………… 2

Q303	CHECK 301 (KNOWLEDGE OF ANY CONTRACEPTIVE METHOD).<br>IF 301=YES FOR ANY METHOD ☐ ↓<br>IF 301=NO FOR ALL METHOD ☐ →			Q316
Q304	In the past year, from whom or where have you seen or heard about birth spacing or family planning?<br><br>***A shekara guda da ta shige, daga wurin wanne ko ina ki ka ga ko ki ka ji maganar tazarar haihuwa ko kaiyade iyali?***<br><br>CIRCLE ALL RESPONSES MENTIONED.<br><br>PROBE: ANY OTHER?<br><br>**A ina kuma?**	**MEDIA SOURCES**<br>RADIO……………………………………<br>TV ………………………………………..<br>NEWSPAPERS……………………………<br>MAGAZINES……………………………….<br>BILLBOARDS……………………………..<br>WALL PAINTING…………………………<br>MOBILE PHONE/SMS………………….<br>INTERNET………………………………..<br>CINEMA…………………………………..<br>LIVE DRAMA/PUPPET SHOW…………...<br>POSTER…………………………………<br>LEAFLET/BROCHURE……………………<br><br>**PUBLIC SECTOR**<br>GOVERNMENT HOSPITAL ………… . . . .<br>WOMEN AND CHILDREN HOSPITAL<br>/CHILD WELFARE CLINIC……………<br>GOVT. HEALTH CENTER . . . .……..<br>GOVERNMENT POST/DISPENSARY……<br>OTHER PUBLIC___________________<br>(SPECIFY)<br><br>**PRIVATE/FBO/NGO SECTOR**<br>FAITH-BASED, CHURCH, MISSION<br>HOSPITAL/CLINIC....................................<br>PRIVATE HOSPITAL/CLINIC...................<br>NURSING/MATERNITY HOME……………<br>TRADITIONAL BIRTH ATTENDANT…….<br>COMMUNITY MIDWIFE……………………<br>COMMUNITY HEALTH WORKER ………..<br>TRADITIONAL HEALER……………………<br>PHARMACY…………………………………..<br>PMS/CHEMIST………………………………<br><br>**OTHER SOURCE**<br>WORKSITE CLINIC…………………<br>MOBILE CLINIC . . . .<br>YOUTH CENTER……………………<br>VCT……..……………………………....<br>BAR……………………………………..<br>KIOSK/SHOP/MARKET……………<br><br>**INTERPERSONAL SOURCES**<br>TEACHER……………………………………<br>PARENTS……………………………………<br>PARENTS-IN-LAW…………………………..<br>SPOUSE/PARTNER………………………...<br>SISTER/BROTHER…………………………<br>SISTER-IN-LAW/BROTHER-IN- LAW……<br>FRIENDS//NEIGHBOURS…………………<br>GRANDPARENTS…………………………<br>PEER EDUCATOR…………………………<br>WOMEN'S GROUP………………………..<br><br>OTHER (SPECIFY)_________________<br><br>HAS NOT SEEN OR HEARD IN THE LAST<br>YEAR……………………………………………<br>DON'T KNOW WHERE HEARD……………...	 AA<br>AB<br>AC<br>AD<br>AE<br>AF<br>AG<br>AH<br>AI<br>AJ<br>AK<br>AL<br><br><br>BA<br><br>BB<br>BC<br>BD<br>BE<br><br><br><br><br>CA<br>CB<br>CC<br>CD<br>CE<br>CF<br>CG<br>CH<br>CI<br><br><br>DA<br>DB<br>DC<br>DD<br>DE<br>DF<br><br><br>EA<br>EB<br>EC<br>ED<br>EE<br>EF<br>EG<br>EH<br>EI<br>EJ<br><br>XX<br><br><br>YY<br>ZZ	

Q305	Have you ever recommended any family planning methods to your friends and/or relatives?<br><br>**Kin ta ba baiwa kawayenki ko 'yanuwanki shawarar kaiyade iyali?**	YES………………………..………………….. 1<br>NO……………………………………..………. 2 →	**Q307**
Q306	Which methods have you recommended?<br><br>**Wanne irin dabarar ki ka bada shawara akai**?<br><br>CIRCLE ALL MENTIONED.<br><br>IF RESPONDENT SAYS "PILL", PROBE FURTHER TO ESTABLISH IF THEY MEAN THE "DAILY PILL" OR THE "EMERGENCY PILL"	FEMALE STERILIZATION…………………….. A<br>MALE STERILIZATION……………….............. B<br>IMPLANT…………………………………............ C<br>IUD……………………………………………… D<br>INJECTABLE... . . . . ….…....………… E<br>DAILY PILL……..……………………………… F<br>EMERGENCY PILL (Postnor2, etc.)………… G<br>MALE CONDOM . . . .………….............. H<br>FEMALE CONIDOM . .. . ….. . . ………...... I<br>STANDARD DAYS METHOD/<br>CYCLE BEADS…..………….……………… J<br>BREASTFEEDING/LAM . . . . . …..…………... K<br>OTHER MODERN METHOD<br>______________________________ L<br>(SPECIFY)<br>RHYTHM METHOD …………………….. M<br>WITHDRAWAL ………..………………... N<br>OTHER TRADITIONAL METHOD<br>____________________________ X<br>(SPECIFY)	
Q307	CHECK 302:<br>AT LEAST ONE "YES" (EVER USED) ☐ ↓ NOT A SINGLE "YES" (NEVER USED) ☐ →		**Q312**
Q308	Now I would like to ask you about the **<u>first</u>** time that you did something or used a method to avoid getting pregnant.<br><br>How many living children did you have at that time, if any?<br><br>How many boys?<br><br>And how many girls?<br><br>**Yanzu ina son in tambayeki game da lokacin da ki ka fara yin wani abun ko kuma ki fara amfani da wata dabara domin hana daukar ciki**<br><br>**'Yaya nawa kike dasu masu rai a wancan lokacin idan da akwai?**<br><br>'**Ya'ya Maza nawa?**<br>**Kuma 'ya 'ya Mata nawa?**<br><br>IF NONE, RECORD '00'.	NUMBER OF CHILDREN . . . . . ☐☐<br>NONE……………………………….……..00 →<br><br>NUMBER OF BOYS ☐☐<br><br>NUMBER OF GIRLS ☐☐	Q309
Q309	CHECK Q233 CURRENTLY PREGNANT<br><br>NOT CURRENTLY PREGNANT (Q233=2 OR =8) ☐ ↓ CURRENTLY PREGNANT (Q233=1) ☐ → Q312		
Q310	Are you (or your partner) **<u>currently</u>** doing something or using any method to delay or avoid getting pregnant?<br><br>***Shin ke ko (abokin zaman ki) na yin wani abu ko amafani da wata dabara yanzu don jinkirta ko gujewa daukan ciki?***<br><br>IF RESPONDENT OR RESPONDENT'S PARTNER HAS EVER BEEN STERILIZED, CIRCLE "YES" (CODE=1)	YES……………………………..……..………1<br>NO……………………………………….………2 →<br>SAYS SHE CAN'T GET PREGNANT……..….3 →	**Q312**<br>**Q312**

Q311	Which method(s) are you (or your partner) currently using?<br><br>***Wacce irin dabara ki* (ko abokin zaman ki) ke *amfani da shi yanzu?***<br><br>MULTIPLE METHODS – CIRCLE ALL MENTIONED<br><br>IF RESPONDENT SAYS "PILL", PROBE FURTHER TO ESTABLISH IF THEY MEAN THE "DAILY PILL" OR THE "EMERGENCY PILL"	FEMALE STERILIZATION……………………..A<br>MALE STERILIZATION……………….............B<br>IMPLANT………………………………...............C<br>IUD………………………………………………D<br>INJECTABLE... . . . . ………..…………..E<br>DAILY PILL………………………………………F<br>EMERGENCY PILL (Postnor2, etc.)…………..G<br>MALE CONDOM . . . . ………….................H<br>FEMALE CONDOM . .. . ..… . . .……….........I<br>STANDARD DAYS METHOD/<br>CYCLE BEADS…..……………..…………….J<br>BREASTFEEDING/LAM . . . . . ….……………..K<br>OTHER MODERN METHOD<br>_________________________________ L<br>(SPECIFY)<br>RHYTHM METHOD …………………………….M<br>WITHDRAWAL ………..……………….............N<br>OTHER TRADITIONAL METHOD<br>_________________________________X<br>(SPECIFY)	
Q312	CHECK Q311:<br><br>CIRCLE METHOD CODE:<br><br>IF MORE THAN ONE METHOD CODE CIRCLED IN Q311, CIRCLE CODE FOR HIGHEST METHOD IN LIST	NO CODE CIRCLED…………………………………00<br>FEMALE STERILIZATION…………….…………..01<br>MALE STERILIZATION……………………………...02<br>IMPLANT……………………………………………...03<br>IUD……………………………………….…………04<br>INJECTABLE... . . . . ………………….………05<br>DAILY PILL ……..………………………………..…06<br>EMERGENCY PILL (Postnor2, etc.)………………07<br>MALE CONDOM . . . . …………………….......08<br>FEMALE CONDOM. .. . . …. . . ……………………..09<br>STANDARD DAYS / SAFE DAYS./ BEADS…........10<br>LAM / BREASTFEEDING.......................................11<br>OTHER MODERN METHOD………………………..12<br>RHYTHM METHOD …………………………………13<br>WITHDRAWAL ……………………….....................14<br>OTHER TRADITIONAL METHOD…………………15	→ Q316 (for 00)
Q313	Since what month and year have you been using [CURRENT METHOD IN Q312] without stopping?<br><br>***Tun wanne wata da shekara ki ke amfani da wannan hanyar ba tare da kin tsaya ba?***	MONTH………………………[__|__]<br><br>YEAR……………………[__|__][__|__]	
Q314	CHECK Q313, Q215, Q249(01)- Q249(03)<br><br>ANY BIRTH OR PREGNANCY TERMINATION AFTER MONTH AND YEAR OF START OF USE OF CONTRACEPTION IN 313?<br><br>**<u>FOR METHODS OTHER THAN MALESTERILIZATION:</u>** GO BACK TO 313, PROBE AND RECORD MONTH AND YEAR AT START OF CONTINUOUS USE OF CURRENT METHOD (MUST BE AFTER LAST BIRTH OR PREGNANCY TERMINATION)<br><br>**<u>FOR MALE STERILIZATION</u>**: GO BACK TO 313, PROBE AND RECORD MONTH AND YEAR OF STERILIZATION	YES [ ] ↓ NO [ ] ↓	

<table>
<tr><td>Q315

C</td><td>CHECK 313:

YEAR IS JANUARY 2009 OR LATER

ENTER CODE FOR METHOD USED IN MONTH OF INTERVIEW IN <u>COLUMN 1</u> OF THE CALENDAR AND IN EACH MONTH BACK TO THE DATE STARTED USING METHOD

THEN CONTINUE WITH Q316</td><td>YEAR IS 2008 OR EARLIER

ENTER CODE FOR METHOD USED IN MONTH OF INTERVIEW IN <u>COLUMN 1</u> OF THE CALENDAR AND EACH MONTH BACK TO JANUARY 2009

THEN SKIP TO →</td><td>Q317</td></tr>
<tr><td>Q316

C</td><td colspan="2">I would like to ask you some questions about the times you or your partner may have used a method to avoid getting pregnant since January 2009.

Yanzu ina so in tambayeki game da lokutan da ke ko abokin zamanki ku ka taba amfani da wata dabara domin hana daukar ciki tun daga Janaury 2009?

USE CALENDAR TO PROBE FOR EARLIER PERIODS OF USE AND NONUSE, STARTING WITH MOST RECENT USE, BACK TO JANUARY 2009.
USE NAMES OF CHILDREN, DATES OF BIRTH, AND PERIODS OF PREGNANCY AS REFERENCE POINTS.

IN COLUMN 1, ENTER METHOD USE CODE OR ‘10’ FOR NONUSE IN EACH BLANK MONTH

ILLUSTRATIVE QUESTIONS:
• When was the last time you used a method? Which method was that?
• Yaushe ne lokacin karshe da ki ka yi amfani da wani dabarar tsarin iyali? Wanne irin dabara ne?

• When did you start using that method? How long after the birth of (NAME)?
• Yaushe ki ka fara amfani da wannan dabarar? Har tsawon wane lokaci ne bayan haihuwar (NAME)
• How long did you use the method then?
• Har tsawon wane lokacin ne ki ka yi amfani da wannan dabarar?

IN COLUMN 2, ENTER CODES FOR DISCONTINUATION NEXT TO THE LAST MONTH OF USE.
NUMBER OF CODES IN COLUMN 2 MUST BE SAME AS NUMBER OF INTERRUPTIONS OF METHOD USE IN COLUMN 1.

ASK WHY SHE STOPPED USING THE METHOD. IF A PREGNANCY FOLLOWED, ASK WHETHER SHE BECAME PREGNANT UNINTENTIONALLY WHILE USING THE METHOD OR DELIBERATELY STOPPED TO GET PREGNANT.

ILLUSTRATIVE QUESTIONS:
• Why did you stop using the (METHOD)? Don me ki ka daina amfani da wannan (METHOD)

• Did you become pregnant while using (METHOD), or did you stop to get pregnant, or did you stop for some other reason?

• Shin Kin dauki ciki ne da ki ke amfani da (DABARA), ko kin daina ne domin ki dauki ciki, ko kin daina ne domin wasu dalilai?

IF DELIBERATELY STOPPED TO BECOME PREGNANT, ASK:
• How many months did it take you to get pregnant after you stopped using (METHOD)? AND ENTER ‘0’ IN EACH SUCH MONTH IN COLUMN 1.
Har tsawon Wata nawa ya dauka kafin ki ka samu ciki bayan kin daina amfani da (METHOD)</td><td></td></tr>
</table>

Q317	CHECK Q312:<br>CIRCLE METHOD CODE:	NO CODE CIRCLED………………………….. 00<br>FEMALE STERILIZATION……….…………….. 01<br>MALE STERILIZATION………………............. 02<br>IMPLANT……………………………............... 03<br>IUD……………………………….…………… 04<br>INJECTABLE... . . . . ……....…………. 05<br>DAILY PILL…………………….……………… 06<br>EMERGENCY PILL (Postnor2, etc.)………… 07<br>MALE CONDOM . . . .……….............. 08<br>FEMALE CONDOM . .. . .…. . . ..…………...... 09<br>STANDARD DAYS METHOD/<br>CYCLE BEADS…..……………………………. 10<br>BREASTFEEDING/LAM . . . . . ….………….. 11<br>OTHER MODERN METHOD………………….. 12<br>RHYTHM METHOD ……………………………. 13<br>WITHDRAWAL ………..…………….............. 14<br>OTHER TRADITIONAL METHOD…………….. 15	Q338
Q318	Who decided which type of family planning/ child birth spacing/child spacing method to use? Is it mainly your decision, mainly your partner's decision, or did you both decide together?<br><br>***Shin wa ya ke yanke shawara wajen wane irin dabarar tsarin iyali/tazara tsakanin haihuwa/tazara tsakanin yara za ku yi amfani da shi? Shin ra' ayin ki ne ke kadai, ko na mijin ki /abokin zaman ki, ko ra'ayin ku ne tare ?***	MAINLY YOU..……….……………….……..1<br>MAINLY PARTNER…………..…………….…..2<br>JOINTLY……………………….……………..3<br>OTHER___________________________6<br>(SPECIFY)	
Q319	Were you ever told by a health or family planning worker about side effects or problems you might have using this family planning method [CIRCLED IN Q317]?<br><br>***Shin ko jami'an kiwon lafiya ko na tsarin iyali sun gaya miki illoli ko matsalolin da zasu iya faruwa idan kin yi amfani da wannan hanyar tsarin iyali?***	YES . . . . ……..…. . . . . . . ……….... 1<br>NO . . . .…….……….. 2<br>DON'T KNOW . . . .………. ……….. 8	 Q321<br>Q321
Q320	Were you told by a health or family planning worker what to do if you experienced side effects or problems with this method [CIRCLED IN Q317]?<br><br>***Shin ko jami'in kiwon lafiya ko na tsarin iyali sun gaya miki abin da za ki yi idan illolin ko idan matsaloli sun faru da wannan dabarar?***	YES . . . .………….. . . .1<br>NO . . . .……….…. . 2<br>DON'T KNOW/CAN'T REMEMBER . . .. ….. .8	
Q321	Were you ever told by a health or family planning worker about **other** methods of family planning (beside the one you are currently using)?<br><br>***Shin ko jami'an kiwon lafiya ko ta tsarin iyali sun taba gaya miki wasu dabaraun tsarin iyali/tazara tsakanin haihuwa/tazara tsakanin yara, da za ki iya amfani da su?(banda wanda ki ke amfani dashi yanzu)***	YES . . . .…….…... . . . . . .1<br>NO . . . .…….….. . . . . .2<br>DON'T KNOW . . . .………..…. 8	
Q322	How many living children did you have when you began using [CURRENT METHOD CIRCLED IN Q317] if any?<br>**'Ya'yan ki nawa ke raye lokacin da kika fara amfani da [CURRENT METHOD CIRCLED IN Q317] idan akwai?**<br><br>IF NONE, RECORD 00.<br><br>How many sons? **Maza nawa?**<br><br>How many daughters? **Mata nawa**?	NUMBER OF CHILDREN…………..[__|__]<br>NONE……………………….……………00<br><br>SONS ………………………………. .[__|__]<br>DAUGHTERS ……………………… [__|__]	 Q323

Q323	CHECK Q317:<br><br>IF CIRCLED FEMALE STERILIZATION “01” MALE STERILIZATION “02”, IMPLANT “03”, IUD “04”, INJECTABLE “05”, DAILY PILL “06”, EMERGENCY PILL “07”, MALE CONDOM “08”, FEMALE CONDOM “09”, STANDARD DAYS/SAFE DAYS/CYCLE BEADS “10”, OR OTHER MODERN METHOD “12” ☐ ↓	IF CIRCLED BREASTFEEDING/LAM “11”, RHYTHM METHOD “13” WITHDRAWAL“14” OR OTHER TRADITIONAL METHOD“15” ☐ →	**Q333**
Q324	From where or whom did you obtain [CURRENT METHOD] last time?<br><br>**Daga wanne wuri n ko wajen wanene ki ka samu [CURRENT METHOD] [CURRENT METHOD] zuwan ki karshe?**<br><br>PROBE: What is the name of this place/person? And where is it located?<br><br>**PROBE: Menene sunan wannan wurin/ ma’aikacin? Kuma a ina wurin yake?**	NAME OF FACILITY /PERSON<br>________________________________<br>________________________________<br><br>*CODE BOXES: OFFICE ONLY* [__|__|__|__|__]<br><br>STREET NAME/ADDRESS____________<br>________________________________<br>LAND MARK DESCRIPTION______________________	
Q325	What type of place/person is this?<br><br>***Wanne irin waje ko mutun ne?***<br><br>SINGLE MENTION.	**PUBLIC SECTOR**<br>GOVT HOSPITAL……………………………11<br>WOMEN AND CHILDREN’S HOSPITAL………………………………….….12<br>CHILD WELFARE CLINIC……………...…..13<br>GOVT. HEALTH CENTER……………………14<br>GOVERNMENT POST/DISPENSARY……………..……......15<br>MATERNITY HOME…………………….…..16<br>MOBILE CLINIC…………….………….……17<br>OTHER PUBLIC______________________18<br>(SPECIFY)<br><br>**PRIVATESECTOR**<br>PRIVATE HOSPITAL/CLINIC........................21<br>PRIVATE DOCTOR’S OFFICE……………..…22<br>NURSING/MATERNITY HOME……….….…23<br>PHARMACY…………………………….....…24<br>PMS/CHEMIST…………………….……......25<br>MOBILE CLINIC…………….……………..26<br>CHW/TBA…………………………………….…27<br>TRADITIONAL HEALER……………………..28<br>OTHER PRIVATE_____________________29<br>(SPECIFY)<br><br>**FAITH-BASED SECTOR**<br>MISSION HOSPITAL……………....................31<br>FAITH-BASED, CHURCH CLINIC…………….32<br><br>**OTHER SOURCE**<br>OTHER NGO HOSPITAL/CLINIC………...….41<br>WORKSITE CLINIC……………………..…..…..42<br>YOUTH CENTER…………………………….….43<br>VENDING MACHINE/DISPENSER……………44<br>VCT…….……………….……….………..........45<br>BAR………………………………………….…..46<br>KIOSK/SHOP/MARKET………………………..47<br>OTHER ______________________________96<br>(SPECIFY)<br>DON'T KNOW . . . .…….….……… 98	
Q326	Have you or your partner faced any challenges in obtaining this [CURRENT METHOD]?<br><br>**Shin ke ko abokin zaman ki ya taba fuskantar wata matsala wajen samun CURRENT METHOD]?**	YES……………………………………………… 1<br>NO………………………………………………… 2 →<br>DON’T KNOW, PARTNER OBTAINS METHOD…………………………… 8 →	 **Q328**<br><br>**Q328**

Q327	What are some of the challenges you have faced in obtaining this [CURRENT METHOD]?<br><br>**Wadanne matsaloli ku ka samu wajen samun CURRENT METHOD]?**<br><br>MULTIPLE RESPONSE – CIRCLE ALL MENTIONED	FEAR OF PARTNER KNOWING; HE OPPOSES USE……………………………….. A<br>FEAR OF OTHER RELATIVES KNOWING, AS THEY OPPOSE USE…………………….. B<br>UNABLE TO LEAVE HOUSEWORK/ WORK………………………………………….. C<br>LACK OF CHILDCARE ……………….......... D<br>HIGH COST OF TRANSPORTATION TO FACILITY…..………………………………….. E<br>FACILITY OFTEN CLOSED………………… F<br>HIGH COST OF SERVICE …………………. G<br>LONG WAITING TIMES AT FACILITY …….. H<br>PERIODIC STOCK-OUTS AT FACILITY…… I<br>PROVIDERS OFTEN AWAY……………….. J<br>UNFRIENDLY STAFF AT FACILITY ………. K<br>OTHER ___________________ X<br>(SPECIFY)	

Q328	CHECK Q317:<br><br>IF CIRCLED DAILY PILL "06" ☐ ↓	IF CIRCLED MALE CONDOM "08" ☐ →	**Q331**
		IF CIRCLED FEMALE STERILIZATION "01", MALE STERILIZATION "02", IMPLANT "03", IUD "04", INJECTABLE "05", EMERGENCY PILL "07", FEMALE CONDOM "09", OTHER MODERN METHOD "12" ☐ →	**Q332**

Q329	FOR DAILY PILL USERS ONLY: Which brand of pills did you buy/get the last time?<br><br>**FOR DAILY PILL USERS ONLY**: ***Wanne irin kwayoyi ki ka saya ko ki ka samu a lokacin zuwan ki na karshe?***<br><br>VERIFY BY ASKING TO SEE THE PACKAGE OF PILLS	CONFIDENCE………………………………<br>LOFEMINAL…………………………………<br>NORIDAY……………………………………<br>MICROGYNON……………………………..<br>DUOFEM……………………………………<br>NEGYNON…………………………………..<br><br>OTHER ___________________<br>[SPECIFY]<br>DON'T KNOW………………………………	01<br>02<br>03<br>04<br>05<br>06<br><br>96<br><br>98
Q330	FOR DAILY PILL USERS ONLY: How many (pill cycles) did you buy/get the last time?<br><br>**FOR DAILY PILL USERS ONLY: Kwayoyi guda nawa ki ka saya ko ki ka samu a lokacin *zuwan ki na karshe*?**	NUMBER OF PILL CYCLES ☐☐<br><br>DON'T KNOW…………….98 →	**ALL skip to Q332**
Q331	FOR CONDOM USERS ONLY: How many condom **<u>pieces</u>** did you buy/get the last time?<br><br>**FOR CONDOM USERS ONLY: Kororon roba guda nawa ki ka saya ko ki ka samu a lokacin *zuwan ki na karshe*?**<br><br>Example RESPONDENT BOUGHT ONE 3-PACK, WRITE "3"	NUMBER OF CONDOM PIECES ☐☐☐<br><br>DON'T KNOW…………….998	
Q332	What price did you pay for this current method [CIRCLED IN Q317]?<br><br>**Nawa ki ka biya ma wannan dabarar da ki ke amfani dashi yanzu?[CIRCLED IN Q317]?**	AMOUNT (in NAIRA) ☐☐☐☐☐<br><br>FREE……………………………………..……00000<br>DON'T KNOW………………………………99998	
Q333	Before you started using [CURRENT METHOD] did you (or your partner) use a different method?<br><br>**Kafin ku ka fara amfani da [CURRENT METHOD] ke ko abokin zaman ki kun yi amfani da wata hanyar tsarin iyali?**	YES . . . . 1<br>NO . . . . 2 →	**Q339**

Q334	Which method were you using before the [CURRENT METHOD]?<br><br>***Wacce irin hanyar tsarin iyali ku ke amfani da ita kafin [CURRENT METHOD]?***<br><br>IF MULTIPLE METHODS MENTIONED, CIRCLE THE HIGHEST METHOD ON THE LIST AND PROCEED WITH QUESTIONS REGARDING THIS METHOD.<br><br>IF RESPONDENT SAYS "PILL", PROBE FURTHER TO ESTABLISH IF THEY MEAN THE "DAILY PILL" OR THE "EMERGENCY PILL"	FEMALE STERILIZATION……………………..01<br>MALE STERILIZATION………………............02<br>IMPLANT…………………………….............03<br>IUD………………………..………………04<br>INJECTABLE... . . . . ……...………….05<br>DAILY PILL……….……………………………06<br>EMERGENCY PILL (Postnor2, etc.)…………..07<br>MALE CONDOM . . . .………….............08<br>FEMALE CONDOM . .. . ..…. . . .………….......09<br>STANDARD DAYS METHOD/<br>CYCLE BEADS…..……………….……………..10<br>BREASTFEEDING/LAM . . . . . …..…………..11<br>OTHER MODERN METHOD<br>_________________________________ 12<br>(SPECIFY)<br>RHYTHM METHOD ………………………… 13<br>WITHDRAWAL ……....…………………......... 14<br>OTHER TRADITIONAL METHOD<br>_________________________________ 15<br>(SPECIFY)	
Q335	How many living children did you have when you began using [PREVIOUS METHOD], if any?<br><br>**Ya'yan ki nawa ke raye lokacin da kika fara amfani da [PREVIOUS METHOD], Idan akwai?**<br><br>IF NONE, RECORD 00.<br><br>How many sons? **Maza nawa?**<br><br>How many daughters? **Mata nawa?**	NUMBER OF CHILDREN………….[__|__]<br>NONE………………………………….00 →<br><br>SONS ……………………………….[__|__]<br><br>DAUGHTERS ………………………[__|__]	**Q336**
Q336	How long did you use the last method [CIRCLED IN Q334]?<br><br>**Har tsawon wanne lokaci ki ka yi amfani da dabara hanyar tsarin iyali na karshe CIRCLED IN Q334]?**<br><br>IF RESPONSE IS A DECIMAL FOR MONTHS & YEARS (2.5YRS), THEN CONVERT IN MONTH AND FILL IN ONLY MONTHS	1 [__|__] DAYS<br>OR<br>2 [__|__] WEEKS<br>OR<br>3 [__|__] MONTHS<br>OR<br>4 [__|__] YEARS<br>UNSURE/CAN'T REMEMBER…………998	
Q337	Why did you stop using the method [CIRCLED IN Q334]?<br><br>**Wanne dalili ya sa ki ka daina amfani da wannan dabaran [CIRCLED IN Q334]?**<br><br>MULTIPLE RESPONSE - CIRCLE ALL MENTIONED.	WANTED TO GET PREGNANT…………... A<br>METHOD FAILED/GOT PREGNANT……... B<br>LACK OF SEXUAL URGE…………….….. C<br>FEAR OF BECOMING INFERTILE………… D<br>CREATED MENSTRUAL PROBLEM……...E<br>CREATED HEALTH PROBLEM…………… F<br>INFREQUENT SEX/NO SEX………………. G<br>INCONVENIENT TO USE………………….. H<br>HARD TO GET………………… ……………. I<br>GAINED WEIGHT…………………………... J<br>LOST WEIGHT……………………………… K<br>COSTS TOO MUCH…………………………L<br>DID NOT LIKE METHOD…………………… M<br>LACK OF PRIVACY…………………………. N<br>SPOUSE/PARTNER DID NOT APPROVE...O<br>MENOPAUSE/HYSTERECTOMY…………. P<br>HEALTH PROVIDER ADVISED TO STOP...Q<br>METHOD NOT AVAILABLE………………... R<br>OTHER____________________________ X<br>(SPECIFY)	**All skip to Q339**

Q338	What are the main reasons why you are not **currently** using a method of family planning/ child birth spacing to delay or avoid pregnancy?<br><br>***Shin wanne dalili ne yasa ba kya amfani da wata dabarar tsarin iyali/taraza tsakanin haihuha/tazara tsakanin yara don jinkirta wa ko hana daukan ciki yanzu?***<br><br>*CIRCLE ALL MENTIONED*<br><br>PROBE: Any other? **Akwai wani?**	**FERTILITY RELATED REASONS**:<br>A. NO SEX<br>B. INFREQUENT SEX<br>C. NOT MARRIED YET/ NO PARTNER<br>D. AWAY FROM SPOUSE<br>E. ALREADY PREGNANT<br>F. BREASTFEEDING<br>G. RECENTLY HAD BABY<br>H. WANTS MORE CHILDREN/TRYING TO GET PREGNANT<br>I. MENOPAUSAL/HYSTERECTOMY →<br>J. CAN'T HAVE (MORE) CHILDREN →<br><br>**OPPOSITION TO USE:**<br>K. RESPONDENT OPPOSES<br>L. PARTNER OPPOSES<br>M. OTHERS OPPOSE<br>N. RELIGIOUS PROHIBITION<br><br>**LACK OF KNOWLEDGE**:<br>O. KNOWS NO METHOD<br>P. DOESN'T KNOW WHICH METHOD TO USE<br>Q. DON'T KNOW HOW TO USE METHOD<br>R. KNOWS NO SOURCE<br><br>**METHOD-RELATED REASONS**:<br>S. HEALTH CONCERNS<br>T. FEAR OF SIDE EFFECTS<br>U. LACK OF ACCESS/TOO FAR<br>V. COSTS TOO MUCH<br>W. INCONVENIENT TO USE<br>X. DON'T LIKE EXISTING METHODS<br>Y. BAD EXPERIENCE WITH EXISTING METHODS<br><br>**FATALISTIC**:<br>Z. UP TO GOD<br><br>**OTHER**:<br>WW. OTHER ____________________<br>XX. OTHER ____________________<br>YY. OTHER ____________________<br>ZZ. DON'T KNOW	 <br><br><br><br><br><br><br><br>**Q343**<br>**Q343**
Q339	CHECK Q317<br><br>NO CODE CIRCLED, IMPLANT, IUD, INJECTABLE, DAILY PILL, EMERGENCY PILL, MALE CONDOM, FEMALE CONDOM, SDM, LAM, OTHER MODERN METHOD, RHYTHM METHOD, WITHDRAWAL, OTHER TRADITIONAL METHOD CIRCLED (0, 03-15) ☐ ↓	FEMALE STERILIZATION OR MALE STERILIZATION CIRCLED (01,02) ☐ →	**Q343**
Q340	Do you or your partner intend to use/continue to use a method to delay or avoid pregnancy within the next twelve (12) months?<br><br>***Shin Ke ko mijin ki/abokin zaman ki, na da niyar amfani/cigaba da wata dabarar don jinkirta ko guje wa daukan ciki nan da watanni goma sha biyu?***	YES . . . .1<br>NO . . . .2 →<br>DON'T KNOW . . . .8 →	 **Q343**<br>**Q343**

Q341	What method would you prefer MOST to use, if you do use a method in the future?<br><br>***Wacce irin dabara za ki fi son yin amfani dashi, in har za ki yi amfani da wata dabara nan gaba?***<br><br>SINGLE MENTION	FEMALE STERILIZATION……………………....01<br>MALE STERILIZATION……………….................02<br>IMPLANT…………………………………...............03<br>IUD…………………………………………………04<br>INJECTABLE... . . . . ………...…………..05<br>DAILY PILL…………………………………………06<br>EMERGENCY PILL (Postnor2, etc.)…………....07<br>MALE CONDOM . . . .………….................08<br>FEMALE CONDOM . .. . ….. . . …………..........09<br>STANDARD DAYS METHOD/<br>CYCLE BEADS…..…………..……………….....10<br>BREASTFEEDING/LAM . . . . . ….……………11 →<br>OTHER MODERN METHOD<br>______________________________ 12<br>(SPECIFY)<br>RHYTHM METHOD ……………………………. 13 }<br>WITHDRAWAL ………...……………….......... .. 14 } →<br>OTHER TRADITIONAL METHOD<br>_______________________________ 15<br>(SPECIFY)	**Q343**<br><br>**Q343**
Q342	Do you know of a place where or person from whom you can obtain this method?<br><br>**Kin san wani wuri ko mutumin da za ki iya amsar wannan dabarar tsarin iyalin?**	YES……………………………………….…. 1<br>NO………………………………………….….. .2	
Q343	Do you approve of using family planning?<br><br>***Kin amince da amfani da dabarar tsarin iyali?***	YES……………………………………………….1<br>NO…………………………………………………2<br>DON'T KNOW……………………………………8	

Quality of Care – Services and Treatment					
Please tell me how you would agree or disagree with the following statements. If you say you disagree, I will ask you to tell me if you strongly disagree or just disagree. If you say you agree, I will ask you to tell me if you strongly agree or just agree.<br><br>***Ina son ki gaya mani ko zaki yarda, ko ba zaki yarda ba, da wadannan maganganu.***<br>***Idan ba ki yarda ba, zan tambayeki, baki yarda ba sosai, ko kuma baki yarda ba ne kawai.***<br>***Idan kin yarda, zan tambayeki, ko kin yarda kwarai ne ko kuma kin yarda kawai.***		**Strongly Agree**<br><br>**Kin yarda sosai**	**Agree**<br><br>**Kin yarda kawai**	**Disagree**<br><br>**Ba ki yarda ba kawai**	**Strongly Disagree**<br><br>**Ba ki yarda ba sosai**
Q344	Family planning providers around here treat clients very badly.<br><br>***Jami'ai masu bayar da dabarun tsarin iyali a kewayen nan suna musgunawa wadanda suke zuwa wajensu***	4	3	2	1
Q345	Women don't like the way they are treated in family planning clinics around here.<br>***Mata basu son yadda ake lura da su a asibitin tsarin iyali a kewayen nan***	4	3	2	1
Q346	Family planning sellers/providers make women like you feel bad when obtaining contraceptives.<br><br>***Jami'ai masu sayar/bayar da dabarun tsarin iyali suna musgunawa mata irin ki alokacin da suke karban dabarar tsarin iyali?***	4	3	2	1
Q347	Women in this community believe that FP providers in this community are knowledgeable<br>**Matan da ke wannan al'ummar sun yarda da cewa Jami'an tsarin iyali na da ilimi matuka akan aikin su**.	4	3	2	1

Beliefs					
Please tell me how you would agree or disagree with the following statements. If you say you disagree, I will ask you to tell me if you strongly disagree or just disagree. If you say you agree, I will ask you to tell me if you strongly agree or just agree.<br><br>***Ina so ki gaya mani ko zaki yarda, ko ba zaki yarda ba, da wadannan maganganu. Idan ba ki yarda ba, zan tambayeki, baki yarda ba sosai, ko kuma baki yarda ba ne kawai. Idan kin yarda, zan tambayeki, ko kin yarda sosai ne ko kuma kin yarda ne kawai.***		**Strongly Agree**<br><br>**Kin Yarda sosai**	**Agree**<br><br>**Kin Yarda kawai**	**Disagree**<br><br>**Ba ki yarda ba kawai**	**Strongly Disagree**<br><br>**Ba ki yarda ba sosai**
Q348	Use of a contraceptive injection can make a woman permanently infertile.<br>***Amfani da allura tsarin iyali na iya hana mace haihuwa har abada***	4	3	2	1
Q349	People who use family planning end up with health problems.<br>***Mutane masu amfani da dabarun tsarin iyali suna karewa da matsalolin rashin lafiya***	4	3	2	1
Q350	Contraceptives can harm your womb.<br><br>***Dabarun tsarin iyali na iya kawo ila ga mahaifar mace***	4	3	2	1
Q351	Contraceptives reduce women's sexual urge.<br>***Dabarun tsarin iyali na rage shaáwar jima'i wa mata***	4	3	2	1
Q352	Contraceptives can cause cancer.<br>***Dabarun tsarin iyali na iya sa ciwon sankara/daji***	4	3	2	1
Q353	Contraceptives can give you deformed babies.<br>***Dabarun tsarin iyali zai iya baki nakasasun yara***	4	3	2	1
Q354	Contraceptives are dangerous to your health.<br>***Dabarun tsarin iyali su na da illa ga lafiyar ki.***	4	3	2	1
Q355	Women who use family planning /child birth spacing may become promiscuous<br>**Matan da ke amfani da *dabarun* tsarin iyali/ tazara tsakanin haihuwa da haihuwa na iya zama haliga?**	4	3	2	1
Q356	A man should accompany his wife to the health facility for family planning<br>***Miji yayi ma matarsa rakiya zuwa cibiyar kiwon lafiya domin samun dabaran tsarin iyali***	4	3	2	1

Section 4: MATERNAL AND CHILD HEALTH			
**Qno**	**Questions and filters**	**Coding categories**	**Skip to**
Q401	CHECK Q225:<br>ONE OR MORE BIRTHS SINCE JANUARY 2012 [ ] ↓	NO BIRTHS SINCE JANUARY 2012 [ ] →	**Q413**
Q402	ENTER NAME AND LINE NUMBER OF YOUNGEST CHILD BORN SINCE JANUARY 2012 FROM Q211 AND Q212:<br>____________ NAME [__|__] LINE NUMBER<br>Now I would like to ask you about the delivery of [NAME OF YOUNGEST CHILD].<br>***Yanzu ina so na tambayeki game da haihuwar ki ta karshe [*****NAME OF YOUNGEST CHILD**].		
Q403	Who assisted with the delivery of [NAME]?<br>*(NAME OF CHILD)*<br>CIRCLE ALL MENTIONED.<br>**Wa ya taimaka maki wajen haihuwar [NAME]?**<br>***NAME OF CHILD)***	DOCTOR/ CLINICAL OFFICER ……………. A<br>NURSE/ MIDWIFE……………………………. B<br>TBA . . . . ………………………… C<br>COMMUNITY HEALTH WORKER …………. D<br>FRIEND/RELATIVE……………………………. E<br>OTHER______________________________ X<br>(SPECIFY)<br>NO ONE……………………………………….. Y	
Q404	Where did you give birth to [NAME]?<br>A ina ki ka haifi [NAME]?	**PUBLIC SECTOR**<br>GOVT HOSPITAL……………………….11<br>WOMEN AND CHILDREN'S HOSPITAL……………………………....12<br>CHILD WELFARE CLINIC…………….....13<br>GOVT. HEALTH CENTER……………..14<br>GOVERNMENT POST/DISPENSARY…………………...15<br>MATERNITY HOME…………………….16<br>MOBILE CLINIC……..………………....17<br>OTHER PUBLIC_________________18<br>(SPECIFY)<br>**PRIVATESECTOR**<br>PRIVATE HOSPITAL/CLINIC..............21<br>PRIVATE DOCTOR'S OFFICE……….22<br>NURSING/MATERNITY HOME………23<br>MOBILE CLINIC………………………..26<br>CHW/TBA………………………………..27<br>TRADITIONAL HEALER……………….28<br>OTHER PRIVATE________________29<br>(SPECIFY)<br>**FAITH-BASED SECTOR**<br>MISSION HOSPITAL……………..........31<br>FAITH-BASED, CHURCH CLINIC..……32<br>**OTHER SOURCE**<br>OTHER NGO HOSPITAL/CLINIC……….41<br>WORKSITE CLINIC……………………….42<br>YOUTH CENTER………………………….43<br>OTHER FACILITY ________________48<br>(SPECIFY)<br>AT HOME…………………………………..51<br>OTHER(specify) __________________96	 <br><br><br><br><br><br><br><br><br><br><br><br><br><br><br><br><br><br><br><br><br><br><br><br><br><br>→ **Q409**<br>→ **Q409**
Q405	What is the name of this place? And where is it located?<br>Menene sunan wannan wajen?<br>Kuma a ina yake?	NAME OF FACILITY_______________________<br>_______________________<br>*CODE BOXES: OFFICE ONLY* [__|__|__|__|__]<br>STREET NAME/ADDRESS_______________________<br>_______________________<br>LAND MARK DESCRIPTION_______________________<br>_______________________	

Q406	When you came to the facility for delivery, did anyone give you information or counsel you on family planning before you delivered?***Lokacin da ki ka zo wannan cibiyar kiwon lafiyar domin haihuwa, shin akwai wanda ya maki bayani ko ya ba ki shawara kan hanyoyin tsarin iyali kafin ki haihu?***	YES……………………………………………1<br>NO……………………………………………..2	
Q407	After you had delivered, did anyone talk to you about using a family planning method postpartum before you left the health facility?<br><br>***Bayan haihuwar ki, ko akwai wanda ya yi maki magana kan hanyar tsarin iyali kafin ki bar cibiyar kiwon lafiyar?***	YES……………………………………………1<br>NO……………………………………………..2	
Q408	After you had delivered, did anyone talk to you about exclusive breastfeeding for contraceptive purposes?<br><br>**Bayan haihuwar ki, ko akwai wanda ya yi ma ki magana kan shayar da nono tsantsa domin tsarin iyali**?	YES……………………………………………1<br>NO……………………………………………..2 }	**Q410**

Q409	Why didn't you deliver in a health facility?<br><br>CIRCLE ALL MENTIONED.<br>**Wanne dalilin ya sa ba ki haihu a cibiyar kiwon lafiya ba?**	COSTS TOO MUCH……………………..…..A<br>FACILITY NOT OPEN………………….…B<br>DIDN'T HAVE TIME/LABOUR PAINS CAME EARLY…….……………………….................C<br>DON'T TRUST THE STAFF………………..D<br>NOT NECESSARY………………………...E<br>TRADITION………..………………………..…F<br>TOO FAR…………………………………..…..G<br>NO TRANSPORT AVAILABLE……………..H<br>NO ONE AVAILABLE TO ACCOMPANY HER ……………………….……………………….I<br>POOR QUALITY SERVICES………………..J<br>DO NOT OFFER SERVICES REQUIRED…K<br>PROVIDERS OFTEN AWAY………………..L<br>DOES NOT ACCEPT INSURANCE………..M<br>NO FEMALE PROVIDER……………………N<br>PARTNER/FAMILY WON'T ALLOW……….O<br>HUSBAND/SPOUSE NOT AT HOME………P<br>DIDN'T HAVE MONEY……………………....Q<br>POOR PROVIDER ATTITUDE………………R<br>POOR FACILITY ENVIRONMENT…………..S<br>FEAR OF HIV TESTING ……………………..T<br>OTHER____________________________ X<br>(SPECIFY)	
Q410	Within 12 months of delivery of [NAME ABOVE], did you start using a family planning method?<br><br>**A cikin watanni goma sha biyu da ki ka haifi [NAME ABOVE], kin fara amfani da dabarar tsarin iyali?**	YES…………………………..……………1<br>NO…………………………………………..2 →	**Q413**
Q411	Which method(s)?<br><br>**Wanne irin dabara (ko dabaru)?**<br><br>PROBE: Any others?<br>**Akwai wata kuma?**	FEMALE STERILIZATION……………………..A<br>MALE STERILIZATION……………….............B<br>IMPLANT……………………………..............C<br>IUD………………….…………….……………D<br>INJECTABLE... . . . . ……………….…...E<br>DAILY PILL……………………………………F<br>EMERGENCY PILL (Postnor2, etc.)…………..G<br>MALE CONDOM . . . .………….............H<br>FEMALE CONDOM . .. . ….. . . ………….......I<br>STANDARD DAYS METHOD/<br>CYCLE BEADS…..…………..………………..J<br>BREASTFEEDING/LAM . . . . . …..……………..K<br>OTHER MODERN METHOD<br>____________________________________ L<br>(SPECIFY)<br>RHYTHM METHOD ……………………………M<br>WITHDRAWAL ………..…………….............N<br>OTHER TRADITIONAL METHOD<br>____________________________________X<br>(SPECIFY)	
Q412	When did you start using the method (how long after delivery of ________[NAME ABOVE]<br>**Yaushe ki ka fara amfani da wannan dabarar (Tun yaushe bayan haihuwar________[NAME ABOVE**<br><br>RECORD COMPLETED DAYS IF LESS THAN 1 WEEK; COMPLETED WEEKS IF MORE THAN 7 DAYS AND LESS THAN 1 MONTH; COMPLETED MONTHS IF 1 MONTH OR MORE	DAYS POST PARTUM……………..1. [__|__]<br><br>WEEKS POST PARTUM…………..2 [__|__]<br><br>MONTHS POST PARTUM…………3 [__|__]<br><br>IMMEDIATELY POST PARTUM/TIME OF DELIVERY…………….…………….. 993	

Q413	CHECK BIRTH HISTORY (Q216 & Q219) FOR ANY LIVE CHILDREN<br><br>YES, HAS ONE OR MORE LIVING CHILD ☐ ↓ NO, DOES NOT HAVE LIVING CHILDREN ☐ →		**Q421**
Now I am going to ask you questions about your experience seeking and receiving health services.<br><br>***Yanzu ina son in tambaye ki game da yadda ki ke neman da karbar shaánin/kiwon lafiya.***			
Q414	In the <u>last three months</u>, have you gone to a health facility for any child health services?<br><br>***Cikin watanni ukku da suka shige kunje asibiti don kiwon lafiyan yaro/yarinya?***	YES . . . .……………...….1<br><br>NO . . . .………....……….2 →	**Q421**
Q415	What types of services did you receive during this/these child health visit(s)?<br><br>CIRCLE ALL MENTIONED.<br>**Wadanne irin abubuwa/kula aka yi ma ki lokacin/lokutan da ki ka je cibiyar kiwon lafiya da yaron/yarinyar?**	IMMUNIZATION…………………………….…..... A<br>DISEASE PREVENTION……………………..…. B<br>DEWORMING……………………………………. C<br>TREATMENT FOR CHILD (FOR DIARRHEA, MALARIA, OR RESPIRATORY INFECTION).... D<br>GROWTH MONITORING OF CHILD………….. E<br>HEALTH CHECK-UP . . . . ….…. …..… F<br>OTHER________________________________ X<br>(SPECIFY)	
Q416	Where did you go for child services at your last visit?<br><br>PROBE: What is the name of this place? And where is it located?<br><br>***Ina ki ka je don nema lafiyan yaro/yarinya ki na karshe?***<br><br>**PROBE:** ***Menene sunan wurin? Kuma a ina wurin yake?***	NAME OF FACILITY____________________________________<br>____<br>*CODE BOXES: OFFICE ONLY* [__|__|__|__|__]<br>STREET NAME/ADDRESS______________________________<br>____<br><br>LAND MARK DESCRIPTION____________________________	

Q417	What type of facility is this?<br><br>***Wanne irin asibiti ne?***	PUBLIC SECTOR<br>GOVERNMENT HOSPITAL……..……...............11<br>WOMEN AND CHILDREN'S HOSPITAL……….12<br>CHILD WELFARE CLINIC……………………13<br>GOVT. HEALTH CENTER………………….…14<br>GOVERNMENT POST/DISPENSARY………….15<br>MATERNITY HOME……………………….….16<br>MOBILE CLINIC……………………………..…17<br>OTHER PUBLIC________________________18<br>(SPECIFY)<br><br>PRIVATE SECTOR<br>PRIVATE<br>HOSPITAL/CLINIC……………………….………21<br>PRIVATE DOCTOR'S OFFICE…………………22<br>NURSING/MATERNITY HOME………………...23<br>PHARMACY………………………………….….24<br>PMS/CHEMIST……………….…………….….25<br>MOBILE CLINIC………………………………….26<br>CHW/TBA……………………………………..….27<br>TRADITIONAL HEALER…………………….....28<br>OTHER PRIVATE______________________29<br>(SPECIFY)<br><br>FAITH-BASED SECTOR<br>MISSION HOSPITAL……………….……...............31<br>FAITH-BASED, CHURCH CLINIC……………...32<br><br>OTHER SOURCE<br>OTHER NGO<br>HOSPITAL/CLINIC………………..…………..41<br>WORKSITE CLINIC………….………………..….42<br>YOUTH CENTER……………………………….....43<br>OTHER ____________________________96<br>(SPECIFY)<br>DON'T KNOW . . . . ….……………... . .98	
Q418	Why did you choose this facility?<br><br>CIRCLE ALL MENTIONED.<br><br>PROBE: ANY OTHER REASON?<br>**Menene ya sa ki ka zabi wannan cibiyar kiwon lafiya?**	SERVICES ARE FREE/ AFFORDABLE……………….. A<br>FACILITY OPEN/CONVENIENT HOURS OF<br>OPERATION…..……………………..……………. ……. B<br>STAFF ARE POLITE AND RESPECTFUL……………. C<br>CONVENIENT TO MY HOME…………….………….. D<br>CONVENIENT TO MY WORK………………………….. E<br>CONVENIENT TO WHERE I SHOP…………………… F<br>CONVENIENT USING AVAILABLE<br>TRANSPORT……………………………….. ………… G<br>GOOD QUALITY SERVICES…………………………… H<br>OFFER SERVICES REQUIRED……………………….. I<br>PROVIDERS AVAILABLE……………………………….. J<br>ACCEPT INSURANCE………………………………….. K<br>OFFER CREDIT FACILITIES…………………………… L<br>FACILITY HAS A GOOD REPUTATION………………. M<br>IT PROVIDES MULTIPLE SERVICES…………………. N<br>REFERRED THERE…………………………………... O<br><br>OTHER___________________________________ X<br>(SPECIFY)	
Q419	Did you receive any information or counseling on family planning/child birth spacing/child spacing during this visit?<br><br>***Kin samu wani bayani ko shawara akan tsarin iyali/tazara tsakanin haihuwa /tazara tsakanin yara a wannan lokacin ziyarar?***	YES . . . .……….…………….1<br><br>NO . . . .………...………….. .2	
Q420	Did you receive a method or a referral for family planning/child birth spacing at that time?<br><br>***Kin samu dabara ko an tura ki wajen tsarin iyali/tazara tsakanin haihuwa/tazara tsakanin yara a wancan lokacin?***	YES, RECEIVED A METHOD. . . . . ……………..1<br>YES, RECEIVED A PRESCRIPTION……………..…..2<br>YES, RECEIVED A REFERRAL . . . . ……………. .3<br>NO, DID NOT RECEIVE ANY OF THESE…………..…4	

Q421	In the <u>last year</u>, have you gone to a health facility for maternal health services, such as prenatal and postpartum care?<br><br>***Cikin shekararda ta shige, kin je asibiti domin kiwon lafiya da ya shafi mata kamar awon ciki ko kuma kula bayan haihuwa/lokacin jego?***	YES . . . .……….1<br><br>NO . . . .……….2 →	**Q427**
Q422	Where did you go most recently for maternal health services?<br><br>PROBE: What is the name of this place? And where is it located?<br><br>***Wanne wuri ki ka je kwanan nan domin samun kiwon lafiyar kafin ki haifu, lokacin da ki ke da juna biyu ko bayan ki haihu/lokacin jego?***<br><br>**PROBE: Menene sunan wurin kuma a wanne wuri yake?**	NAME OF FACILITY<br>______________________________<br>____<br>___<br>*CODE BOXES: OFFICE ONLY* [__|__|__|__|__]<br><br>STREET<br>NAME/ADDRESS_________________________<br>____<br>___<br><br>LAND MARK<br>DESCRIPTION_______________________<br>____	
Q423	What type of health facility did you go to for maternal health services at your last visit?<br><br>***Wanne irin cibiyar kiwon lafiya kika je na karshe, domin awon ciki ko kula kafin/bayan haihuwa?***	**PUBLIC SECTOR**<br>GOVERNMENT HOSPITAL……..……....................... 11<br>WOMEN AND CHILDREN'S HOSPITAL…………….. 12<br>CHILD WELFARE CLINIC…………………..……….. 13<br>GOVT. HEALTH CENTER…………………..……….. 14<br>GOVERNMENT POST/DISPENSARY……………….. 15<br>MATERNITY HOME……………………………………. 16<br>MOBILE CLINIC…………………………………………. 17<br>OTHER PUBLIC___________________________ 18<br>(SPECIFY)<br><br>**PRIVATE SECTOR**<br>PRIVATE<br>HOSPITAL/CLINIC………………………...………… 21<br>PRIVATE DOCTOR'S OFFICE……………………….. 22<br>NURSING/MATERNITY HOME………….…………… 23<br>PHARMACY……………………………...…………... 24<br>PMS/CHEMIST………………..………..…………… 25<br>MOBILE CLINIC…………………………………………. 26<br>CHW/TBA…………………………………...………… 27<br>TRADITIONAL HEALER…………………….………… 28<br><br>OTHER PRIVATE__________________________ 29<br>(SPECIFY)<br><br>**FAITH-BASED SECTOR**<br>MISSION HOSPITAL………….……………............... 31<br>FAITH-BASED, CHURCH CLINIC……………………. 32<br><br>**OTHER SOURCE**<br>OTHER NGO<br>HOSPITAL/CLINIC…………………….……………… 41<br>WORKSITE CLINIC…………….……………............. 42<br>YOUTH CENTER…………………………….............. 43<br>VCT……………………………………………............ 45<br>OTHER __________________________________ 96<br>(SPECIFY)<br>DON'T KNOW . . . .………………. . ………. 98	

Q424	Why did you choose this facility?<br><br>***Menene dalilin da ya sa ki ka zabi wannan cibiyar kiwon lafiyar?***<br><br>*PROBE: ANY OTHER REASON?*<br><br>***Akwai wani dalilin kuma?***<br><br>MULTIPLE RESPONSES	SERVICES ARE FREE/ AFFORDABLE…..… ……………. A<br>FACILITY OPEN/CONVENIENT HOURS OF OPERATION…..………………….……….………………. B<br>STAFF ARE POLITE AND RESPECTFUL…. ……………. C<br>CONVENIENT TO MY HOME……………..… ……………. D<br>CONVENIENT TO MY WORK……………..… …………….. E<br>CONVENIENT TO WHERE I SHOP……………………….. F<br>CONVENIENT USING AVAILABLE TRANSPORT……….. G<br>GOOD QUALITY SERVICES…………….………………….. H<br>OFFER SERVICES REQUIRED…………..……………….. I<br>PROVIDERS AVAILABLE…………………………………. J<br>ACCEPT INSURANCE……………………………………. K<br>OFFER CREDIT FACILITIES…………………………….. L<br>FACILITY HAS A GOOD REPUTATION………………….. M<br>IT PROVIDES MULTIPLE SERVICES……….………….. N<br>REFERRED THERE……………………………..……….. O<br><br>OTHER______________________________________ X<br>(SPECIFY)	
Q425	At your last visit, did you receive any information or counseling on family planning/ child birth spacing?<br><br>**Lokacin ziyarar ki ta karshe, shin kin sami bayanai ko shawara kan hanyar tsarin iyali/ tazarar haihuwa?**	YES . . . . ….……..…….1<br><br>NO . . . . ….………….2	
Q426	Did you receive a method or a referral for family planning/ contraceptive method at that time?<br><br>**Shin kin karbi wata dabara ko an tura ki wajen dabaran tsarin iyali/tazara tsakanin haihuwa/tazara tsakanin yara a wancan lokacin**	YES, RECEIVED A METHOD. . . . . . . ….…….…. . . 1<br>YES, RECEIVED A PRESCRIPTION……………....2<br>YES, RECEIVED A REFERRAL . . . . ……..….3<br>NO, DID NOT RECEIVE ANY OF THESE…………4	
Q427	I don't want to know the results, but In the <u>last year</u>, have you gone to a health facility for an HIV test?<br><br>***Ba na son nasan sakamakon gwajin, a shekarar da ta wuce ko kinje cibiyar kiwon lafiya domin gwajin cutar kanjamau?***	YES . . . . …….………..…1<br><br>NO . . . . ….………..…..2 →	**Q432**
Q428	Where did you go for your last HIV test?<br><br>**Awace cibiyar kiwon lafiyar ki ka je zuwan kin a karshe domin gwajin cutar kanjamau?**<br><br>PROBE: What is the name of this place? And where is it located?<br><br>**PROBE: Menene sunan wurin kuma a ina yake?**	NAME OF FACILITY______________________<br><br>*CODE BOXES: OFFICE ONLY* [__|__|__|__|__]<br><br>STREET NAME/ADDRESS_________________<br><br>LAND MARK DESCRIPTION _______________	

Q429	What type of facility did you go to for your last HIV test?<br><br>**Wacce irin cibiyar kiwon lafiya ki ka je zuwan kin a karshe domin gwajin cutar kanjamau?**	**PUBLIC SECTOR**<br>GOVERNMENT HOSPITAL……..……....................... 11<br>WOMEN AND CHILDREN'S HOSPITAL…………….. 12<br>CHILD WELFARE CLINIC………………..……….. 13<br>GOVT. HEALTH CENTER………………..……….. 14<br>GOVERNMENT POST/DISPENSARY……………….. 15<br>MATERNITY HOME…………………………………… 16<br>MOBILE CLINIC………………………………………… 17<br>OTHER PUBLIC___________________________ 18<br>(SPECIFY)<br><br>**PRIVATE SECTOR**<br>PRIVATE<br>HOSPITAL/CLINIC………………………...………… 21<br>PRIVATE DOCTOR'S OFFICE……………………….. 22<br>NURSING/MATERNITY HOME…………….………… 23<br>PHARMACY……………………………...………….. 24<br>PMS/CHEMIST……………….……….….…….…… 25<br>MOBILE CLINIC………………………………………… 26<br>CHW/TBA…………………………………...………… 27<br>TRADITIONAL HEALER………………….………… 28<br><br>OTHER PRIVATE_________________________ 29<br>(SPECIFY)<br><br>**FAITH-BASED SECTOR**<br>MISSION HOSPITAL……………….……….............. 31<br>FAITH-BASED, CHURCH CLINIC……………………. 32<br><br>**OTHER SOURCE**<br>OTHER NGO<br>HOSPITAL/CLINIC……………………..……………… 41<br>WORKSITE CLINIC……………..…………............. 42<br>YOUTH CENTER……………………………............... 43<br>VCT…………………………………………….............. 45<br>OTHER ______________________________ 96<br>(SPECIFY)<br>DON'T KNOW . . . .…..…………. . ………. 98	
Q430	At the time of your last test, did you receive any information or counseling on family planning/ child birth spacing?<br><br>***Lokacin gwajin ki na karshe, shin ko kin sami bayani ko shawara kan hanyar tsarin iyali / tazarar haihuwa?***	YES . . . .…………..…………… 1<br><br>NO . . . .……..………………. 2	
Q431	Did you receive a method or a referral for family planning/contraceptive method at that time?<br><br>**Shin kin karbi wata dabara ko an tura ki wajen dabaran tsarin iyali/tazara tsakanin haihuwa/tazara tsakanin yara a wancan lokacin**	YES, RECEIVED A CONDOM………………………………... 1<br>YES, RECEIVED A METHOD OTHER THAN<br>A CONDOM………………... . . . . ……... ………. …… 2<br>YES, RECEIVED A PRESCRIPTION. . . . ……. . . …………… 3<br>YES, RECEIVED A REFERRAL . . . . . . ……... . ……………. 4<br>NO, DID NOT RECEIVE ANY OF THESE……..……………… 5	

Q432	In the last year, how often did you visit a pharmacy?<br><br>***A cikin shekarar da ta shige sau nawa ki ka ziyarci kantin magani?***	TIMES PER YEAR………………..1 [ ][ ]<br><br>TIMES PER MONTH………………2 [ ][ ]<br><br>TIMES PER WEEK..………………3…… [ ][ ]<br><br>Never visit a pharmacy………….……………….. 997 →	Q435
Q433	Have you ever received any information or counseling on family planning/birth spacing while purchasing or obtaining medicine from this pharmacy?<br><br>**Kin ta ba samun wani bayani ko shawara kan hanyar tsarin iyali/tazarar haihuwa lokacin da kike sayen maganin a wannan kantin magani?**	YES . . . .…………1<br>NO . . . .…………2	

Q434	In the past year, did you receive a method, information or counseling or a referral for family planning/ child birth spacing methods at the pharmamcy?<br><br>***A cikin shekarar da ta shige, kin ta ba samun wani dabara, bayani, ko shawara gameda da dabarar tsarin iyali ko an tura ki wajen da ake tsarin iyali / tazara tsakanin haihuwa a kantin maganin****?*	YES, RECEIVED A METHOD…………………..……..A<br>YES, RECEIVED INFORMATION OR COUNSELING…………………………..… . . . . .B<br>YES, RECEIVED A REFERRAL . . . …………... ... .. .C<br>NO, DID NOT RECEIVE ANY OF THESE………..….Y	
Q435	In the last year, how often did you visit a PMS/ chemist?<br><br>**A Shekarar da ta shige, ki na zuwa kantin magani/kemis akai akai, kamar sau nawa?**	TIMES PER YEAR………….……...1 [ ][ ]<br>TIMES PER MONTH……….…….2 [ ][ ]<br>TIMES PER WEEK..……….………3.…. [ ][ ]<br>Never visit a PMS/ chemist…………………….. 997 →	Q501
Q436	In the past year, did you receive a method, information or counseling or a referral for family planning/ child birth spacing methods at the PMS/chemist ?<br><br>**A cikin shekarar da ta shige, kin ta ba samun wani dabara, bayani, ko shawara gameda da dabarar tsarin iyali ko an tura ki wajen da ake tsarin iyali / tazara tsakanin haihuwa a kantin maganin/kemis**	YES, RECEIVED A METHOD…………………...……A<br>YES, RECEIVED INFORMATION OR COUNSELING……………. . . . . …............ . .B<br>YES, RECEIVED A REFERRAL . . . . . . …. . …..… .C<br>NO, DID NOT RECEIVE ANY OF THESE…..…......Y	

<table>
<tr><th colspan="4">SECTION 5: SEXUAL ACTIVITY AND MARRIAGE</th></tr>
<tr><th>Qno</th><th>Questions and filters</th><th>Coding categories</th><th>Skip to</th></tr>
<tr><td></td><td colspan="3">Now I would like to ask you some questions about your recent sexual activity. Let me assure you again that your answers are completely confidential and will not be told to anyone. If we should come to a question that you don't want to answer, just let me know and we will go to the next question

Yanzu ina son in yi mi ki tambayoyi akan jima'in da ki ka yi kwanannan.Ina son in sake tabbatar maki duk amsoshin da ki ka bada sirri ne, domin ba za 'a fadawa wani ba, idan muka zo wurin tambayar da ba kya son amsawa, ki fada mun don muje tambaya ta gaba.

MAKE SURE YOU ARE SITTING IN A PRIVATE PLACE TO DISCUSS THE FOLLOWING QUESTIONS.</td></tr>
<tr><td>Q501</td><td>Now I need to ask you some questions about sexual activity in order to gain a better understanding of some family life issues. How old were you when you had sexual intercourse for the very first time?

Yanzu ina son na yi maki wasu tambayoyi a kan jima'i domin samun kyaykyawar fahimta akan harkar iyali

Shekarun ki nawa cikakku lokacin da ki ka fara yin jima'i?</td><td>AGE………………………..…. [__|__]

NEVER HAD SEXUAL INTERCOURSE………………00 →</td><td>Q510</td></tr>
<tr><td>Q502</td><td>The first time you had sexual intercourse; did you or your partner use a family planning/birth spacing method to avoid getting pregnant?

Lokacin da ki ka yi jima'in farko, ke ko mijin ki/abokin zamanki yayi amfani da han yar tasrin iyali/tazarar haihuwa domin hana daukar ciki?</td><td>YES……………………………………………………………1
NO…………………………………………………………2 →
CAN'T REMEMBER……………………………………..8 →</td><td>
Q504
Q504</td></tr>
<tr><td>Q503</td><td>Which method(s) was used?

Wacce irin dabarar tsarin iyali ku ka yi amfani da ita?

CIRCLE ALL MENTIONED

IF RESPONDENT SAYS "PILL", PROBE FURTHER TO ESTABLISH IF THEY MEAN THE "DAILY PILL" OR THE "EMERGENCY PILL".</td><td>FEMALE STERILIZATION…………………….A
MALE STERILIZATION……………….............B
IMPLANT…………………………………...........C
IUD……………………………………………….D
INJECTABLE... . . . . ………...………….E
DAILY PILL……………………………………..F
EMERGENCY PILL (Postnor2, etc.)……….....G
MALE CONDOM . . . . ……………............H
FEMALE CONDOM . .. . ….. . . ……………........I
STANDARD DAYS METHOD/ SAFE DAYS/ CYCLE BEADS…..………………...................J
BREASTFEEDING/LAM . . . . . …..…………..K
OTHER MODERN METHOD
______________________________ L
(SPECIFY)
RHYTHM METHOD ……..………………….…..M
WITHDRAWAL ………..……………….............N
OTHER TRADITIONAL METHOD
______________________________X
(SPECIFY)</td><td></td></tr>
<tr><td>Q504</td><td>When was the <u>last</u> time you had sexual intercourse?

Yaushe ki ka yi jima'i na karshe?

WHEN LESS THAN A DAY, RECORD "00" DAYS.
IF LESS THAN ONE WEEK AGO, RECORD DAYS
IF LESS THAN ONE MONTH AGO, RECORD WEEKS AGO
IF LESS THAN 12 MONTHS AGO, RECORD MONTHS
IF 12 MONTHS OR MORE, RECORD IN YEARS</td><td>DAYS AGO……………1 [__|__]
OR
WEEKS AGO…………2 [__|__]
OR
MONTHS AGO……… 3 [__|__]
OR
YEARS AGO…………4 [__|__] →</td><td>Q510</td></tr>
</table>

Q505	How many times have you had sex in the last three (3) months?<br><br>**Sau nawa ki ka yi jima'l cikin watanni uku da suka shige?**	NUMBER OF TIMES…………… [__|__|__]<br><br>**OR**<br>NONE……………………………………………000<br>DAILY……………………………………………991<br>WEEKLY ……………………………………...992<br>MONTHLY……………………………………..993<br>OTHER___________________________ 996<br>(SPECIFY)<br>DON'T KNOW……………………………….. 998	
Q506	In total, how many men have you had sex with in the last 12 months?<br>**Jimillar maza nawa kika yi jima'l dasu cikin watanni 12 da suka shige?**<br><br>IF MORE THAN 95, WRITE 95.	NUMBER OF PARTNERS…..[__|__]<br><br>TOO MANY TO ESTIMATE……96	
Q507	In the last twelve (12) months, have you ever given or received money, gifts, or favours in return for sex?<br><br>**A cikin watanni 12 da suka shige, shin kin taba bayar da kudi ko karban kudi, kyauta ko wata alfarma sakamakon yin jima'i**?	YES . . . . ….... . . . . 1<br>NO . . . . ….... 2	
Q508	The last time you had sexual intercourse, did you or your partner use a family planning/birth spacing/child spacing method?<br><br>**Lokacin da ki ka yi jima' i na karshe, shin ke ko abokin zamanki ya yi amfani da wata hanyar tsarin iyali/ dabarar tazarar haihuwa?**	YES……………………………………….. 1<br>NO…………………………………………..2 →<br>CAN'T REMEMBER………………………8 →	 Q510<br>Q510
Q509	Which method was used?<br><br>**Wacce irin dabara tsarin iyali ku ka yi amfani da ita?**<br><br>CIRCLE ALL MENTIONED.<br><br>IF RESPONDENT SAYS "PILL", PROBE FURTHER TO ESTABLISH IF THEY MEAN THE "DAILY PILL" OR THE "EMERGENCY PILL"	FEMALE STERILIZATION……………………A<br>MALE STERILIZATION…………….............B<br>IMPLANT……………………………….............C<br>IUD……………….…………………………….D<br>INJECTABLE... . . . . …….....………..E<br>DAILY PILL……………………………………..F<br>EMERGENCY PILL (Postnor2, etc.)……….....G<br>MALE CONDOM . . . . ……….................H<br>FEMALE CONDOM . .. . ….. . . ……….........I<br><br>STANDARD DAYS METHOD/ SAFE DAYS/ CYCLE BEADS…..………………...............................J<br>BREASTFEEDING/LAM . . . . . …..…………..K<br>OTHER MODERN METHOD<br>_________________________________ L<br>(SPECIFY)<br>RHYTHM METHOD ……………………….…..M<br>WITHDRAWAL ……….………………............N<br>OTHER TRADITIONAL METHOD<br>___________________________________X<br>(SPECIFY)	

Now I would like to ask you some questions about marriage. Remember that your responses will be kept confidential.

Yanzu ina so nayi maki wasu tambayoyi akan auren ki.Duk amsoshin ki za su kasance a sirrance

Q510	What is your marital status now: are you currently married or living with man as if married, widowed, divorced, or separated?<br><br>***Menene matsayin auren ki yanzu: Kina da aure ko ki na zaune da namiji kamar zaman aure, ko mijin ki ya rasu, ko auren ki ya mutu ko kuma kin yi yaji ne ko kuma baki taba aure ba?***	CURRENTLY MARRIED…………………….…..1<br>LIVING WITH A MAN……………….................2<br>REMARRIED…………………….……………...…3<br><br>WIDOWED……………….……….…………….4<br>DIVORCED………………………….………....5<br>SEPARATED…………………………….….…6<br>NEVER MARRIED…………………………..…7	(4–7) **Q512**
Q511	Is your husband/partner living with you now, or is he staying elsewhere?<br><br>***Shin yanzu kina tare da mijin ki/abokin zaman, ko shi yana wani waje dabam ne?***	LIVING WITH YOU……..……………….... 1<br>STAYING ELSEWHERE …………………2	
Q512<br><br>**C**	CHECK Q510:<br><br>DETERMINE MONTHS MARRIED, REMARRIED OR LIVING WITH A MAN SINCE JANUARY 2009. ENTER 'X' IN COLUMN 3 OF CALENDAR FOR EACH MONTH MARRIED, REMARRIED OR LIVING WITH A MAN. ENTER 'O' IN COLUMN 3 OF CALENDAR FOR EACH MONTH SHE WAS NOT IN UNION.<br><br>FOR WOMEN WHO ARE WIDOWED/DIVORCED/SEPARATED/NEVER MARRIED(Q510=4 OR Q510=5 OR Q510=6 OR Q510=7):<br>PROBE FOR DATE WHEN LAST MARRIAGE STARTED AND TERMINATION DATE. ENTER 'X' IN COLUMN 3 OF CALENDAR FOR EACH MONTH MARRIED ENTER 'O' IN COLUMN 3 OF CALENDAR FOR EACH MONTH WIDOWED/DIVORCED/SEPARATED		
Q513	CHECK MARITAL STATUS Q510<br><br>CURRENTLY MARRIED:<br>IF CIRCLED RESPONSES '1' FOR CURRENTLY MARRIED OR '2' FOR LIVING WITH A MAN OR '3' FOR REMARRIED<br>[ ] ↓	NOT CURRENTLY MARRIED:<br>IF CIRCLED RESPONSES '4' FOR WIDOWED OR '5' FOR DIVORCED OR '6' FOR SEPERATED [ ] →<br><br>'7' FOR NEVER MARRIED. [ ] →	Q518<br><br>**Q601**
Q514	Besides yourself, does your husband/partner have other wives?<br>***Ban da ke, shin mijinki/abokin zamanki yana da wasu matan ko yana zaune da wasu matan kamar zaman aure?***	YES . . . .………………. . . …..1<br>NO . . . .…………... . . …......2<br>DON'T KNOW . . . .…………... ……….8	 **Q517**<br>**Q517**
Q515	Including yourself, in total, how many wives does your husband/partner have?<br>***Har da ke gabaki daya mata nawa mijin ki/abokin zaman ki yake dasu?***	TOTAL NUMBER OF WIVES………….. [__|__]<br>DON'T KNOW . . . .………..… . . 98	
Q516	Are you the first, second, third, fourth…… wife?<br><br>***Shin ke ce matar fari, ta biyu, ta uku, ta hudu…., a cikin matan mijin ki /abokan zaman ki?***	RANK…………… [ ][ ]	
Q517	Do you think your spouse/partner has any other sexual partners who are not wives? If so, how many do you think?<br><br>***A tunanin ki, mijinki/abokin zamanki yana da wadanda yake yin jima'lidasu wadanda ba matansa bane? Idan haka ne, su nawa?***	NUMBER OF PARTNERS…………….. [ ][ ]<br><br>IF NONE, FILL IN '00'<br><br>DON'T KNOW…………………………….98	

Q518	Have you been married or lived with a man as if married only once or more than once?<br><br>**Kin ta ba yin aure, ko zama da namiji kamar kun yi aure a kalla sau daya ko da dama**?	ONLY ONCE……………….……….. 1<br>MORE THAN ONCE……………….. 2	
Q519	CHECK 518<br><br>**MARRIED/LIVED WITH MAN ONLY ONCE** ☐ ↓<br>In what month and year did you start living with your husband/ partner?<br>**A wane wata kuma cikin wacce shekara kika fara zama da mijinki/abokin zamanki?**<br><br>**MARRIED/LIVED WITH MORE THAN ONE MAN** ☐ ↓<br>Now I would like to ask about when you started living with your first husband/partner. In what month and year was that?<br>**Yanzu ina son in tambeyiki game da lokacin da ki ka fara zama da mijin ki/abokin Zaman ki.A wane wata kuma cikin wane shekara ne?**	MONTH…………………….…………….. ☐☐<br><br>DON'T KNOW MONTH………………………..98<br><br>YEAR……………….…………………….…… ☐☐☐☐<br><br>DON'T KNOW YEAR………………………….9998	
Q520	How old were you when you **<u>first</u>** started living with him?<br><br>**Shekarar ki nawa lokacin farko da kika fara zama da shi?**	AGE…...…………… ☐☐<br><br>DON'T REMEMBER…….98	

<table>
<tr><th></th><th colspan="3">SECTION 6: FERTILITY PREFERENCES</th></tr>
<tr><th></th><th>Questions and filters</th><th>Coding categories</th><th>Skip to</th></tr>
<tr><td>Q601</td><td colspan="2">CHECK Q312: METHOD CURRENTLY USING

DOES NOT USE FEMALE STERILIZATION ☐ ↓   FEMALE STERILIZATION (Q312=1) ☐ →</td><td>Q604</td></tr>
<tr><td colspan="4">Now, I would like to ask you a few questions about you and your opinion about births.

Yanzu, ina son in yi maki wadansu tamboyoyi akan ki da ra'ayin ki game da haife-haife</td></tr>
<tr><td>Q602</td><td>CHECK Q233:
NOT PREGNANT OR UNSURE ☐ ↓
Now I have some questions about the future. Would you like to have (a/another) child, or would you prefer not to have any (more) children?
Yanzu ina da wasu tambayoyi game da nan gaba.Kina fatan samun wani da/ya ko kuma ba kya son samun karin 'ya'ya?

PREGNANT ☐ ↓
Now I have some questions about the future. After the birth of the child you are expecting now, would you like to have another child, or would you prefer not to have any more children?
Bayan wannan haihuwar da ki ke sa rai yanzu, har tsawon wane lokaci ki ke so ki dakata kafin ki sake wata haihuwar?</td><td>HAVE (A/ANOTHER) CHILD…….1
NO MORE/NONE………………...2
SAYS SHE CAN'T GET PREGNANT……………………......3
UNDECIDED/DON'T KNOW…….8</td><td>
Q604
Q604
Q604</td></tr>
<tr><td>Q603</td><td>CHECK Q602:
NOT PREGNANT OR UNSURE ☐ ↓
How long would you like to wait from now before having (a/another) child?
Har tsawon wane lokaci ki ke so ki dakata kafin ki sake haifar wani 'dan?
IF RESPONSE IS IN MONTHS AND YEARS (E.G. 2 ½ YEARS), CONVERT TO MONTHS (e.g. 30 months)

PREGNANT ☐ ↓
After the birth of the child you are expecting now, how long would you like to wait before having another child?
Bayan wannan haihuwar da ki ke sa rai yanzu,har tsawon wane lokaci ki ke so ki dakata kafin ki sake wata haihuwar?
IF RESPONSE IS IN MONTHS AND YEARS (E.G. 2 ½ YEARS), CONVERT TO MONTHS (e.g. 30 months)</td><td>MONTHS . . . . 1 [__][__]
YEARS . . . . 2 [__][__]
SOON/NOW . . . .…… .. . .. .993
AFTER MARRIAGE…………………..995
OTHER ____________________ 996
(SPECIFY)
DON'T KNOW . . . . 998</td><td></td></tr>
</table>

Q604	CHECK Q510: CURRENTLY MARRIED<br><br>CURRENTLY MARRIED OR LIVING TOGETHER OR REMARRIED (Q510=1, 2 or 3) ☐ ↓<br><br>NOT IN A UNION ☐ → Q510=(4, 5, 6 OR 7)		Q609
Q605	Now let's talk about your partner and his preferences for the future.<br><br>***Yanzu zamuyi magana akan mijin /abokin zamanki da abin da ya fi so nan gaba.***<br><br>CHECK Q602:<br><br>NOT PREGNANT OR UNSURE ☐ ↓<br>Would he like to have (a/another) child, or would he prefer not to have any (more) children?<br><br>***Zai so ya samu wani da ko kuma baya son samun Karin 'ya 'ya***<br><br>PREGNANT ☐ ↓<br>After the child you are expecting now, would your partner like to have another child, or would he prefer not to have any more children?<br><br>**Bayan wannan haihuwar da ki ke sa rai yanzu, mijin ki /abokin zaman ki zai so samun wani da/ya ko baya son samun Karin 'ya'ya?**	HAVE (A/ANOTHER) CHILD . . . .1<br><br>NO MORE/NONE . . . . 2<br><br>SAYS SHE CAN'T GET PREGNANT………..……..….3<br><br>DON'T KNOW PARTNER'S DESIRE…………….…………….…..8	(2, 3, 8) → **Q607**
Q606	Again, this question relates to your partner's preferences for the future.<br>***Har yanzu, wannan tambayar ta shafi abinda mijin' ki/abokin zaman ki zai so nan gaba***<br><br>CHECK Q605:<br><br>NOT PREGNANT OR UNSURE ☐ ↓<br>How long would your partner/husband like to wait before the birth of (a/another) child?<br><br>**Har tsawon wane lokaci *mijin ki / abokin zaman ki zai so ya jira kafin ki sake haifan wani 'da?***<br><br>IF RESPONSE IS IN MONTHS AND YEARS (E.G. 2 ½ YEARS), CONVERT TO MONTHS (e.g. 30 months)<br><br>PREGNANT ☐ ↓<br>After the birth of the child that you and your partner are now expecting, how long would your partner like to wait before you have another child?<br><br>***Bayan wannan haihuwar da ku ke jira ke da mijin/ abokin zaman ki,har tsawon wanelokaci ku ke son ku jira kafin haifan wani*** *'da?*<br><br>IF RESPONSE IS IN MONTHS AND YEARS (E.G. 2 ½ YEARS), CONVERT TO MONTHS (e.g. 30 months)	MONTHS . . . . .1 [ ][ ]<br><br>YEARS . . . . 2 [ ][ ]<br><br>SOON/NOW . . . . 993<br><br>OTHER ________________ 996<br>(SPECIFY)<br><br>SAYS SHE CAN'T GET PREGNANT…994<br><br>DON'T KNOW PARTNERS DESIRE. . .998	
Q607	Does your husband/partner want the same number of children that you want, or does he want more or fewer than you want?<br><br>***Shin mijin ki/abokin zaman ki yana bukatar yawan 'ya 'ya kamar yadda ki ke so, ko yana son fiye da ki ke so, ko kasa da yadda ki ke so?***	SAME NUMBER……………………..1<br>MORE CHILDREN………………….…2<br>FEWER CHILDREN………………...3<br>DON'T KNOW……………………..…..8	
Q608	Who (will) decides how many children that you are going to have Would you say that it is mainly your decision, mainly your husband's/partner's decision, or do you decide together?<br><br>***Shin wa yake shawarar yara nawa za ki samu – Za ki iya cewa ra'ayinki ne ke kadai, ko na mijin ki/abokin zaman ki ne, ko dukkan ku biyu ku ke shawar ar?***	MAINLY YOU…………………..……1<br>MAINLY PARTNER…………………2<br>JOINTLY…………….……………...3<br><br>OTHER_____________________6<br>(SPECIFY)	

Q609	CHECK BIRTH HISTORY (Q216 & Q219): ANY LIVING CHILDREN?<br><br>Now let's talk about you again and your childbearing preferences.<br><br>PROBE FOR A NUMERIC RESPONSE.<br><br>HAS LIVING CHILDREN ☐<br>If you could go back to the time you did not have any children and could have exactly the number of children you wanted to have in your whole life, how many would that be?<br>***Idan zaki koma lokacin da baki fara samun yara ba kuma aka baki zabin yawan yaran da ki ke so a rayuwar ki, yara nawa za ki yi fatan samu?***<br><br>NO LIVING CHILDREN ☐<br>If you could have exactly the number of children you wanted to have in your whole life, how many would that be?<br>***Idan za ki iya samun yawan yaran da ki ke so a rayuwar ki,kamar nawa zaki so?***	NONE . . . . 00<br><br>NUMBER . . . . [__|__]<br><br>OTHER ______________________ 96<br>(SPECIFY)	**Q611**<br><br><br><br>**Q611**
Q610	**<u>Of this number</u>**, how many of these children would you like to be boys, how many would you like to be girls and for how many would the sex not matter?<br><br>**Cikin wannan jimlar, 'ya'ya nawa zaki fi so su kasance maza ne kuma 'ya'ya nawa zaki fi so su kasance mata ne kuma 'ya 'ya nawa su kasance ko wane babu damuwa?**	BOYS GIRLS EITHER<br>NUMBER [__|__] [__|__] [__|__]<br><br>OTHER ______________________ 96<br>(SPECIFY)	

Now, I would like to ask you a few questions about how other women like you think about children and births.

Yanzu, ina so nayi maki tambayoyi game da yadda wasu mata kamar ki, ke yin tunani game da yara da kuma haife haife

Q611	In your opinion, how long should a couple wait to have their first child?<br><br>***A ra'ayin ki har tsawon wanne lokaci ya kamata ma'aurata su jira kafin su samu haihuwar su ta farko?***	IMMEDIATELY…………………….1<br>LESS THAN ONE YEAR…………2<br>MORE THAN 1 YR BUT LESS THAN 2 YRS……………………...3<br>2 OR MORE YEARS…………..…4<br>OTHER ___________________6<br>(SPECIFY)<br>DON'T KNOW………………..….8	
Q612	In your opinion, what should be the ideal age in years for a woman to get her first child/ pregnancy?<br><br>***A ra'ayin ki kamar shekaru nawa ne ya kamata mace ta kai kafin ta samu ciki, ko, dan ta/yar ta na/ farko?***	AGE IN YEARS………………….[__|__]<br><br>DON'T KNOW……………………….98	
Q613	In your opinion, what should be the ideal age gap between two children?<br><br>***A ra'ayin ki kamar shekaru nawa ne ya kamata a samu tsakanini 'ya'ya biyu?***<br><br>IF RESPONSE IS IN MONTHS AND YEARS (E.G. 2.5 YEARS), CONVERT TO MONTHS (e.g. 30 months)	MONTHS………………….1 [__|__]<br>OR<br>YEARS…………………….2 [__|__]<br><br>DON'T KNOW…………………….998	

SECTION 7: SPOUSAL AND INTERPERSONAL COMMUNICATION			
**Qno**	**Questions and filters**	**Coding categories**	**Skip to**
Q701	Now, I would like to ask you a few questions on discussions that you might have had with other people on health issues.<br><br>***Yanzu zanyi miki wasu yan tamboyoyi akan tattaunawa da maiyiwa kika taba yi game da kiwon lafiya da wasu mutane.***<br><br>CHECK Q510:<br>CURRENTLY MARRIED OR LIVING TOGETHER (Q510=1, 2 OR 3) ☐	NOT IN A UNION (Q510= 4, 5,6, OR 7) ☐ →	**Q709**
Q702	How often have you talked to your spouse/partner about the number of children you would like to have in the last six (6) months?<br><br>***Sau nawa ki ka yi magana da mijin ki game da yawan yaran da zaki so ki samu a cikin watanni shida da suka shige?***	NUMBER OF TIMES TOPIC DISCUSSED ☐☐<br><br>NOT DISCUSSED AT ALL IN LAST SIX (6) MONTHS……………………………………………..97	
Q703	How often have you talked to your spouse/partner about the use of a family planning method in the last six (6) months?<br><br>***Sau nawa ki ka yi magana da mijin ki/ abokin zaman ki game da yin amfani da dabarar tsarin iyali a cikin watanni shida da suka shige?***	NUMBER OF TIMES TOPIC DISCUSSED ☐☐<br><br>NOT DISCUSSED AT ALL IN LAST SIX (6) MONTHS……………………………………………..97	
Q704	Who usually starts a discussion about family planning, you or your spouse/partner?<br><br>***Shin wa ya ke fara maganar tsarin iyali, ke ce ko mijin ki/abokin zamanki?***	YOU...1<br>SPOUSE/PARTNER..2<br>EITHER……………………………………..…….3<br>NEITHER / NEVER TALK ABOUT FP……………4	
Q705	How difficult is it to start a conversation about family planning with your spouse/partner – is it very difficult, somewhat difficult, or easy?<br><br>***Yaya wahalar fara maganar tsarin iyali/ tazara tsakanin haihuwa/tazara tsakanin yara da mijin/ abokin zaman ki – da wahala sosai, da dan wahala, da sauki?***	VERY DIFFICULT ……………………………..…..1<br>SOMEWHAT DIFFICULT ……………………..….2<br>EASY…………………………………………….…..3	
Q706	Do you intend to talk to your spouse/partner about family planning in the next three months?<br><br>***Shin kina da niyyar magana da mijin ki akan tsarin iyali/tazarar tsakanin haihuwa/tazara tsakanin yara a cikin watanni uku nan gaba?***	YES ……………………………………………..….1<br>NO…………………………………………………2<br>DON'T KNOW…………………………….……….8	
Q707	Does your husband/partner approve or disapprove of couples using a contraceptive method to avoid pregnancy/child birth spacing?<br><br>**Ko mijin ki zai iya amincewa ko bai amince ba da ma'aurata suyi amfani da dabarar tsarin iyali/ tazarar haihuwa/tazara tsakanin yara?**	APPROVE…. . . . . ….……….. ..1<br>DISAPPROVE. . . . . ….……….. .2<br>DON'T KNOW……………………………….……8	
Q708	Has your spouse/partner ever accompanied you (or your child) to a health facility?<br><br>**Ko mijinki/ abokin zamanki ya ta ba raka ki (ko yaro/yarinyar ku) zuwa cibiyar kiwon lafia?**	YES . . . . ….……….. 1<br>NO . . . . ….……….. .2	

Q709	Besides yourself (or your husband/partner) who else influences the decision whether you use a method of birth spacing or not?<br><br>**Banda ke kan ki (ko mijinki/abokin zamanki), raayin wanene zai shafi kudurin da zaki yi game da amfani da dabarar tsari iyali/ tazarar haihuwa ko rashin yin hakan?**<br><br>CIRCLE ALL MENTIONED.	MOTHER………………. ....A<br>MOTHER-IN-LAW …………....……….………B<br>SISTER(S)…………………………………….……C<br>SISTER(S)-IN-LAW……………………….…….D<br>GRANDMOTHER……………………….............E<br>FRIEND(S)……………………………………....F<br>HEALTH WORKER..G<br>COMMUNITY LEADER.....................................H<br>RELIGIOUS LEADER…………………………….I<br>AUNT……….…………………………………..…J<br>OTHER RELATIVES…………………………...K<br>OTHER ______________________________ X<br>(SPECIFY)<br>NO ONE……………………..…..……………..…Y	
Q710	If you wanted to use a method of family planning, would you need anyone's permission?<br><br>**Idan kina son ki yi amfani da dabarar tsarin iyali, kina bukatar daukar izini daga wani/wata?**	YES . . . .………………1<br>NO . . . .………………...2 →<br>DON'T KNOW……………………………………...8 →	 **Q712**<br>**Q712**
Q711	From whom would you need permission?<br><br>***A wurin wa zaki nemi izini?***<br><br>CIRCLE ALL MENTIONED.	HUSBAND/PARTNER……………………..…A<br>FATHER.. B<br>MOTHER…………. ....C<br>FATHER-IN-LAW..D<br>MOTHER-IN-LAW …………....…………….E<br>SISTER(S)…………………………………..…F<br>SISTER(S)-IN-LAW……………………….…..G<br>OTHER RELATIVE……………………….….H<br><br>OTHER ____________________________X<br>(SPECIFY)	
Q712	Do you think that government officials should speak publicly about family planning/child birth spacing?<br><br>***A ganin ki, ya kamata ma áikatan gwamnati su yi magana a wajen taro akan dabarun tsarin iyali/ tazara tsakanin haihuwa da haihuwa?***	YES…………….………………….…………..….1<br>NO………………………………….………...…2<br>DON'T KNOW ………………………………..……8	
Q713	In the past year, have you heard or seen a Local Government official speaking publicly **against** family planning/ child birth spacing?<br><br>***A cikin shekara daya da ta shige,kin ji ko kin ga wani jami'in Karamar Hukuma na magana a bainar jama'a kan rashin goyon bayan dabarun tsarin iyali/ tazara tsakanin haihuwa da haihuwa?***	YES…………………………….……………….1<br>NO……………………………………………..….2<br>DON'T REMEMBER …………………………..8	
Q714	In the past year, have you heard or seen a Local Government Official speaking publicly **in favor** of family planning/ child birth spacing?<br><br>***A cikin shekara daya da ta shige,Kin ji ko kin ga wani jami'in Karamar Hukuma na magana a bainar jama'a kan goyon bayan dabarun tsarin iyali/ tazara tsakanin haihuwa da haihuwa?***	YES……………………………………..……1<br>NO……………………………………………….2<br>DON'T REMEMBER ………………………….8	
Q715	Do you think that religious leaders should speak publicly about family planning/child birth spacing?<br><br>***A ganin ki shuwagabannin addini na iya magana a wajen taron jama' a kan mahimmancin tsarin iyali/tazara tsakanin haihuwa?***	YES . . . .…..……..…1<br>NO . . . .…..……….2<br>DON'T KNOW……………….…………………8	

Q716	In the past year, have you heard or seen a religious leader speaking publicly **against** family planning/child birth spacing?<br>***A cikin shekarar da ta shige kin taba jin ko ganin wani shugaban addini na magana a wajen taron jama' a kan rashin mahimmancin tsarin iyali/tazara tsakanin haihuwa/tazara tsakanin yara?***	YES…………………………………………..…1<br>NO……………………………………….....….2<br>DON'T REMEMBER …………………………8	
Q717	In the past year, have you heard or seen a religious leader speaking publicly **in favor of** family planning/child birth spacing?<br><br>***A cikin shekarar da ta shige kin taba jin ko ganin wani shugaban addini na magana a wajen taron jama' a kan goyon bayan tsarin iyali/tazara tsakanin haihuwa/tazara tsakanin yara?***	YES…………………………………………….1<br>NO……………………………………….…..2<br>DON'T REMEMBER ………………………...8	
Q718	How many of your close friends and relatives do you think use family planning: none, some, most, or all?<br><br>**Kawayanki da yanwuwan ki na kusa da ke, guda nawa ne su ke amfani da dabarar *tsarin iyali/tazara tsakanin haihuw/tazara tsakanin yara: ba ko daya, kadan daga cikin su, mafiya yawa daga cikin su, ko gaba ki dayansu?***	NONE………………………………………….1<br>SOME…………………………………………2<br>MOST…………………………………………3<br>ALL……………………………………..……4<br>DON'T KNOW……………………………..…..8	
Q719	Do you think there are some people within this community who will call you bad names or avoid your company if they knew that you were using a family planning/contraceptive method?<br><br>***Shin kina ganin cewa da akwai wasu mutane a cikin wannan al'uma da za su kira ki da sunaye mara sa kyau ko kuma baza su so su yi ma'amala dake ba in sun san kina amfani da dabarar tsarin iyali/tazara tsakanin haihuwa/tazara tsakanin yara?***	YES . . . . ….……….…..1<br>NO . . . . …………..…..2<br>DON'T KNOW……..…………………...…….8	
Q720	Do you think there are some people within this community who will praise, encourage, or talk favorably about you if they knew that you were using a family planning/contraceptive method?<br><br>***Shin kina ganin cewa da akwai wasu mutane a cikin wannan al'uma wanda zasu yabeki ko su baki goyon baya ko suyi magana mai kyau akan ki , in sun san kina amfani da dabarar tsarin iyali/tazara tsakanin haihuwa/tazara tsakanin yara?***	YES . . . . ….………..1<br>NO . . . . ….…….…..2<br>DON'T KNOW……..……….………………….8	
Q721	Are you willing to pay for family planning services (commodities and consumables)?<br><br>**Za ki iya biyan kudi domin samun al'amurran da suka shafi tsarin iyali (magunguna da kayan aikin)?**	YES . . . . ….………..1<br>NO . . . . ….…….…..2<br>DON'T KNOW……..……….………………….8	
Q722	Do you think that the government should offer free FP services?<br>(Commodities and consumables)?<br><br>**Kina gani ya kamata gwamnati ta rika bada magunguna da kayan aiki na dabarun tsarin iyali ga Al'uma kyauta?**	YES . . . . ….………..1<br>NO . . . . ….…….…..2<br>DON'T KNOW……..……….………………….8	

Please tell me how you would agree or disagree with the following statements.<br>***Don Allah Ina son ki gaya mini ko zaki yarda ko ba zaki yarda da wadannan maganganun ba.***		**Strongly Agree**<br><br>***Kin yarda sosai***	**Agree**<br><br>**Kin yarda kawai**	**Disagree**<br><br>**Ba ki yarda ba kawai**	**Strongly Disagree**<br><br>**Ba ki yarda ba sosai**
Q723	You could start a conversation with your partner about family planning/child birth spacing.<br><br>***Za ki iya fara magana da mijin/abokin zamanki akan tsarin iyali***	4	3	2	1
Q724	It is a woman's right to access and use family planning/child birth spacing<br><br>**Yanci da kuma damar mata ne samu da kuma amfani da hanyar kaiyade iyali/tazarar haihuwa?**	4	3	2	1
Q725	You could convince your spouse/partner that you should use a method of family planning/child birth spacing.<br><br>***Za ki iya shawo kan mijin/abokin zaman ki don ki fara amfani da dabarar tsarin iyali***	4	3	2	1
Q726	You could obtain a family planning/child birth spacing if you decided to use one.<br><br>***Za ki iya samun dabarar tsarin iyali /tazara tsakanin haihuwa/tazara tsakani yara*** **idan kina son ki yi amfani da shi**.	4	3	2	1
Q727	You could use a method of family planning/ child birth spacing even if your partner doesn't want you to.<br><br>***Za ki iya yin amfani da dabarar tsarin iyali /tazara tsakanin haihuwa/tazara tsakani yara ko mijin/abokin zaman ki in bai amince ba.***	4	3	2	1
Q728	You could use a method of family planning/child birth spacing even if none of your friends or neighbors uses one.<br><br>***Za ki iya yin amfani da dabarar tsarin iyali /tazara tsakanin haihuwa/tazara tsakani yara ko da kawayen ki ko makobtan ki basu amfani da shi***	4	3	2	1
Q729	You could use a method of family planning/child birth spacing even if your religious leader did not think you should use one.<br><br>***Za ki iya yin amfani da dabarar tsarin iyali /tazara tsakanin haihuwa/tazara tsakani yara ko da wani shugaban addinin yana ganin cewa bai kamata ki yi amfani dashi ba***.	4	3	2	1
Q730	You could continue to use a family planning/child birth spacing method even if you experience some side effects.<br><br>***Za ki iya cigaba da amfani da dabarar tsarin iyali/tazara tsakanin haihuwa/tazara tsakanin yara ko da kin samu wasu matsaloli lokacin da ki ke amfani da dabarun.***	4	3	2	1

**INSTRUCTIONS: Ask question Q731, then question Q732, Q733 AND Q734, for each person listed in the left column before moving on to the next person.**				
	Q731. In the past one year, have you talked about family planning with __________?<br><br>**A shekara daya da ta shige, kin yi maganar tsarin kaiyade iyali da---------------------?**	Q732. How important is this person's opinion to you with regard to family planning/birth spacing issues?<br><br>***Kamar yaya ki ka dauki muhimmanci ra'ayin wanan mutumin kan tsarin kaiyade iyali da tazarar haihuwa?***<br><br>***(ya na da muhimmanci ne sosai, muhimmanci kawai ko ba babu muhimmanci gaba daya)***	Q733. Do you think that this person would support your using a method of family planning?<br>***Ki na tunanin wannan*** *mutumin zai goyi bayanki kan tsarin* ***kaiyade iyali?***	Q734. Is this person's approval necessary for you to use family planning?<br>***Yanke hukuncin wannan mutumin yana da muhimmnci kan kiyi amfani da tsarin kaiyade iyali?***
a) Your mother<br>**mahaifiyar ki**	YES............. 1<br>NO.............. 2<br>NA………… 7→(**b**)	NOT AT ALL…….. 1<br>SOMEWHAT……… 2<br>VERY …………… 3	YES.................. 1<br>NO.................... 2<br>DK.................... 8	YES……… 1<br>NO………. 2
b) Mother-in-law<br>***uwar minjin ki***	YES............. 1<br>NO.............. 2<br>NA………… 7→(**c**)	NOT AT ALL…….. 1<br>SOMEWHAT……… 2<br>VERY …………… 3	YES.................. 1<br>NO.................... 2<br>DK.................... 8	YES……… 1<br>NO………. 2
c) Sister-in-law<br>**ya/kanwan mijin ki**	YES............. 1<br>NO.............. 2<br>NA………… 7→(**d**)	NOT AT ALL…….. 1<br>SOMEWHAT……… 2<br>VERY …………… 3	YES.................. 1<br>NO.................... 2<br>DK.................... 8	YES……… 1<br>NO………. 2
d) Father-in-law<br>**Mahaifin mijin ki**	YES............. 1<br>NO.............. 2<br>NA………… 7→(**e**)	NOT AT ALL…….. 1<br>SOMEWHAT……… 2<br>VERY …………… 3	YES.................. 1<br>NO.................... 2<br>DK.................... 8	YES……… 1<br>NO………. 2
e) Members of your religious community<br>**Aluma addinki**	YES............. 1<br>NO.............. 2<br>NA………… 7→**(f)**	NOT AT ALL…….. 1<br>SOMEWHAT……… 2<br>VERY …………… 3	YES.................. 1<br>NO.................... 2<br>DK.................... 8	YES……… 1<br>NO………. 2
f) Best friend?<br>***Babban kawar ki***	YES............. 1<br>NO.............. 2<br>NA………… 7→**Q801**	NOT AT ALL…….. 1<br>SOMEWHAT……… 2<br>VERY …………… 3	YES.................. 1<br>NO.................... 2<br>DK.................... 8	YES……… 1<br>NO………. 2

SECTION 8: GENDER INEQUITY MEASURES			
**Qno**	**Questions and filters**	**Coding categories**	**Skip to**
Now I would like to ask you some questions regarding your partner and how you and your partner make household decisions. Please remember to be as honest as possible and that your responses will remain confidential; that is, no one will see your answers.<br><br>**Yanzu ina son in tambayeki gameda abokin zamanki, kan yadda ke da abokin zamanki kuke yanke shawara bisa ga al'amurran da suka shafi gida. Ina son ki baiyana mani gaskiya kuma duk amsar da ki ka bani zasu kasance cikin sirri, babu wanda zai ga amsar da ki ka bayar.**			
Q801	CHECK Q510: CURRENTLY MARRIED<br><br>CURRENTLY MARRIED OR LIVING TOGETHER OR REMARRIED (Q510=1 OR 2 OR 3) ☐ ↓	NOT IN A UNION (Q510=4 OR 5 OR 6 OR 7) ☐ →	**Q811**
Q802	How old was your partner on his last birthday?<br>**Shekarar abokin zamanki nawa ne cikakku**?	AGE IN COMPLETED YEARS……………[__|__]	
Q803	Did your husband/partner ever attend school?<br>**Shin ko mijinki/abokin zamanki ya ta ba zuwa makaranta?**	YES . . . . ……………………………. 1<br>NO . . . . ……………………………… 2 →	 **Q806**
Q804	What was the highest level of school he attended: quranic, primary, secondary, or higher?<br><br>**Menene zurfin ilmin sa a karatun:**<br>**Kur'ani, firamare, sakandare, ko gaba da sakandare?**	QURANIC ONLY……………………………………… 0 →<br>PRIMARY………………………………………………… 1<br>JUNIOR SECONDARY (JSS)…......…………………… 2<br>SENIOR SECONDARY (SSS)……………………… 3<br>HIGHER………………………………………………. 4 } →<br>DON'T KNOW………………………………………… 8 }	**Q806**<br><br><br><br>**Q806**
Q805	What is the highest (class/year) he completed **<u>at that level?</u>**<br><br>**Menene aji /shekara mafi girma da ya kammala a wannan gurbin?**<br><br>RECORD "00" IF NO COMPLETED YEARS	CLASS/YEAR…………………….….[__|__]<br><br>DON'T KNOW………………………………98	
Q806	What is your partner's occupation, that is, what kind of work does he mainly do when he is/was working?<br><br>***Menene sana'ar mijin ki/abokin zamanki, watau wanne irin aiki ya ke yi ko yayi, lokacin da yake yin aiki****?*	HEALTH SERVICE PROVIDER<br>& RELATED WORKERS ………………………………….. 01<br>PROFESSIONAL, TECHNICAL, ADMINISTRATIVE,<br>MANAGERIAL & RELATED WORKERS ……………….. 02<br>SALES & RELATED WORKERS ………………………… 03<br>SERVICE, INSTALLATIONS, MAINTENANCE<br>& REPAIR WORKERS …………………………………… 04<br>AGRICULTURAL, ANIMAL HUSBANDRY, FORESTRY<br>WORKERS, FISHERMEN & HUNTERS ………………… 05<br>TRANSPORTATION, PRODUCTION,<br>CONSTRUCTION & EXTRACTION WORKERS ………. 06<br>STUDENT ONLY (NOT EMPLOYED) ………………….. 07<br>NOT EMPLOYED (NOT STUDENT) …………………….. 08<br>OTHER ______________________________ 96<br>(SPECIFY)	
Q807	Is your husband/partner currently working?<br><br>**Shin mijinki ko abokin zamanki na aiki yanzu?**	YES . . . . …………………… 1 →<br>NO . . . . …………………… 2<br>DON'T KNOW……...………………………………….. 8	**Q809**
Q808	What has he been doing for most of the time over the last 12 months?<br><br>***Wanne irin aiki ya fi yi a koda yaushe cikin watanni 12 da suka shige?***	NOTHING/IDLE………………………………………….. 01 }<br>LOOKING FOR WORK…………………………………. 02 }<br>GOING TO SCHOOL/STUDYING……………………. 03 }<br>UNABLE TO WORK DUE TO ILLNESS/DISABILITY.. 04 }<br>RETIRED…………………………………………………. 05 }<br><br>HOUSEHOLD/FARM WORK……………………….. 06<br>OTHER (SPECIFY)__________________________ 96	 <br>**Q811**

Q809	Does he usually work throughout the year, seasonally, or only once in a while?<br><br>**Yana aiki ne kusan ko wane lokaci a cikin shekara, aikin rani ko lokaci- lokaci?**	THROUGHOUT THE YEAR . . . . ……….. 1<br>SEASONALLY/PART OF THE YEAR . . …………….. 2<br>ONCE IN A WHILE . . . . ……….. 3	
Q810	Does he usually earn cash for this work?<br>**Yana samun kudi idan yayi aikin?**	YES . . . . …………………………1<br>NO . . . . …………………………… 2<br>DON'T KNOW……………………………………… 8	

Q811	What is your occupation, that is, what kind of work do you mainly do?<br><br>**Menene sana'ar ki, watau wanne irin aiki ki ka fi yi?**	HEALTH SERVICE PROVIDER<br>& RELATED WORKERS ………………………………….. 01<br>PROFESSIONAL, TECHNICAL, ADMINISTRATIVE,<br>MANAGERIAL & RELATED WORKERS ……………….. 02<br>SALES & RELATED WORKERS ………………………… 03<br>SERVICE, INSTALLATIONS, MAINTENANCE<br>& REPAIR WORKERS …………………………………… 04<br>AGRICULTURAL, ANIMAL HUSBANDRY, FORESTRY<br>WORKERS, FISHERMEN & HUNTERS ………………… 05<br>TRANSPORTATION, PRODUCTION,<br>CONSTRUCTION & EXTRACTION WORKERS ………. 06<br>STUDENT ONLY (NOT EMPLOYED) ………………….. 07<br>NOT EMPLOYED (NOT STUDENT) ……………………. 08<br>OTHER ……………………………………………………… 96<br>(SPECIFY) ______________________________________	
Q812	As you know, some women take up jobs for which they are paid in cash or kind. Others sell things, have a small business or work on the family farm or in the family business. In the last seven days, have **you** done any of these things or any other work?<br><br>**Kamar yadda ki ka sani, wasu matan na yin aikinda ake biyan su kudi ko kayan kudi.Wasu kuma suna kasuwanci ko yin aikin noma ko harkar kasuwancin iyali,**<br>**A cikin satin da ya shige, kin yi irin wannan aikin ko wani aiki?**	YES . . . . ………………………………… 1 →<br>NO . . . . ………………………………… 2	**Q814**
Q813	Have you done any work in the last 12 months?<br>**Kin yi wani aiki a cikin watanni 12 da suka shige?**	YES . . . . ………………………………… 1<br>NO . . . . ………………………………… 2 →	**Q819**
Q814	Do you do this work for a member of your family, for someone else, or are you self-employed?<br><br>**Ki na yin wannan aikin ne ma iyalinki ko wani mutum ko kuma kina yin aiki kanki ne?**	FOR FAMILY MEMBER . . . . ………….. 1<br>FOR SOMEONE ELSE . . . . ………………... 2<br>SELF-EMPLOYED…………………………..................... 3 →	**Q817**
Q815	Does your employer provide health care services on site for its employees?<br>**Shin wanda ki ke yi ma aiki na sanar daku akan al'amurran da suka shafi kiwon lafiya?**	YES . . . . ……………………….. 1<br>NO . . . . ……………………… 2<br>DON'T KNOW…………………………………………… 8	
Q816	Would you access family planning/contraceptive services if your employer offered the service on site (at the work place)?<br>**Ki na iya amsar hanyar tsarin kaiyade iyali idan wanda ya dauke ki aiki ya bayar dasu wajen aiki?**	YES . . . . ……………………. 1<br>NO . . . . ……………………... 2<br>DON'T KNOW………………………………………… 8	
Q817	Do you usually work throughout the year, or do you work seasonally, or only once in a while?<br><br>**Ki na aiki ne kowanne lokaci cikin shekara, Ko kina aikin rani ne ko jifa-jifa**?	THROUGHOUT THE YEAR . . . . ………. 1<br>SEASONALLY/PART OF THE YEAR . .. . ………… 2<br>ONCE IN A WHILE . . . . ………. 3	
Q818	Are you paid in cash or kind for this work, or are you not paid at all?<br>***Ana biyan ki kudi ne ko kayan kudi ko kuma ba'a biyanki?***	IN CASH ONLY………………………….……………… 1<br>IN CASH AND IN KIND………………….................. 2<br>IN KIND ONLY……………………………..……………. 3<br>NOT PAID……………………….……………………… 4	

Q819	Do you have health insurance?<br><br>**Ki na da inshorar kiwon lafiya?**	YES . . . . 1<br>NO . . . . 2 →<br>DON'T KNOW……………………………………… 8 →	 **Q822**<br>**Q822**
Q820	What type of health insurance do you have?<br>**Wacce irin Inshorar kiwon lafiya ki ke da ita?**	NATIONAL HEALTH INSURANCE …………………… 1<br>COMMUNITY HEALTH INSURANCE ……………… 2<br>OTHER________________________________ 6<br>(SPECIFY)<br>DON'T KNOW………………………………………… 8	
Q821	Does your health insurance cover family planning/child birth spacing services?<br>**Shin Inshorar ta kumshi hanyar kaiyade iyali da shirin tazarar haihuwa?**	YES . . . . 1<br>NO . . . . 2<br>DON'T KNOW……………………………………… 8	
Q822	Do you think health insurance should cover family planning/child birth spacing services?<br>**Ki na ganin cewa Inshorar ya kamta ta kumshi hanyar kaiyade iyali da shirin tazarar haihuwa?**	YES . . . . 1<br>NO . . . . 2<br>DON'T KNOW……………………………………… 8	
Q823	CHECK Q510: CURRENTLY MARRIED<br><br>CURRENTLY MARRIED OR LIVING TOGETHER OR REMARRIED (Q510=1 OR 2 OR 3) ☐ ↓	NOT IN A UNION (Q510=4 OR 5 OR 6 OR 7) ☐ →	**Q829**
Q824	CHECK Q813 AND Q818:<br><br>IF SHE IS CURRENTLY WORKING AND EARNS CASH (Q818=1 OR 2) ☐ ↓	IF SHE DOESN'T WORK OR DOES NOT EARN CASH (Q813=2 OR Q818=3 OR 4) ☐ →	**Q826**
Q825	Who decides how the money that you earn will be used: mainly you, mainly your partner, or you and your partner jointly?<br>**Wanene yake yanke shawarar yadda za ki kashe kudin da kika samu,ke da kan ki, mijin ki/abokin zamanki kadai ko ke da abokin zamanki?**	RESPONDENT . . . . 1<br>PARTNER . . . . 2<br>RESPONDENT AND PARTNER JOINTLY. ………… 3<br><br>OTHER ________________________________ 6<br>(SPECIFY)	
Q826	CHECK Q807 AND Q810:<br><br>IF HE IS CURRENTLY WORKING AND EARNS CASH (Q810=1) ☐ ↓	IF HE DOESN'T WORK OR DOES NOT EARN CASH (Q807=2 OR 8; OR Q810=2 OR 8) ☐ →	**Q828**
Q827	Who decides how the money that your partner earns will be used: Mainly you, mainly your partner, or you and your partner jointly?<br><br>**Wanene ke yanke shawarar yadda mijin/abokin zaman ki ya ke kashe kudin da ya samu?**<br>**ke da mijin ki/abokin zamanki, ke da kan ki, mijin ki/abokin zamanki kadai ko ke da abokin zamanki?**	RESPONDENT . . . . 1<br>PARTNER . . . . 2<br>RESPONDENT AND PARTNER JOINTLY. ………… 3<br><br>OTHER ____________________________ 6<br>(SPECIFY)	

Q828	Sometimes in a marriage or a relationship, a man prohibits his wife from doing certain things. Does your husband prohibit you from:<br>**Wani lokaci a dangantakar zaman aure, miji yakan hana mata yin wasu abubuwa. Shin ko mijinki yana hanaki yin wadannan:**	YES	NO
	a.Working outside the home?<br>**Aiki wajen gida?**	1	2
	b. Having visits from people?<br>**Ziyarar wasu mutane**	1	2
	c.Visiting your friends?<br>**Ziyarar kawaye?**	1	2
	d.Visiting your family?<br>**Ziyarar Iyalin ki?**	1	2
	e.Using contraceptives?<br>**Amfani da dabarar tsarin iyali?**	1	2
	f. Using a mobile phone?<br>**Amfani da wayar salula**	1	2

Q829	Do you have any money of your own that you alone can decide how to use?<br><br>**Kina da kudin kanki wanda ke da kanki za ki yi shawarar yadda za kiyi amfani da su**?	YES . . . . 1<br>NO 2	

Q830	In a couple, who do you think should have the greater say in each of the following decisions: the husband, the wife, or both equally:<br>**A ma'arauta, wanene ki ke tunanin yake da ta tacewa kan kowanne daga cikin wadannan shawarwarin: miji ne, mata ne, dukansu ne baki sani bane ko ya danganta**	HUSBAND<br><br>**MIJI NE**	WIFE<br><br>**MATAN NE**	BOTH EQUALLY<br><br>**DUK KANSU NE**	DK/DEPENDS<br><br>**BA KI SANI BA NE KO YA DANGANTA**
	a) Making large household purchases?<br>**Babbar sayayya a gida?**	1	2	3	8
	b) Making small daily household purchases?<br>**Sayayyar yau da kullum ta gida?**	1	2	3	8
	c)Deciding when to visit family, friends, or relatives?<br>**Yanke shawarar lokacin da za'a ziyarci iyali, kawaye/abokai ko 'yanuwa?**	1	2	3	8
	d)Deciding when and where to seek medical care for your own health?<br>***Yanke shawarar lokaci da wurin da za'a nemi kulawar kiwon lafiya?***	1	2	3	8

Q831	Sometimes a man is annoyed or angered by things that his wife does. In your opinion, is a man justified in hitting or beating his wife in the following situations?<br>***Wani lokaci ran miji yakan baci ko yayi hushi dalilin abubuwan da matar sa ta ke yi, A ra'ayinki, ya kamata miji ya bugi matarsa a waddannan yanayin?***	YES	NO	DON'T KNOW
	a. If she goes out without telling him?<br>**Idan ta fita ba tareda ta shaida ma sa ba**?	1	2	8
	b.If she neglects the house or the children?<br>**Idan ba ta kula da gida ko yara ba?**	1	2	8
	c. If she argues with him?<br>**Idan tayi gaddama da shi?**	1	2	8
	d. If she refuses to have sex with him?<br>***Idan ta ki yin jima'I da shi?***	1	2	8
	e.If she cooks the food improperly?<br>***Idan ba tayi girkin abinci dakyau ba?***	1	2	8
	f. If he suspects her of being unfaithful?<br>***Idan yana zargin ta da bin maza a waje?***	1	2	8
	g. . If she refuses to have another child?<br>**Idan ta ki yadda da ta kara haihuwa?**	1	2	8

Now I am going to read some statements to you about relationships and children. For each statement, please tell me your opinion as to whether you strongly agree, agree, disagree, or strongly disagree with it.

Yanzu ina son in karanta maki wasu maganganu gameda yanayin dangantaka da 'ya'ya. Ko wacce magana, ki shaida mani ra'ayinki kan ko kin yadda sosai, kin yadda kawai ko baki yadda ba, ko kuma ba ki yadda da maganar ba sosai?

		**Strongly Agree Kin yarda sosai**	**Agree Kin yarda kawai**	**Disagree Ba ki yarda ba kawai**	**Strongly disagree Baki yarda ba sosai**
Q832	The husband should be the one to decide whether the couple should use a family planning/birth spacing/child spacing method<br><br>**Miji shine wanda zai yanke shawarar ko ma'arauta suyi amfani da hanyar kaiyade iyali/tazarar haihuwa**	4	3	2	1
Q833	Couples who practice family planning have a better quality of life than those who do not.<br>***Ma'aratan daa suke amfani da hanyar kaiyade iyali suna da kyakkyawar rayuwa fiye da wadanda basa yi***	4	3	2	1
Q834	Husbands and wives should discuss family planning.<br>**Miji da mata su rika tattaunawa kan hanyar kayyade iyali.**	4	3	2	1
Q835	Men should not allow their wives to use family planning.<br>***Maza kada su bar matan su suyi amfani da hanyar kayyade tsarin iyali***	4	3	2	1
Q836	A woman who uses family planning without her husband's knowledge should be punished.<br>**Duk matar da tayi amfani da hanyar kayyade tsarin iyali ba tarei da sanin mijinta ba, a hukunta ta**	4	3	2	1
Q837	A woman who has no children is not complete/fulfilled.<br>***Duk macen da ba ta da 'ya'ya ba ta cika mace ba***	4	3	2	1
Q838	A man who has no children is not complete/fulfilled.<br>***Duk namijin da ba ya da 'ya'ya bai cika namiji ba***	4	3	2	1
Q839	It is good to have many children because one is not sure who among them will survive to care of the parents at old age.<br>***Yana dakyau idan aka haihu da yawa tun da mutum baya da tabbacin wanda zai kula dashi idan ya tsufa***	4	3	2	1
Q840	The number of children a couple will have is for God only to decide.<br>**Addadin yaya da ma'aurata zasu Haifa daga Allah yake**	4	3	2	1
Q841	A woman should continue bearing children until she has at least one son.<br>***Mace ta cigaba da haihuwa har sai ta samu 'da namiji***	4	3	2	1
Q842	A woman should continue bearing children until she has at least one daughter.<br>***Mace ta cigaba da haihuwa har sai ta samu 'ya mace***	4	3	2	1

	SECTION 9: MEDIA EXPOSURE		
**Qno**	**Questions and filters**	**Coding categories**	**Skip to**
Q901	Now I would like to talk to you about your information needs and where you get information regarding health issues.<br><br>**Yanzu ina son in yi maki tambaya game da yadda ki ke bukatar samun labarai da kuma yadda ki ke samun labaran da suka shafi harkar kiwon lafiya?**<br><br>What are your main sources for receiving health information?<br>**Wadanne hanyoyi ne ki ke samun labaran kiwon lafiya?**<br><br>PROBE SEPARATELY FOR:<br>A. Media sources: **Ta hanyar jaridun da ake bugawa ko wanda ake sauraro**<br>**B.** Health personnel sources: **Ta hanyar Jami'an kiwon lafiya**<br>C Community sources: **Ta hanyar al'umma**<br>D Interpersonal sources: **Ta mu'amulla da jama'a**<br><br>PROBE: Any other source? **Akwai wata hanyar kuma?**<br><br>(FOR EACH CATEGORY)<br><br>CIRCLE ALL MENTIONED.	**Media Sources**<br>RADIO .... AA<br>TV .... AB<br>VIDEOS .... AC<br>NEWSPAPERS .... AD<br>MAGAZINES/BOOKS .... AE<br>FLYERS/LEAFLETS/ POSTERS/ STICKERS........ AF<br>BILL BOARDS…….. AG<br>WALL PAINTING…….. AH<br>FACE BOOK…….. AI<br>INTERNET…….. AJ<br>E-MAIL…….. AK<br>SMS/CELLPHONE/TEXT MESSAGE…….. AL<br><br>**Health Personnel Sources**<br>CLINICAL OFFICER/DOCTOR…….. BA<br>NURSE/MIDWIFE…….. BB<br>COMMUNITY HEALTH WORKER…….. BC<br>PHARMACY/PHARMACIST…….. BD<br>PMV/CHEMIST…….. BE<br>HOSPITAL…….. BF<br>CLINIC …….. BG<br>TBA…….. BH<br>HERBALIST/TRADITIONAL HEALER…….. BI<br><br>**Community Sources**<br>CINEMA/MOBILE CINEMA…….. CA<br>VIDEO SHOPS/VIEWING CENTER…….. CB<br>SOCIAL/COMMUNITY HALLS…….. CC<br>COMMUNITY OUTREACH EVENTS (THEATRE, PUPPETS, ROAD SHOWS, ETC)…….. CD<br>CHANGE TO LIFE EVENTS (WEDDING, NAMING CEREMONY, HOUSEWARMING) …….. CE<br>PEER EDUCATION…….. CF<br>SCHOOL…….. CG<br>NGOS…….. CH<br>FBOS/CHURCH/MOSQUES…….. CI<br>COMMUNITY MEETINGS/ASSOCIATIONS…….. CJ<br>WOMEN'S/MEN'S GROUPS…….. CK<br>SOCIAL MOBILIZER/MOBILIZERS …….. CL<br><br>**Interpersonal Sources**<br>PARENTS…….. DA<br>IN-LAWS …….. DB<br>SPOUSE/PARTNER…….. DC<br>SIBLINGS…….. DD<br>SISTER/BROTHER IN LAWS…….. DE<br>FRIENDS/NEIGHBORS…….. DF<br>OTHER RELATIVES…….. DG<br>CHILDREN …….. DH<br><br>**OTHER SOURCES:** ___________ XX<br><br>NONE…….. YY<br>DON'T KNOW…….. ZZ	
Q902	Do you read newspaper(s)?<br><br>***Ki na karanta jaridu?***	YES .... 1<br>NO 2	

Q903	Do you read magazine(s)?<br><br>***Ki na karanta mujallu?***	YES ..... 1<br>NO 2	

Q904	CHECK: Q902 & 903: READ NEWSPAPERS AND/OR MAGAZINES<br>IF YES TO EITHER ☐ ↓	IF NO TO BOTH ☐ →	**Q907**
Q905	Have you read any articles on family planning/child birth spacing in newspapers/magazines in the past three months?<br><br>**Kin ta ba karanta wani sharhi kan tsarin kaiyade iyali/ tazarar haihuwa a jaridu ko mujallu a watanni 3 da suka shige?**	YES ..... 1<br>NO ..... 2 →	**Q907**
Q906	What information did you read in the newspapers/magazines about family planning/ birth spacing?<br><br>**Wanne labarai ki ka karanta a jaridu kan *dabarun tsarin iyali /tazara tsakanin haihuwa?***<br><br>MULTIPLE RESPONSES - CIRCLE ALL MENTIONED.<br><br>IF RESPONDENT SAYS "PILL", PROBE FURTHER TO ESTABLISH IF THEY MEAN THE "DAILY PILL" OR THE "EMERGENCY PILL"	**METHODS**<br>DAILY PILLS ..... A<br>IUD ..... B<br>MALE CONDOMS ..... C<br>FEMALE CONDOMS ..... D<br>INJECTABLES ..... E<br>IMPLANTS ..... F<br>EC/MORNING AFTER PILL/ POSTINOR 2 ..... G<br>FEMALE STERILIZATION ..... H<br>MALE STERILZATION ..... I<br>STANDARD DAYS METHOD (SDM) /CYCLE BEADS ..... J<br><br>**ISSUE**<br>AGE AT MARRIAGE ..... K<br>DELAYING FIRST BIRTH ..... L<br>DELAYING AGE AT FIRST SEX/ABSTINENCE ... M<br>SPACING BETWEEN BIRTHS ..... N<br>LIMITING FAMILY SIZE ..... O<br>GOVT STATEMENTS REGARDING FP ..... P<br>SPOUSE/PARTNER COMMUNICATION (TALK TO YOUR PARTNER) ..... Q<br>GO FOR FAMILY PLANNING ..... R<br>PROMOTING BENEFITS OF FP ..... S<br>PROVIDERS STATEMENTS REGARDING FP ..... T<br>RELIGIOUS LEADERS STATEMENTS REGARDING FP ..... U<br>TALK TO A HEALTH PROVIDER ABOUT FP ..... V<br><br>OTHERS (Specify)________ X<br>DON'TREMEMBER ..... Z	
Q907	Do you listen to the radio?<br><br>***Ki na sauraren rediyo?***	YES ..... 1<br>NO ..... 2 →	**Q913**
Q908	Which radio stations do you listen to?<br><br>***Wadanne tashoshin rediyo ki ke sauraro?***<br><br>LIST THE FULL NAME OF THE TOP THREE RADIO STATIONS MENTIONED	CODE BOXES: OFFICE USE ONLY<br>1.________ [ ][ ][ ]<br>2.________ [ ][ ][ ]<br>3.________ [ ][ ][ ]	
Q909	How many days in a week do you listen to radio?<br><br>***Kwanaki nawa a sati ki ke sauraren rediyo?***	NUMBER OF DAYS IN A WEEK [ ]<br><br>LESS THAN WEEKLY/NOT REGULARLY 8	
Q910	On average how long in total do you listen to radio on a normal day?<br><br>***A taikace tsawon lokaci nawa a yini ki ke jin rediyo?***	HOURS PER DAY ..... 1 [__|__]<br>OR<br>MINUTES PER DAY ..... 2 [__|__]	

Q911	Have you heard any family planning/ child birth spacing information on the radio in the past three months?<br>***Kin taba jin bayani a rediyo game da dabarun tsarin iyali /tazara tsakanin haihuwa a cikin watanni ukku da suka shige?***	YES.............................………..………………..1<br>NO...............................………….....................2	→ Q913

Q912	What information did you hear on the radio about family planning/birth spacing?<br><br>**Wanne labarai ki ka ji a rediyo kan *dabarun tsarin iyali /tazara tsakanin haihuwa?***<br><br>MULTIPLE RESPONSES - CIRCLE ALL MENTIONED.<br><br>IF RESPONDENT SAYS "PILL", PROBE FURTHER TO ESTABLISH IF THEY MEAN THE "DAILY PILL" OR THE "EMERGENCY PILL"	**METHODS**<br>DAILY PILLS ....A<br>IUD ....B<br>MALE CONDOMS .... ……C<br>FEMALE CONDOMS ……………………………..D<br>INJECTABLES…………………………………….. E<br>IMPLANTS………………………………………… F<br>EC/MORNING AFTER PILL/ POSTINOR 2 .... G<br>FEMALE STERILIZATION................................. H<br>MALE STERILZATION ....I<br>STANDARD DAYS METHOD (SDM)<br>/CYCLE BEADS .... J<br><br>**ISSUE**<br>AGE AT MARRIAGE…………………………………K<br>DELAYING FIRST BIRTH………………………….. L<br>DELAYING AGE AT FIRST SEX/ABSTINENCE….M<br>SPACING BETWEEN BIRTHS……………….……..N<br>LIMITING FAMILY SIZE……………………. ……….O<br>GOVT STATEMENTS REGARDING FP……….......P<br>SPOUSE/PARTNER COMMUNICATION<br>(TALK TO YOUR PARTNER) …………… …………Q<br>GO FOR FAMILY PLANNING ……………………… R<br>PROMOTING BENEFITS OF FP…………………… S<br>PROVIDERS STATEMENTS REGARDING FP …..T<br>RELIGIOUS LEADERS STATEMENTS<br>REGARDING FP ………………………………………U<br>TALK TO A HEALTH PROVIDER<br>ABOUT FP ……………………………………………..V<br><br>OTHERS (Specify)_________________________X<br>DON'TREMEMBER ……………………………………Z		
Q913	In your opinion, how acceptable or not acceptable are the following topics on radio?<br><br>***A ra'ayinki, menene amincewarki ko rashin amincewarki kan wadannan al'amuran a rediyo?***	ACCEPTABLE<br><br>**Kin amince**	UNACCEPTABLE<br><br>***Ko ba ki amince ba***	
	a. Family planning/birth spacing*:*<br>***Dabarun tsarin iyali /tazara tsakanin haihuwa***	1	2	
	b. HIV/AIDS:<br>***Ciwon sida/Kanjamau***	1	2	
	c. Maternal health (antenatal care, delivery services, postpartum care) *:*<br>**Kiwon lafiyar mata masu ciki (Kulawar daukar ciki,kulawar haihuwa da kulawar bayan haihuwa)**	1	2	
	d. Child health (immunizations, disease prevention, nutrition)<br><br>**Kiwon lafiyar yara(Allurar raga kafi, riga kafin cututtuka, abinci mai gina jiki)**	1	2	
	e. Reproductive health (STIs, infertility problems)<br><br>***Kiwon lafiya da ya shafi haihuwa(Cututtukan ciwon sanyi da matsalolin rashin haihuwa)***	1	2	

Q914	**ASK THE FOLLOWING QUESTION BASED ON CITY:**<br><br>**FCT ABUJA:**<br>Have you ever heard about a radio program called "Second Chance"?<br><br>**A cikin shekara daya da ta shige, kin ta ba ji ko ganin wannan kalmar "Mu hade tare"**<br><br>**IBADAN OR ILORIN:**<br>Have you ever heard about a radio program called "Ireti Eda"?<br><br>**KADUNA/ZARIA:**<br>Have you ever heard about a radio program called "Komai Nisan Jifa"?<br>**Kin ta ba sauraron wani shiri a gidan rediyo " komai nisan jifa''**<br><br>**BENIN CITY:**<br>Have you ever heard about a radio program called "Life Don Beta"?<br><br>**PROBE:** "You may have heard this program played on the radio or on a CD or through a radio listener's group"<br>**PROBE: Maiyuwa kin ji wannan shirin a rediyo ko "CD".**	YES……………………………………………………1<br><br>NO……………………………………………………2→<br><br>DON'T KNOW ……………………………………………8→	 <br>**Q919**<br><br>**Q919**
Q915	What was the program about?<br><br>***Menene shirin ya kumsa ?***<br><br>CIRCLE ALL MENTIONED	AGE AT MARRIAGE……………………………………A<br>DELAYING FIRST BIRTH……………………………..B<br>USE/GO FOR FAMILY PLANNING..…………………..C<br>DELAYING AGE AT FIRST SEX……………………...D<br>ABSTINENCE……………………………………………..E<br>SPACING BETWEEN BIRTHS………………………..F<br>LIMITING FAMILY SIZE………………………………G<br>TALK TO HEALTH PROVIDER ABOUT FP…………H<br>TALK TO SPOUSE/PARTNER ABOUT FP…….……..I<br>PLAN YOUR FAMILY …………………………………. J<br>GET IT TOGETHER……………………………………K<br><br>OTHERS (Specify)_________________________X<br>DON'T KNOW ……………………………………………Z	
Q916	Have you ever listened to this radio program?<br><br>***Kin ta ba sauraron wannan shirin?***	YES……………………………………………………1<br>NO……………………………………………………2→<br>DON'T KNOW…………………………………………..8→<br>DON'T LISTEN TO THE RADIO…………………….7→	 **Q918**<br>**Q918**<br>**Q918**
Q917	How often did you listen to this program – every week, once or twice a month, or less than once a month?<br><br>***Kin ta ba halartar wani taron da a ka sa wannan shirin ko ake tattaunawa akai?***	EVERY WEEK…………………………………………1<br>ALMOST EVERY WEEK……………………………..2<br>ONCE OR TWICE A MONTH………………………..3<br>LESS THAN ONCE A MONTH………………………4<br>USED TO LISTEN BUT DON'T ANYMORE……….5<br>ONLY LISTENED ONCE……………………………..6	
Q918	Have you ever attended a meeting where this program was played or discussed?<br><br>***Kin ta ba halartar wani taron da a ka sa wannan shirin ko ake tattaunawa akai?***	YES……………………………………………………1<br>NO……………………………………………………2<br>DON'T KNOW ……………………………………………8	
Q919	Have you heard a radio jingle or spot with people talking about family planning or child spacing during a naming ceremony?<br><br>***Kin ta ba jin wani sako a radio da ya ke maganan akan tazara sakanin haifuwa/tsarin Iyari a lokacin tauron suna?***	YES……………………………………………………1<br>NO……………………………………………………2 →<br>DON'T KNOW ……………………………………………8 →	 **Q921**<br>**Q921**

Q920	What were the key messages of this radio spot or jingle?<br><br>Mennene muhimmin abinda sakon nan radio ke dauke dashi?	AGE AT MARRIAGE……………………………………A<br>DELAYING FIRST BIRTH……………………………..B<br>USE/GO FOR FAMILY PLANNING..…………………..C<br>DELAYING AGE AT FIRST SEX……………………..D<br>ABSTINENCE…………………………………………..E<br>SPACING BETWEEN BIRTHS………………………..F<br>LIMITING FAMILY SIZE………………………………G<br>TALK TO HEALTH PROVIDER ABOUT FP…………H<br>TALK TO SPOUSE/PARTNER ABOUT FP…………..I<br>PLAN YOUR FAMILY ………………………………… J<br><br>OTHERS (Specify)_________________________X<br>DON'T KNOW ……………………………………………Z	
Q921	Have you heard a radio jingle or spot with people talking about family planning or child spacing in a hair dressing salon/ barbing salon?<br>***Kin ta ba jin wani sako a radio da mutane su ke magana akan tazara sakanin haifuwa/tsarin Iyali a wurin gyaran gashi (saloon) na mata?***	YES……………………………………………………….1<br>NO…………………………………………………….2<br>DON'T KNOW ……………………………………………8	 → **Q923**<br>→ **Q923**
Q922	What were the key messages of this radio spot or jingle?<br><br>Mennene muhimmin abinda sakon radio nan, ke dauke dashi?	AGE AT MARRIAGE……………………………………A<br>DELAYING FIRST BIRTH……………………………..B<br>USE/GO FOR FAMILY PLANNING..…………………..C<br>DELAYING AGE AT FIRST SEX……………………..D<br>ABSTINENCE…………………………………………..E<br>SPACING BETWEEN BIRTHS………………………..F<br>LIMITING FAMILY SIZE………………………………G<br>TALK TO HEALTH PROVIDER ABOUT FP…………H<br>TALK TO SPOUSE/PARTNER ABOUT FP…………..I<br>PLAN YOUR FAMILY ………………………………… J<br><br>OTHERS (Specify)_________________________X<br>DON'T KNOW ……………………………………………Z	
Q923	Have you heard a radio jingle or spot with a family planning service provider answering questions about FP or talking to a couple?<br>***Kin ta ba jin wani sako a radio da ma'aikaciyar kiwon lafiya ke ansa tambayyoyi akan tazaran sakanin haifuwa/tsarin iyali ko kuma sakanin ma aurata?***	YES……………………………………………………….1<br>NO…………………………………………………….2<br>DON'T KNOW ……………………………………………8	 → **Q925**<br>→ **Q925**
Q924	What were the key messages of this radio spot or jingle?<br><br>Mennene muhimmin abinda sakon radio nan, ke dauke dashi?	AGE AT MARRIAGE……………………………………A<br>DELAYING FIRST BIRTH……………………………..B<br>USE/GO FOR FAMILY PLANNING..…………………..C<br>DELAYING AGE AT FIRST SEX……………………..D<br>ABSTINENCE…………………………………………..E<br>SPACING BETWEEN BIRTHS………………………..F<br>LIMITING FAMILY SIZE………………………………G<br>TALK TO HEALTH PROVIDER ABOUT FP…………H<br>TALK TO SPOUSE/PARTNER ABOUT FP…………..I<br>PLAN YOUR FAMILY ………………………………… J<br><br>OTHERS (Specify)_________________________X<br>DON'T KNOW ……………………………………………Z	
Q925	Have you heard a radio jingle or spot with a couple talking about FP?<br>***Kin ta ba jin wani sako a radio da ma'aurata ke Magana akan tazaran sakanin haifuwa/tsarin iyali?***	YES……………………………………………………….1<br>NO…………………………………………………….2<br>DON'T KNOW ……………………………………………8	 → **Q927**<br>→ **Q927**
Q926	What were the key messages of this radio spot or jingle?<br><br>Mennene muhimmin abinda sakon radio nan, ke dauke dashi?	AGE AT MARRIAGE……………………………………A<br>DELAYING FIRST BIRTH……………………………..B<br>USE/GO FOR FAMILY PLANNING..…………………..C<br>DELAYING AGE AT FIRST SEX……………………..D<br>ABSTINENCE…………………………………………..E<br>SPACING BETWEEN BIRTHS………………………..F<br>LIMITING FAMILY SIZE………………………………G<br>TALK TO HEALTH PROVIDER ABOUT FP…………H<br>TALK TO SPOUSE/PARTNER ABOUT FP…………..I<br>PLAN YOUR FAMILY ………………………………… J<br><br>OTHERS (Specify)_________________________X<br>DON'T KNOW ……………………………………………Z	

Q927	Have you heard a woman sharing her experience supporting the use of FP (Testimonial) on radio?<br><br>Kin taba jin sako a radio da wata mace ke shaidawa game da kwarewan ta da kuma goyobayan ta akan tazara sakanin haihuwa/tsarin iyali?	YES…………………………………………………….1<br>NO……………………………………………………….2<br>DON'T KNOW ……………………………………………..8	 **Q929**<br>**Q929**
Q928	What were the key messages of this radio spot or jingle?<br><br>Mennene muhimmin abinda sakon radio nan, ke dauke dashi?	AGE AT MARRIAGE……………………………………A<br>DELAYING FIRST BIRTH……………………………..B<br>USE/GO FOR FAMILY PLANNING..…………………..C<br>DELAYING AGE AT FIRST SEX……………………..D<br>ABSTINENCE……………………………………………..E<br>SPACING BETWEEN BIRTHS………………………..F<br>LIMITING FAMILY SIZE………………………………G<br>TALK TO HEALTH PROVIDER ABOUT FP…………H<br>TALK TO SPOUSE/PARTNER ABOUT FP…………..I<br>PLAN YOUR FAMILY ………………………………… J<br><br>OTHERS (Specify)_________________________X<br>DON'T KNOW ……………………………………………Z	
Q929	Do you watch television?<br><br>***Ki na kallon talabijin?***	YES ……………………………………………………1<br><br>NO…………………………………………………………2	 <br>**Q936**
Q930	How many days in a week do you watch television?<br><br>***Kwana ki nawa a sati ki ke kallon talabijin?***	NUMBER OF DAYS PER WEEK [ ]<br><br>NOT REGULARLY………………………….…8	
Q931	On average how long in total do you watch television on a normal day?<br>***A kalla kamar sau nawa kike kallon talabijin a rana?***	HOURS PER DAY…………………1 [__|__]<br><br>OR<br><br>MINUTES PER DAY………………2 [__|__]	
Q932	What channels do you generally watch on TV?<br><br>LIST THE FULL NAME OF THE TOP THREE TV STATIONS MENTIONED<br>**Wadanne tashoshi ki ka fi kallo akasari?**	CODE BOXES:<br>OFFICE USE ONLY<br><br>1.________________________________ [ ][ ][ ]<br>2.________________________________ [ ][ ][ ]<br>3.________________________________ [ ][ ][ ]	

Q933	What kind of TV programs do you normally watch?<br><br>CIRCLE ALL MENTIONED.<br><br>**Wanne shirin talebijin ki ke kallo?**	NEWS/CURRENT AFFAIRS……………………A<br>RELIGIOUS PROGRAMS..……………………..B<br>TALK SHOWS…………………………………….C<br>NATURE PROGRAMS…………………………..D<br>DOCUMENTARIES……………………………...E<br>SOAP OPERAS/DRAMA………………………..F<br>CARTOONS……………………………………….G<br>MUSIC TELEVISION……………………………..H<br>POLITICAL DEBATES……………………………I<br>SPORTS…………………………………………...J<br>CONTESTS………………………………………..K<br>FILMS/MOVIES…………………………………..L<br><br>OTHER ______________________________X<br>(SPECIFY)	
Q934	Have you seen any family planning/child birth spacing programs/ information on the TV in the past three months?<br><br>***Shin ko kin ga shirye- shirye / bayanai game da dabarar tsarin iyali /tazara tsakanin haihuwa da haihuwa a cikin watanni uku da suka shige a talabijin?***	YES……………………………………………1<br><br>NO…………………………………………….2	 <br>**Q936**

Q935	What information did you see on the TV about family planning/birth spacing?<br><br>**Wanne labarai ki ka gani a talebijin gameda *dabarar tsarin iyali /tazara tsakanin haihuwa?***<br><br>MULTIPLE RESPONSES - CIRCLE ALL MENTIONED.<br><br>IF RESPONDENT SAYS “PILL”, PROBE FURTHER TO ESTABLISH IF THEY MEAN THE “DAILY PILL” OR THE “EMERGENCY PILL”	**METHODS**<br>DAILY PILLS... A<br>IUD .... B<br>MALE CONDOMS... C<br>FEMALE CONDOMS ……………………………D<br>INJECTABLES…………………………………… E<br>IMPLANTS………………………………………… F<br>EC/MORNING AFTER PILL/ POSTINOR 2.........G<br>FEMALE STERILIZATION .... H<br>MALE STERILZATION..I<br>STANDARD DAYS METHOD (SDM)<br>/CYCLE BEADS ....J<br><br>**ISSUE**<br>AGE AT MARRIAGE…………………………….. K<br>DELAYING FIRST BIRTH………………………. L<br>DELAYING AGE AT FIRST SEX/ABSTINENCE…M<br>SPACING BETWEEN BIRTHS……………………N<br>LIMITING FAMILY SIZE…………………… ………O<br>GOVT STATEMENTS REGARDING FP……….. P<br>SPOUSE/PARTNER COMMUNICATION<br>(TALK TO YOUR PARTNER) …………… ………. Q<br>GO FOR FAMILY PLANNING …………………….. R<br>PROMOTING BENEFITS OF FP………………….. S<br>PROVIDERS STATEMENTS REGARDING FP …..T<br>RELIGIOUS LEADERS STATEMENTS<br>REGARDING FP ………………………………………U<br>TALK TO A HEALTH PROVIDER<br>ABOUT FP ……………………………………………..V<br><br>OTHERS (Specify)_________________________X<br>DON’TREMEMBER ………………………………….Z	

Q936	Have you seen a television jingle or spot that shows people talking about family planning or child spacing during a naming ceremony?<br>**Kin ta ba ganin wani shirin talebijin ko wani dandali da yake nuna jama’a na magana gameda *dabarar tsarin iyali /tazara tsakanin haihuwa lokacin bikin zana sunan jariri?***	YES……………………………………………………..1<br>NO……………………………………………………..2<br>DON’T KNOW ....…...8	 →Q938<br>→Q938
Q937	What were the key messages of this television spot or jingle?<br><br>**Menene muhimman sakonni da wannan shirin talebijin yake bayarwa?**	AGE AT MARRIAGE……………………………………A<br>DELAYING FIRST BIRTH……………………………...B<br>USE/GO FOR FAMILY PLANNING..…………………..C<br>DELAYING AGE AT FIRST SEX……………………...D<br>ABSTINENCE……………………………………………E<br>SPACING BETWEEN BIRTHS………………………..F<br>LIMITING FAMILY SIZE………………………………G<br>TALK TO HEALTH PROVIDER ABOUT FP…………H<br>TALK TO SPOUSE/PARTNER ABOUT FP…………..I<br>PLAN YOUR FAMILY ………………………………… J<br>GET IT TOGETHER……………………………………K<br><br>OTHERS (Specify)__________________________X<br>DON’T KNOW ....…….Z	
Q938	Have you seen a television jingle or spot that shows people talking about family planning or child spacing in a hair dressing salon/ barbing salon?<br>**Kin ta ba ganin wani shirin talebijin ko wani dandali da yake nuna jama’a na magana gameda *dabarar tsarin iyali /tazara tsakanin haihuwa a wajen gyaran gashi ko kitso ko kuma gun askin maza?***	YES……………………………………………………..1<br>NO……………………………………………………..2<br>DON’T KNOW ....…...8	 →Q940<br>→Q940

Q939	What were the key messages of this television spot or jingle?<br><br>**Menene muhimman sakonni da wannan shirin talebijin yake bayarwa?**	AGE AT MARRIAGE……………………………………A<br>DELAYING FIRST BIRTH……………………………...B<br>USE/GO FOR FAMILY PLANNING...………………...C<br>DELAYING AGE AT FIRST SEX……………………...D<br>ABSTINENCE…………………………………………..E<br>SPACING BETWEEN BIRTHS………………………..F<br>LIMITING FAMILY SIZE………………………………G<br>TALK TO HEALTH PROVIDER ABOUT FP…………H<br>TALK TO SPOUSE/PARTNER ABOUT FP…………..I<br>PLAN YOUR FAMILY ………………………………… J<br>GET IT TOGETHER……………………………………K<br><br>OTHERS (Specify)_________________________X<br>DON'T KNOW ....…….Z	
Q940	Have you seen a television jingle or spot that shows a family planning service provider answering questions about FP or talking to a couple?<br>**Kin ta ba ganin wani shirin talebijin ko wani dandali da yake nuna jami'in kaiyade tsarin iyali na amsa tambayoyi gameda *dabarar tsarin iyali ko ya/ta na Magana da ma'arauta?***	YES……………………………………………………..1<br>NO………………………………………………………2<br>DON'T KNOW ....…...8	 →Q942<br>→Q942
Q941	What were the key messages of this television spot or jingle?<br><br>**Menene muhimman sakonni da wannan shirin talebijin yake bayarwa?**	AGE AT MARRIAGE……………………………………A<br>DELAYING FIRST BIRTH……………………………...B<br>USE/GO FOR FAMILY PLANNING...………………...C<br>DELAYING AGE AT FIRST SEX……………………...D<br>ABSTINENCE…………………………………………..E<br>SPACING BETWEEN BIRTHS………………………..F<br>LIMITING FAMILY SIZE………………………………G<br>TALK TO HEALTH PROVIDER ABOUT FP…………H<br>TALK TO SPOUSE/PARTNER ABOUT FP…………..I<br>PLAN YOUR FAMILY ………………………………… J<br>GET IT TOGETHER……………………………………K<br><br>OTHERS (Specify)_________________________X<br>DON'T KNOW ....…….Z	
Q942	Have you seen a television jingle or spot that shows a couple talking about FP?<br><br>**Kin ta ba ganin wani shirin talebijin ko wani dandali da yake nuna ma'aurata na Magana gameda kaiyade tsarin iyali?**	YES……………………………………………………..1<br>NO………………………………………………………2<br>DON'T KNOW ....…....8	 →Q944<br>→Q944
Q943	What were the key messages of this television spot or jingle?<br>**Menene muhimman sakonni da wannan shirin talebijin yake bayarwa?**	AGE AT MARRIAGE……………………………………A<br>DELAYING FIRST BIRTH……………………………...B<br>USE/GO FOR FAMILY PLANNING...………………...C<br>DELAYING AGE AT FIRST SEX……………………...D<br>ABSTINENCE…………………………………………..E<br>SPACING BETWEEN BIRTHS………………………..F<br>LIMITING FAMILY SIZE………………………………G<br>TALK TO HEALTH PROVIDER ABOUT FP…………H<br>TALK TO SPOUSE/PARTNER ABOUT FP…………..I<br>PLAN YOUR FAMILY ………………………………… J<br>GET IT TOGETHER……………………………………K<br><br>OTHERS (Specify)_________________________X<br>DON'T KNOW ....…….Z	
Q944	Have you seen a woman on television sharing her experience supporting the use of FP (Testimonial)?<br>**Kin ta ba ganin mata a talebijin ta na baiyana ma jama'a goyon bayanta kan amfanin kaiyade tsarin iyali (Shaida)?**	YES……………………………………………………..1<br>NO………………………………………………………2<br>DON'T KNOW ....…...8	 →Q946<br>→Q946
Q945	What were the key messages of this television spot or jingle?<br><br>**Menene muhimman sakonni da wannan shirin talebijin yake bayarwa?**	AGE AT MARRIAGE……………………………………A<br>DELAYING FIRST BIRTH……………………………...B<br>USE/GO FOR FAMILY PLANNING...………………...C<br>DELAYING AGE AT FIRST SEX……………………...D<br>ABSTINENCE…………………………………………..E<br>SPACING BETWEEN BIRTHS………………………..F<br>LIMITING FAMILY SIZE………………………………G<br>TALK TO HEALTH PROVIDER ABOUT FP…………H<br>TALK TO SPOUSE/PARTNER ABOUT FP…………..I<br>PLAN YOUR FAMILY ………………………………… J<br>GET IT TOGETHER……………………………………K<br><br>OTHERS (Specify)_________________________X<br>DON'T KNOW ....…….Z	

Q946	In your opinion, how acceptable or not acceptable are the following topics on TV?<br><br>***A ra'ayinki, menene amincewarki ko rashin amincewarki kan wadannan al'amuran a Talebijin?***	**ACCEPTABLE**<br><br>**Kin amince**	**UNACCEPTABLE**<br><br>**Ko ba ki amince ba**	
	a. Family planning/birth spacing *:* ***Dabarun tsarin iyali /tazara tsakanin haihuwa***	1	2	
	b. HIV/AIDS: ***Ciwon sida/Kanjamau***	1	2	
	c. Maternal health (antenatal care, delivery services, postpartum care) : **Kiwon lafiyar mata masu ciki (Kulawar daukar ciki,kulawar haihuwa da kulawar bayan haihuwa**	1	2	
	d. Child health (immunizations, disease prevention, nutrition) **Kiwon lafiyar yara(Allurar raga kafi, riga kafin cututtuka, abinci mai gina jiki)**	1	2	
	e. Reproductive health (STIs, infertility problems): ***Kiwon lafiya da shafi haihuwa(Cututtukan ciwon sanyi da matsalolin rashin haihuwa)***	1	2	
Q947.	Do you go to video shows/viewing centers?<br><br>***Ki na zuwa wajen kallon bidiyo ko wajen da jama'a ke taruwa domin kallo?***	YES………………………………….1<br>NO……………………………………2 →		**Q949**
Q948	How **often** do you go to video shows?<br><br>**Kamar sau nawa kike zuwa wajen kallon bidiyo?**<br>SINGLE RESPONSE	MORE THAN ONCE PER WEEK………..1<br>EVERY WEEK ……………………………..2<br>A COUPLE OF TIMES A MONTH ………..3<br>AT LEAST ONCE A MONTH ……………4<br>AT LEAST ONCE A YEAR ……………….5<br>RARELY…………………………………….6		
Q949	Do you have access to mobile phone?<br><br>**Kina da wayar salula?**	YES…………………………………………..1<br>NO……………………………………………2 →<br><br>DON'T KNOW MOBILE PHONE…………..8 →		**Q953**<br><br>**Q953**
Q950	Do you own a mobile phone that is mainly for your own use?<br><br>***Shin kina da wayar salular da ke kadai kike amfani da ita?***	YES……………………………………………..1<br>NO………………………………………………2 →		**Q953**
Q951	In the past six(6) months, have you received any family planning/child birth spacing messages on your mobile phone?<br><br>**Kin sami wani sakon kaiyade tsarin iyali/tazarar haihuwa a wayar salular ki a watanni 6 da suka wuce?**	YES……………………………………………1<br>NO………………………………………………2 →<br>DON'T KNOW…………………………………8 →		**Q953**<br>**Q953**
Q952	How useful was the family planning message you received on your mobile phone?<br><br>**Shin sakon da kika samu ta wayar salular ya na da amfani a gareki?**	VERY USEFUL…………………………………...1<br>SOMEWHAT USEFUL…………………………2<br>NOT USEFUL……………………………………..3<br>DON'T KNOW……………………………………..8		
Q953	In the past year, have you heard or seen the phrase "Get it Together"<br><br>*A* ***cikin shekara daya da ta shige, kin ta ba ji ko ganin wannan kalmar "Mu hade tare"***	YES…………………………………………………1<br><br>NO……………………………………………………..2 →<br><br>DON'T KNOW……………………………………8 →		**Q957**<br><br>**Q957**
Q954	What does this phrase mean to you?<br>***Me wannan kalmar ta ke nufi?***<br><br>CIRCLE ALL MENTIONED	AGE AT MARRIAGE…………………………………A<br>DELAYING FIRST BIRTH……………………………B<br>USE/GO FOR FAMILY PLANNING……………………C<br>DELAYING AGE AT FIRST SEX……………………..D<br>ABSTINENCE…………………………………………..E<br>SPACING BETWEEN BIRTHS………………………..F<br>LIMITING FAMILY SIZE……………………………….G<br>TALK TO HEALTH PROVIDER ABOUT FP…………H<br>TALK TO SPOUSE/PARTNER ABOUT FP…………..I<br>PLAN YOUR FAMILY ……………………………… J<br>GET IT TOGETHER……………………………………K<br><br>OTHERS (Specify)_________________________X<br>DON'T KNOW …………………………………… …….Z		

Q955	Where did you see or hear this phrase?<br><br>***A ina ki ka ga ko ki ka ji wannan kalmar?***<br><br>CIRCLE ALL MENTIONED	RADIO DRAMA PROGRAM......A<br>RADIO JINGLE/SPOT......B<br>TELEVISION......C<br>NEWSPAPER ......D<br>POSTER ......E<br>UMBRELLA......F<br>SHOPPING BAG......G<br>TSHIRT...... ......H<br>LEAFLET/STICKERS......I<br>BADGE/BUTTON......J<br>CAPS......K<br>WRISTBAND......L<br>ON A SIGN AT A HEALTH FACILITY......M<br>ON A SIGN AT A PHARMACY / CHEMIST......N<br>AT A ROADSHOW / RALLY......O<br>AT A PARADE......P<br>BILLBOARD......Q<br>PENS......R<br><br>OTHER (Specify)________________X<br>DON'T KNOW......Z	

Q956	In the past year, have you attended any meetings about family planning/child birth spacing that were led by someone wearing a T-shirt with this phrase?<br><br>***A cikin shekara daya da ta wuce, kin ta ba halartar taro gameda tsarin iyali/tazarar haihuwa da aka kaddamar wadda wani yake sanye da riga mai dauke da wannan kalmar****?*	YES......1<br>NO......2<br>DON'T KNOW......8	
Q957	In the past year, have you seen/heard the phrase "Know, Talk. Go"?<br>***A cikin shekara daya da ta wuce, Kin ta ba jin wannan kalmar "ki sani, ki yi maganar, Je ki"***	YES......1<br>NO......2 →<br><br>DON'T KNOW......8 →	**Q960**<br><br>**Q960**
Q958	What does this phrase mean to you?<br><br>***Me wannan kalmar ta ke nufi?***<br><br>CIRCLE ALL MENTIONED	AGE AT MARRIAGE......A<br>DELAYING FIRST BIRTH......B<br>USE/GO FOR FAMILY PLANNING......C<br>DELAYING AGE AT FIRST SEX......D<br>ABSTINENCE......E<br>SPACING BETWEEN BIRTHS......F<br>LIMITING FAMILY SIZE......G<br>TALK TO HEALTH PROVIDER ABOUT FP......H<br>TALK TO SPOUSE/PARTNER ABOUT FP......I<br>PLAN YOUR FAMILY ...... J<br>GET IT TOGETHER......K<br><br>OTHERS (Specify)________________X<br>DON'T KNOW ......Z	
Q959	Where did you see or hear this phrase?<br><br>***A ina ki ka gani ko ki ka ji wannnan kalmar?***<br><br>CIRCLE ALL MENTIONED	RADIO DRAMA PROGRAM......A<br>RADIO JINGLE/SPOT ......B<br>TELEVISION......C<br>NEWSPAPER ......D<br>POSTER ......E<br>UMBRELLA......F<br>SHOPPING BAG......G<br>TSHIRT...... ......H<br>LEAFLET/STICKERS......I<br>BADGE/BUTTON......J<br>CAPS......K<br>WRISTBAND......L<br>ON A SIGN AT A HEALTH FACILITY......M<br>ON A SIGN AT A PHARMACY / CHEMIST......N<br>AT A ROADSHOW / RALLY......O<br>AT A PARADE......P<br>BILLBOARD......Q<br>PENS......R<br><br>OTHER (Specify)________________X<br>DON'T KNOW......Z<br>DON'T KNOW......Z	

Q960	In the past year, have you seen/heard the phrase ""No dulling"?<br><br>***A cikin shekara daya da ta wuce, kin ta ba jin wannan kalmar ""No dulling"?***	YES……………………………………………………..1<br>NO………………………………………………………2 →<br>DON'T KNOW…………………………………………8 →	 **Q963**<br>**Q963**

Q961	What does this phrase mean to you?<br><br>***Me wannan kalmar ta ke nufi?***<br><br>CIRCLE ALL MENTIONED	AGE AT MARRIAGE……………………………………A<br>DELAYING FIRST BIRTH……………………………..B<br>USE/GO FOR FAMILY PLANNING.…………………..C<br>DELAYING AGE AT FIRST SEX……………………..D<br>ABSTINENCE…………………………………………..E<br>SPACING BETWEEN BIRTHS………………………..F<br>LIMITING FAMILY SIZE………………………………G<br>TALK TO HEALTH PROVIDER ABOUT FP…………H<br>TALK TO SPOUSE/PARTNER ABOUT FP…………..I<br>PLAN YOUR FAMILY ………………………………… J<br>GET IT TOGETHER……………………………………K<br><br>OTHERS (Specify)__________________________X<br>DON'T KNOW ………………………………………..Z	
Q962	Where did you see or hear this phrase?<br><br>***A ina ki ka gani ko ki ka ji wannnan kalmar?***<br><br>CIRCLE ALL MENTIONED	RADIO DRAMA PROGRAM...…………..……….……..A<br>RADIO JINGLE/SPOT …...……………………..………..B<br>TELEVISION……………………………….…………….C<br>NEWSPAPER ………………………………………..…..D<br>POSTER ………………………………………………….E<br>UMBRELLA………………………..………………..…….F<br>SHOPPING BAG……………………….………………G<br>TSHIRT………. …………………………………………...H<br>LEAFLET/STICKERS…………………………………..I<br>BADGE/BUTTON……………………………………….J<br>CAPS……………………………………………………K<br>WRISTBAND……………………….………………….L<br>ON A SIGN AT A HEALTH FACILITY……………….M<br>ON A SIGN AT A PHARMACY / CHEMIST…………N<br>AT A ROADSHOW / RALLY…………………………..O<br>AT A PARADE…………………………………………..P<br>BILLBOARD……………………………………………..Q<br>PENS……………………………………………………..R<br><br>OTHER (Specify)___________________________X<br>DON'T KNOW…………………………………………….Z	
Q963	Do you understand Yoruba language?<br><br>***Kina jin harshen Yarbanci?***	YES……………………………………………………1<br>NO……………………………………………………...2 →	 **Q973**
Q964	In the past year, have you heard the phrase "Se o jasi" – (meaning "Are you into it?" or Are you part of it")?<br><br>***A shekara daya da ta shige, kin ji wannan kalmar "Se o jasi" –(ma'ana " Kina cikinta"? Ko kina da alaka da ita"***	YES……………………………………………………1<br>NO……………………………………………………...2 →<br><br>DON'T KNOW…………………………………………8 →	 **Q967**<br><br>**Q967**
Q965	What does this phrase mean to you?<br><br>***Menene wannan Kalmar ta ke nufi?***<br><br>[IF RESPONDENT ONLY GIVES THE COMMON DEFINITION OF THE PHRASE, PROBE TO SEE IF THERE IS ANY OTHER MEANING.<br><br>CIRCLE ALL MENTIONED	AGE AT MARRIAGE……………………………………A<br>DELAYING FIRST BIRTH……………………………..B<br>USE/GO FOR FAMILY PLANNING.…………………..C<br>DELAYING AGE AT FIRST SEX……………………..D<br>ABSTINENCE…………………………………………..E<br>SPACING BETWEEN BIRTHS………………………..F<br>LIMITING FAMILY SIZE………………………………G<br>TALK TO HEALTH PROVIDER ABOUT FP…………H<br>TALK TO SPOUSE/PARTNER ABOUT FP…………..I<br>PLAN YOUR FAMILY ………………………………… J<br>GET IT TOGETHER……………………………………K<br><br>OTHERS (Specify)__________________________X<br>DON'T KNOW ………………………………………..Z	
Q966	Where did you see or hear this phrase?<br><br>***A ina ki ga ko ki ka ji wannan Kalmar?***	RADIO DRAMA PROGRAM...…………..……….……..A<br>RADIO JINGLE/SPOT …...……………………..………..B<br>TELEVISION……………………………….…………….C<br>NEWSPAPER ………………………………………..…..D<br>POSTER ………………………………………………….E<br>UMBRELLA………………………..………………..…….F	

	CIRCLE ALL MENTIONED	SHOPPING BAG……………………………………….G<br>TSHIRT………. …………………………………………..H<br>LEAFLET/STICKERS…………………………………..I<br>BADGE/BUTTON……………………………………….J<br>CAPS………………………………………………………K<br>WRISTBAND……………………………………………L<br>ON A SIGN AT A HEALTH FACILITY……………….M<br>ON A SIGN AT A PHARMACY / CHEMIST…………N<br>AT A ROADSHOW / RALLY………………………….O<br>AT A PARADE………………………………………….P<br>BILLBOARD……………………………………………..Q<br>PENS……………………………………………………..R<br><br>OTHER (Specify)____________________________X<br>DON'T KNOW……………………………………………Z	
Q967	In the past year, have you heard the phrase "Mo ti feto si" – Iwo nko? (Meaning "I have planned my own, what about you?)<br><br>***A shekara daya da ta shige, Kin ji wannan kalmar."Mo ti feto si" – Iwo nko? (Ma'ana "Na tsara nawa, ke fa?***	YES………………………………………………………1<br><br>NO……………………………………………………….2 →<br><br>DON'T KNOW…………………………………………8 →	 <br>**Q970**<br><br>**Q970**
Q968	What does this phrase mean to you?<br><br>***Menene wannan kalmar ta ke nufi?***<br><br>[IF RESPONDENT ONLY GIVES THE COMMON DEFINITION OF THE PHRASE, PROBE TO SEE IF THERE IS ANY OTHER MEANING.<br><br>CIRCLE ALL MENTIONED	AGE AT MARRIAGE…………………………………….A<br>DELAYING FIRST BIRTH……………………………..B<br>USE/GO FOR FAMILY PLANNING..…………………..C<br>DELAYING AGE AT FIRST SEX……………………..D<br>ABSTINENCE……………………………………………..E<br>SPACING BETWEEN BIRTHS……………………….F<br>LIMITING FAMILY SIZE………………………………G<br>TALK TO HEALTH PROVIDER ABOUT FP…………H<br>TALK TO SPOUSE/PARTNER ABOUT FP…………..I<br>PLAN YOUR FAMILY …………………………………. J<br>GET IT TOGETHER……………………………………K<br><br>OTHERS (Specify)__________________________X<br>DON'T KNOW ………………………………………….Z	
Q969	Where did you see or hear this phrase?<br><br>***A ina ki ka ga ko ki ka ji wannan kalmar?***<br><br>CIRCLE ALL MENTIONED	RADIO DRAMA PROGRAM………………………………A<br>RADIO JINGLE/SPOT ……………………………………B<br>TELEVISION………………………………………………C<br>NEWSPAPER …………………………………………….D<br>POSTER ………………………………………………….E<br>UMBRELLA……………………………………………….F<br>SHOPPING BAG…………………………………………G<br>TSHIRT………. …………………………………………..H<br>LEAFLET/STICKERS…………………………………..I<br>BADGE/BUTTON……………………………………….J<br>CAPS………………………………………………………K<br>WRISTBAND……………………………………………L<br>ON A SIGN AT A HEALTH FACILITY……………….M<br>ON A SIGN AT A PHARMACY / CHEMIST…………N<br>AT A ROADSHOW / RALLY………………………….O<br>AT A PARADE………………………………………….P<br>BILLBOARD……………………………………………..Q<br>PENS……………………………………………………..R<br><br>OTHER (Specify)____________________________X<br>DON'T KNOW……………………………………………Z	

Q970	In the past year, have you heard the phrase "Ki la siri ewa re" – "ifeto somo bibi lasiri ewa mi" (meaning what is the secret of your beauty? Family planning is the secret of my beauty)<br><br>**A cikin shekara daya da ta shige, kinji wannan Kalmar "Ki la siri ewa re" – "ifeto somo bibi lasiri ewa mi"?**<br>***(Ma'ana-Menene sirrin kyawon ki? Tsarin iyali shine sirrin kyau na)***	YES………………………………………………………1<br><br>NO……………………………………………………….2 →<br><br>DON'T KNOW…………………………………………8 →	 <br>**Q973**<br><br>**Q973**

Q971	What does this phrase mean to you?<br><br>***Menene wannan kalmar ta ke nufi?***<br><br>[IF RESPONDENT ONLY GIVES THE COMMON DEFINITION OF THE PHRASE, PROBE TO SEE IF THERE IS ANY OTHER MEANING.<br><br>CIRCLE ALL MENTIONED	AGE AT MARRIAGE……………………………………A<br>DELAYING FIRST BIRTH……………………………..B<br>USE/GO FOR FAMILY PLANNING..…………………..C<br>DELAYING AGE AT FIRST SEX……………………...D<br>ABSTINENCE…………………………………………...E<br>SPACING BETWEEN BIRTHS………………………..F<br>LIMITING FAMILY SIZE………………………………G<br>TALK TO HEALTH PROVIDER ABOUT FP…………H<br>TALK TO SPOUSE/PARTNER ABOUT FP…………..I<br>PLAN YOUR FAMILY …………………………………. J<br>GET IT TOGETHER……………………………………K<br><br>OTHERS (Specify)__________________________X<br>DON'T KNOW ……………………………………….Z	
Q972	Where did you see or hear this phrase?<br><br>***A ina ki ka ga ko ji wannan kalmar?***<br><br>CIRCLE ALL MENTIONED	RADIO DRAMA PROGRAM..…………..………..…..A<br>RADIO JINGLE/SPOT …..………………………….B<br>TELEVISION……………………………………………C<br>NEWSPAPER …………………………………………..D<br>POSTER ……………………………………………….E<br>UMBRELLA……………………………………………..F<br>SHOPPING BAG………………………………………G<br>TSHIRT………. ………………………………………...H<br>LEAFLET/STICKERS…………………………………..I<br>BADGE/BUTTON………………………………………J<br>CAPS……………………………………………………K<br>WRISTBAND……………………………………………L<br>ON A SIGN AT A HEALTH FACILITY……………….M<br>ON A SIGN AT A PHARMACY / CHEMIST…………N<br>AT A ROADSHOW / RALLY…………………………..O<br>AT A PARADE…………………………………………P<br>BILLBOARD……………………………………………..Q<br>PENS……………………………………………………..R<br><br>OTHER (Specify)___________________________X<br>DON'T KNOW……………………………………………Z	
Q973	Do you understand Hausa language?<br><br>***Ki na jin Hausa?***	YES……………………………………………………1<br>NO……………………………………………………..2 →	**Q977**
Q974	In the past year, have you heard the phrase "Kun gane, tazaran haihuwa" (meaning Go for family planning)?<br>***A cikin shekara daya da ta shige,kin ta ba jin wa ta kalma "Ko kun gane, tazaran haihuwa".***	YES……………………………………………………1<br><br>NO……………………………………………………..2 →<br><br>DON'T KNOW……………………………………......8 →	**Q977**<br><br>**Q977**
Q975	What does this phrase mean to you?<br><br>***Me wannan kalmar ta ke nufi?***<br><br>[IF RESPONDENT ONLY GIVES THE COMMON DEFINITION OF THE PHRASE, PROBE TO SEE IF THERE IS ANY OTHER MEANING.<br><br>CIRCLE ALL MENTIONED	AGE AT MARRIAGE……………………………………A<br>DELAYING FIRST BIRTH……………………………..B<br>USE/GO FOR FAMILY PLANNING..…………………..C<br>DELAYING AGE AT FIRST SEX……………………...D<br>ABSTINENCE…………………………………………...E<br>SPACING BETWEEN BIRTHS………………………..F<br>LIMITING FAMILY SIZE………………………………G<br>TALK TO HEALTH PROVIDER ABOUT FP…………H<br>TALK TO SPOUSE/PARTNER ABOUT FP…………..I<br>PLAN YOUR FAMILY …………………………………. J<br>GET IT TOGETHER……………………………………K<br><br>OTHERS (Specify)__________________________X<br>DON'T KNOW ……………………………………….Z	

Q976	Where did you see or hear this phrase?<br><br>***A ina ki ka ga ko ki ka ji wannnan kalmar?***<br><br>CIRCLE ALL MENTIONED	RADIO DRAMA PROGRAM……………………………A<br>RADIO JINGLE/SPOT ………………………………B<br>TELEVISION……………………………………………C<br>NEWSPAPER ……………………………………………D<br>POSTER …………………………………………………E<br>UMBRELLA………………………………………………F<br>SHOPPING BAG………………………………………G<br>TSHIRT………. ………………………………………H<br>LEAFLET/STICKERS…………………………………I<br>BADGE/BUTTON………………………………………J<br>CAPS……………………………………………………K<br>WRISTBAND……………………………………………L<br>ON A SIGN AT A HEALTH FACILITY………………M<br>ON A SIGN AT A PHARMACY / CHEMIST…………N<br>AT A ROADSHOW / RALLY…………………………O<br>AT A PARADE…………………………………………P<br>BILLBOARD……………………………………………Q<br>PENS……………………………………………………R<br><br>OTHER (Specify)___________________________X<br>DON'T KNOW……………………………………………Z	
Q977	In the past year, have you seen or heard the word "NURHI"<br><br>***A cikin shekara daya da ta shige, kin ta ba gani ko jin wannan kalmar "NURHI"***	YES……………………………………………………1<br>NO………………………………………………………2<br>DON'T KNOW……………………………………………8	
Q978	Which of the following logos have you seen in the past year?<br><br>***Wacce alama ki ka gani a cikin shekara daya da ta wuce***?<br><br>**SHOW A CARD WITH THREE LOGOS.**	LOGO A/PUZZLE…………………………A<br>LOGO B…………………………………………B<br>LOGO C…………………………………………C<br>NONE……………………………………………Y →	**Q981**

Q979	Where did you see this logo?<br><br>***A ina ki ka ga wannan alamar?***<br><br>CIRCLE ALL MENTIONED	TELEVISION……………………………………………A<br>NEWSPAPER ……………………………………………B<br>POSTER …………………………………………………C<br>UMBRELLA………………………………………………D<br>SHOPPING BAG…………………………………………E<br>TSHIRT………. ………………………………………F<br>LEAFLET/STICKERS…………………………………G<br>BADGE/BUTTON………………………………………H<br>CAPS……………………………………………………I<br>WRISTBAND……………………………………………J<br>ON A SIGN AT A HEALTH FACILITY………………K<br>ON A SIGN AT A PHARMACY / CHEMIST…………L<br>AT A ROADSHOW / RALLY…………………………M<br>AT A PARADE…………………………………………N<br>BILLBOARD……………………………………………O<br>PENS……………………………………………………P<br><br>OTHER (Specify)___________________________X<br>DON'T KNOW……………………………………………Z	
Q980	What does this logo(s) mean to you?<br><br>***Menene wannan alamar ta ke nufi?***	AGE AT MARRIAGE……………………………………A<br>DELAYING FIRST BIRTH……………………………B<br>USE/GO FOR FAMILY PLANNING…………………C<br>DELAYING AGE AT FIRST SEX……………………D<br>ABSTINENCE……………………………………………E<br>SPACING BETWEEN BIRTHS………………………F<br>LIMITING FAMILY SIZE……………………………G<br>TALK TO HEALTH PROVIDER ABOUT FP…………H<br>TALK TO SPOUSE/PARTNER ABOUT FP…………I<br>PLAN YOUR FAMILY ………………………………… J<br>GET IT TOGETHER……………………………………K<br><br>OTHERS (Specify)_________________________X<br>DON'T KNOW ……………………………………Z	

Q981	In the past year, have you seen a card with the phrase "Be Successful" written on it and the picture of three men in front?<br><br>**A shekara daya data shige, kin ta ba ganin wani kati mai rubutu '' Be successful- ''Ki yi nasara'' da hoton maza 3 akan shi.**	YES……………………………………………………1<br>NO……………………………………………………..2<br>DON'T KNOW……………………………………………8	 → Q983<br>→ Q983
Q982	What were the key messages on the card?<br><br>**Menene muhimman sakonni da katin yake dauke da shi?**	AGE AT MARRIAGE………………………………………A<br>DELAYING FIRST BIRTH……………………………...B<br>USE/GO FOR FAMILY PLANNING...………………...C<br>DELAYING AGE AT FIRST SEX……………………...D<br>ABSTINENCE…………………………………………...E<br>SPACING BETWEEN BIRTHS………………………..F<br>LIMITING FAMILY SIZE………………………………G<br>TALK TO HEALTH PROVIDER ABOUT FP…………H<br>TALK TO SPOUSE/PARTNER ABOUT FP…………..I<br>PLAN YOUR FAMILY ………………………………… J<br>GET IT TOGETHER……………………………………K<br><br>OTHERS (Specify)_________________________X<br>DON'T KNOW ……………………………….….Z	
Q983	In the past year, have you seen a card with the phrase "Be Beautiful" written on it and pictures of three beautiful women in front?<br><br>**A shekara daya data shige, kin ta ba ganin wani kati mai rubutu "Be Beautiful"—""Ki kasance kyakkyawa'' da hotunan mata 3 kyawawa?**	YES……………………………………………………1<br>NO……………………………………………………..2<br>DON'T KNOW……………………………………………8	 → Q985<br>→ Q985
Q984	What were the key messages on the card?<br><br>**Menene muhimman sakonni da katin yake dauke da shi?**	AGE AT MARRIAGE………………………………………A<br>DELAYING FIRST BIRTH……………………………...B<br>USE/GO FOR FAMILY PLANNING...………………...C<br>DELAYING AGE AT FIRST SEX……………………...D<br>ABSTINENCE…………………………………………...E<br>SPACING BETWEEN BIRTHS………………………..F<br>LIMITING FAMILY SIZE………………………………G<br>TALK TO HEALTH PROVIDER ABOUT FP…………H<br>TALK TO SPOUSE/PARTNER ABOUT FP…………..I<br>PLAN YOUR FAMILY ………………………………… J<br>GET IT TOGETHER……………………………………K<br><br>OTHERS (Specify)_________________________X<br>DON'T KNOW ……………………………………..Z	
Q985	In the past year, have you seen any health provider wearing a badge/button that said "Ask me about FP?"<br>***A cikin shekarar da ta wuce,kin taba ganin wani jami'in kiwon lafiya da yake sanye da wata alama mai cewa "Tambaye ni gameda Tsarin Iyali"***	YES……………………………………………………1<br>NO……………………………………………………..2<br>DON'T KNOW……………………………………………8	
Q986	**In the** past three months**, have you accessed the internet, web, or email at least once through the following?**<br>***A cikin*** ***watanni 3 da suka shige****, kin ta ba bude yanar gizon internet, ko wasikar yanar gizo ko da sau daya?***		
	**a.** a mobile phone?<br>***Wayar salula***	YES……………………………………………………1<br>NO……………………………………………………..2<br>DONT KNOW INTERNET………………………………8	 <br>→ **Q989**
	b. A computer?<br>**Na'ura mai kwakwalwa**	YES……………………………………………………1<br>NO……………………………………………………..2	
Q987	In the past three months, have you accessed the internet, web, facebook or email at least once?<br><br>***A cikin watanni ukku da suka shige, kin ta ba bude yanar gizon internet, dandalin sada zumunci na facebook ko wasikar yanar gizo ko da sau daya?***	YES……………………………………………………1<br><br>NO……………………………………………………..2<br><br>DON'T KNOW……………………………………………8	 <br>→ **Q989**<br><br>→ **Q989**

Q988	Have you seen any family planning/child birth Spacing messages on the internet, web, facebook or email in the past three months?<br><br>***Kin ta ba ganin wani sako kan tsarin iyali/ tazarar haihuwa a yanar gizon internet ko dandalin sada zumunci na facebook ko wasikar yanar gizo a cikin watanni 3 da su ka shige?***	YES……………………………………………………1<br>NO………………………………………………….2<br>DON'T KNOW…………………………………………8	
Q989	Do you belong to any groups, clubs, or organizations?<br><br>***Ki na cikin wata kungiya, kulof –kulof ko kungiyoyi?***	YES……………………………………………………1<br><br>NO………………………………………………….2	→ **Q991**
Q990	Have you heard or seen any family planning/child birth spacing information at these meetings?<br><br>***Kin ta ba ji ko ganin wani abu akan tsarin iyali/ tazarar haihuwa a wajen wadannan taron?***	YES……………………………………………………1<br>NO………………………………………………….2<br>DON'T KNOW…………………………………………8	
Q991	In the past year, have you heard any information about family planning/child birth spacing at any of the following events [READ LIST]:<br><br>Naming Ceremonies<br>Freedom ceremonies from an apprenticeship<br>School graduation ceremonies<br>Christmas/Eid celebration<br>Wedding<br><br>**A cikin shekarar da ta shige, kin ta ba jin wata magana akan tsarin iyali/ tazarar haihuwa a daya daga cikin wadannan taron, Bikin kammala koyon sana'a, kammala karatu, bukukuwan kirismeti ko salla.**<br>**Bikin kammala makaranta ko, Bikin aure**	NAMING CEREMONY……………………………A<br>FREEDOM CEREMONY…………………………B<br>GRADUATION…………………………………C<br>CHRISTMAS/EID………………………………D<br>WEDDING……………………………………E<br>NONE…………………………………………Y	

SECTION 10: MIGRATION HISTORY			
**Qno**	**Questions and filters**	**Coding categories**	**Skip to**
Now I would like to ask you about how often you visit other areas. Specifically, if you have visited an urban area or city/town and if you have visited a rural area or a village.<br><br>***Yanzu ina so na san yadda kike ziyartar wurare, musamman idan kin ziyarci wani gari ko birni/babban gari ko kuma idan kin ziyarci wani kauye ko karkara?***			
Q1001	Where were you born? In this city, another city, a town or a rural area/village?<br><br>***A ina aka haife ki? A wannan birnin, wani birnin, gari ko karkara/kauye?***	THIS CITY……………………………………………………………...1<br>ANOTHER CITY………………………………………………………2<br>A TOWN………………………………………………………………..3<br>A RURAL AREA OR VILLAGE………………………………………4<br>ABROAD………………………………………………………………..5	
Q1002	How long have you been living continuously in this city?<br><br>***Tun yaushe ki ke zaune a wannan birnin?***<br><br>IF A FEW WEEKS TO 11 MONTHS, RECORD 00-11. OTHERWISE, RECORD NUMBER OF YEARS.	NUMBER OF MONTHS……………………1 [__|__]<br><br>NUMBER OF YEARS………………………2 [__|__]<br><br>HAVE ALWAYS LIVED IN THIS CITY………………………......995 →	**Q1004**
Q1003	Just before you moved here, did you live in ABUJA, KADUNA, ZARIA, BENIN, IBADAN, ILORIN OR ELSEWHERE?<br><br>**IF THE RESPONDENT MENTION ELSEWHERE, ASK IF THE PLACE IS RURAL AREA OR TOWN**<br>***Kafin ki dawo wannan wurin, kin ta ba zama a ABUJA, KADUNA, ZARIA.BENIN IBADAN, ILORIN ko wani garin?***	ABUJA …………………………………………….. …………….. .. 01<br>BENIN ……………………………...................... . ……………… 02<br>IBADAN………………………………………………………………… 03<br>ILORIN……………………………………………….. ……………… 04<br>KADUNA ……………………………….. ………… ……………….. 05<br>ZARIA ………………………….......................... ………………... 06<br><br>ANOTHER CITY ……………………………………………………… 07<br>ANOTHER TOWN …………………………………………………… 08<br>RURAL AREA/VILLAGE……………………………………………… 09<br>ANOTHER COUNTRY……………………………………………….. 10	
Q1004	Since JANUARY 2010, have you moved residences/houses?<br><br>***Tun daga January 2010, kin taba canja wurin zama ko gida?***	YES . . . . …………………………1<br><br>NO . . . . ……………………………..2	
Q1005	Since you started living here, have you stayed away from this city for 6 (six) months or longer?<br><br>**Tun lokacin da ki ka fara zama anan, Kin ta ba barin wannan wurin tsawon wata 6 ko fiye da haka?**	YES . . . . ………………………..1<br><br>NO . . . . ……………………….2	
Q1006	Which **<u>city or town</u>** did you visit **<u>most often in the past 12</u>** months?<br><br>WRITE NAME OF THE CITY, LGA AND STATE<br><br>***Wanne <u>birni ko gari</u> <u>ki ka fi yawan kai ziyara a watani 12</u> da suka shige?***	SHE HAS TRAVELED IN THE PAST YEAR………………………1<br><br>NAME OF TOWN/CITY ________________________________<br><br>LGA NAME:____<br><br>*CODE BOXES: OFFICE ONLY* [__|__|__]<br><br>STATE NAME:____<br><br>*CODE BOXES: OFFICE ONLY*[__|__|__]<br><br>SHE HAS NOT TRAVELED IN THE PAST YEAR………………2 →	**Q1011**

Q1007	How often did you visit this city/town [NAME OF CITY/TOWN Q1006] in the **last 12 months**?<br><br>**Kamar sau nawa kike ziyartar wannan birni/gari [NAME OF CITY/TOWN Q1006] a cikin watanni goma sha biyu da suka shige?**	PER WEEK……………………..…..…1 [__|__]<br><br>PER MONTH…………………………2 [__|__]<br><br>PER YEAR……………………………3 [__|__]	
Q1008	How long do you usually stay when you visit [NAME OF CITY/TOWN]?<br><br>**Tsawon wane lokaci ki kan zauna idan ki ka kai ziyarar [NAME OF CITY/TOWN]?**	DAYS…………………………………..1 [__|__]<br><br>WEEKS ………………………………..2 [__|__]<br><br>MONTHS ………………..…………...3 [__|__]	
Q1009	During your visit(s), do you ever discuss family planning/child birth spacing/child spacing with anybody?<br><br>***A lokacin ziyarar,shin ko kin taba neman dabarar tsarin iyali/tazara tsakanin haihuwa/tazara tsakanin yara daga wani mutum?***	YES………………………………………………………1<br><br>NO……………………………………………………..2	
Q1010	During your visit(s), do you ever seek family planning spacing services?<br><br>***A lokacin ziyarar, shin ko kin taba nemi hanyoyin tsarin iyali/tazara tsakanin haihuwa/tazara tsakanin yara?***	YES……………………………………………….……1<br><br>NO…………………………………………………..…..2	
Q1011	Do friends or family from ANOTHER CITY OR TOWN IN NIGERIA ever come to visit you?<br><br>**Akwai kawaye ko 'yanuwa DAKE ZAMA A WANI BIRNI KO GARI A CIKIN NIGERIA sun ta ba kawo maki ziyara?**	YES…………………………………….…..….…..………1<br><br>NO……………………………………..………….……..2 →	**Q1014**
Q1012	Do you ever talk about family planning with your friends and family who are visiting you here?<br><br>**Shin *ko kin taba magana akan tsarin iyali da kawaye da iyalin ki idan su ka ziyarce ki anan*?**	YES……………………………………………….……1<br><br>NO……………………………………………………...2	
Q1013	Do your friends and family who visit ever seek family planning services when they are visiting?<br><br>***Shin kokawaye ko 'yan uwanki sun ta ba neman hanyar kaiyade tsarin iyali lokacin da suka kawo ziyarar?***	YES…………………………………………………………1<br><br>NO……………………………….……………….……..2<br><br>DON'T KNOW……………………………….…………8	
Q1014	In the past **12 months**, have you visited a rural area in Nigeria in order to visit family or friends?<br><br>***A wata 12 da suka shige, kin taba ziyartan wani karkara (kauye) a Nijeriya da niyar ziyartan 'yan'uwa ko kawaye?***	YES. . . . .……….………….1<br><br>NO . . . .………………..……2 →	**End of QRE**

Q1015	Which rural area did you visit **most often in the past 12** months?<br><br>***Wacce karkarar (kauye) ki ke yawan kai ziyara a watani 12 da suka shige?***<br><br>*GIVE THE NAME OF THE VILLAGE, LGA AND STATE*	NAME OF VILLAGE________________________<br><br>LOCAL GOVERNMENT AREA:___________________________________<br><br>*CODE BOXES: OFFICE ONLY* [__|__|__]<br><br>STATE____________________________<br><br>*CODE BOXES: OFFICE ONLY* [__|__|__]	
Q1016	How often did you visit this rural area [NAME OF PLACE IN Q1015 in the **last 12 months**?<br><br>Sau nawa ki ka ziyarci wanan kauye a watanin 12 da suka shige?**[NAME OF PLACE IN Q 1015)**	PER WEEK……………………..…..1 [__|__]<br>PER MONTH…………………………2 [__|__]<br>PER YEAR……………………………3 [__|__]	
Q1017	How long do you usually stay when you visit [NAME OF PLACE)?<br><br>**Tsawon wane lokaci ki ke zama idan kin kai ziyarar [NAME OF PLACE)?**	DAYS…………………………………..1 [__|__]<br><br>WEEKS ……………………………… 2 [__|__]<br><br>MONTHS ………………..………...3 [__|__]	
Q1018	During your visit(s), do you ever discuss family planning/child birth spacing/child spacing with anybody?<br><br>***Lokacin da ki ka kai ziyarar, shin ko kin ta ba tattaunawa akan tsarin iyali/tazara tsakanin haihuwa/tazara tsakanin yara da wata/wanni?***	YES…………………………………………………….1<br><br>NO……………………………………………….…..2	
Q1019	During your visit(s), do you ever seek family planning spacing services?<br><br>***Lokacin ziyarar, shin ko kin taba neman hanyoyin tsarin iyali/tazara tsakanin haihuwa/tazara tsakanin yara?***	YES……………………………………………….…1<br><br>NO……………………………………………………...2	
Q1020	Do friends or family from this rural area [MENTIONED IN Q1015] ever come to visit you?<br><br>**Shin kawaye ko iyali daga wanan wurin [MENTIONED IN Q 1015] sun taba ziyartan ki?**	YES……………………………………………….……1<br><br>NO……………………………………………………..2 →	**End of QRE**
Q1021	Do you ever talk about family planning with your friends and family who are visiting you here?<br><br>**Shin *ko kin taba magana akan tsarin iyali da kawaye da iyalin ki idan su ka ziyarce ki anan?***	YES……………………………………………………1<br><br>NO……………………………………………………..2	
Q1022	Do your friends and family who visit ever seek family planning services when they are visiting?<br><br>***Shin ko kawayen ki ko iyalan ki sun taba neman hanyoyin tsaran iyali yayin da suka ziyarce ki?***	YES……………………………………………………1<br>NO……………………………………………………..2<br>DON'T KNOW………………………………...………8	

INSTRUCTIONS:
ONLY ONE CODE SHOULD APPEAR IN ANY BOX

ALL MONTHS SHOULD BE FILLED IN COL 1 AND COL 3

INFORMATION TO BE CODED FOR EACH COLUMN

COL 1 BIRTHS, PREGNANCIES, CONTRACEPTIVE USE

B BIRTHS
P PREGNANCIES
A ABORTION
M MISCARRIAGE
S STILL BIRTH

0 NO METHOD
1 FEMALE STERILIZATION
2 MALE STERILIZATION
3 IMPLANT
4 IUD
5 INJECTABLES
6 DAILY PILL
7 EMERGENCY PILL
8 MALE CONDOM
9 FEMALE CONDOM
H STANDARD DAYS METHOD
L LAM
X OTHER MODERN METHOD (SPECIFY________)
R RHYTHM METHOD
W WITHDRAWAL
Y OTHER TRADITIONAL METHODS

COL 2 DISCONTINUATION OF CONTRACEPTIVE USE

0 INFREQUENT SEX/HUSBAND AWAY
1 METHOD FAILED/BECAME PREGNANT WHILE USING
2 WANTED TO BECOME PREGNANT
3 HUSBAND/PARTNER DISAPPROVED
4 WANTED MORE EFFECTIVE METHOD
5 FEAR OF SIDE EFFECTS/HEALTH CONCERNS
6 LACK OF ACCESS/TOO FAR
7 COSTS TOO MUCH
8 INCONVENIENT TO USE
9 FATALISTIC/UP TO GOD
F DIFFICULT TO GET PREGNANT/MENOPAUSAL
A MARITAL DISSOLUTION/SEPARATION
D LACK OF SEXUAL SATISFACTION
L CREATED MENSTRUAL PROBLEM
M GAINED WEIGHT
G DID NOT LIKE METHOD
N LACK OF PRIVACY FOR USE
X OTHER ___________________________
(SPECIFY)
Z DON'T KNOW

COL 3 MARRIAGE

X MARRIED, REMARRIED OR LIVING WITH A MAN
O NOT MARRIED/WIDOWED/SEPARATED/DIVORCED

				COL1	COL2	COL3			
	12	DEC	1				1	DEC	
	11	NOV	2				2	NOV	
	10	OCT	3				3	OCT	
	9	SEP	4				4	SEP	
2	8	AUG	5				5	AUG	2
0	7	JUL	6				6	JUL	0
1	6	JUN	7				7	JUN	1
4	5	MAY	8				8	MAY	4
	4	APR	9				9	APR	
	3	MAR	10				10	MAR	
	2	FEB	11				11	FEB	
	1	JAN	12				12	JAN	
	12	DEC	13				13	DEC	
	11	NOV	14				14	NOV	
	10	OCT	15				15	OCT	
	9	SEP	16				16	SEP	
2	8	AUG	17				17	AUG	2
0	7	JUL	18				18	JUL	0
1	6	JUN	19				19	JUN	1
3	5	MAY	20				20	MAY	3
	4	APR	21				21	APR	
	3	MAR	22				22	MAR	
	2	FEB	23				23	FEB	
	1	JAN	24				24	JAN	
	12	DEC	25				25	DEC	
	11	NOV	26				26	NOV	
	10	OCT	27				27	OCT	
	9	SEP	28				28	SEP	
2	8	AUG	29				29	AUG	2
0	7	JUL	30				30	JUL	0
1	6	JUN	31				31	JUN	1
2	5	MAY	32				32	MAY	2
	4	APR	33				33	APR	
	3	MAR	34				34	MAR	
	2	FEB	35				35	FEB	
	1	JAN	36				36	JAN	

INSTRUCTIONS:
ONLY ONE CODE SHOULD APPEAR IN ANY BOX

ALL MONTHS SHOULD BE FILLED IN COL 1 AND COL 3

INFORMATION TO BE CODED FOR EACH COLUMN

COL 1 <u>BIRTHS, PREGNANCIES, CONTRACEPTIVE USE</u>

- B BIRTHS
- P PREGNANCIES
- A ABORTION
- M MISCARRIAGE
- S STILL BIRTH

- 0 NO METHOD
- 1 FEMALE STERILIZATION
- 2 MALE STERILIZATION
- 3 IMPLANT
- 4 IUD
- 5 INJECTABLES
- 6 DAILY PILL
- 7 EMERGENCY PILL
- 8 MALE CONDOM
- 9 FEMALE CONDOM
- H STANDARD DAYS METHOD
- L LAM
- X OTHER MODERN METHOD (SPECIFY________)
- R RHYTHM METHOD
- W WITHDRAWAL
- Y OTHER TRADITIONAL METHODS

COL 2 <u>DISCONTINUATION OF CONTRACEPTIVE USE</u>

- 0 INFREQUENT SEX/HUSBAND AWAY
- 1 METHOD FAILED/BECAME PREGNANT WHILE USING
- 2 WANTED TO BECOME PREGNANT
- 3 HUSBAND/PARTNER DISAPPROVED
- 4 WANTED MORE EFFECTIVE METHOD
- 5 FEAR OF SIDE EFFECTS/HEALTH CONCERNS
- 6 LACK OF ACCESS/TOO FAR
- 7 COSTS TOO MUCH
- 8 INCONVENIENT TO USE
- 9 FATALISTIC/UP TO GOD
- F DIFFICULT TO GET PREGNANT/MENOPAUSAL
- A MARITAL DISSOLUTION/SEPARATION
- D LACK OF SEXUAL SATISFACTION
- L CREATED MENSTRUAL PROBLEM
- M GAINED WEIGHT
- G DID NOT LIKE METHOD
- N LACK OF PRIVACY FOR USE
- X OTHER ____________________________ (SPECIFY)
- Z DON'T KNOW

COL 3 <u>MARRIAGE</u>

- X MARRIED, REMARRIED OR LIVING WITH A MAN
- O NOT MARRIED/WIDOWED/ SEPARATED/DIVORCED

				COL1	COL2	COL3			
	12	DEC	37				37	DEC	
	11	NOV	38				38	NOV	
	10	OCT	39				39	OCT	
	9	SEP	40				40	SEP	
2	8	AUG	41				41	AUG	2
0	7	JUL	42				42	JUL	0
1	6	JUN	43				43	JUN	1
1	5	MAY	44				44	MAY	1
	4	APR	45				45	APR	
	3	MAR	46				46	MAR	
	2	FEB	47				47	FEB	
	1	JAN	48				48	JAN	
	12	DEC	49				49	DEC	
	11	NOV	50				50	NOV	
	10	OCT	51				51	OCT	
	9	SEP	52				52	SEP	
2	8	AUG	53				53	AUG	2
0	7	JUL	54				54	JUL	0
1	6	JUN	55				55	JUN	1
0	5	MAY	56				56	MAY	0
	4	APR	57				57	APR	
	3	MAR	58				58	MAR	
	2	FEB	59				59	FEB	
	1	JAN	60				60	JAN	
	12	DEC	61				61	DEC	
	11	NOV	62				62	NOV	
	10	OCT	63				63	OCT	
	9	SEP	64				64	SEP	
2	8	AUG	65				65	AUG	2
0	7	JUL	66				66	JUL	0
0	6	JUN	67				67	JUN	0
9	5	MAY	68				68	MAY	9
	4	APR	69				69	APR	
	3	MAR	70				70	MAR	
	2	FEB	71				71	FEB	
	1	JAN	72				72	JAN	

OFFICE USE ONLY: QUESTIONNAIRE NO: [__|__|__|__|__|__|__|__|__|__]
(cluster code 4 digits + HH number 3 digits + household division number + Respondent line number 2 digits)

RECORD END TIME.	HOUR……………………………….[__|__]<br>MINUTES…………………………..[__|__]

COMMENTS:

END THE INTERVIEW AND THANK THE RESPONDENT FOR HER TIME AND PARTICIPATION.
