# Supplementary material for: On the sustainability of a family planning program in Nigeria when funding ends
Source: PLoS One. 2019 Sep 26;14(9):e0222790. doi: 10.1371/journal.pone.0222790 (PMC6762171; doi:10.1371/journal.pone.0222790)
Supplement: S2 File — (PDF) [file pone.0222790.s005.pdf]

OFFICE USE ONLY: QUESTIONNAIRE NO: [__|__|__|__|__|__|__|__|__|__|__]
(City/state code 2 digit + Cluster code 4 digits + HH number 3 digits + Respondent line number 2 digits)

MLE SUSTAINABILITY STUDY
NIGERIA
WOMAN QUESTIONNAIRE 2017: HAUSA

STATE NAME & CODE ____________________

CITY/STATE NAME & CODE____________________

(Ilorin=4, Kaduna=5, Jos=9, Plateau State=10)

LGA NAME & CODE ____________________

LOCALITY NAME & CODE ____________________

CLUSTER NAME & CODE ____________________

STRUCTURE NUMBER ____________________

HOUSEHOLD NUMBER ____________________

NAME & LINE NUMBER OF WOMAN ____________________

[SURNAME, GIVEN NAME]

NAME OF HOUSEHOLD HEAD ____________________

ADDRESS OF HOUSEHOLD

____

____

INTERVIEWER VISITS

**VISIT No.**	**1**	**2**	**3**	**FINAL VISIT**
DATE	DAY/ MONTH/ YEAR [____/_____/_17_]	DAY/ MONTH/ YEAR [____/_____/__17_]	DAY/ MONTH/ YEAR [____/_____/__17_]	DAY [____|____] MONTH [____|____] YEAR [_2_|_0_|_1_|_7_]
INTERVIEWER'S NAME	____________	____________	____________	____________
INTERVIEWER CODE	[ ][ ][ ]	[ ][ ][ ]	[ ][ ][ ]	[ ][ ][ ]
RESULT*	[ ]	[ ]	[ ]	[ ]
**NEXT VISIT: DATE**	[____/_____/17_]	[____/_____/17_]		TOTAL NO. OF VISITS [ ]
**TIME**	[ ][ ] [ ][ ] H H M M	[ ][ ] [ ][ ] H H M M		

***RESULT CODES:**
1. COMPLETED
2. NOT AT HOME
3. POSTPONED
4. REFUSED
5. PARTLY COMPLETED
6. INCAPACITATED
7. OTHER ____________________(Specify)

LANGUAGE

MAIN LANGUAGE OF INTERVIEW TRANSLATOR USED? YES = 1 NO = 2
LANGUAGE CODES: HAUSA =1 YORUBA =2 ENGLISH = 3 PIDGIN =4 OTHERS = 6 (Specify________________)

**SUPERVISOR**	**FIELD EDITOR**	**OFFICE EDITOR**	**KEYED BY**
NAME………………………………	NAME………………………..	NAME…………………………………….	NAME……………………………………………………………
CODE: [ ][ ][ ]	CODE: [ ][ ][ ]	CODE: [ ][ ]	CODE: [ ][ ]
DATE [__ / ___/ 17_] DD MM YY	DATE [____ / ____/ 17_] DD MM YY	DATE [____ / ____/ 17_] DD MM YY	DATE [_____/ _____/ 17_] DD MM YY

RECORD START TIME: HOUR…………………………[__|__] MINUTES………………….[__|__]

Individual Consent: Woman's Survey on Family Planning
Yoruba

Introduction

Hello! My name is _________________________________; I am part of a research team working for the Data Research and Mapping Consult, LTD. (DRMC)under the Measurement, Evaluation (MLE) Project for the Nigerian Urban Reproductive Health Initiative (NURHI). We are carrying out research on child birth spacing in urban areas of Nigeria. Your participation in this study will help to improve family planning services in this city. We will be asking questions to all women 15 – 49 years from this household for this study.

Mo ki yin! ***Oruko mi ni________________________________, Mo je okan ninu awon oluwadi ti o nse ise fun ile ise*****Data Research and Mapping Consult, LTD. (DRMC)** ***labe akoso akanse ise*** **Measurement, Learning & Evaluation (MLE)fun eto alaafia abiye ni awon ilu nla ni orile ede Naijiria (NURHI).** ***A n se agbeyewo/iwadi lori bi a se nfi alafo si aarin omo kan ti a bi si ekeji ni agbeegbe ilu nla ti Naijiriya*****). Ikopayin** ***ninu iwadi yi yio se iranwo lati mu ifeto-somo-bibi dara si ni ilu nla yi. A o maa beere ibeere lowo gbogbo obinrin laarin odun meedogun si odun mokandinlaadota (15-49 years) lati inu idile yi fun iwadi yi.***

Explanation of Procedure

The interview will take place in or around your home, somewhere private. The interview will take about 60 minutes. I will ask you questions about your home, child birth spacing, health-care seeking, and family size decisions. You may choose not to give the interview, or not to answer a question for any reason. You can stop the interview at any time by telling me that you want to stop it. If you decide not to give the interview or not to answer a question, no harm will come to you, and there will be no effect on your access to health services in the future.

Iforowanilenuwo naa yo waye ninu tabi ni agbegbe ile yin, nibi ikoko. Iforowanilenuwo naa yoo gba to ogota iseju. Mo maa beere awon ibeere nipa ile yin, ifaye sile laarin ibimo kan si ikeji, wiwa itoju ilera, ati ipinnu nipa bi ebi se ni lati tobi to. E le yan lati ma se iforowanilenuwo naa, tabi lati mase dahun ibeere kan fun idi kankan. E le da iforowanilenuwo naa duro nigbakugba nipa siso fun mi wipe e fe da'nu duro. Ti e ba yan lati mase iforowanilenuwo naa tabi lati mase dahun ibeere kan, ko si ijiya fun yin, ati pe ko si ipa ti o ni lori anfaani ti idile yin ni si awon eto ilera l'ojo iwaju.

Confidentiality

Your answers will not be shared with anyone outside this research project. Your name will not appear on the survey. We will not share answers with community members, health providers, family or anyone else. At the end of the study, we will put all the answers together and make a report.

Once the study is finished, the completed surveys will be destroyed.

Awon idahun yin ni a ko ni se alabapin pelu eni kankan ti ko si ninu iwadi yi. Oruko yin ko ni jeyo ninu iwadi naa. A ko ni se alabapin awon idahun (oro) yin pelu awon ara agbegbe yi, awon olupese (osise) ilera, ebi tabi enikankan. Ni ipari Iwadi naa, a o ko gbogbo idahun (oro) jo a o si ko abajade re.

Ni kete ti a ba pari iwadi yi, gbogbo eto iwadi naa ni a o baje.

Benefits

Research helps society by providing new knowledge. You may not benefit directly from this survey. However, your answers will be important for planning better programs to make sure women can access the health care they need.

Iwadi maa n ran awujo lowo lati pese imo tuntun. Eyin funra yin lee ma ni anfaani ni pato latari pe e kopa ninu iwadi yi. Sibe-sibe, awon idahun yin si iwadi wa yi yio ran wa lowo lati se agbekale awon eto ti o le mu ki awon obinrin ri eto ilera ti won nilo gba.

Risks and Discomfort

There is the possibility you may feel uncomfortable about a question I ask. If you feel uncomfortable about any of the questions, you do not have to answer them. I can skip those questions and go on to the next section. You can end the interview at any time. There is also the possibility that someone may approach us during the interview to find out what we are discussing. We intend to do this interview in private, if someone approaches us, we will stop the interview until we can continue in private.

Some questions may not apply to you, but the interviewer must ask the same questions of everyone.

O seese ki e lora nipa ibeere ti mo fe beere lowo yin. Ti ibeere kankan ko ba ba yin lara mu, ko je dandan wipe ki e dahun won. Mo lee fo awon ibeere na ki a si lo si awon ti o tele. E le mu opin wa si iforo-wanil'enuwo yi ni igbakugba. O seese ki enikan wole l'akoko ti a n soro lati mo ohun ti a n so. Ero wa ni lati se iforo-wanil'enuwo yi ni bonkele, bi enikeni ba wa sodo wa, a o da iforo-wanil'enuwo naa duro titi di igba ti a o te siwaju laisi eti keta.

Awon ibeere kan lee ma ni se pelu yin, sugbon oluwadi ni lati beere awon ibeere kan naa lowo olukuluku.

Costs and PaymentIndividual Consent:

There are no costs for being in this study. You will not receive any compensation for taking part in this study.

Ko si ohunkohun ti e nilati fi sile fun kikopa ninu iwadi yi. A ko si ni fun yin ni ohun gba-mabinu kankan fun ikopa yin ninu iwadi yi.

Questions /Your rights as Participants

This study has been approved by the National Health Research Ethics Committee of Nigeria (NHREC(assigned no . NHREC/01/01/2007, approved duration from 03/05/2017 to 02/05/2018) and the University of North Carolina (USA) (#17-1215). If you have any questions about this study or the results, you can contact the following: The study coordinator, Mr. Fasiku Adekunle David at the Data Research and Mapping Consult, LTD. at 08023635726 or the Institutional Review Board at the University of North Carolina at +1 919-966-3113. You have the right to ask, and have answers, to any questions you may have about this research. If you have questions or concerns, you should contact the researchers listed above, or ask me before or after the interview. Do you have any questions now?

Iwadi yi ti gba ase National Health Research Ethics Committee ti Naijiria (NHREC) (*NHREC|01|)01|2007 fun Akoko lati 03|05|2017 si 02|05|2018 *ati ile eko nla ni ilu Amerika (the University of North Carolina (USA) (#17-1215)*. Ti e ba ni ibeere kankan nipa iwadi yi tabi abajade re, e le kan si awon wonyi: Oludari iwadi naa, Ogbeni Fasiku Adekunle David ni ile ise Data Research and Mapping Consult Ltd lori ero ibanisoro 08023635726 tabi *Ajo awon Alayewo ti University of North Carolina* lori ero ibanisoro *+1 919-966-3113. E ni eto lati beere ohunkohun nipa iwadi yi, ki e si gba idahun. Ti e ba ni ibeere tabi ifiyesi, ki e kan si awon oruko ti a ko siwaju tabi ki e beere lowo mi ki a to beere tabi ti a ba pari iwadi yi. Se e ni ibeere kankan bayi?

Consent

Now, can you tell me if you agree to participate in this research? If you say yes, it means that you have agreed to be part of the study.

Ni bayi, nje e le so fun mi ti e ba faramo lati kopa ninu iwadi yi? Ti e ba so pe beeni, o tumo si pe e ti gba lati kopa ninu iwadi yi.

May I begin the interview now?
Se mo le bere iforowanilenuwo naa bayi?

Yes ………………………1 ⟶ **CONTINUE**

No …………………………2 ⟶ **THANK RESPONENT AND END INTERVIEW**

Would you like a copy of this document?

Nje e nife si eda iwe yii.

Interviewer's signature... **DATE [____/_____/2017]**

SECTION 1: BACKGROUND CHARACTERISTICS

Qno	Questions and filters	Coding categories	Skip to
	Thank you for agreeing to participate in this survey. As I mentioned in asking for your consent, we are looking to assess your health and information needs. To begin, we are going to ask you some background questions about yourself.<br><br>***Nagode da amincewar ki a wannan binciken. Kamar yadda na fada miki wajen neman izinin ki, muna neman bayanai akan lafiyar ki. Da farko, zamu tambaye ki wasu mahimman abubuwa game da ke kanki.***		
Q101	In what month and year were you born?<br><br>***A wanne wata da shekara aka haife ki?***	MONTH . . . .[__|__]<br>DON'T KNOW MONTH . . . . 98<br>YEAR . . . .[__|__][__|__]<br>DON'T KNOW YEAR 9998	
Q102	How old were you on your last birthday?<br><br>***Shekarunki nawa cikakku?***<br><br>COMPARE AND CORRECT Q101 AND/OR Q102 IF INCONSISTENT	AGE IN COMPLETED YEARS……..[__|__]	
Q103	CHECK 101 & 102:<br><br>IF AGE (15-49)YRS ☐ ↓ ELSE ☐ →<br><br>CHECK THAT CONSENT FORM HAS BEEN COMPLETED<br><br>IF NOT, ENSURE THAT YOU OBTAIN CONSENT FROM THE RESPONDENT BEFORE YOU CONTINUE		**END INTER-VIEW**
Q103a	How long have you been living continuously in this house?<br><br>**Tun yaushe kike zama a wannan gidan?**<br><br>IF A FEW WEEKS TO 11 MONTHS, RECORD 00-11. OTHERWISE, RECORD NUMBER OF YEARS.	MONTHS………………………… 1 [__|__]<br><br>YEARS……. ………………………2 [__|__]<br><br>ALWAYS………………………….. 995<br>VISITOR ……………………..…. 996	
Q103b	Have you ever been interviewed before for this study?<br><br>PROBE: Has anyone ever read you that consent form before?<br><br>**A taba yi maki tambayoyi a wannan binciken?**<br><br>**PROBE; Ko wata ta taba karanta maki takardar daukar izini kafin amsa tambayoyi?**	YES………………………………….....................1<br>NO …………………………………………………....2	
Q104	Have you ever attended school, formal or Qur'anic?<br><br>***Kin ta ba shiga makarantar boko ko ta Allo (Kur'ani)?***	YES………………………………….....................1<br>NO …………………………………………………....2 →	**Q108**
Q105	What is the highest level of school you **attended:** Quranic only, primary, junior secondary, senior secondary, or higher?<br><br>***Mene ne zurfin ilmin ki: Makarantar Allo (Al kur'ani) kadai, firamare, karamar sakandare, babbar sakandare ko gaba da sakandare?***	QURANIC ONLY……………………………...............0 →<br>PRIMARY………………………….………………....1<br>JUNIOR SECONDARY (JSS)……...................... ...2<br>SENIOR SECONDARY (SSS)……..........................3<br>HIGHER…………………………..….....................4 →	**Q108**<br><br><br><br>**Q110**
Q106	What is the highest (class/year) you completed at that level?<br><br>***Aji nawa ne mafi zurfi da ki ka kammala a wannan matakin?***<br><br>IF NO YEAR COMPLETED, WRITE "00"	CLASS/YEAR……………………[__|__]	
Q107	CHECK 105<br>PRIMARY (CODE 1) ↓ ☐ JSS OR SSS (CODE 2 OR 3) ☐ →		**Q110**
Q108	Now I would like you to read a sentence to me, but please tell me, what language you are most comfortable with?<br><br>**Yanzu ina son ki karanta mani wannan shadara , amma kafin ki karanta mani wanne harshe kika fi kwarancewa da shi?**<br><br>CIRCLE CODE FOR LANGUAGE SELECTED AND HAND THE RESPONDENT A LITERACY CARD IN THE SELECTED LANGUAGE	HAUSA……………………………….……….. …01<br>YORUBA……………………….................. …02<br>IGBO……………………………………………03<br>ENGLISH…………………………………………04<br>PIDGIN ENGLISH………………………………05<br>RESPONDENT CANNOT<br>READ………………………………………..... 11 →<br>BLIND/VISUALLY IMPAIRED……………….. ..21 →<br>OTHER__________________________ 96<br>(SPECIFY)	 <br><br><br><br><br>**Q110**<br>**Q110**

Q109	Now I would like you to read this sentence to me.<br><br>**Yanzu ina son ki karanta mani wannan shadara?**<br><br>SHOW A SENTENCE FROM THE LITERACY CARD TO THE RESPONDENT<br>IF RESPONDENT CANNOT READ THE WHOLE SENTENCE, PROBE:<br>Can you read any part of the sentence to me?<br>Kina iya karanta mani wani wuri daga wannan shadarar?	CANNOT READ AT ALL……………………….. 1<br>ABLE TO READ ONLY PARTS OF SENTENCE . . . .…………... 2<br>ABLE TO READ WHOLE SENTENCE……………………..…….….…… 3<br>NO CARD WITH REQUIRED LANGUAGE……………………………………… 4	
Q110	What is your religion?<br><br>***Menene addinin ki?***	CATHOLIC………………………….………. 1<br>PROTESTANT/OTHER CHRISTIAN……………………..……………. 2<br>MUSLIM…………………………...……….….. 3<br>NO RELIGION …………………..………………. 5<br>OTHER______________________________ 6<br>(SPECIFY)	**Q113**
Q111	How religious do you consider yourself? Do you consider yourself …<br><br>READ OUT RESPONSES<br><br>***Yaya ki ka dauki kanki a addinance? Kin dauki kan ki mai yin addini sosai, mai yin addini daidai gwargwado ko ba mai yin addini ba…..***	STRONGLY RELIGIOUS ……….…………… 1<br>SOMEWHAT RELIGIOUS ……….………….. 2<br>NOT AT ALL RELIGIOUS…….……………. 3	
Q112	To what degree does your religion influence the decisions you make about family planning? Would you say that your beliefs: never, somewhat, often, or always influence the decisions you make about family planning?<br><br>***A wanne matsayi ne addinin ki yake shafar kudurin da kike yi game da tsarin iyali? Zaki iya cewa addininki baya shafar kudurin da ki ke yi game da tsarin iyali,yana shafar kudurin ki ko da yaushe/ ko sa' i -sa' i .***	NEVER……………………………..…1<br>SOMEWHAT……...…………….….…2<br>OFTEN/FREQUENTLY…………..….3<br>ALWAYS…………………………..….4<br>DON'T KNOW (ABOUT FP)……..….8	
Q113	What is your ethnic group?<br><br>***Menene kabilar ki?***	________________________________<br>CODE BOX OFFICE USE ONLY [ ][ ][ ]	
Q114	What language do you usually speak at home?<br><br>***Wanne harshe/yarei ki ka fi amfani dashi a gida?***	HAUSA………………………………1<br>YORUBA……………………………2<br>IGBO…………………………………3<br>ENGLISH………………………….. 4<br>PIDGIN ENGLISH………………….5<br>OTHER____________________ 6<br>(SPECIFY)	
Q115	Outside your home, what language do you speak the most?<br><br>***Idan kin fita daga gida, wanne harshe/yarei kika fi magana da shi ko da yaushe?***	HAUSA……………………………..…1<br>YORUBA…………………………..….2<br>IGBO…………………………….……3<br>ENGLISH……………………………..4<br>PIDGIN ENGLISH……………….….5<br>OTHER_____________________6<br>(SPECIFY)	

SECTION 2: REPRODUCTION			
QNo.	Questions and filters	Coding categories	Skip to
Now I would like to ask you about all the births you have had during your life. Please be as honest as possible and know that your answers will not be shared with anyone else.<br><br>***Yanzu ina so nayi miki tambayoyi gameda dukkan haife- haife da ki ka yi a rayuwarki. Ki yi iyakar kokari ki gaya mini gaskiya, kuma ki sani cewa duk amsoshin ki za'a barsu a sirrance ba za a gayawa kowa ba.***			
Q201	Have you ever given birth?<br><br>***Kin taba haihuwa?***	YES………………………………… 1<br><br>NO………………………………. ….. 2 →	**Q206**
Q202	Do you have any sons or daughters to whom you have given birth who are now living with you?<br><br>***A yanzu haka kina da 'ya'ya maza ko mata da ki ka haifa wadanda kuke tare?***	YES…………………………….…….1<br><br>NO…………………………….……….2 →	**Q204**
Q203	How many of these sons live with you?<br>And how many daughters live with you?<br><br>***'Ya'yanki maza nawa ne kuke tare?***<br><br>***Da kuma 'ya'ya mata nawa ne kuke tare?***	SONS AT HOME………………. [__|__]<br><br>DAUGHTERS AT HOME………[__|__]	
Q204	Do you have any sons or daughters to whom you have given birth who are alive but do **not** live with you?<br><br>***Kina da 'ya'ya maza ko mata wadanda ki ka haifa, suna raye amma ba kwa zama tare dasu?***	YES…………………………………...1<br><br>NO…………………….…………….2 →	**Q206**
Q205	How many sons are alive but not living with you?<br><br>And how many daughters are alive but not living with you?<br><br>***'Ya'yan ki maza nawa ne ke raye amma ba kwa tare?***<br>***Da kuma 'ya'ya mata nawa ne ke raye amma ba kwa zama tare?***	SONS ELSEWHERE…………….…. [__|__]<br><br>DAUGHTERS ELSEWHERE………… [__|__]	
Q206	Sometimes it happens that children die. It may be painful to talk about and I am sorry to ask you about painful memories, but it is important to get correct information. Have you given birth to a boy or a girl who was born alive but later died?<br><br>***Wani lokaci yana faruwa cewa yara na mutuwa. Kiyi hakuri idan na tuna miki da abinda yariga ya wuce, amma ya kamata mu samu gaskiyar bayani. Kin taba haihuwan 'da namiji ko'ya mace wanda ya/ta zo da rai amma daga baya ya/ta rasu?***<br><br>IF NO, PROBE: Any baby who cried or showed signs of life but did not survive?<br><br>IF NO, PROBE:***Akwai 'dan da yayi kuka, ko ya nuna alamar rai amma daga baya ya rasu?***	YES…………………………………….1<br><br>NO……………………………….……….2 →	**Q208**
Q207	How many boys have died?<br><br>And how many girls have died?<br><br>**'Ya'ya maza nawa ne suka rasu?**<br><br>***Da kuma 'ya'ya mata nawa ne suka rasu?***	BOYS DEAD…………….……………… [__|__]<br><br>GIRLS DEAD…………………………… [__|__]	
Q208	SUM ANSWERS TO 203, 205, AND 207, AND ENTER TOTAL.<br><br>If none, record 00.	TOTAL…………………………..………[__|__]	

Q209	CHECK 208<br><br>Just to make sure that I have this right: you have had in TOTAL _______ birth. Is that correct?<br><br>***Dan tabbatar da ganin banyi kuskure ba, jimilar (dukka) 'ya'yan da ki ka haifa ……Hakane?***<br><br>YES ☐ NO ☐ → PROBE AND CORRECT 201-208 as necessary.	
Q210	ANY LIVE BIRTHS (Q208=1 OR MORE BIRTHS) ☐ NO LIVE BIRTHS (Q208=0) ☐ →	**Q233**

BIRTH HISTORY

Now I would like to record the names of all your births whether still alive or not, starting with the first one you had.
Yanzu Ina son in rubuta sunayen dukan 'ya'yan da kika haifa da wadanda ke raye hadi da wadanda suka rasu. Mu fara da haihuwar ki ta farko,
RECORD NAMES OF **ALL** THE **LIVE** BIRTHS in Q212. RECORD TWINS AND TRIPLETS ON SEPARATE LINES. IF THERE ARE MORE THAN 18 BIRTHS, USE AN ADDITIONAL QUESTIONNAIRE. STARTING WITH THE SECOND ROW. CIRCLE LINE NUMBER AND NAME OF ALL CHILDREN BORN IN JANUARY 2015 OR LATER.

(Q211) Line no.	(Q212) What name was given to your baby? *Wanne suna a ka ba wa da/yar ki (jaririn)?*	(Q213) Were any of these births twins/triplets? *Wannan haihuwar tagwaye ne ko 'yan ukku (3)?*	(Q214) Is (NAME) a boy or a girl? *Shin (Suna) namiji ne ko mace?*	(Q215) In what month and year was (NAME) born? PROBE: What is her/his birthday? (other probes: what season was it) *Wanne wata ne da shekara ki ka haifi (SUNA)?* *PROBE: Yaushe ne ranar haihuwar sa/ta? (Other probes: A wane lokaci (damina ko rani)?*	(Q216) Is (NAME) still alive? *Shin (SUNA) ya/ta na raye?*	(Q217) IF ALIVE: How old is (NAME) currently? RECORD AGE IN COMPLETED YEARS IF LESS THAN 1 YEAR, RECORD "00" *Idan 'dan/'yar ta/ya na raye, Shekarun (SUNA) nawa ne yanzu?*	(Q218) IF ALIVE: Is (Name) living with you? ***(SUNA) ya/ta na zama tare da ke?***	(Q219) **IF DEAD:** How old was (NAME) when she/he died? IF LESS THAN 2 YEARS, PROBE: ***Shekarun (SUNA) nawa ne kafin rasuwar sa/ta?*** How many months old was (NAME)? ***Watannin (SUNA) nawa ne kafin rasuwar sa/ta?*** IF LESS THAN 1 MONTH OLD, RECORD DAYS; MONTHS IF LESS THAN TWO YEARS; OR YEARS IF 2 OR MORE YEARS OLD	(Q220) Were there any other live births between (NAME OF PREVIOUS BIRTH) and (NAME), including any children who died after birth? ***Akwai wata haihuwa mai rai a tsakanin (NAME OF PREVIOUS BIRTH) da (NAME), Harda 'ya'yan da suka rasu bayan haihuwa?***
01	NAME: ______________________________	SING……1<br>MULT…..2	BOY…1<br>GIRL...2	MONTH [__|__]<br>YEAR [__|__][__|__]	YES….1<br>NO……2 → **Q219**	AGE IN YEARS<br>[__|__]	YES….1<br>NO……2<br>**ALL SKIP TO (02)**	DAYS……..1 [__|__]<br>MONTHS…2 [__|__]<br>YEARS……3 [__|__]<br>DK……….…….998	
02	NAME: ___________________________	SING……1<br>MULT…..2	BOY…1<br>GIRL...2	MONTH [__|__]<br>YEAR [__|__][__|__]	YES….1<br>NO……2 → **Q219**	AGE IN YEARS<br>[__|__]	YES….1<br>NO……2<br>**ALL SKIP TO Q220**	DAYS……..1 [__|__]<br>MONTHS…2 [__|__]<br>YEARS……3 [__|__]<br>DK……………..….998	YES…1 → ADD BIRTH<br>NO....2 → NEXT BIRTH
03	NAME: ______________________________	SING……1<br>MULT…..2	BOY…1<br>GIRL...2	MONTH [__|__]<br>YEAR [__|__][__|__]	YES….1<br>NO……2 → **Q219**	AGE IN YEARS<br>[__|__]	YES….1<br>NO……2<br>**ALL SKIP TO Q220**	DAYS……..1 [__|__]<br>MONTHS…2 [__|__]<br>YEARS……3 [__|__]<br>DK……………..….998	YES…1 → ADD BIRTH<br>NO....2 → NEXT BIRTH
04	NAME: ______________________________	SING……1<br>MULT…..2	BOY…1<br>GIRL...2	MONTH [__|__]<br>YEAR [__|__][__|__]	YES….1<br>NO……2 → **Q219**	AGE IN YEARS<br>[__|__]	YES….1<br>NO……2<br>**ALL SKIP TO Q220**	DAYS……..1 [__|__]<br>MONTHS…2 [__|__]<br>YEARS……3 [__|__]<br>DK……………..….998	YES…1 → ADD BIRTH<br>NO....2 → NEXT BIRTH

(Q211) Line no.	(Q212) What name was given to your baby?<br><br>***Wanne suna a ka ba wa da/yar ki (jaririn)?***	(Q213) Were any of these births twins/triplets?<br><br>***Wannan haihuwar tagwaye ne ko 'yan ukku (3)?***	(Q214) Is (NAME) a boy or a girl?<br><br>***Shin (Suna) namiji ne ko mace?***	(Q215) In what month and year was (NAME) born? PROBE: What is her/his birthday? (other probes: what season was it)<br><br>***Wanne wata ne da shekara ki ka haifi (SUNA)?***<br><br>***PROBE: Yaushe ne ranar haihuwar sa/ta?***<br>***(Other probes: A wane lokaci (damina ko rani)?***	(Q216) Is (NAME) still alive?<br><br>***Shin (SUNA) ya/ta na raye?***	(Q217) IF ALIVE: How old is (NAME) currently?<br><br>RECORD AGE IN COMPLETED YEARS<br><br>IF LESS THAN 1 YEAR, RECORD "00"<br><br>***Idan 'dan/'yar ta/ya na raye, Shekarun (SUNA) nawa ne yanzu?***	(Q218) IF ALIVE: Is (Name) living with you?<br><br>***(SUNA) ya/ta na zama tare da ke?***	(Q219) <u>**IF DEAD:**</u> How old was (NAME) when she/he died?<br>IF LESS THAN 2 YEARS, PROBE:<br>***Shekarun (SUNA) nawa ne kafin rasuwar sa/ta?***<br><br>How many months old was (NAME)?<br><br>***Watannin (SUNA) nawa ne kafin rasuwar sa/ta?***<br><br>IF LESS THAN 1 MONTH OLD, RECORD DAYS; MONTHS IF LESS THAN TWO YEARS; OR YEARS IF 2 OR MORE YEARS OLD	(Q220) Were there any other live births between (NAME OF PREVIOUS BIRTH) and (NAME), including any children who died after birth?<br><br>***Akwai wata haihuwa mai rai a tsakanin (NAME OF PREVIOUS BIRTH) da (NAME), Harda 'ya'yan da suka rasu bayan haihuwa?***
05	NAME:<br>______________________	SING……1<br>MULT…..2	BOY…1<br>GIRL...2	MONTH [__|__]<br>YEAR [__|__][__|__]	YES….1<br>NO……2 → **Q219**	AGE IN YEARS<br>[__|__]	YES….1<br>NO……2<br>**ALL SKIP TO Q220**	DAYS……..1 [__|__]<br>MONTHS…2 [__|__]<br>YEARS……3 [__|__]<br>DK…………..….998	YES…1→ ADD BIRTH<br>NO....2→ NEXT BIRTH
06	NAME:<br>______________________	SING……1<br>MULT…..2	BOY…1<br>GIRL...2	MONTH [__|__]<br>YEAR [__|__][__|__]	YES….1<br>NO……2 → **Q219**	AGE IN YEARS<br>[__|__]	YES….1<br>NO……2<br>**ALL SKIP TO Q220**	DAYS……..1 [__|__]<br>MONTHS…2 [__|__]<br>YEARS……3 [__|__]<br>DK…………..….998	YES…1→ ADD BIRTH<br>NO....2→ NEXT BIRTH
07	NAME:<br>______________________	SING……1<br>MULT…..2	BOY…1<br>GIRL...2	MONTH [__|__]<br>YEAR [__|__][__|__]	YES….1<br>NO……2 → **Q219**	AGE IN YEARS<br>[__|__]	YES….1<br>NO……2<br>**ALL SKIP TO Q220**	DAYS……..1 [__|__]<br>MONTHS…2 [__|__]<br>YEARS……3 [__|__]<br>DK…………..….998	YES…1→ ADD BIRTH<br>NO....2→ NEXT BIRTH
08	NAME:<br>______________________	SING……1<br>MULT…..2	BOY…1<br>GIRL...2	MONTH [__|__]<br>YEAR [__|__][__|__]	YES….1<br>NO……2 → **Q219**	AGE IN YEARS<br>[__|__]	YES….1<br>NO……2<br>**ALL SKIP TO Q220**	DAYS……..1 [__|__]<br>MONTHS…2 [__|__]<br>YEARS……3 [__|__]<br>DK…………..….998	YES…1→ ADD BIRTH<br>NO....2→ NEXT BIRTH
09	NAME:<br>______________________	SING……1<br>MULT…..2	BOY…1<br>GIRL...2	MONTH [__|__]<br>YEAR [__|__][__|__]	YES….1<br>NO……2 → **Q219**	AGE IN YEARS<br>[__|__]	YES….1<br>NO……2<br>**ALL SKIP TO Q220**	DAYS……..1 [__|__]<br>MONTHS…2 [__|__]<br>YEARS……3 [__|__]<br>DK…………..….998	YES…1→ ADD BIRTH<br>NO....2→ NEXT BIRTH

(Q211)<br>Line no.	(Q212)<br>What name was given to your baby?<br><br>***Wanne suna a ka ba wa da/yar ki (jaririn)?***	(Q213)<br>Were any of these births twins/triplets?<br><br>***Wannan haihuwar tagwaye ne ko 'yan ukku (3)?***	(Q214)<br>Is (NAME) a boy or a girl?<br><br>***Shin (Suna) namiji ne ko mace?***	(Q215)<br>In what month and year was (NAME) born? PROBE: What is her/his birthday? (other probes: what season was it)<br><br>***Wanne wata ne da shekara ki ka haifi (SUNA)?***<br><br>***PROBE: Yaushe ne ranar haihuwar sa/ta?***<br>***(Other probes: A wane lokaci (damina ko rani)?***	(Q216)<br>Is (NAME) still alive?<br><br>***Shin (SUNA) ya/ta na raye?***	(Q217)<br>IF ALIVE:<br>How old is (NAME) currently?<br><br>RECORD AGE IN COMPLETED YEARS<br><br>IF LESS THAN 1 YEAR, RECORD "00"<br><br>***Idan 'dan/'yar ta/ya na raye, Shekarun (SUNA) nawa ne yanzu?***	(Q218)<br>IF ALIVE:<br>Is (Name) living with you?<br><br>**(SUNA) ya/ta na zama tare da ke?**	(Q219)<br>**IF DEAD:**<br>How old was (NAME) when she/he died?<br>IF LESS THAN 2 YEARS, PROBE:<br>***Shekarun (SUNA) nawa ne kafin rasuwar sa/ta?***<br><br>How many months old was (NAME)?<br><br>***Watannin (SUNA) nawa ne kafin rasuwar sa/ta?***<br><br>IF LESS THAN 1 MONTH OLD, RECORD DAYS; MONTHS IF LESS THAN TWO YEARS; OR YEARS IF 2 OR MORE YEARS OLD	(Q220)<br>Were there any other live births between (NAME OF PREVIOUS BIRTH) and (NAME), including any children who died after birth?<br><br>***Akwai wata haihuwa mai rai a tsakanin (NAME OF PREVIOUS BIRTH) da (NAME), Harda 'ya'yan da suka rasu bayan haihuwa?***
10	NAME:<br>______________________________	SING……1<br>MULT…..2	BOY…1<br>GIRL...2	MONTH [__|__]<br>YEAR [__|__][__|__]	YES…..1<br>NO……2<br>↓ **Q219**	AGE IN YEARS<br>[__|__]	YES….1<br>NO……2<br>**ALL SKIP TO Q220**	DAYS……..1 [__|__]<br>MONTHS…2 [__|__]<br>YEARS …..3 [__I__]<br>DK……………..…998	YES…1→ ADD BIRTH<br>NO....2→ NEXT BIRTH
11	NAME:<br>______________________________	SING……1<br>MULT…..2	BOY…1<br>GIRL...2	MONTH [__|__]<br>YEAR [__|__][__|__]	YES…...1<br>NO…….2<br>↓ **Q219**	AGE IN YEARS<br>[__|__]	YES….1<br>NO……2<br>**ALL SKIP TO Q220**	DAYS……..1 [__|__]<br>MONTHS…2 [__|__]<br>YEARS …..3 [__I__]<br>DK……………..…998	YES…1→ ADD BIRTH<br>NO....2→ NEXT BIRTH
12	NAME:<br>___________________________	SING……1<br>MULT…..2	BOY…1<br>GIRL...2	MONTH [__|__]<br>YEAR [__|__][__|__]	YES…...1<br>NO…….2<br>↓ **Q219**	AGE IN YEARS<br>[__|__]	YES….1<br>NO……2<br>**ALL SKIP TO Q220**	DAYS……..1 [__|__]<br>MONTHS…2 [__|__]<br>YEARS …..3 [__I__]<br>DK……………..…998	YES…1→ ADD BIRTH<br>NO....2→ NEXT BIRTH
13	NAME:<br>______________________________	SING……1<br>MULT…..2	BOY…1<br>GIRL...2	MONTH [__|__]<br>YEAR [__|__][__|__]	YES…...1<br>NO…….2<br>↓ **Q219**	AGE IN YEARS<br>[__|__]	YES….1<br>NO……2<br>**ALL SKIP TO Q220**	DAYS……..1 [__|__]<br>MONTHS…2 [__|__]<br>YEARS …..3 [__I__]<br>DK……………..…998	YES…1→ ADD BIRTH<br>NO....2→ NEXT BIRTH
14	NAME:<br>______________________________	SING……1<br>MULT…..2	BOY…1<br>GIRL...2	MONTH [__|__]<br>YEAR [__|__][__|__]	YES…...1<br>NO…….2<br>↓ **Q219**	AGE IN YEARS<br>[__|__]	YES….1<br>NO……2<br>**ALL SKIP TO Q220**	DAYS……..1 [__|__]<br>MONTHS…2 [__|__]<br>YEARS …..3 [__I__]<br>DK……………..…998	YES…1→ADD BIRTH<br>NO....2→ NEXT BIRTH

(Q211) Line no.	(Q212) What name was given to your baby?<br><br>***Wanne suna a ka ba wa da/yar ki (jaririn)?***	(Q213) Were any of these births twins/triplets?<br><br>***Wannan haihuwar tagwaye ne ko 'yan ukku (3)?***	(Q214) Is (NAME) a boy or a girl?<br><br>***Shin (Suna) namiji ne ko mace?***	(Q215) In what month and year was (NAME) born? PROBE: What is her/his birthday? (other probes: what season was it)<br><br>***Wanne wata ne da shekara ki ka haifi (SUNA)?***<br><br>***PROBE: Yaushe ne ranar haihuwar sa/ta?***<br>(***Other probes: A wane lokaci (damina ko rani)?***	(Q216) Is (NAME) still alive?<br><br>***Shin (SUNA) ya/ta na raye?***	(Q217) IF ALIVE: How old is (NAME) currently?<br><br>RECORD AGE IN COMPLETED YEARS<br><br>IF LESS THAN 1 YEAR, RECORD "00"<br><br>***Idan 'dan/'yar ta/ya na raye, Shekarun (SUNA) nawa ne yanzu?***	(Q218) IF ALIVE: Is (Name) living with you?<br><br>**(SUNA) ya/ta na zama tare da ke?**	(Q219) **<u>IF DEAD:</u>** How old was (NAME) when she/he died? IF LESS THAN 2 YEARS, PROBE:<br>***Shekarun (SUNA) nawa ne kafin rasuwar sa/ta?***<br><br>How many months old was (NAME)?<br><br>***Watannin (SUNA) nawa ne kafin rasuwar sa/ta?***<br><br>IF LESS THAN 1 MONTH OLD, RECORD DAYS; MONTHS IF LESS THAN TWO YEARS; OR YEARS IF 2 OR MORE YEARS OLD	(Q220) Were there any other live births between (NAME OF PREVIOUS BIRTH) and (NAME), including any children who died after birth?<br><br>***Akwai wata haihuwa mai rai a tsakanin (NAME OF PREVIOUS BIRTH) da (NAME), Harda 'ya'yan da suka rasu bayan haihuwa?***
15	NAME:<br>______________________________	SING……1<br>MULT…..2	BOY…1<br>GIRL...2	MONTH [__|__]<br>YEAR [__|__][__|__]	YES…...1<br>NO…….2<br>↓ **Q219**	AGE IN YEARS<br>[__|__]	YES….1<br>NO……2<br>**ALL SKIP TO Q220**	DAYS……..1 [__|__]<br>MONTHS…2 [__|__]<br>YEARS …..3 [__|__]<br>DK……………..…998	YES…1→ ADD BIRTH<br>NO....2→ NEXT BIRTH
16	NAME:<br>______________________________	SING……1<br>MULT…..2	BOY…1<br>GIRL...2	MONTH [__|__]<br>YEAR [__|__][__|__]	YES…...1<br>NO…….2<br>↓ **Q219**	AGE IN YEARS<br>[__|__]	YES….1<br>NO……2<br>**ALL SKIP TO Q220**	DAYS……..1 [__|__]<br>MONTHS…2 [__|__]<br>YEARS …..3 [__|__]<br>DK……………..…998	YES…1→ ADD BIRTH<br>NO....2→ NEXT BIRTH
17	NAME:<br>___________________________	SING……1<br>MULT…..2	BOY…1<br>GIRL...2	MONTH [__|__]<br>YEAR [__|__][__|__]	YES…...1<br>NO…….2<br>↓ **Q219**	AGE IN YEARS<br>[__|__]	YES….1<br>NO……2<br>**ALL SKIP TO Q220**	DAYS……..1 [__|__]<br>MONTHS…2 [__|__]<br>YEARS …..3 [__|__]<br>DK……………..…998	YES…1→ ADD BIRTH<br>NO....2→ NEXT BIRTH

Q221	Have you had any live births since the birth of (NAME OF LAST BIRTH)?<br><br>***Kin sake samun haihuwa tun haihuwar (NAME OF LAST BIRTH)?***	YES……………………………..…1 →<br><br>NO…………………………..…..2	RECORD BIRTH(S) IN BIRTH HISTORY TABLE
Q222	Before the birth of (NAME OF FIRST BIRTH), did you have any other live births?<br><br>***Kafin haihuwar (NAME OF FIRST BIRTH), kin kara samu wata haihuwa mai rai?***	YES…………………………………1 →<br><br>NO…………………………………...2	RECORD BIRTH(S) IN BIRTH HISTORY TABLE
Q223	COMPARE Q208 WITH NUMBER OF BIRTHS IN HISTORY ABOVE AND MARK:<br>NUMBERS ARE SAME ☐ ↓ NUMBERS ARE DIFFERENT ☐ → (PROBE AND RECONCILE)<br><br>CHECK: FOR EACH BIRTH: YEAR OF BIRTH IS RECORDED. ☐<br>FOR EACH LIVING CHILD: CURRENT AGE IS RECORDED. ☐<br>FOR EACH DEAD CHILD: AGE AT DEATH IS RECORDED. ☐<br>FOR AGE AT DEATH 12 MONTHS OR 1 YEAR: PROBE TO DETERMINE EXACT NUMBER OF MONTHS. ☐		
Q224<br>**C**	FOR EACH BIRTH **SINCE JANUARY 2012**, ENTER 'B' IN THE MONTH OF BIRTH IN COLUMN 1 OF THE CALENDAR. WRITE THE NAME OF THE CHILD TO THE LEFT OF THE 'B' CODE. FOR EACH BIRTH, ASK THE NUMBER OF MONTHS THE PREGNANCY LASTED AND RECORD 'P' IN EACH OF THE PRECEDING MONTHS ACCORDING TO THE DURATION OF PREGNANCY. (NOTE: THE NUMBER OF 'P's MUST EQUAL THE NUMBER OF COMPLETED MONTHS OF PREGNANCY.)		
Q225	CHECK Q215 AND ENTER THE NUMBER OF **BIRTHS SINCE 2015**. IF NONE, RECORD "0". ☐		
Q226	CHECK Q225:<br>YES, HAS ONE OR MORE BIRTHS SINCE **January 2015** ☐ ↓<br>NO BIRTHS SINCE **January 2015** ☐ →		**Q233**
Q227	ENTER NAME AND LINE NUMBER OF YOUNGEST CHILD BORN SINCE JANUARY 2015 FROM Q211 AND Q212:<br><br>--- ☐☐<br>NAME LINE NUMBER<br><br>LIVING (Q216=1) ☐ ↓<br>DEAD (Q216=2) ☐ →		**Q231**
Q228	Are you currently breastfeeding (NAME OF LAST CHILD)?<br><br>**Kina shayar da (NAME OF LAST CHILD) nono ne yanzu?**	YES………………………………………… 1<br><br>NO………………………………………… 2 →	 <br>**Q231**
Q229	How many times did you breastfeed (NAME) in the last 24 hours?<br><br>**Kamar sau nawa kika shayar da (NAME OF LAST CHILD) nono a cikin awa 24 da ta shige?**<br><br>IF NUMBER IS NOT NUMERIC, PROBE FOR APPROXIMATE NUMBER.	NUMBER OF FEEDINGS………………… ☐☐	
Q230	In the last 24 hours, did (NAME) drink anything from a bottle with a nipple or eat any foods or liquids?<br><br>**A cikin awa ashirin da hudu (24) da suka wuce, Shin (NAME) ya/ta sha wani abu daga kwalba mai bulunboti ko ya/ta ci wani abinci ko abinci mai ruwa-ruwa**?	YES…………………………………………… 1<br><br>NO……………………………………………... 2<br><br>DON'T KNOW………………………………… 8	

Q231	At the time you became pregnant with [NAME ABOVE], did you want to become pregnant then, did you want to wait until later, or did you not want to have any more children at all?<br><br>***A lokacin da ki ka sami cikin (SUNA), kin so dauka ciki ne, ko kin so ki jinkirta sai zuwa gaba, ko kuma ba ki da sha'awar sake haihuwar ko kadan?***	THEN……………………………………..…. 1 →<br>LATER…………………………………..… 2<br>NOT AT ALL……………………………… 3 →	**Q233**<br><br>**Q233**
Q232	How much longer would you have liked to wait?<br><br>***Har tsawon wanne lokaci ki ka so ki jira?***<br><br>IF RESPONSE IS A DECIMAL FOR YEARS (2.5YRS), THEN CONVERT INTO MONTHS AND FILL IN ONLY MONTHS.	MONTHS……………………….……1 [__|__]<br>OR<br>YEARS..………………….……………2 [__|__]<br>OR<br>OTHER (SPECIFY)_________________996<br>OR<br>DON'T KNOW……………….……………...998	
Q233	Are you currently pregnant now?<br><br>***A yanzu haka, kina da ciki ne?***	YES………………………………………..… 1<br>NO……………………………….…………. 2 →<br>UNSURE…………………………………… 8 →	 **Q240**<br>**Q240**
Q234<br><br>**C**	How many months pregnant are you?<br><br>**Cikin na ki wata nawa ne?**<br><br>RECORD NUMBER OF COMPLETED MONTHS.<br><br>ENTER 'P'S IN THE CALENDAR IN COLUMM 1, BEGINNING WITH THE MONTH OF INTERVIEW AND FOR THE TOTAL NUMBER OF COMPLETED MONTHS.	MONTHS………………………… [__|__]	
Q235	At any time during this pregnancy, have you gone for an antenatal check up?<br><br>**Shin a yayin da kike da wanan cikin, kin je awon ciki?**	YES……….………………………………... 1<br>NO…………………………...…………… 2 →	 **Q238**

Q235a	From where or whom did you obtain (A CHECK UP) last time? PROBE: What is the name of this place/person? And where is it/he/she located?<br><br>**Daga ina ko wajen wanene ki ka samu yin awon ciki na karshe?**<br><br>**PROBE: Menene sunan wurin ko ma'aikacin? Kuma a ina ne wurin kuma ma'akacin shi/ita a ina ya/ta ke?**<br><br>WRITE NAME OF PERSON ONLY IF PROVIDER WORKS AS AN INDIVIDUAL	NAME OF FACILITY /PERSON<br>________________________________<br>________________________________<br>*CODE BOXES: OFFICE ONLY*<br>[__|__|__|__|__|__]<br><br>STREET NAME/ADDRESS_________________<br>______________________________<br><br>LAND MARK DESCRIPTION___________________	

Q236	From which type of facility did you obtain (A CHECK UP) last time?<br><br>**Daga wacce cibiyar kiwon lafiya ki ka yi awon karshe?**	**PUBLIC SECTOR**<br>GOVT HOSPITAL………………….……. 11<br>WOMEN AND CHILDREN'S HOSPITAL…………………….......... .. 12<br>CHILD WELFARE CLINIC………………….. 13<br>GOVT. HEALTH CENTER……….......... 14<br>GOVERNMENT POST/DISPENSARY...... . 15<br>MATERNITY HOME………......………… 16<br>MOBILE CLINIC…….………….......... 17<br>OTHER PUBLIC___________________ 18<br>(SPECIFY)<br><br>**PRIVATESECTOR**<br>PRIVATE HOSPITAL/CLINIC..................... 21<br>PRIVATE DOCTOR'S OFFICE……...…..... 22<br>NURSING/MATERNITY HOME……....…… 23<br>PHARMACY…………………..……..…… 24<br>PMS/CHEMIST…………………..…..…… 25<br>MOBILE CLINIC………….……….......... 26<br>CHW/TBA…………………….…….......... 27<br>TRADITIONAL HEALER………..…….......... 28<br>OTHER PRIVATE___________________ 29<br>(SPECIFY)<br><br>**FAITH-BASED SECTOR**<br>MISSION HOSPITAL………….............. 31<br>FAITH-BASED, CHURCH CLINIC…..…… 32<br><br>**OTHER SOURCE**<br>OTHER NGO HOSPITAL/CLINIC………… 41<br>WORKSITE CLINIC………….…………..… 42<br>OTHER ___________________________<br>(SPECIFY) 96<br>DON'T KNOW . . . .………. 98	
Q237	Was this place you received your most recent antenatal visit in this city, in another city or town, or in a rural area?<br><br>**Wanne wurin ki ka samu kulawar awon ciki na karshe, a wannan birnin ne, ko wani birnin ko garin ko kuma a wata karkara ne?**	THIS CITY/TOWN ………………………..... 1<br>ANOTHER CITY/TOWN…………………. 2<br>A RURAL AREA ………………………..… 3	
Q238	At the time you became pregnant, did you want to become pregnant then, did you want to wait until later, or did you not want to have any more children at all?<br><br>***A lokacin da ki ka sami ciki, kin so dauka ne, ko kin so ki jinkirta sai zuwa gaba, ko kuma ba kida sha'awar sake haihuwar ko kadan?***	THEN……………………..……...……..... 1 →<br><br>LATER……………………………………… 2<br><br>NOT AT ALL…………………………….… 3 →	**Q240**<br><br><br><br>**Q240**
Q239	How much longer would you have liked to wait?<br><br>***Har tsawon wane lokaci kika so ki jira?***<br><br>IF RESPONSE IS A DECIMAL FOR MONTHS & YEARS (2.5YRS), THEN CONVERT IN MONTH AND FILL IN ONLY MONTHS.	MONTHS…………………….……..1 [__|__]<br>OR<br>YEARS..………………………………..2 [__|__]<br>OR<br>OTHER (SPECIFY)_________________996<br>OR<br>DON'T KNOW………………………………998	
Q240	When did your last menstrual period start?<br><br>***Yaushe ki ka fara ganin al'da/hailar ki ta karshe?***<br><br>______________________________<br>(DATE, IF GIVEN)<br><br>IF LESS THAN ONE DAY, CIRCLE "1" AND WRITE "00" DAYS AGO.	DAYS AGO……………………….…….1 [__|__]<br>WEEKS AGO……………………...…….2 [__|__]<br>MONTHS AGO…………………...……. 3 [__|__]<br>YEARS AGO……………………...…….4 [__|__]<br><br>IN MENOPAUSE/HAS HAD HYSTERECTOMY…………………….……994<br>BEFORE LAST BIRTH……………………......995<br>NEVER MENSTRUATED……………….…..996<br>CANT REMEMBER…………………..…..998	

Q241	From one menstrual period to the next, are there certain days when a woman is more likely to get pregnant if she has sex?<br><br>***Shin daga haila zuwa wata hailar da akwai ranakun da mace zata iya daukan ciki idan ta yi jima'i?***	YES……………………….……………1<br>NO…………………………….……………..2 →<br>DON'T KNOW……………….……………..8 →	 <br>**Q243**<br>**Q243**
Q242	Which days are these? Would you say that this time is just before her period begins, during her period, right after her period has ended, or halfway between two periods?<br><br>***Wadanne kwanaki ne?Shin za ki iya cewa wannan lokacin yana iya faruwa dab da ta fara haila ne, lokacin da take haila ne, dab da ta gama haila ne ko kuma tsakanin haila da haila?***	JUST BEFORE HER PERIOD BEGINS…... 1<br>DURING HER PERIOD…………………..…… 2<br>RIGHT AFTER IT ENDS..……......……….. 3<br>HALFWAY BETWEEN TWO PERIODS….. 4<br><br>OTHER___________________________ 6<br>(SPECIFY)<br>DON'T KNOW……………….……………….. 8	
Q243	Do you think that a woman who is breastfeeding her baby can become pregnant?<br><br>***Shin a tunanin ki matar da ke shayar da 'da/'ya na iya daukar ciki?***	YES . . . .…….……….. 1<br>NO . . . .…..…….…… 2<br>DEPENDS……………..………….…………. 3<br>DON'T KNOW . . . . ….…….……. 8 →	 <br><br>**Q245**
Q244	What are the criteria in which breastfeeding is effective as a family planning / birth spacing method?<br>Anything else?<br><br>MARK ALL THAT APPLY.<br><br>***Wadanne matakai ne masu kyau na shayar da nono da suka dace da kayyade iyali/tazarar haihuwa?***<br><br>***Akwai wani abu kuma?***	EXCLUSIVE BREASTFEEDING<br>(NO SUPPLEMENTS)……………. …….. A<br>EXCLUSIVE BREASTFEEDING<br>FOR UP TO 6 MONTHS………………..... B<br>MENSTRUAL PERIOD HASN'T<br>RETURNED………..…………………… C<br><br>OTHERS ___________________________ X<br>(SPECIFY)<br>NO CRITERIA/NOT A METHOD ………… Y<br>DON'T KNOW……………………………….. Z	

CHECK FOR PRESENCE OF OTHERS. BEFORE CONTINUING, MAKE EVERY EFFORT TO ENSURE PRIVACY.			
Q245	Have you ever had a pregnancy that ended in a miscarriage, abortion or stillbirth?<br><br>***Kin taba yin cikin da ki ka yi barin cikin, ya zube da kanshi, ko dan ya zo ba rai?***<br><br>By <u>miscarriage</u> I mean a pregnancy that <u>ended just by itself</u>.<br>***Abun nufi da bari ina nufin cikin da ya zube da kanshi***<br><br>By <u>abortion</u>, I mean a pregnancy that ended, because <u>you did something or used something</u>.<br>***Abun nufi da zubar da ciki, ina nufin cikin da ya zube domin kin yi wani abu ko amfani da wani abu, misali aka cire maki.***<br><br>By <u>still birth</u>, I mean a baby that was born at full term and <u>did not show any sign of life</u>.<br>***Abun nufi da haihuwar da 'dan baizo da rai ba ina nufin haihuwar da cikin yai wata tara amma bai nuna alamar rai ba.***	YES……………………………….1<br><br>NO………………………………..2	**Q253**
Q246	How many miscarriages have you ever had?<br>**Sau nawa ki ka ta ba yin bari?**	NUMBER OF MISCARRIAGES…………[__]__]	
Q247	How many abortions have you ever had?<br>***Sau nawa ki ka ta ba zubda ciki?***	NUMBER OF ABORTIONS……………..…[__]__]	
Q248	How many still births have you ever had?<br>**Sau nawa kika taba haihuwa amma 'dan/'yar bai/bata zo da rai ba?**	NUMBER OF STILL BIRTHS……………………..…………..….[__]__]	

C	PREGNANCY OUTCOME SINCE JANUARY 2012	249. Since January 2012, have you had a pregnancy that ended in a (PREGNANCY OUTCOME)?<br><br>***Tun January 2012, Kin ta ba daukar cikin da ya kasance akwai haihuwa*** (PREGNANCY OUTCOME)?	250. How many times has this occurred since January 2012?<br><br>***Sau nawa hakan ya faru tun daga January 2012?***	251. When did the last such pregnancy end?<br><br>***Yaushe wannan daukar cikin ya faru na karshe?***	252. How many months pregnant were you when the last such pregnancy ended?<br><br>***Ki na da cikin wata nawa lokacin da irin wannan ya faru na karshe?***<br><br>ENTER 'M' FOR MISCARRIAGE, 'A' FOR ABORTION, OR 'S' FOR STILLBIRTH IN COLUMN 1 OF THE CALENDAR IN THE MONTH IN WHICH THE PREGNANCY WAS TERMINATED. ENTER 'P's FOR THE NUMBER OF COMPLETED MONTHS. THE TOTAL NUMBER OF 'P's MUST BE ONE LESS THAN THE NUMBER OF MONTHS PREGNANT AT THE TIME OF THE TERMINATION. FOLLOW THIS PROCEDURE FOR THE NUMBER OF TIMES THE EVENT HAPPENED ACCORDING TO Q250.
(01)	Stillbirth (pregnancy was full term but the baby showed no signs of life)<br><br>***'Dan/'yar da baizo da rai ba***	YES…..………….1<br>NO……….…….2<br>(02)	NUMBER OF TIMES……..[__|__]	MONTH…….[__|__]<br><br>YEAR….[__|__|__|__]	MONTHS…….[__|__]
(02)	Miscarriage (pregnancy ended just by itself)<br><br>***Cikin da ya zube da kanshi***	YES…..………….1<br>NO……….…….2<br>(03)	NUMBER OF TIMES……..[__|__]	MONTH…….[__|__]<br><br>YEAR….[__|__|__|__]	MONTHS…….[__|__]
(03)	Abortion (pregnancy ended by something you did or used, e.g. manual vacuum aspiration)<br><br>***Cikin da ya zube domin kin yi wani abu ko amfani da wani abu, misali aka cire maki.***	YES……………...1<br>NO…..…………2<br>Q253	NUMBER OF TIMES……..[__|__]	MONTH………[__|__]<br><br>YEAR….[__|__|__|__]	MONTHS…….[__|__]

Q253	CHECK Q208 AND Q245:<br><br>ONE OR MORE BIRTHS, ABORTIONS, MISCARRIAGES, OR STILLBIRTHS (Q208=1 OR MORE **OR** Q245=1) ☐ ↓	HAS HAD NO BIRTHS (Q208=0), NO ABORTIONS, NO MISCARRIAGE, **AND** NO STILLBIRTHS (Q245=2) ☐ →	**Q301**
Q254	How old were you when you got pregnant for the very **first time?**<br><br>**Shekarar ki nawa lokacin da ki ka dauki cikin farko?**	[___]___]<br>RECORD EXACT AGE	
Q255	At the time you became pregnant the first time, did you want to become pregnant **then**, did you want to wait until **later,** or did you **not want** to have any children at all?<br><br>***A lokacin da ki ka sami ciki, kin so dauka ne, ko kin so ki jinkirta sai zuwa gaba, ko kuma ba kida sha'awar sake haihuwar ko kadan?***	THEN………………………………..……………..1 →<br>LATER………………………. …………….. …..2<br>NOT AT ALL………………………………………3 →	**Q301**<br><br>**Q301**
Q256	How much longer would you have liked to wait?<br><br>***Har tsawon wane lokaci kika so ki jira?***<br><br>IF RESPONSE IS A DECIMAL FOR MONTHS & YEARS (2.5YRS), THEN CONVERT IN MONTH AND FILL IN ONLY MONTHS.	MONTHS……..1 [__][__]<br>OR<br>YEARS………...2 [__][__]<br>OR<br>OTHER (SPECIFY)______________996<br>OR<br>DON'T KNOW…………………………998	

SECTION 3: CONTRACEPTION

Qno	Questions and filters	Coding categories	Skip to

CHECK FOR PRESENCE OF OTHERS. BEFORE CONTINUING, MAKE EVERY EFFORT TO ENSURE PRIVACY.

Now I would like to talk about family planning/child spacing/birth spacing, the various ways or methods that a couple/ partners can use to delay or avoid a pregnancy.

Yanzu ina so in yi magana akan dabarun tsarin iyali/tazara tsakanin haihuwa/ tazara tsakanin yara daban daban da ma'aurata masu zama tare kebi don jinkirta daukar ciki ko guje wa daukan ciki.

INSTRUCTIONS: CIRCLE '1' FOR EACH METHOD MENTIONED SPONTANEOUSLY. THEN PROCEED DOWN COLUMN READING THE NAME AND DESCRIPTION OF EACH METHOD NOT MENTIONED SPONTANEOUSLY. CIRCLE '2' FOR EACH METHOD RECOGNIZED UPON LISTENING TO DESCRIPTION. CIRCLE '3' FOR EACH METHOD NOT MENTIONED NOR RECOGNIZED.

Q301. Which ways or methods have you heard of? **Wadanne dabarun tsarin iyali daban-daban ki ka ta ba ji?**

PROBE: HAVE YOU HEARD OF ANY OTHER WAYS OR METHODS THAT WOMEN OR MEN CAN USE TO AVOID PREGNANCY?

FOR METHODS NOT MENTIONED SPONTANEOUSLY, ASK: Have you ever heard of (METHOD)? **Kin taba jin (METHOD)?**

Q302. Have you ever used (METHOD)?

Kin taba amfani da? (METHOD)?

		Yes, MENTIONED SPONTANEOUSLY	Yes, WHEN PROBED	No	Q302
01	FEMALE STERILIZATION/ TUBAL LIGATION: Women can have an operation to avoid having any more children.<br><br>***Ana iya yi wa mata aiki a mahaifa don kar su sake samun haihuwa.***	1	2	3→ **Next Method**	Have you ever had an operation to avoid having any more children?<br>Shin ko an taba yi maki aiki domin kar ki sake haihuwa?<br><br>YES………………. 1<br>NO………………… 2
02	MALE STERILIZATION/VASECTOMY: Men can have an operation to avoid having any more children.<br><br>***Ana iya yi wa maza dandaka don hana haihuwa***	1	2	3→ **Next Method**	Has your partner ever had an operation to avoid having any more children?<br>Shi ko an taba yi ma mijinki/abokin zaman ki aiki domin kar ya sake haihuwa<br><br>YES………………. 1<br>NO………………… 2
03	DAILY PILL: Women can take a pill every day to avoid becoming pregnant.<br><br>***Mata na iya shan kwayar magani a ko wacce rana don hana daukan ciki***	1	2	3→ **Next Method**	YES………………. 1<br>NO………………… 2
04	IUD: Women can have a loop or coil placed inside them by a health provider.<br><br>***Likitoci ko jami'an kula da kiwon lafiya kan sawa mata wata irin roba/ karfe a farjinsu don hana daukan ciki.***	1	2	3→ Next Method	YES………………. 1<br>NO………………… 2
05	INJECTABLES OR INJECTIONS: Women can have an injection by a health provider that stops them from becoming pregnant for one or more months.<br><br>***Jami'in kiwon lafiya na yi wa mata allura wadda za ta hana su samun ciki na wata daya ko fiye da haka.***	1	2	3→ Next Method	YES………………. 1<br>NO………………… 2

06	IMPLANTS/IMPLANON/JADELLE: Women can have a small rod placed in their upper arm by a health provider, which can prevent pregnancy for one to three years.<br><br>***Likita ko jami'in kiwon lafiya kan sawa mata wasu kananan karafa a dantsen su don su hana daukan ciki na tsawon shekara daya(1) zuwa shekera ukku (3)?***	1	2	3 → Next Method	YES………………. 1<br>NO………………… 2
07	MALE CONDOM: Men can put a rubber sheath on their penis before sexual intercourse.<br><br>**Maza na iya sa kororon roba a azzakarin su kafin suyi jima'i**	1	2	3 → Next Method	YES………………. 1<br>NO………………… 2
08	FEMALE CONDOM: Women can place a sheath in their vagina before sexual intercourse.<br><br>**Mata na iya sanya irin na su kororon roba a farjin su kafin su yi jima'i**	1	2	3 → Next Method	YES………………. 1<br>NO………………… 2
09	STANDARD DAYS/CYCLE BEADS: Every month that a woman is sexually active she can avoid pregnancy by not having sexual intercourse on the days of the month she is most likely to get pregnant. She keeps track of this using a color-coded string of beads that indicates fertile and non-fertile days of a menstrual cycle.<br>**A kowanne wata da mace ke bukatar yin jima'i zata iya gujewa daukar ciki idan ba ta yi jima'i ba a ranakun da zata iya daukar ciki.**<br>**Mace na iya amfani da zaren da aka daura ma duwatsun ado masu kala domin ta san ranar da za ta iya daukar ciki da kuma ranakun da baza ta iya daukar ciki ba lokacin zagayowar al'adar ta.**	1	2	3 → Next Method	YES………………. 1<br>NO………………… 2
10	RHYTHM METHOD: Every month that a woman is sexually active she can avoid pregnancy by not having sexual intercourse on the days of the month she is most likely to get pregnant.<br><br>***Mace na iya gujewa yin jima'i daidai lokacin da take ganin tana iya daukan ciki.***	1	2	3 → Next Method	YES………………. 1<br>NO………………… 2
11	WITHDRAWAL Men can be careful and pull out before climax.<br>***Namiji na iya zare azzakarin sa a yayin jima'i daidai lokacin da ya ji zai yi zuwan kai.***	1	2	3 → Next Method	YES………………. 1<br>NO………………… 2
12	EMERGENCY CONTRACEPTION/MORNING AFTER PILL/ POSTINOR 2 Women can take pills up to 3 days after sexual intercourse to avoid becoming pregnant.<br><br>***Don gujewa daukan ciki mace na iya shan kwayoyin maganin hana daukan ciki har na tsawon kwanaki ukku(3) bayan jima'i***	1	2	3 → Next Method	YES………………. 1<br>NO………………… 2
13	LACTATIONAL AMENORRHEA (LAM) Up to six (6) months after childbirth, a woman can use a method that requires that she feeds the baby with only breastmilk (no other formula, water or other food) and that her menstrual period has not returned.<br><br>***Mata kan yi amfani da hanyar shayar da nono (banda wani abinci ko sanadari/madara/ruwa) akai-akai dare da rana har na tsawon wata shida (6), wanda haka yakansa rashin ganin jinin hailar su***	1	2	3 → Next Method	YES………………. 1<br>NO………………… 2
14	SPERMICIDE Women can place a suppository, jelly, or cream in their vagina before intercourse.<br><br>**Mace zata iya shafa wani irin mai ko basilin (mai wanda yake da wani sanadari) a al'aurar ta kafin ta yi jima'i.**	1	2	3 → Next Method	YES………………. 1<br>NO………………… 2
15	DIAPHRAGM Women can place a thin flexible disk in their vagina before intercourse.<br>**Mace na iya sa wani irin faifai mara karfi a cikin farjin ta kafin ta yi jima'i.**	1	2	3 → Next Method	YES………………. 1<br>NO………………… 2

16	Have you heard of any other ways or methods that women or men can use to avoid pregnancy?<br><br>***Kin ta ba jin wata dabara ko ta gargajiya da mata ko maza ke amfani dasu don gujewa daukan ciki?***	YES______________ 1<br>(SPECIFY)<br><br>NO…………………… 3 → Q303	YES……………… 1<br>NO………………… 2
17	NOTE: IF RESPONDENT MENTIONS ABSTINENCE AS A METHOD OF FP, DO NOT RECORD AS A METHOD. PROBE FOR ANY OTHER METHOD KNOWN.	YES______________ 1<br>(SPECIFY)<br>NO………………… 3 → Q303	YES……………… 1<br>NO………………… 2

Q303	CHECK 301 (KNOWLEDGE OF ANY CONTRACEPTIVE METHOD).<br>IF 301=YES FOR ANY METHOD ☐ ↓ IF 301=NO FOR ALL METHOD ☐ →	**Q317**

Q304	In the past year, from whom or where have you seen or heard about birth spacing or family planning?<br><br>***A shekara da ta shige, daga wurin wa ko ina ki ka ga ko ki ka ji maganar tazarar haihuwa ko kayyade iyali?***<br><br>CIRCLE ALL RESPONSES MENTIONED.<br><br>PROBE: ANY OTHER?<br><br>**A ina kuma?**	**MEDIA SOURCES**<br>RADIO……………………………………… AA<br>TV ………………………………………… AB<br>NEWSPAPERS…………………………… AC<br>MAGAZINES……………………………… AD<br>BILLBOARDS…………………………… AE<br>WALL PAINTING………………………… AF<br>MOBILE PHONE/SMS………………… AG<br>INTERNET……………………………… AH<br>CINEMA………………………………… AI<br>LIVE DRAMA/PUPPET SHOW………….. AJ<br>POSTER………………………………… AK<br>LEAFLET/BROCHURE…………………… AL<br><br>**PUBLIC SECTOR**<br>GOVERNMENT HOSPITAL ………… . . . . BA<br>WOMEN AND CHILDREN HOSPITAL /CHILD WELFARE CLINIC………………… BB<br>GOVT. HEALTH CENTER . . . . . . ……….. BC<br>GOVERNMENT POST/DISPENSARY…… BD<br>OTHER PUBLIC___________________ BE<br>(SPECIFY)<br>**PRIVATE/FBO/NGO SECTOR**<br>FAITH-BASED, CHURCH, MISSION HOSPITAL/CLINIC.................................... CA<br>PRIVATE HOSPITAL/CLINIC................... CB<br>NURSING/MATERNITY HOME…………… CC<br>TRADITIONAL BIRTH ATTENDANT……. CD<br>COMMUNITY MIDWIFE…………………… CE<br>COMMUNITY HEALTH WORKER ……….. CF<br>TRADITIONAL HEALER…………………… CG<br>PHARMACY………………………………… CH<br>PMS/CHEMIST……………………………… CI<br><br>**OTHER SOURCE**<br>WORKSITE CLINIC……………………….. DA<br>MOBILE CLINIC . . . . ….... DB<br>YOUTH CENTER………………………….. DC<br>VCT……..………………………............ DD<br>BAR………………………………………… DE<br>KIOSK/SHOP/MARKET…………………… DF<br><br>**INTERPERSONAL SOURCES**<br>TEACHER…………………………………… EA<br>PARENTS…………………………………… EB<br>PARENTS-IN-LAW………………………….. EC<br>SPOUSE/PARTNER……………………….... ED<br>SISTER/BROTHER………………………… EE<br>SISTER-IN-LAW/BROTHER-IN- LAW…… EF<br>FRIENDS//NEIGHBOURS…………………. EG<br>GRANDPARENTS…………………………. EH<br>PEER EDUCATOR………………………… EI<br>WOMEN'S GROUP……………………….. EJ<br><br>OTHER (SPECIFY)_________________ XX<br>HAS NOT SEEN OR HEARD IN THE LAST YEAR…………………………………………… YY<br>DON'T KNOW WHERE HEARD…………….. ZZ	

Q305	Have you ever recommended any family planning methods to your friends and/or relatives?<br><br>**Kin ta ba baiwa kawayenki ko 'yanuwanki shawarar kaiyade iyali?**	YES………………………………………………. 1<br><br>NO………………………………………………… 2	→ Q308
Q306	Which methods have you recommended?<br><br>**Wacce irin dabarar ki ka bada shawara akai**?<br><br>CIRCLE ALL MENTIONED.<br><br>IF RESPONDENT SAYS "PILL", PROBE FURTHER TO ESTABLISH IF THEY MEAN THE "DAILY PILL" OR THE "EMERGENCY PILL"	FEMALE STERILIZATION…………………….. .... A<br>MALE STERILIZATION……………………… .... B<br>IMPLANT………………………………………. .... C<br>IUD……………………………………………….. .... D<br>INJECTABLE………………………………… .... E<br>DAILY PILL……………………………………........ F<br>EMERGENCY PILL (Postnor2, etc)……….......... G<br>MALE CONDOM…………………………….......... H<br>FEMALE CONDOM……………………………....... …. I<br>STANDARD DAYS METHOD/CYCLE BEADS……………………………………...... … J<br>BREASTFEEDING/LAM……………………....... …. K<br>OTHER MODERN METHOD<br>______________________________ L<br>(SPECIFY)<br>RHYTHM METHOD. .... M<br>WITHDRAWAL.. N<br>OTHER TRADITIONAL METHOD<br>______________________________ X<br>(SPECIFY)	
Q307	When was the last time that you made a family planning method recommendation to your friends and/or relatives?<br><br>**Yaushe ne lokacin karshe da kika bada shawarar tazarar kaiyade iyali zuwa ga kawaye da 'yanuwan ki?**	LESS THAN A YEAR AGO .... 1<br>1-2 YEARS AGO .... 2<br>3-5 YEARS AGO .... 3<br>MORE THAN 5 YEARS AGO................................. 4<br>DON'T REMEMBER... 8	
Q308	CHECK 302:<br>AT LEAST ONE "YES" (EVER USED) ☐ ↓<br><br>NOT A SINGLE "YES" (NEVER USED) ☐ →		**Q313**
Q309	Now I would like to ask you about the **first** time that you did something or used a method to avoid getting pregnant.<br><br>How many living children did you have at that time, if any?<br>How many boys?<br>And how many girls?<br><br>**Yanzu ina son in tambayeki game da lokacin da ki ka fara yin wani abun ko kuma ki ka fara amfani da wata dabara domin hana daukar ciki**<br><br>**'Ya'ya nawa kike dasu masu rai a wancan lokacin, idan da akwai?**<br><br>**'Ya'ya maza nawa?**<br>**Kuma 'ya 'ya mata nawa?**<br><br>IF NONE, RECORD '00'.	NUMBER OF CHILDREN . . . . . ☐☐<br>NONE…………………………………..00 → **GO TO**<br><br>NUMBER OF BOYS ☐☐<br><br>NUMBER OF GIRLS ☐☐	**Q309a**
Q309a	CHECK 302:<br>EVER USED IMPLANT (06 = 1 "YES") ☐ ↓<br><br>NEVER USED IMPLANT (06 = 2 "NO") ☐ →		**Q310**
Q309b	I would like to ask a few questions about your experience with implant use. Was the implant you used the last time one rod or two rods?<br><br>***Ina son in yi maki wasu yan tambayoyi gameda sanin yadda ake amfani da karafan da ake sawa a dantse. Shi kananan karafun ada aka sa maki a dantse na karshe daya ne ko biyu?***	ONE ROD……………………………...……..……1<br>TWO RODS…..………………..……………..…….2<br>DON'T KNOW……………………………..……..…8	

Q309c	At the time of implant insertion, were you told by the health care provider when and where to go for removal of the implant?<br><br>**A lokacin da za'a sa maki karafan a dantsen ki, jamian kiwon lafiya sun gaya maki lokaci da inda zaki je a cire wadannan karafan?**	YES, TOLD WHERE TO GO FOR REMOVAL ………1<br>YES, TOLD WHEN TO GO FOR REMOVAL………...2<br>YES, TOLD BOTH WHEN AND WHERE TO GO FOR REMOVAL………………………………………………3<br>NO, NOT TOLD WHEN OR WHERE TO GO FOR REMOVAL……………………………………………...4<br>DON'T REMEMBER……………………………………..8	
Q310	CHECK Q233 CURRENTLY PREGNANT<br><br>NOT CURRENTLY PREGNANT (Q233=2 OR =8) ☐ ↓	CURRENTLY PREGNANT (Q233=1) ☐	**Q313**
Q311	Are you (or your partner) **currently** doing something or using any method to delay or avoid getting pregnant?<br><br>***Shin ke ko (abokin zaman ki) na yin wani abu ko amafani da wata dabara yanzu don jinkirta ko gujewa daukan ciki?***<br><br>IF RESPONDENT OR RESPONDENT'S PARTNER HAS EVER BEEN STERILIZED, CIRCLE "YES" (CODE=1)	YES……………………………………………………1<br>NO……………………………………………………2<br>SAYS SHE CAN'T GET PREGNANT………..3	 **Q313**<br>**Q313**
Q312	Which method(s) are you (or your partner) currently using?<br><br>***Wacce irin dabara ke* (ko abokin zaman ki) ke *amfani da shi yanzu?***<br><br>MULTIPLE METHODS – CIRCLE ALL MENTIONED<br><br>IF RESPONDENT SAYS "PILL", PROBE FURTHER TO ESTABLISH IF THEY MEAN THE "DAILY PILL" OR THE "EMERGENCY PILL"	FEMALE STERILIZATION……………………...A<br>MALE STERILIZATION…………………...........B<br>IMPLANT……………………………………...........C<br>IUD…………………………………………………D<br>INJECTABLE... . . . . ………..…………..E<br>DAILY PILL………………………………………F<br>EMERGENCY PILL (Postnor2, etc.)…………..G<br>MALE CONDOM . . . .……….................H<br>FEMALE CONDOM . .. . ..… . . ………….........I<br>STANDARD DAYS METHOD/<br>CYCLE BEADS…………………………………..J<br>BREASTFEEDING/LAM . . . . . …..……………..K<br>OTHER MODERN METHOD<br>_______________________________ L<br>(SPECIFY)<br>RHYTHM METHOD ……..……………………….M<br>WITHDRAWAL ………..……………….............N<br>OTHER TRADITIONAL METHOD<br>_______________________________X<br>(SPECIFY)	
Q313	CHECK Q312:<br><br>CIRCLE METHOD CODE:<br><br>IF MORE THAN ONE METHOD CODE CIRCLED IN Q312, CIRCLE CODE FOR HIGHEST METHOD IN LIST	NO CODE CIRCLED…………………………………00<br>FEMALE STERILIZATION…………………………..01<br>MALE STERILIZATION……………………………...02<br>IMPLANT……………………………………………...03<br>IUD……………………………………………………04<br>INJECTABLE... . . . . ……………………….05<br>DAILY PILL ……………………………………..…06<br>EMERGENCY PILL (Postnor2, etc.)……………….07<br>MALE CONDOM . . . .…………………......08<br>FEMALE CONDOM . .. . . …. . . ……………….…09<br>STANDARD DAYS METHOD /CYCLE BEADS………………………………………….........10<br>BREASTFEEDING / LAM ....11<br>OTHER MODERN METHOD………………………..12<br>RHYTHM METHOD ………………………….……13<br>WITHDRAWAL ……………………….....................14<br>OTHER TRADITIONAL METHOD…………………..15	**Q317**
Q314	Since what month and year have you been using [CURRENT METHOD IN Q313] without stopping?<br><br>***Tun wanne wata da shekara ki ke amfani da wannan hanyar ba tare da kin tsaya ba?***	MONTH…………….[__|__]<br><br>YEAR………..[__|__|__|__]	

Q315	CHECK Q314, Q215, Q251(01)- Q251(03)<br><br>ANY BIRTH OR PREGNANCY TERMINATION AFTER MONTH AND YEAR OF START OF USE OF CONTRACEPTION IN 314?<br>YES ☐ NO ☐<br><br>**<u>FOR METHODS OTHER THAN MALESTERILIZATION:</u>** GO BACK TO 314, PROBE AND RECORD MONTH AND YEAR AT START OF CONTINUOUS USE OF CURRENT METHOD (MUST BE AFTER LAST BIRTH OR PREGNANCY TERMINATION)<br><br>**<u>FOR MALE STERILIZATION</u>**: GO BACK TO 314, PROBE AND RECORD MONTH AND YEAR OF STERILIZATION	

Q316<br><br>**C**	CHECK Q314:<br><br>YEAR IS JANUARY 2012 OR LATER ☐<br><br>ENTER CODE FOR METHOD USED IN MONTH OF INTERVIEW IN **<u>COLUMN 1</u>** OF THE CALENDAR AND IN EACH MONTH BACK TO THE DATE STARTED USING METHOD<br><br>**ENTER METHOD SOURCE CODE IN COLUMN 2 OF THE CALENDAR IN THE MONTH SHE STARTED USING**<br><br>THEN CONTINUE WITH Q317	YEAR IS EARLIER THAN 2012 ☐<br><br>ENTER CODE FOR METHOD USED IN MONTH OF INTERVIEW IN **<u>COLUMN 1</u>** OF THE CALENDAR AND EACH MONTH BACK TO JANUARY 2012<br><br>ENTER METHOD SOURCE CODE IN JANUARY 2012 CELL OF COLUMN 2<br><br>THEN SKIP TO →	**Q318**

Q317<br><br>**C**	I would like to ask you some questions about the times you or your partner may have used a method to avoid getting pregnant since January 2012.<br><br>**Yanzu ina so in tambayeki game da lokutan da ke ko abokin zamanki ku ka taba amfani da wata dabara domin hana daukar ciki tun daga Janaury 2012?**<br><br>USE CALENDAR TO PROBE FOR EARLIER PERIODS OF USE AND NONUSE, STARTING WITH MOST RECENT USE, BACK TO JANUARY 2012.<br>USE NAMES OF CHILDREN, DATES OF BIRTH, AND PERIODS OF PREGNANCY AS REFERENCE POINTS.<br><br>**IN COLUMN 1**, ENTER METHOD USE CODE OR '0' FOR NONUSE IN EACH BLANK MONTH<br><br>ILLUSTRATIVE QUESTIONS:<br>• When was the last time you used a method? Which method was that?<br>**Yaushe ne lokacin karshe da ki ka yi amfani da wata dabarar tsarin iyali? Wacce irin dabara ce?**<br><br>• When did you start using that method? How long after the birth of (NAME)?<br>**Yaushe ki ka fara amfani da wannan dabarar? Har tsawon wane lokaci ne bayan haihuwar (NAME)**<br><br>• How long did you use the method then?<br>**Har tsawon wane lokacin ne ki ka yi amfani da wannan dabarar?**<br><br>**IN COLUMN 2,** ENTER THE CODE FOR THE SOURCE OF THE METHOD IN ONLY THE FIRST MONTH OF EACH USE. FOR RESPONDENTS WHO HAVE MULTIPLE PERIODS OF USE, ASK FOR THE SOURCE OF EACH PERIOD.<br><br>ILLUSTRATIVE QUESTIONS:<br>• Where did you get the method when you started using it?<br>A wanne wurin kika samu wannan dabarar lokacin da kika fara amfani da ita?<br>• Where did you get advice on how to use the method [for rhythm or withdrawal]?<br>Wanne wurin kika je domin samun shawarar yadda zakiyi amfani da[juriyar rashin yin jima'i ko zare kan azzakari kafin fitowar maniyi]<br><br>**IN COLUMN 3,** ENTER CODES FOR DISCONTINUATION NEXT TO THE LAST MONTH OF USE. NUMBER OF CODES IN COLUMN 3 MUST BE SAME AS NUMBER OF INTERRUPTIONS OF METHOD USE IN COLUMN 1.<br><br>ASK WHY SHE STOPPED USING THE METHOD. IF A PREGNANCY FOLLOWED, ASK WHETHER SHE BECAME PREGNANT UNINTENTIONALLY WHILE USING THE METHOD OR DELIBERATELY STOPPED TO GET PREGNANT.<br><br>ILLUSTRATIVE QUESTIONS:<br>• Why did you stop using the (METHOD)? **Don me ki ka daina amfani da wannan (METHOD)?**<br><br>• Did you become pregnant while using (METHOD), or did you stop to get pregnant, or did you stop for some other reason?<br>**Shin Kin dauki ciki ne da ki ke amfani da (METHOD), ko kin daina ne domin ki dauki ciki, kokuma kin daina ne domin wasu dalilai?**<br><br>IF DELIBERATELY STOPPED TO BECOME PREGNANT, ASK:<br>• How many months did it take you to get pregnant after you stopped using (METHOD)? AND ENTER '0' IN EACH SUCH MONTH IN COLUMN 1.<br>**Har tsawon wata nawa ya dauka kafin ki ka samu ciki bayan kin daina amfani da (METHOD)**	

Q318	CHECK Q313:<br>CIRCLE METHOD CODE:	NO CODE CIRCLED………………………….. 00<br>FEMALE STERILIZATION…………………….. 01<br>MALE STERILIZATION……………….............. 02<br>IMPLANT…………………………….............. 03<br>IUCD……………………………………………. 04<br>INJECTABLE... . . . . ……….…………. 05<br>DAILY PILL…………………………………… 06<br>EMERGENCY PILL (Postnor2, etc.)………… 07<br>MALE CONDOM . . . . ………............... 08<br>FEMALE CONDOM . .. . ..… . . ……….…...... 09<br>STANDARD DAYS METHOD/<br>CYCLE BEADS…..………………………… 10<br>BREASTFEEDING/LAM . . . . . …..………….. 11<br>OTHER MODERN METHOD………………….. 12<br>RHYTHM METHOD ……..…………………… 13<br>WITHDRAWAL ………..……..……….............. 14<br>OTHER TRADITIONAL METHOD……..…….. 15	00 → **Q334**
Q319	Who decided which type of family planning/ child birth spacing/child spacing method to use? Is it mainly your decision, mainly your partner's decision, or did you both decide together?<br><br>***Shin wa ya ke yanke shawara wajen wacce irin dabarar tsarin iyali/tazara tsakanin haihuwa/tazara tsakanin yara za ku yi amfani da shi? Shin ra' ayin ki ne ke kadai, ko na mijin ki /abokin zaman ki, ko ra'ayin ku ne tare ?***	MAINLY YOU..……….……………….……1<br>MAINLY PARTNER………..………………2<br>JOINTLY…………………….………………3<br>OTHER___________________________6<br>(SPECIFY)	
Q320	Were you ever told by a health or family planning worker about side effects or problems you might have using this family planning method [CIRCLED IN Q318]?<br><br>***Shin ko jami'in kiwon lafiya ko na tsarin iyali sun gaya miki abin da za ki yi idan illolin ko idan matsaloli sun faru da wannan dabarar tsarin iyalin?***	YES . . . .………. . . . . . . ………... 1<br>NO . . . . …………………. 2<br>DON'T KNOW . . . . ………… ……….. 8	2 → **Q322**<br>8 → **Q322**
Q321	Were you told by a health or family planning worker what to do if you experienced side effects or problems with this method [CIRCLED IN Q318]?<br><br>*Shin ko jami'in kiwon lafiya ko na tsarin iyali sun gaya miki abin da za ki yi idan illolin ko idan matsaloli sun faru da wannan dabarar tsarin iyalin?* [CIRCLED IN Q318]?	YES . . . .…………... . . .1<br>NO . . . .………….. . 2<br>DON'T KNOW/CAN'T REMEMBER. .. ..…. .8	
Q322	Were you ever told by a health or family planning worker about **<u>other</u>** methods of family planning (beside the one you are currently using)?<br><br>***Shin ko jami'an kiwon lafiya ko na tsarin iyali sun taba gaya miki wasu dabarun tsarin iyali/tazara tsakanin haihuwa/tazara tsakanin yara, da za ki iya amfani da su (banda wanda ki ke amfani dashi yanzu)***	YES . . . . …..……….. . . . . . .1<br>NO . . . . …………... . . . . .2<br>DON'T KNOW . . . . …………... 8	

Q323	How many living children did you have when you began using [CURRENT METHOD CIRCLED IN Q318] if any?<br><br>**'Ya'yan ki nawa ke raye lokacin da kika fara amfani da [CURRENT METHOD CIRCLED IN Q317] idan akwai?**<br><br>IF NONE, RECORD 00.<br><br>How many sons? **Maza nawa ne?**<br><br>How many daughters? **Mata nawa ne**?	NUMBER OF CHILDREN…………..[__|__]<br>NONE………………………………00 → GO TO<br><br>SONS ……………………………… .[__|__]<br>DAUGHTERS ……………………… [__|__]	**Q324**
Q324	CHECK Q318:<br><br>IF CIRCLED FEMALE STERILIZATION "01" MALE STERILIZATION "02", IMPLANT "03", IUD "04", INJECTABLE "05", DAILY PILL "06", EMERGENCY PILL "07", MALE CONDOM "08", FEMALE CONDOM "09", STANDARD DAYS/SAFE DAYS/CYCLE BEADS "10", OR OTHER MODERN METHOD "12" ☐ ↓	IF CIRCLED BREASTFEEDING/LAM "11", RHYTHM METHOD "13" WITHDRAWAL"14" OR OTHER TRADITIONAL METHOD"15" ☐ →	**Q329**
Q325	From where or whom did you obtain [CURRENT METHOD] last time?<br><br>**Daga wanne wuri ko wajen wanene ki ka samu [CURRENT METHOD] [CURRENT METHOD] zuwan ki karshe?**<br><br>PROBE: What is the name of this place/person? And where is it located?<br><br>**PROBE: Menene sunan wannan wurin/ ma'aikacin? Kuma a ina wurin yake?**	NAME OF FACILITY /PERSON<br>________________________________<br>________________________________<br><br>*CODE BOXES: OFFICE ONLY* [__|__|__|__|__|__]<br><br>STREET NAME/ADDRESS____________<br>________________________________<br>LAND MARK DESCRIPTION_____________________	

Q325a.	What type of place/person is this?<br><br>***Wanne irin waje ko mutun ne?***<br><br>SINGLE MENTION.	**PUBLIC SECTOR**<br>GOVT HOSPITAL…………………………11<br>WOMEN AND CHILDREN'S HOSPITAL……………………………….12<br>CHILD WELFARE CLINIC………….….13<br>GOVT. HEALTH CENTER………….……14<br>GOVERNMENT POST/DISPENSARY.....15<br>MATERNITY HOME…………..……..…16<br>MOBILE CLINIC………..……………….17<br><br>OTHER PUBLIC______________________18<br>(SPECIFY)<br>**PRIVATESECTOR**<br>PRIVATE HOSPITAL/CLINIC..................21<br>PRIVATE DOCTOR'S OFFICE................22<br>NURSING/MATERNITY HOME…………23<br>PHARMACY………..…………….....…24<br>PMS/CHEMIST……………………...…....25<br>MOBILE CLINIC……………….……….......26<br>CHW/TBA………………….….…………27<br>TRADITIONAL HEALER…………………..28<br><br>OTHER PRIVATE____________________29<br>(SPECIFY)<br>**FAITH-BASED SECTOR**<br>MISSION HOSPITAL………….................31<br>FAITH-BASED, CHURCH CLINIC…........32<br>**OTHER SOURCE**<br>OTHER NGO HOSPITAL/CLINIC.......….41<br>WORKSITE CLINIC…………......……….42<br>YOUTH CENTER…….………..…………43<br>VENDING MACHINE/DISPENSER…........………44<br>VCT…..……………………………..........45<br>BAR………………..…………… …..……..46<br>KIOSK/SHOP/MARKET…………… …..47<br><br>OTHER ____________________________96<br>(SPECIFY)<br>DON'T KNOW . . . . …..………. 98	
Q326	CHECK Q318:<br><br>IF CIRCLED DAILY PILL "06" ☐ ↓	IF CIRCLED FEMALE STERILIZATION "01", MALE STERILIZATION "02", IMPLANT "03", IUD "04", INJECTABLE "05", EMERGENCY PILL "07", MALE CONDOM "08",<br>FEMALE CONDOM "09", STANDARD DAYS/SAFE DAYS/CYCLE BEADS "10",<br>OTHER MODERN METHOD "12" ☐ →	**Q328**
Q327	FOR DAILY PILL USERS ONLY: How many (pill cycles) did you buy/get the last time?<br><br>**FOR DAILY PILL USERS ONLY: Kwayoyi guda nawa ki ka saya ko ki ka samu a lokacin *zuwan ki na karshe*?**	NUMBER OF PILL CYCLES ☐☐<br><br>DON'T KNOW…………….98	
Q328	What price did you pay for this current method [CIRCLED IN Q318]?<br><br>PLEASE INCLUDE TOTAL COST FOR ALL SERVICES RELATED TO THE METHOD AS WELL AS CONSUMABLES<br><br>**Nawa ki ka biya a wannan dabarar da ki ke amfani da ita yanzu? [CIRCLED IN Q318]?**	AMOUNT (in NAIRA) ☐☐☐☐☐<br><br>FREE…………………………………...……00000<br>DON'T KNOW…………………………….…99998	

Q329	Before you started using [CURRENT METHOD] did you (or your partner) use a different method?<br><br>**Kafin ku ka fara amfani da [CURRENT METHOD] ke ko abokin zaman ki kun yi amfani da wata hanyar tsarin iyali?**	YES . . . . 1<br><br>NO . . . . 2 →	**Q335**

Q330	Which method were you using before the [CURRENT METHOD]?<br><br>***Wacce irin hanyar tsarin iyali ku ke amfani da ita kafin* [CURRENT METHOD]?**<br><br>IF MULTIPLE METHODS MENTIONED, CIRCLE THE HIGHEST METHOD ON THE LIST AND PROCEED WITH QUESTIONS REGARDING THIS METHOD.<br><br>IF RESPONDENT SAYS "PILL", PROBE FURTHER TO ESTABLISH IF THEY MEAN THE "DAILY PILL" OR THE "EMERGENCY PILL"	FEMALE STERILIZATION……………………..01<br>MALE STERILIZATION……………….............02<br>IMPLANT…………………………….............03<br>IUD…..…………………………………………04<br>INJECTABLE... . . . . ……..………….05<br>DAILY PILL……….……………………………06<br>EMERGENCY PILL (Postnor2, etc.)…………..07<br>MALE CONDOM . . . . ………….............08<br>FEMALE CONDOM . .. . ..…. . . ……….........09<br>STANDARD DAYS METHOD/<br>CYCLE BEADS…..………………………………10<br>BREASTFEEDING/LAM . . . . . ….…………..11<br>OTHER MODERN METHOD<br>________________________________ 12<br>(SPECIFY)<br>RHYTHM METHOD …….……………………. 13<br>WITHDRAWAL ………..……………….......... 14<br>OTHER TRADITIONAL METHOD<br>________________________________ 15<br>(SPECIFY)	
Q331	How many living children did you have when you began using [PREVIOUS METHOD], if any?<br><br>**'Ya'yan ki nawa ke raye lokacin da kika fara amfani da [PREVIOUS METHOD], Idan akwai?**<br><br>IF NONE, RECORD 00.<br><br>How many sons? **Maza nawa ne?**<br><br>How many daughters? **Mata nawa ne?**	NUMBER OF CHILDREN………..[__|__]<br><br>NONE……………………………….00 → **GO TO**<br><br>SONS ………………………………[__|__]<br><br>DAUGHTERS ………………………[__|__]	**Q332**
Q332	How long did you use the last method [CIRCLED IN Q330]?<br><br>**Har tsawon wanne lokaci ki ka yi amfani da dabarar hanyar tsarin iyali na karshe CIRCLED IN Q330]?**<br><br>IF RESPONSE IS A DECIMAL FOR MONTHS & YEARS (2.5YRS), THEN CONVERT IN MONTH AND FILL IN ONLY MONTHS	1 [__|__] DAYS<br>OR<br>2 [__|__] WEEKS<br>OR<br>3 [__|__] MONTHS<br>OR<br>4 [__|__] YEARS<br>UNSURE/CAN'T REMEMBER…………998	
Q333	Why did you stop using the method [CIRCLED IN Q330]?<br><br>**Wanne dalili ya sa ki ka daina amfani da wannan dabarar *tsarin iyalin* [CIRCLED IN Q330]?**<br><br>MULTIPLE RESPONSE - CIRCLE ALL MENTIONED.	WANTED TO GET PREGNANT…………... A<br>METHOD FAILED/GOT PREGNANT……... B<br>LACK OF SEXUAL URGE…………………. C<br>FEAR OF BECOMING INFERTILE………… D<br>CREATED MENSTRUAL PROBLEM……...E<br>CREATED HEALTH PROBLEM…………… F<br>INFREQUENT SEX/NO SEX……………… G<br>INCONVENIENT TO USE………………….. H<br>HARD TO GET………………. ……………. I<br>GAINED WEIGHT…………………………... J<br>LOST WEIGHT……………………………… K<br>COSTS TOO MUCH………………………….L<br>DID NOT LIKE METHOD…………………… M<br>LACK OF PRIVACY…………………………. N<br>SPOUSE/PARTNER DID NOT APPROVE...O<br>MENOPAUSE/HYSTERECTOMY…………. P<br>HEALTH PROVIDER ADVISED TO STOP...Q<br>METHOD NOT AVAILABLE……………….. R<br>OTHER____________________________ X<br>(SPECIFY)	**All skip to Q335**

Q334	What are the main reasons why you are not **currently** using a method of family planning/ child birth spacing to delay or avoid pregnancy?<br><br>***Shin wanne dalili ne yasa ba kya amfani da wata dabarar tsarin iyali/tazara tsakanin haihuha/tazara tsakanin yara don jinkirta ko hana daukan ciki yanzu?***<br><br>*CIRCLE ALL MENTIONED*<br><br>PROBE: Any other? **Akwai wani?**	**FERTILITY RELATED REASONS**:<br>A. NO SEX<br>B. INFREQUENT SEX<br>C. NOT MARRIED YET/ NO PARTNER<br>D. AWAY FROM SPOUSE<br>E. ALREADY PREGNANT<br>F. BREASTFEEDING<br>G. RECENTLY HAD BABY<br>H. WANTS MORE CHILDREN/TRYING TO GET PREGNANT<br>I. MENOPAUSAL/HYSTERECTOMY →<br>J. CAN'T HAVE (MORE) CHILDREN →<br><br>**OPPOSITION TO USE:**<br>K. RESPONDENT OPPOSES<br>L. PARTNER OPPOSES<br>M. OTHERS OPPOSE<br>N. RELIGIOUS PROHIBITION<br><br>**LACK OF KNOWLEDGE**:<br>O. KNOWS NO METHOD<br>P. DOESN'T KNOW WHICH METHOD TO USE<br>Q. DON'T KNOW HOW TO USE METHOD<br>R. KNOWS NO SOURCE<br><br>**METHOD-RELATED REASONS**:<br>S. HEALTH CONCERNS<br>T. FEAR OF SIDE EFFECTS<br>U. LACK OF ACCESS/TOO FAR<br>V. COSTS TOO MUCH<br>W. INCONVENIENT TO USE<br>X. DON'T LIKE EXISTING METHODS<br>Y. BAD EXPERIENCE WITH EXISTING METHODS<br><br>**FATALISTIC**:<br>Z. UP TO GOD<br><br>**OTHER**:<br>WW. OTHER ___________________________<br>XX. OTHER ___________________________<br>YY. OTHER ___________________________<br>ZZ. DON'T KNOW	 <br><br><br><br><br><br><br><br>**Q339**<br>**Q339**

Q335	CHECK Q318<br><br>NO CODE CIRCLED, IMPLANT, IUD, INJECTABLE, DAILY PILL, EMERGENCY PILL, MALE CONDOM, FEMALE CONDOM, SDM, LAM, OTHER MODERN METHOD, RHYTHM METHOD, WITHDRAWAL, OTHER TRADITIONAL METHOD CIRCLED (0, 03-15) ☐ ↓	FEMALE STERILIZATION OR MALE STERILIZATION CIRCLED (01,02) ☐ →	**Q339**
Q336	Do you or your partner intend to use/continue to use a method to delay or avoid pregnancy within the next twelve (12) months?<br><br>***Shin ke ko mijinki/abokin zaman ki, na da niyyar amfani/cigaba da wata dabarar tsarin iyalin don jinkirta ko gujewa daukan ciki nan da watanni goma sha biyu (12)?***	YES . . . . ….…....1<br>NO . . . . ….…....2 →<br>DON'T KNOW . . . . ….…....8 →	 **Q339**<br>**Q339**
Q337	What method would you prefer MOST to use, if you do use a method in the future?<br><br>***Wacce irin dabara za ki fi son yin amfani da ita, in har za ki yi amfani da wata dabara nan gaba?***<br><br>SINGLE MENTION	FEMALE STERILIZATION…………………….....01<br>MALE STERILIZATION……………….................02<br>IMPLANT……………………………................03<br>IUD…..………………………………………….04<br>INJECTABLE... . . . . ……...……….…...05<br>DAILY PILL………………………………….…..06<br>EMERGENCY PILL (Postnor2, etc.)…………....07<br>MALE CONDOM . . . . ……….................08<br>FEMALE CONDOM . .. . ..… . . ………….........09<br>STANDARD DAYS METHOD/<br>CYCLE BEADS…..……………..…………………....10<br>BREASTFEEDING/LAM . . . . . …..……………11 →<br>OTHER MODERN METHOD<br>______________________________ 12<br>(SPECIFY)<br>RHYTHM METHOD ……..……………………. 13 }<br>WITHDRAWAL ………...………………......... .. 14 } →<br>OTHER TRADITIONAL METHOD<br>______________________________ 15<br>(SPECIFY)<br>DON'T KNOW………………………………….…98 →	**Q339**<br><br>**Q339**<br><br>**Q339**
Q338	Do you know of a place where or person from whom you can obtain this method?<br><br>***Kin san wani wuri ko mutumin da za ki iya samun wannan dabarar tsarin iyalin?***	YES……………………………………….…. 1<br>NO…………………………………….……. 2	
Q339	Do you approve of using family planning?<br><br>***Kin amince da amfani da dabarar tsarin iyali?***	YES………………………………………………1<br>NO…………………………………………………2<br>DON'T KNOW……………………………………8	

Quality of Care – Services and Treatment					
Please tell me how you would agree or disagree with the following statements. If you say you disagree, I will ask you to tell me if you strongly disagree or just disagree. If you say you agree, I will ask you to tell me if you strongly agree or just agree.<br><br>***Ina son ki gaya mani ko kin yarda, ko ba ki yarda ba, da wadannan maganganu. Idan ba ki yarda ba, zan tambayeki, baki yarda ba sosai, ko kuma baki yarda ba ne kawai. Idan kin yarda, zan tambayeki, ko kin yarda kwarai ne ko kuma kin yarda kawai.***		Strongly Agree<br><br>**Kin yarda sosai**	Agree<br><br>**Kin yarda kawai**	Disagree<br><br>**Ba ki yarda ba kawai**	Strongly Disagree<br><br>**Ba ki yarda ba sosai**
Q340	Family planning providers around here treat clients very badly.<br><br>***Jami'ai masu bayar da dabarun tsarin iyali a kewayen nan suna musgunawa wadanda suke zuwa wajensu***	4	3	2	1

Q341	Women don't like the way they are treated in family planning clinics around here.<br><br>***Mata basu son yadda ake lura da su a asibitin tsarin iyali a kewayen nan***	4	3	2	1
Q342	Family planning sellers/providers make women like you feel bad when obtaining contraceptives.<br><br>***Jami'ai masu sayar/bayar da dabarun tsarin iyali suna musgunawa mata irin ki a lokacin da suke karban dabarar tsarin iyali?***	4	3	2	1
Q343	Women in this community believe that FP providers in this community are knowledgeable<br><br>***Matan da ke wannan al'ummar sun yarda da cewa Jami'an tsarin iyali na da ilimi matuka akan aikin su*.**	4	3	2	1

Beliefs					
Please tell me how you would agree or disagree with the following statements. If you say you disagree, I will ask you to tell me if you strongly disagree or just disagree. If you say you agree, I will ask you to tell me if you strongly agree or just agree.<br><br>***Ina so ki gaya mani ko zaki yarda, ko ba zaki yarda ba, da wadannan maganganu. Idan ba ki yarda ba, zan tambayeki, baki yarda ba ne sosai, ko kuma baki yarda ba ne kawai. Idan kuma kin yarda, zan tambayeki, ko kin yarda ne sosai ko kuma kin yarda ne kawai.***		**Strongly Agree**<br><br>**Kin Yarda sosai**	**Agree**<br><br>**Kin Yarda kawai**	**Disagree**<br><br>**Ba ki yarda ba kawai**	**Strongly Disagree**<br><br>**Ba ki yarda ba sosai**
Q344	Use of a contraceptive injection can make a woman permanently infertile.<br><br>***Amfani da allurar tsarin iyali na iya hana mace haihuwa har abada***	4	3	2	1
Q345	People who use family planning end up with health problems.<br><br>***Mutane masu amfani da dabarun tsarin iyali suna karewa da matsalolin rashin lafiya***	4	3	2	1
Q346	Contraceptives can harm your womb.<br><br>***Dabarun tsarin iyali na iya kawo illa ga mahaifar mace***	4	3	2	1
Q347	Contraceptives reduce women's sexual urge.<br><br>***Dabarun tsarin iyali na rage shaáwar jima'i wa mata***	4	3	2	1
Q348	Contraceptives can cause cancer.<br><br>***Dabarun tsarin iyali na iya sa ciwon sankara/daji***	4	3	2	1
Q349	Contraceptives can give you deformed babies.<br><br>***Dabarun tsarin iyali zai iya sa haihuwar nakasasun 'ya'ya***	4	3	2	1
Q350	Contraceptives are dangerous to your health.<br><br>***Dabarun tsarin iyali na da illla ga lafiyar ki.***	4	3	2	1
Q351	Women who use family planning /child birth spacing may become promiscuous<br><br>***Matan da ke amfani da dabarun tsarin iyali/ tazara tsakanin haihuwa da haihuwa na iya zama harija?***	4	3	2	1
Q352	A man should accompany his wife to the health facility for family planning<br><br>***Miji na iya yi ma matarsa rakiya zuwa cibiyar kiwon lafiya domin samun dabarun tsarin iyali***	4	3	2	1

Section 4: MATERNAL AND CHILD HEALTH			
**Qno**	**Questions and filters**	**Coding categories**	**Skip to**
Q401	CHECK Q225:<br>ONE OR MORE BIRTHS SINCE JANUARY 2015 ☐ ↓	NO BIRTHS SINCE JANUARY 2015 ☐ →	**Q413**
Q402	ENTER NAME AND LINE NUMBER OF YOUNGEST CHILD BORN SINCE JANUARY 2015 FROM Q211 AND Q212:<br>________________ NAME [__|__] LINE NUMBER<br><br>Now I would like to ask you about the delivery of [NAME OF YOUNGEST CHILD].<br>***Yanzu ina so na tambayeki game da haihuwar ki ta karshe*** **[NAME OF YOUNGEST CHILD**].		
Q403	Who assisted with the delivery of [NAME]?<br>*(NAME OF CHILD)*<br>CIRCLE ALL MENTIONED.<br>**Wa ya taimaka maki wajen haihuwar [NAME]?**<br>***NAME OF CHILD)***	DOCTOR/ CLINICAL OFFICER ……………. A<br>NURSE/ MIDWIFE…………………………… B<br>TBA . . . . ……………………… C<br>COMMUNITY HEALTH WORKER …………. D<br>FRIEND/RELATIVE…………………………… E<br>OTHER____________________ X<br>(SPECIFY)<br>NO ONE……………………………………….. Y	
Q404	Was the place you delivered in this city, in another city, or in a rural area?<br>***Wajen da ki ka haihu a wannan birnin ne ko a wani birnin ne ko a kauye?***	THIS CITY/TOWN…………………………… 1<br>ANOTHER CITY OR TOWN……………….. 2<br>A RURAL AREA…………………………… 3	

Q405	Where did you give birth to [NAME]?<br><br>***A ina ki ka haifi [NAME]?***	**PUBLIC SECTOR**<br>GOVT HOSPITAL……………………………….11<br>WOMEN AND CHILDREN'S HOSPITAL……12<br>CHILD WELFARE CLINIC……………..............13<br>GOVT. HEALTH CENTER………..…………14<br>GOVERNMENT<br>POST/DISPENSARY…………………………..15<br>MATERNITY HOME……………………………16<br>MOBILE CLINIC……..………………….……..17<br>OTHER PUBLIC______________________18<br>(SPECIFY)<br>**PRIVATESECTOR**<br>PRIVATE HOSPITAL/CLINIC.......................21<br>PRIVATE DOCTOR'S OFFICE…………….22<br>NURSING/MATERNITY HOME……………23<br>MOBILE CLINIC…………….……….…...….26<br>CHW/TBA…………………………………….27<br>TRADITIONAL HEALER…………..…….28<br>OTHER PRIVATE__________________29<br>(SPECIFY)<br>**FAITH-BASED SECTOR**<br>MISSION HOSPITAL………….........31<br>FAITH-BASED, CHURCH CLINIC..……32<br>**OTHER SOURCE**<br>OTHER NGO HOSPITAL/CLINIC……….41<br>WORKSITE CLINIC………….…………42<br>YOUTH CENTER………………………….43<br>OTHER FACILITY ________________48<br>(SPECIFY)<br>AT HOME…………………………………..51 →<br>OTHER(specify) __________________96 →	**Q409**<br>**Q409**
Q405a	What is the name of this place? And where is it located?<br><br>**Menene sunan wannan wajen?**<br>**Kuma a ina yake?**	NAME OF<br>FACILITY________________________________<br>____<br>*CODE BOXES: OFFICE ONLY* [__|__|__|__|__]<br>STREET<br>NAME/ADDRESS________________________<br>____<br><br>LAND MARK<br>DESCRIPTION_____________________________<br>_____________________________________	
Q405b	What price did you pay for delivery services?<br><br>**Nawa kika biya lokacin haihuwar (SUNA)?**<br><br>PLEASE INCLUDE TOTAL COST FOR ALL SERVICES RELATED TO THE DELIVERY AS WELL AS CONSUMABLES	AMOUNT<br>(in NAIRA) [| | | |]<br><br>FREE…………………………………...……00000<br>DON'T KNOW…………….……………….…99998	
Q406	When you came to the facility for delivery, did anyone give you information or counsel you on family planning before you delivered?<br><br>***Lokacin da ki ka zo wannan cibiyar kiwon lafiyar domin haihuwa, shin akwai wanda yai maki bayani ko ya ba ki shawara kan hanyoyin tsarin iyali kafin ki haihu?***	YES……………….…………………….………1<br><br>NO……………………………………………..2	
Q407	After you had delivered, did anyone talk to you about using a family planning method postpartum before you left the health facility?<br><br>***Bayan haihuwar ki, ko akwai wanda ya yi maki magana kan hanyar tsarin iyali da ta shafi kula bayan haihuwa/lokacin jego kafin ki bar cibiyar kiwon lafiyar?***	YES……………………………………………1<br><br>NO……………………………………………..2	

Q407a	After you had delivered, did anyone talk to you about exclusive breastfeeding for contraceptive purposes?<br><br>**Bayan da kika haihu, akwai wanda yai maki magana kan shayar da nonon uwa kadai domin hana daukar ciki?**	YES……………………………………………1<br>NO……………………………………………..2	
Q408	Were you provided with any information or counseling about family planning/birth spacing methods from a health or family planning worker either before you delivered or after?<br><br>***Shin kafin ki haihu ko bayan kin haihu , ko akwai wani ma'aikacin /jami'an kiwon lafiya ko na tsarin iyali da suka yi maki bayani ko aka baki shawara akan tsarin iyali/tazara tsakanin haihuwa/tazara tsakanin yara ?***	YES, BEFORE DELIVERY………………….1<br>YES, AFTER DELIVERY…………………… 2<br>YES, AT BOTH TIMES…………………….. 3<br>NO, NOT AT ALL…………………………… 4<br>DON'T KNOW/REMEMBER……………….. . 8	→ **Q410**
Q409	Why didn't you deliver in a health facility?<br><br>CIRCLE ALL MENTIONED.<br><br>***Wanne dalilin ya sa ba ki haihu a cibiyar kiwon lafiya ba?***	COSTS TOO MUCH…………………………….A<br>FACILITY NOT OPEN………………………….B<br>DIDN'T HAVE TIME/LABOUR PAINS CAME EARLY…………………………....................C<br>DON'T TRUST THE STAFF………………….…..D<br>NOT NECESSARY……………………………...E<br>TRADITION……….……………………….….F<br>TOO FAR………………………………………..G<br>NO TRANSPORT AVAILABLE………………...H<br>NO ONE AVAILABLE TO ACCOMPANY HER ……………………………………….…..I<br>POOR QUALITY SERVICES…………….…....J<br>DO NOT OFFER SERVICES REQUIRED……K<br>PROVIDERS OFTEN AWAY…………………..L<br>DOES NOT ACCEPT INSURANCE…………..M<br>NO FEMALE PROVIDER………………………N<br>PARTNER/FAMILY WON'T ALLOW…………O<br>HUSBAND/SPOUSE NOT AT HOME…………P<br>DIDN'T HAVE MONEY……………………….....Q<br>POOR PROVIDER ATTITUDE…………………R<br>POOR FACILITY ENVIRONMENT……..……..S<br>FEAR OF HIV TESTING ………………………..T<br><br>OTHER________________________________ X<br>(SPECIFY)	
Q410	Within 12 months of delivery of [NAME ABOVE], did you start using a family planning method?<br><br>***A cikin watanni sha biyu (12) da ki ka haifi [NAME ABOVE], kin fara amfani da dabarar tsarin iyali?***	YES…………………………..……….…….1<br><br>NO…………………………………….…….2 →	**Q412a**

Q411	Which method(s)?<br><br>***Wacce irin dabara (ko dabaru)?***<br><br>PROBE: Any others?<br>***Akwai wata kuma?***	FEMALE STERILIZATION……………………..A<br>MALE STERILIZATION……………….............B<br>IMPLANT……………………………….............C<br>IUD…………………………………………………D<br>INJECTABLE... . . . . ……....…………..E<br>DAILY PILL………………………………………F<br>EMERGENCY PILL (Postnor2, etc.)…………..G<br>MALE CONDOM . . . . ………….............H<br>FEMALE CONDOM . .. . ….. ... . ……………........I<br>STANDARD DAYS METHOD/<br>CYCLE BEADS…..……….…………………..J<br>BREASTFEEDING/LAM . . . . . …..…………..K<br>OTHER MODERN METHOD<br>____________________________________ L<br>(SPECIFY)<br>RHYTHM METHOD ……………………………M<br>WITHDRAWAL ………...…………….............N<br>OTHER TRADITIONAL METHOD<br>____________________________________X<br>(SPECIFY)	
Q412	When did you start using the method (how long after delivery of ________[NAME ABOVE]<br><br>***Yaushe ki ka fara amfani da wannan dabarar (Tun yaushe bayan haihuwar________[NAME ABOVE***<br><br>RECORD COMPLETED DAYS IF LESS THAN 1 WEEK; COMPLETED WEEKS IF MORE THAN 7 DAYS AND LESS THAN 1 MONTH; COMPLETED MONTHS IF 1 MONTH OR MORE	DAYS POST PARTUM……………..1 [__|__]<br><br>WEEKS POST PARTUM……….…..2 [__|__]<br><br>MONTHS POST PARTUM…………3 [__|__]<br><br>IMMEDIATELY POST PARTUM/TIME OF DELIVERY…………………………….. 993	
Q412a	Now I'm going to ask you a question about [NAME ABOVE]'s health.<br>Yanzu ina son in yi maki tambaya gamedakiwon lafiyar [NAME ABOVE] da muke magana akai?.<br><br>In the last 24 hours, has [NAME ABOVE] had three or more loose or watery stools?<br><br>A cikin awa ashirin da hutu (24) da ta wusce, ko [NAME ABOVE] yayi gudawa mai ruwa-ruwa?	YES……………………………………………1<br>NO……………………………………………..2	
Q412b	Has [NAME ABOVE] been ill with a fever in the last 24 hours?<br><br>Ko [NAME ABOVE] yayi zazzabi a cikin awa ashirin da hudu (24) da suka shige?	YES……………………………………………1<br>NO……………………………………………..2	
Q412c	Did [NAME ABOVE] take any Malaria treatment in the past 14 days (2 weeks)?<br><br>Ko [NAME ABOVE] ya/ta karbi maganin zazzabin cizon sauro a cikin kwanaki goma sha hudu (14) watau sati biyu (2) da suka shige?	YES……………………………………………1<br>NO……………………………………………..2	
Q412d	Did [NAME ABOVE] sleep last night under a mosquito net?<br><br>Shin [NAME ABOVE] ya/ta kwana a gidan sauro daren jiya?	YES……………………………………………1<br>NO……………………………………………..2	
Q413	CHECK BIRTH HISTORY (Q216 & Q219) FOR ANY LIVE CHILDREN<br><br>YES, HAS ONE OR MORE LIVING CHILD ☐ ↓	NO, DOES NOT HAVE LIVING CHILDREN ☐ →	**Q419**
Now I am going to ask you questions about your experience seeking and receiving health services.<br><br>***Yanzu ina son in tambaye ki gameda yadda ki ke nema da kuma samun sha'anin kiwon lafiya.***			

Q414	In the <u>last three months</u>, have you gone to a health facility for any child health services?<br><br>***Cikin watanni ukku (3) da suka shige kunje asibiti don kiwon lafiyan yaro/yarinya?***	YES . . . .……..…………..….1<br><br>NO . . . .…...…….....………2 →	**Q419**
Q415	What types of services did you receive during this/these child health visit(s)?<br><br>***Wadanne irin abubuwa/kula aka yi ma ki lokacin/lokutan da ki ka je cibiyar kiwon lafiya da yaron/yarinyar?***<br><br>CIRCLE ALL MENTIONED.	IMMUNIZATION……………………………..... ………….. A<br>DISEASE PREVENTION………………….…. …………… B<br>DEWORMING………………………………………………….. C<br>TREATMENT FOR CHILD (FOR DIARRHEA,<br>MALARIA, OR RESPIRATORY INFECTION).................... D<br>GROWTH MONITORING OF CHILD………………………… E<br>HEALTH CHECK-UP . . . . …….. …..……………… F<br>OTHER______________________________________ X<br>(SPECIFY)	
Q415a	Where did you go for child services at your last visit?<br><br>PROBE: What is the name of this place? And where is it located?<br><br>***Ina ki ka je don nema lafiyan yaro/yarinya ki na karshe?***<br><br>**PROBE:** ***Menene sunan wurin? Kuma a ina wurin yake?***	NAME OF<br>FACILITY_________________________________<br>____<br>*CODE BOXES: OFFICE ONLY* [__|__|__|__|__]<br>STREET<br>NAME/ADDRESS____________________________<br>____<br><br>LAND MARK<br>DESCRIPTION____________________________	
Q416	What type of facility is this?<br><br>***Wanne irin asibiti ne?***	PUBLIC SECTOR<br>GOVERNMENT HOSPITAL…….……............................. 11<br>WOMEN AND CHILDREN'S HOSPITAL………………… 12<br>CHILD WELFARE CLINIC……………………………….. 13<br>GOVT. HEALTH CENTER……..……………………….. 14<br>GOVERNMENT POST/DISPENSARY…………………… 15<br>MATERNITY HOME…………………….…..…………... 16<br>MOBILE CLINIC…………………………….…………… 17<br>OTHER PUBLIC____________________________ 18<br>(SPECIFY)<br><br>PRIVATE SECTOR<br>PRIVATE HOSPITAL/CLINIC…………………………… 21<br>PRIVATE DOCTOR'S OFFICE………………………….. 22<br>NURSING/MATERNITY HOME……………….............. 23<br>PHARMACY…………………………….…………….….. 24<br>PMS/CHEMIST…………………..…………...…………… 25<br>MOBILE CLINIC……………………….…………………… 26<br>CHW/TBA…………………………………….…………… 27<br>TRADITIONAL HEALER……………………….................. 28<br>OTHER PRIVATE____________________________ 29<br>(SPECIFY)<br><br>FAITH-BASED SECTOR<br>MISSION HOSPITAL…………….……......................... 31<br>FAITH-BASED, CHURCH CLINIC……………................ 32<br><br>OTHER SOURCE<br>OTHER NGO<br>HOSPITAL/CLINIC………………….…………………... 41<br>WORKSITE CLINIC…………………………….………….. 42<br>YOUTH CENTER……………………………….................. 43<br>OTHER _____________________________________ 96<br>(SPECIFY)<br>DON'T KNOW . . . .………………..................... 98	

Q416a	What price did you pay for child health services at your last visit?<br><br>**Nawa kika biya da kika kai dan ki cibiyar kiwon lafiya a lokacin ziyarar karashe?**<br><br>PLEASE INCLUDE TOTAL COST FOR ALL SERVICES RELATED TO THE CHILD HEALTH VISIT AS WELL AS CONSUMABLES	AMOUNT (in NAIRA) [| | | |]<br><br>FREE…………………………………...……00000<br>DON'T KNOW……………………………..…99998	
Q417	Did you receive any information or counseling on family planning/child birth spacing during this visit?<br><br>***Shin kin samu wani bayani ko shawara akan tsarin iyali/tazara tsakanin haihuwa /tazara tsakanin yara a wannan lokacin ziyarar?***	YES . . . . ……………………………... . . . .….. 1<br><br>NO . . . . ……………………………….….. 2	
Q418	Did you receive a method or a referral for family planning/child birth spacing at that time?<br><br>***Shin kin samu dabara ko an tura ki wajen tsarin iyali/tazara tsakanin haihuwa/tazara tsakanin yara a wancan lokacin?***	YES, RECEIVED A METHOD. . . . . …..…. …………… 1<br>YES, RECEIVED A PRESCRIPTION………………………… 2<br>YES, RECEIVED A REFERRAL . . . . …………………… 3<br>NO, DID NOT RECEIVE ANY OF THESE……………………. 4	
Q419	In the <u>last year</u>, have you gone to a health facility for maternal health services, such as prenatal and postpartum care?<br><br>***Cikin shekarar da ta shige, kin je asibiti domin kiwon lafiya da ya shafi mata kamar awon ciki ko kuma kula bayan haihuwa/lokacin jego?***	YES . . . . ……………..…. . ………….1<br><br>NO . . . . ………..……………….2 →	**Q423**
Q419a	Where did you go most recently for maternal health services?<br>***Wanne wuri ki ka je kwanan nan domin samun kiwon lafiyar kafin ki haifu, lokacin da ki ke da juna biyu ko bayan ki haihu/lokacin jego?***<br><br>PROBE: What is the name of this place? And where is it located?<br><br>**PROBE: Menene sunan wurin kuma a wanne wuri yake?**	NAME OF FACILITY ______________________________<br>____<br>*CODE BOXES: OFFICE ONLY* [__|__|__|__|__]<br><br>STREET NAME/ADDRESS_________________________<br>____<br><br>LAND MARK DESCRIPTION______________________<br>____	

Q420	What type of health facility did you go to for maternal health services at your last visit?<br><br>***Wacce irin cibiyar kiwon lafiya kika je ta karshe, domin awon ciki ko kula kafin/bayan haihuwa?***	**PUBLIC SECTOR**<br>GOVERNMENT HOSPITAL……..…............................ 11<br>WOMEN AND CHILDREN'S HOSPITAL………………… 12<br>CHILD WELFARE CLINIC…………………................... 13<br>GOVT. HEALTH CENTER……..……………................... 14<br>GOVERNMENT POST/DISPENSARY………………….. 15<br>MATERNITY HOME……………………………………… 16<br>MOBILE CLINIC…………………………………………… 17<br>OTHER PUBLIC______________________________ 18<br>(SPECIFY)<br><br>**PRIVATE SECTOR**<br>PRIVATE HOSPITAL/CLINIC……………………………... 21<br>PRIVATE DOCTOR'S OFFICE…………………………… 22<br>NURSING/MATERNITY HOME………………………….. 23<br>PHARMACY………………………………...……………… 24<br>PMS/CHEMIST…………………..………..………………… 25<br>MOBILE CLINIC………………………….………………… 26<br>CHW/TBA………………………………….................... 27<br>TRADITIONAL HEALER………………………………….. 28<br><br>OTHER PRIVATE_____________________________ 29<br>(SPECIFY)<br><br>**FAITH-BASED SECTOR**<br>MISSION HOSPITAL……………..…............................. 31<br>FAITH-BASED, CHURCH CLINIC……………………….. 32<br><br>**OTHER SOURCE**<br>OTHER NGO HOSPITAL/CLINIC………………………… 41<br>WORKSITE CLINIC……………….……………………….. 42<br>YOUTH CENTER………………………………………….. 43<br>VCT……………………………………….................... 45<br>OTHER ___________________________________ 96<br>(SPECIFY)<br>DON'T KNOW . . . .……………………………….. 98	
Q420a	What price did you pay for maternal health services at your last visit?<br><br>**Nawa kika biya lokacin da kika je wurin awon ciki kafin/bayan haihuwa**<br><br>PLEASE INCLUDE TOTAL COST FOR ALL SERVICES RELATED TO THE MATERNAL HEALTH VISIT AS WELL AS CONSUMABLES	AMOUNT (in NAIRA) |__|__|__|__|__| <br>FREE…………………………………...……00000<br>DON'T KNOW…………………….……..…99998	
Q421	At your last visit, did you receive any information or counseling on family planning/ child birth spacing?<br><br>***Lokacin ziyarar ki ta karshe, shin kin sami bayanai ko shawara kan hanyar tsarin iyali/ tazarar haihuwa?***	YES . . . .……...……………..1<br><br>NO . . . .…….………………..2	
Q422	Did you receive a method or a referral for family planning/ contraceptive method at that time?<br><br>**Shin kin karbi wata dabara ko an tura ki wajen dabarar tsarin iyali/tazara tsakanin haihuwa/tazara tsakanin yara a wancan lokacin?**	YES, RECEIVED A METHOD. . . . . ……..……... . . 1<br>YES, RECEIVED A PRESCRIPTION……………..……... 2<br>YES, RECEIVED A REFERRAL . . . .……...…. 3<br>NO, DID NOT RECEIVE ANY OF THESE…………….. 4	

Q423	I don't want to know the results, but in the last year, have you gone to a health facility for an HIV test?<br><br>***Ba na son nasan sakamakon gwajin, a shekarar da ta wuce ko kinje cibiyar kiwon lafiya domin gwajin cutar kanjamau?***	YES . . . .1<br><br>NO . . . .2 →	Q427
Q423a	Where did you go for your last HIV test?<br><br>**A wacce cibiyar kiwon lafiyar ki ka je zuwan ki na karshe domin gwajin cutar kanjamau?**<br><br>PROBE: What is the name of this place? And where is it located?<br><br>**PROBE: Menene sunan wurin kuma a ina yake?**	NAME OF FACILITY______________________<br><br>*CODE BOXES: OFFICE ONLY* [__|__|__|__|__]<br><br>STREET NAME/ADDRESS_________________<br><br>LAND MARK DESCRIPTION _______________	
Q424	What type of facility did you go to for your last HIV test?<br><br>**Wacce irin cibiyar kiwon lafiya ki ka je zuwan kin na karshe domin gwajin cutar kanjamau?**	**PUBLIC SECTOR**<br>GOVERNMENT HOSPITAL.................................... 11<br>WOMEN AND CHILDREN'S HOSPITAL..................... 12<br>CHILD WELFARE CLINIC.................................... 13<br>GOVT. HEALTH CENTER.................................... 14<br>GOVERNMENT POST/DISPENSARY..................... 15<br>MATERNITY HOME.................................... 16<br>MOBILE CLINIC.................................... 17<br>OTHER PUBLIC_____________________ 18<br>(SPECIFY)<br><br>**PRIVATE SECTOR**<br>PRIVATE HOSPITAL/CLINIC.................................... 21<br>PRIVATE DOCTOR'S OFFICE.................................... 22<br>NURSING/MATERNITY HOME.................................... 23<br>PHARMACY.................................... 24<br>PMS/CHEMIST.................................... 25<br>MOBILE CLINIC.................................... 26<br>CHW/TBA.................................... 27<br>TRADITIONAL HEALER.................................... 28<br><br>OTHER PRIVATE__________________________ 29<br>(SPECIFY)<br><br>**FAITH-BASED SECTOR**<br>MISSION HOSPITAL.................................... 31<br>FAITH-BASED, CHURCH CLINIC.................................... 32<br><br>**OTHER SOURCE**<br>OTHER NGO<br>HOSPITAL/CLINIC.................................... 41<br>WORKSITE CLINIC.................................... 42<br>YOUTH CENTER.................................... 43<br>VCT.................................... 45<br>OTHER ____________________________________ 96<br>(SPECIFY)<br>DON'T KNOW 98	
Q424a	What price did you pay for HIV test services at your last visit?<br><br>**Nawa kika biya lokcin zuwan ki gwajin cutar kanjamau na karshe?**<br><br>PLEASE INCLUDE TOTAL COST FOR ALL SERVICES RELATED TO THE HIV TEST VISIT AS WELL AS CONSUMABLES	AMOUNT<br>(in NAIRA) [| | | |]<br><br>FREE..00000<br>DON'T KNOW....................................99998	
Q425	At the time of your last test, did you receive any information or counseling on family planning/ child birth spacing?<br><br>***Lokacin gwajin ki na karshe, shin ko kin sami bayani ko shawara kan hanyar tsarin iyali / tazarar haihuwa?***	YES . . . . 1<br><br>NO 2	

Q426	Did you receive a method or a referral for family planning/contraceptive method at that time?<br><br>**Shin kin karbi wata dabara ko an tura ki wajen dabarun tsarin iyali/tazara tsakanin haihuwa/tazara tsakanin yara a wancan lokacin?**	YES, RECEIVED A CONDOM……………………….............. 1<br>YES, RECEIVED A METHOD OTHER THAN<br>A CONDOM………………... . . . . . . ……... ……………… 2<br>YES, RECEIVED A PRESCRIPTION. . . . .…… . . …………… 3<br>YES, RECEIVED A REFERRAL . . . .……………………… 4<br>NO, DID NOT RECEIVE ANY OF THESE……..……………… 5	
Q427	In the last year, how often did you visit a pharmacy?<br><br>***A cikin shekarar da ta shige sau nawa ki ka ziyarci kantin magani?***	TIMES PER YEAR………………..1 [ ][ ]<br>TIMES PER MONTH……………..2 [ ][ ]<br>TIMES PER WEEK..………………3 [ ][ ]<br>Never visit a pharmacy…………………………….……….. 997 →	**Q430**
Q428	Have you ever received any information or counseling on family planning/birth spacing while purchasing or obtaining medicine from this pharmacy?<br><br>***Kin ta ba samun wani bayani ko shawara kan hanyar tsarin iyali/tazarar haihuwa lokacin da kike sayen maganin a wannan kantin magani?***	YES . . . .……….……….…. 1<br>NO . . . .….…………..…… 2	
Q429	In the past year, did you receive a method, information or counseling or a referral for family planning/ child birth spacing methods at the pharmacy?<br><br>***A cikin shekarar da ta shige, kin ta ba samun wata dabara, bayani, ko shawara gameda da dabarar tsarin iyali ko an tura ki wajen da ake tsarin iyali / tazara tsakanin haihuwa a kantin maganin****?*	YES, RECEIVED A METHOD…………………..…..…..A<br>YES, RECEIVED INFORMATION OR<br>COUNSELING………………………..…. . . . . .B<br>YES, RECEIVED A REFERRAL . . . …………... ... .. .C<br>NO, DID NOT RECEIVE ANY OF THESE………..….Y	
Q430	In the last year, how often did you visit a PMS/ chemist?<br><br>***A shekarar da ta shige, kamar sau nawa ki ke zuwa kantin magani/kemis akai akai,?***	TIMES PER YEAR………………...1 [ ][ ]<br>TIMES PER MONTH……………..2 [ ][ ]<br>TIMES PER WEEK..………………3…. [ ][ ]<br>Never visit a PMS/ chemist…………………….. 997 →	Q501
Q431	In the past year, did you receive a method, information or counseling or a referral for family planning/ child birth spacing methods at the PMS/chemist?<br><br>***A cikin shekarar da ta shige, kin ta ba samun wata dabara, bayani, ko shawara gameda da dabarar tsarin iyali ko an tura ki wajen da ake tsarin iyali / tazara tsakanin haihuwa a kantin magani/kemis?***	YES, RECEIVED A METHOD………………….....................…A<br>YES, RECEIVED INFORMATION OR COUNSELING………… .B<br>YES, RECEIVED A REFERRAL . . . . . . …. . ..…. …………..…..C<br>NO, DID NOT RECEIVE ANY OF THESE…..…………….........Y	

SECTION 5: SEXUAL ACTIVITY AND MARRIAGE			
**Qno**	**Questions and filters**	**Coding categories**	**Skip to**
Q501	Now I need to ask you some questions about sexual activity in order to gain a better understanding of some family life issues.<br>How old were you when you had sexual intercourse for the very first time?<br><br>***Yanzu ina son na yi maki wasu tambayoyi a kan jima'i domin samun kyaykyawar fahimta akan harkar iyali***<br><br>***Shekarun ki nawa cikakku lokacin da ki ka fara yin jima'i?***	AGE……………………..…[ ][ ]<br><br>NEVER HAD SEXUAL INTERCOURSE…………….…00 →	 <br><br>**Q509**
Q502	The first time you had sexual intercourse; did you or your partner use a family planning/birth spacing method to avoid getting pregnant?<br><br>***Lokacin da ki ka yi jima'in farko, ke ko mijin ki/abokin zamanki yayi amfani da hanyar tsarin iyali/tazarar haihuwa domin hana daukar ciki?***	YES…………………………….……………………1<br>NO……………………………………………………2 →<br>CAN'T REMEMBER…………………………………..8 →	 <br>**Q504**<br>**Q504**
Q503	Which method(s) was used?<br><br>**Wacce irin dabarar tsarin iyali ku ka yi amfani da ita?**<br><br>CIRCLE ALL MENTIONED<br><br>IF RESPONDENT SAYS "PILL", PROBE FURTHER TO ESTABLISH IF THEY MEAN THE "DAILY PILL" OR THE "EMERGENCY PILL".	FEMALE STERILIZATION…………………….A<br>MALE STERILIZATION……………….............B<br>IMPLANT……………………………….............C<br>IUD……………………………………….......D<br>INJECTABLE... . . . . ………..…………E<br>DAILY PILL……………………………………..F<br>EMERGENCY PILL (Postnor2, etc.)………....G<br>MALE CONDOM . . . .……….............H<br>FEMALE CONDOM . .. . ….. . . ……….........I<br>STANDARD DAYS METHOD/ SAFE DAYS/<br>CYCLE BEADS…..………………..................J<br>BREASTFEEDING/LAM . . . . . ….…………..K<br>OTHER MODERN METHOD<br>______________________________ L<br>(SPECIFY)<br>RHYTHM METHOD ……..………………….….M<br>WITHDRAWAL ………..…………………...........N<br>OTHER TRADITIONAL METHOD<br>______________________________X<br>(SPECIFY)	
Now I would like to ask you some questions about your recent sexual activity. Let me assure you again that your answers are completely confidential and will not be told to anyone. If we should come to a question that you don't want to answer, just let me know and we will go to the next question<br><br>***Yanzu ina son in yi mi ki tambayoyi akan jima'in da ki ka yi kwanannan. Ina son in sake tabbatar maki duk amsoshin da ki ka bada sirri ne, domin ba za'a fadawa wani ba, idan muka zo wurin tambayar da ba kya son amsawa, ki fada mini don muje tambaya ta gaba.***<br><br>**MAKE SURE YOU ARE SITTING IN A PRIVATE PLACE TO DISCUSS THE FOLLOWING QUESTIONS.**			
Q504	When was the last time you had sexual intercourse?<br><br>**Yaushe ki ka yi jima'i na karshe?**<br><br>WHEN LESS THAN A DAY, RECORD "00" DAYS.<br>IF LESS THAN ONE WEEK AGO, RECORD DAYS<br>IF LESS THAN ONE MONTH AGO, RECORD WEEKS AGO<br>IF LESS THAN 12 MONTHS AGO, RECORD MONTHS<br>IF 12 MONTHS OR MORE, RECORD IN YEARS	DAYS AGO……………1 [__|__]<br>OR<br>WEEKS AGO…………2 [__|__]<br>OR<br>MONTHS AGO……… 3 [__|__]<br>OR<br>YEARS AGO…………4 [__|__] →	 <br><br><br><br><br><br>**Q509**

Q505	How many times have you had sex in the last three (3) months?<br><br>***Sau nawa ki ka yi jima'i cikin watanni ukku (3) da suka shige?***	NUMBER OF TIMES…………… [__|__|__]<br><br>**OR**<br>NONE……………………………………….…….000<br>DAILY………………………………………..….991<br>WEEKLY ……………………………………...992<br>MONTHLY……………………………………..993<br>OTHER_____________________________ 996<br>(SPECIFY)<br>DON'T KNOW………………….…………… 998	
Q506	In total, how many men have you had sex with in the last 12 months?<br><br>***Jimillar maza nawa kika yi jima'i dasu cikin watanni goma sha biyu (12) da suka shige?***<br><br>IF MORE THAN 95, WRITE 95.	NUMBER OF PARTNERS…..[__|__]<br><br>TOO MANY TO ESTIMATE……96	
Q507	The last time you had sexual intercourse, did you or your partner use a family planning/birth spacing/child spacing method?<br>***A lokaci na karshe da kika yi jima'i, ke ko mijinki/abokin zamanki kun yi amfani da hanyar tsarin iyali/ tazarar haihuwa?***	YES……………………………………….….. 1<br>NO………………………………………….2 →<br>CAN'T REMEMBER………………………8 →	 **Q509**<br>**Q509**
Q508	Which method was used?<br><br>**Wacce irin dabara tsarin iyali ku ka yi amfani da ita?**<br><br>CIRCLE ALL MENTIONED.<br><br>IF RESPONDENT SAYS "PILL", PROBE FURTHER TO ESTABLISH IF THEY MEAN THE "DAILY PILL" OR THE "EMERGENCY PILL"	FEMALE STERILIZATION……………………A<br>MALE STERILIZATION……………….............B<br>IMPLANT…………………………….............C<br>IUD……………………………………………….D<br>INJECTABLE... . . . . ….……...….….….E<br>DAILY PILL……….………………………………F<br>EMERGENCY PILL (Postnor2, etc.)……….....G<br>MALE CONDOM . . . .………….............H<br>FEMALE CONDOM . .. . ….. . . ………….........I<br>STANDARD DAYS METHOD/ CYCLE BEADS…..……………..................................J<br>BREASTFEEDING/LAM . . . . . …..…………..K<br>OTHER MODERN METHOD<br>________________________________ L<br>(SPECIFY)<br>RHYTHM METHOD ………………………….M<br>WITHDRAWAL ………..……………….........N<br>OTHER TRADITIONAL METHOD<br>__________________________________X<br>(SPECIFY)	
**Now I would like to ask you some questions about marriage. Remember that your responses will be kept confidential.**<br><br>***Yanzu ina so nayi maki wasu tambayoyi akan auren ki.Duk amsoshin ki za su kasance a sirrance***			
Q509	Have you ever been married or lived together with a man as if married?<br><br>***Kin taba yin aure ko zama da namiji kamar zaman aure?***	YES, EVER MARRIED………………….….… 1<br>YES, LIVED WITH A MAN……………..……. …. 2<br>NO………………… ………………………….… 3	
Q510	What is your marital status now: are you currently married or living with man as if married, widowed, divorced, or separated?<br><br>***Menene matsayin auren ki yanzu: Kina da aure ko ki na zaune da namiji kamar zaman aure, ko mijin ki ya rasu, ko auren ki ya mutu ko kuma kin yi yaji ne ko kuma baki taba yin aure ba?***	CURRENTLY MARRIED………………………..1<br>LIVING WITH A MAN………………................2<br>REMARRIED……………………………….….3<br><br>WIDOWED……………….………..…………..4 }<br>DIVORCED…………………………….……....5 } →<br>SEPARATED………………………………..…6 }<br>NEVER MARRIED…………………………..…7 }	 <br><br><br><br>**Q512**
Q511	Is your husband/partner living with you now, or is he staying elsewhere?<br><br>***Shin yanzu kina tare da mijin ki/abokin zamanki, ko shi yana wani waje daban ne?***	LIVING WITH YOU……..…………………... 1<br>STAYING ELSEWHERE ………………………2	

Q512<br>**C**	CHECK Q510:<br><br>DETERMINE MONTHS MARRIED, REMARRIED OR LIVING WITH A MAN SINCE JANUARY 2012. ENTER 'X' IN COLUMN 4 OF CALENDAR FOR EACH MONTH MARRIED, REMARRIED OR LIVING WITH A MAN. ENTER 'O' IN COLUMN 4 OF CALENDAR FOR EACH MONTH SHE WAS NOT IN UNION.<br><br><u>FOR WOMEN WHO ARE WIDOWED/DIVORCED/SEPARATED/NEVER MARRIED(Q510=4 OR Q510=5 OR Q510=6 OR Q510=7):</u><br>PROBE FOR DATE WHEN LAST MARRIAGE STARTED AND TERMINATION DATE. ENTER 'X' IN COLUMN 4 OF CALENDAR FOR EACH MONTH MARRIED. ENTER 'O' IN COLUMN 4 OF CALENDAR FOR EACH MONTH WIDOWED/DIVORCED/SEPARATED/NEVER MARRIED		
Q513	CHECK MARITAL STATUS Q510<br><br>CURRENTLY MARRIED:<br>IF CIRCLED RESPONSES '1' FOR CURRENTLY MARRIED OR '2' FOR LIVING WITH A MAN OR '3' FOR REMARRIED ☐<br><br>NOT CURRENTLY MARRIED:<br>IF CIRCLED RESPONSES '4' FOR WIDOWED OR '5' FOR DIVORCED OR '6' FOR SEPERATED ☐ →<br><br>'7' FOR NEVER MARRIED ☐ →		**Q517**<br><br>**Q601**
Q514	Besides yourself, does your husband/partner have other wives?<br><br>***Ban da ke, shin mijinki/abokin zamanki yana da wasu matan ko yana zaune da wasu matan kamar zaman aure?***	YES . . . .……………….. . . …..1<br>NO . . . .………….. . . ..2 →<br>DON'T KNOW . . . .……………. ……….8 →	 **Q517**<br>**Q517**
Q515	Including yourself, in total, how many wives does your husband/partner have?<br><br>***Har da ke gabaki daya mata nawa mijinki/abokin zaman ki yake dasu?***	TOTAL NUMBER OF WIVES…………. [__|__]<br>DON'T KNOW . . . .………..… . . 98	
Q516	Are you the first, second, third, fourth…… wife?<br><br>***Shin ke ce matar fari, ta biyu, ta ukku, ta hudu…., a cikin matan mijin ki /abokan zaman ki?***	RANK…………… ☐☐	
Q517	Have you been married or lived with a man as if married only once or more than once?<br><br>***Kin ta ba yin aure, ko zama da namiji kamar kun yi aure a kalla sau daya ko fiye da haka?***	ONLY ONCE……………………….. 1<br><br>MORE THAN ONCE……………….. 2	
Q518	CHECK 517<br><br>**MARRIED/LIVED WITH MAN ONLY ONCE** ☐<br>In what month and year did you start living with your husband/ Partner?<br>***A wanne wata kuma cikin wacce shekara kika fara zama da mijinki/abokin zamanki?***<br><br>**MARRIED/LIVED WITH MORE THAN ONE MAN** ☐<br>Now I would like to ask about when you started living with your first husband/partner. In what month and year was that?<br>***Yanzu ina son in tambaya game da lokacin da ki ka fara zama da mijin ki/abokin zaman ki.A wannne wata da wacce shekara ne?***	MONTH…………………………………… ☐☐<br><br>DON'T KNOW MONTH………………………..98<br><br>YEAR……………………………………. ☐☐☐☐<br><br>DON'T KNOW YEAR………………………..9998	
Q519	How old were you when you **<u>first</u>** started living with him?<br><br>***Shekarar ki nawa lokacin farko da kika fara zama da shi?***	AGE…..…………… ☐☐<br><br>DON'T REMEMBER…….98	

SECTION 6: FERTILITY PREFERENCES

	Questions and filters		Coding categories	Skip to
Q601	CHECK Q313: METHOD CURRENTLY USING<br><br>DOES NOT USE FEMALE STERILIZATION ☐ ↓		FEMALE STERILIZATION (Q313=1) ☐ →	Q606

Now, I would like to ask you a few questions about you and your opinion about births.

Yanzu, ina son in yi maki wadansu tamboyoyi akan ki da ra'ayin ki gameda haife-haife

	Questions and filters		Coding categories	Skip to
Q602	CHECK Q233:<br>NOT PREGNANT OR UNSURE ☐ ↓<br><br>Now I have some questions about the future. Would you like to have (a/another) child, or would you prefer not to have any (more) children?<br><br>***Yanzu ina da wasu tambayoyi gameda rayuwar gaba.Kina fatan samun wani 'da/'ya ko kuma ba kya son samun karin 'ya'ya?***	PREGNANT ☐ ↓<br><br>Now I have some questions about the future. After the birth of the child you are expecting now, would you like to have another child, or would you prefer not to have any more children?<br><br>***Yanzu ina da wasu tambayoyi gameda nan gaba.Bayan wannan haihuwar da ki ke sa rai yanzu, kina bukatar sake wata haihuwa ko kuma baki bukatar kara haihuwar wasu 'ya'yan?***	HAVE (A/ANOTHER) CHILD…….1<br>NO MORE/NONE………………...2<br>SAYS SHE CAN'T GET PREGNANT…………………........3<br>UNDECIDED/DON'T KNOW…….8	 Q604<br>Q604<br>Q604
Q603	CHECK Q602:<br><br>NOT PREGNANT OR UNSURE ☐ ↓<br><br>How long would you like to wait from now before having (a/another) child?<br><br>***Har tsawon wane lokaci daga yanzu ki ke so ki dakata kafin ki sake haifar wani 'dan?***<br><br>IF RESPONSE IS IN MONTHS AND YEARS (E.G. 2 ½ YEARS), CONVERT TO MONTHS (e.g. 30 months)	PREGNANT ☐ ↓<br><br>After the birth of the child you are expecting now, how long would you like to wait before having another child?<br><br>***Bayan wannan haihuwar da ki ke sa rai yanzu,har tsawon wane lokaci ki ke so ki dakata kafin ki sake wata haihuwar?***<br><br>IF RESPONSE IS IN MONTHS AND YEARS (E.G. 2 ½ YEARS), CONVERT TO MONTHS (e.g. 30 months)	MONTHS . . . . 1 [ ][ ]<br>YEARS . . . . 2 [ ][ ]<br>SOON/NOW . . . .…… .. . .. .993<br>AFTER MARRIAGE…………………..995<br>OTHER ____________________ 996<br>(SPECIFY)<br>DON'T KNOW 998	

Q604	CHECK Q603:<br><br>NOT PREGNANT OR UNSURE ☐ ↓		PREGNANT ☐ →	Q606
Q605	In the next few weeks, if you discovered that you were pregnant, would that be a big problem, a small problem or no problem at all?<br><br>***Nan da 'yan sati kadan,In ki ka ga cewa kina da ciki,wannan zai iya zama babban matsala,karamar matsala ko ba matsala ko kadan?***		BIG PROBLEM . . . . 1<br>SMALL PROBLEM . . . . 2<br>NO PROBLEM . . . . 3<br>SAYS SHE CAN'T GET PREGNANT/NOT HAVING SEX …….4	

Q606	CHECK Q510: CURRENTLY MARRIED<br><br>CURRENTLY MARRIED OR LIVING TOGETHER OR REMARRIED (Q510=1, 2 or 3) ☐ ↓<br><br>NOT IN A UNION ☐ → Q510=(4, 5, 6 OR 7)		**Q611**
Q607	Now let's talk about your partner and his preferences for the future.<br><br>***Yanzu zamuyi magana akan mijinki /abokin zamanki da abin da ya fi so a rayuwarsa nan gaba.***<br><br>CHECK Q602:<br><br>NOT PREGNANT OR UNSURE ☐ ↓<br>Would he like to have (a/another) child, or would he prefer not to have any (more) children?<br>***Zai so ya samu wani 'da ko kuma baya son samun karin 'ya 'ya***<br><br>PREGNANT ☐ ↓<br>After the child you are expecting now, would your partner like to have another child, or would he prefer not to have any more children?<br>***Bayan wannan haihuwar da ki ke sa rai yanzu, mijin ki /abokin zamanki zai so samun wani 'da/'ya ko baya son samun karin 'ya'ya?***	HAVE (A/ANOTHER) CHILD . … . . …1<br><br>NO MORE/NONE . . . . 2<br>SAYS SHE CAN'T GET PREGNANT………….………….3<br>DON'T KNOW PARTNER'S DESIRE………………………………….8<br>(2, 3, 8 → Q609)	**Q609**
Q608	Again, this question relates to your partner's preferences for the future.<br><br>***Har yanzu, wannan tambayar ta shafi abinda mijinki/abokin zamanki zai so a rayuwarsa nan gaba***<br><br>CHECK Q607:<br><br>NOT PREGNANT OR UNSURE ☐ ↓<br>How long would your partner/husband like to wait before the birth of (a/another) child?<br>**Har tsawon wanne lokaci *mijin ki / abokin zaman ki zai so ya jira kafin ki sake haifan wani 'dan?***<br>IF RESPONSE IS IN MONTHS AND YEARS (E.G. 2 ½ YEARS), CONVERT TO MONTHS (e.g. 30 months)<br><br>PREGNANT ☐ ↓<br>After the birth of the child that you and your partner are now expecting, how long would your partner like to wait before you have another child?<br>***Bayan wannan haihuwar da ku ke jira ke da mijinki/ abokin zaman ki,har tsawon wanne lokaci ku ke son ku jira kafin haifan wani 'dan?***<br>IF RESPONSE IS IN MONTHS AND YEARS (E.G. 2 ½ YEARS), CONVERT TO MONTHS (e.g. 30 months)	MONTHS . . . . .1 ☐☐<br>YEARS . . . . 2 ☐☐<br><br>SOON/NOW . . . . 993<br>OTHER ________________ 996<br>(SPECIFY)<br>SAYS SHE CAN'T GET PREGNANT…994<br>DON'T KNOW PARTNERS DESIRE. . .998	
Q609	Does your husband/partner want the same number of children that you want, or does he want more or fewer than you want?<br><br>***Shin mijinki/abokin zamanki yana bukatar yawan 'ya 'ya kamar yadda ki ke so, ko yana son fiye da yadda ki ke so, ko kasa da yadda ki ke so?***	SAME NUMBER……………………..1<br>MORE CHILDREN…………………..2<br>FEWER CHILDREN………………...3<br>DON'T KNOW……………………..…8	

Q610	Who (will) decides how many children that you are going to have Would you say that it is mainly your decision, mainly your husband's/partner's decision, or do you decide together?<br><br>***Shin wa yake yanke shawarar yara nawa za ki samu – Za ki iya cewa ra'ayinki ne ke kadai, ko na mijinki/abokin zamanki ne, ko dukkan ku biyu ku ke yanke shawarar?***	MAINLY YOU……………………..…….1<br>MAINLY PARTNER…………………2<br>JOINTLY……………………………..3<br><br>OTHER______________________6<br>(SPECIFY)	

Q611	CHECK BIRTH HISTORY (Q216 & Q219): ANY LIVING CHILDREN?<br>Now let's talk about you again and your childbearing preferences.<br><br>PROBE FOR A NUMERIC RESPONSE.<br><br>HAS LIVING CHILDREN ☐<br>If you could go back to the time you did not have any children and could have exactly the number of children you wanted to have in your whole life, how many would that be?<br><br>***Idan zaki koma lokacin da baki fara samun yara ba kuma aka baki zabin yawan yaran da ki ke so a rayuwar ki, yara nawa za ki yi fatan samu?***<br><br>NO LIVING CHILDREN ☐<br>If you could have exactly the number of children you wanted to have in your whole life, how many would that be?<br><br>***Idan za ki iya samun yawan yaran da ki ke so a rayuwar ki,kamar nawa zaki so?***	NONE . . . . 00<br><br>NUMBER . . . . [_][_]<br><br>OTHER ______________________ 96<br>(SPECIFY)	**Q613**<br><br><br><br>**Q613**
Q612	**<u>Of this number</u>**, how many of these children would you like to be boys, how many would you like to be girls and for how many would the sex not matter?<br><br>***Cikin wannan jimlar, 'ya'ya nawa zaki fi so su kasance maza ne kuma 'ya'ya nawa zaki fi so su kasance mata ne kuma 'ya 'ya nawa su kasance ko wanne jinsi babu damuwa?***	BOYS GIRLS EITHER<br>NUMBER [_][_] [_][_] [_][_]<br><br>OTHER ______________________ 96<br>(SPECIFY)	
Now, I would like to ask you a few questions about how other women like you think about children and births.<br><br>***Yanzu, ina so nayi maki tambayoyi game da yadda wasu mata kamar ki, ke yin tunani game da yara da kuma haife haife***			
Q613	In your opinion, how long should a couple wait to have their first child?<br><br>***A ra'ayin ki har tsawon wanne lokaci ya kamata ma'aurata su jira kafin su samu haihuwar su ta farko?***	IMMEDIATELY……………………………….. 1<br>LESS THAN ONE YEAR……………………. 2<br>MORE THAN 1 YR BUT LESS THAN 2 YRS…………………….................. 3<br>2 OR MORE YEARS…………..……………. 4<br>OTHER ______________________ 6<br>(SPECIFY)<br>DON'T KNOW………………….….………... 8	
Q614	In your opinion, what should be the ideal age in years for a woman to get her first child/ pregnancy?<br><br>***A ra'ayin ki kamar shekaru nawa ne ya kamata mace ta kai kafin ta samu ciki, ko, 'danta/yarta na/ ta farko?***	AGE IN YEARS………………….[__|__]<br><br>DON'T KNOW……………………….98	
Q615	In your opinion, what should be the ideal age gap between two children?<br><br>***A ra'ayin ki kamar shekaru nawa ne ya kamata a samu tsakanin haihuwar 'ya'ya biyu?***<br><br>IF RESPONSE IS IN MONTHS AND YEARS (E.G. 2.5 YEARS), CONVERT TO MONTHS (e.g. 30 months)	MONTHS………………….1 [__|__]<br>OR<br>YEARS…………………….2 [__|__]<br><br>DON'T KNOW…………………….998	

Q616	What are the impacts on the mother if she uses a method of family planning?<br><br>***Menene sakamako (maikyau ko mara kyau) na yin amfani da dabarun tsarin iyali , ga uwa /mahaifiya idan ta yi amfani da shi?***<br><br>PROBE: ANY OTHER REASONS?<br><br>DO <u>NOT</u> READ ANSWERS<br><br>CIRCLE ALL MENTIONED.	EASY TO SPACE PREGNANCIES/CHILDREN... A<br>BETTER NUTRITIONAL STATUS……………..…… B<br>LOWER INCIDENCE OF ANEMIA…………….….C<br>LESS PREGNANCY COMPLICATIONS……….... D<br>AVOID STIS/HIV……………………………….…… E<br>REDUCE UNWANTED PREGNANCIES………..… F<br>BETTER HEALTH………………………………..…G<br>SPOUSAL HARMONY/MARITAL HAPPINESS... H<br>FEWER CHILDREN TO EDUCATE……………....I<br>SHE HAS MORE FREE TIME……………….…….J<br>FAMILY HAS MORE MONEY……………….…….K<br>PEACE OF MIND………………………………..…..L<br>PARTNER/SPOUSAL DISCORD……………….....M<br>PROBLEMS WITH MENSTRUAL CYCLE……....N<br>HEALTH PROBLEMS………………………………O<br>GAIN WEIGHT……………………………………….P<br>LOSE WEIGHT………………………………………Q<br>FATIGUE/WEAKNESS…………………………..…R<br>INFERTILITY…………………………….…………S<br>OTHER________________________________W<br>(SPECIFY)<br>OTHER________________________________X<br>(SPECIFY)<br>DON'T KNOW…………………………………….…Z<br><br>NONE…………………………………………..… Y	
Q617	What are the impacts on the child if the mother uses a method of family planning?<br><br>***Menene sakamako (maikyau ko mara kyau) na yin amfani da dabarun tsarin iyali , ga yaro idan uwar/ mahaifiyar tayi amfani da shi?***<br><br>PROBE: ANY OTHER REASONS?<br><br>DO <u>NOT</u> READ ANSWERS<br><br>CIRCLE ALL MENTIONED.	BETTER GROWTH……………………………….. A<br>BETTER NUTRITIONAL STATUS……………… B<br>BETTER HEALTH………………………………… C<br>BETTER SURVIVAL CHANCE………………….. D<br>BETTER ATTENTION BY MOTHER……………. E<br>BETTER EDUCATED…………………………….. F<br>MORE OPPORTUNITIES………………………… G<br>DEFORMATIONS………………………………… H<br>PROBLEMS WITH GROWTH…………………… I<br>NUTRITIONAL PROBLEMS……………………… J<br>FREQUENT ILLNESS…………………………….. K<br><br>OTHER_____________________________ W<br>(SPECIFY)<br><br>OTHER_____________________________ X<br>(SPECIFY)<br>DON'T KNOW………………………………………Z<br>NONE……………………………………………… Y	

SECTION 7: SPOUSAL AND INTERPERSONAL COMMUNICATION

Qno	Questions and filters	Coding categories	Skip to
Q701	Now, I would like to ask you a few questions on discussions that you might have had with other people on health issues.<br>***Yanzu zanyi miki wasu 'yan tamboyoyi akan tattaunawa da maiyiwa kika taba yi game da kiwon lafiya da wasu mutane.***<br><br>CHECK Q510:<br>CURRENTLY MARRIED OR LIVING TOGETHER (Q510=1, 2 OR 3) ☐ ↓	NOT IN A UNION (Q510= 4, 5,6, OR 7) ☐ →	**Q710**
Q702	Have you and your spouse/partner ever discussed the number of children you would like to have?<br><br>***Shin ke da mijinki/abokin zamanki kun taba maganar yara nawa ku ke son ku samu?***	YES …………………………………………… 1<br>NO…………………………………………….. 2 →	 **Q704**
Q703	How often have you talked to your spouse/partner about the number of children you would like to have in the last six (6) months?<br><br>***Sau nawa ki ka yi magana da mijinki/ abokin zamanki game da yawan yaran da zaki so ki samu a cikin watanni shidda (6) da suka shige?***	NUMBER OF TIMES TOPIC DISCUSSED ☐☐<br><br>NOT DISCUSSED AT ALL IN LAST SIX (6) MONTHS……………………………………………..97	
Q704	Have you ever discussed the use of a family planning method with your spouse/ partner?<br><br>***Shin kin taba maganan amfani da dabarar tsarin iyalii da mijinki/abokin zaman ki?***	YES ………………………………………… 1<br>NO………………………………………….. 2 →	 **Q707**
Q705	How often have you talked to your spouse/partner about the use of a family planning method in the last six (6) months?<br><br>***Sau nawa ki ka yi magana da mijin ki/ abokin zamanki gameda yin amfani da dabarar tsarin iyali a cikin watanni shidda (6) da suka shige?***	NUMBER OF TIMES TOPIC DISCUSSED ☐☐<br><br>NOT DISCUSSED AT ALL IN LAST SIX (6) MONTHS……………………………………………..97	
Q706	Who usually starts a discussion about family planning, you or your spouse/partner?<br><br>***Shin wa ya ke fara maganar tsarin iyali, ke ce ko mijinki/abokin zamanki?***	YOU...1<br>SPOUSE/PARTNER..2<br>EITHER……………………………………….……3	
Q707	How difficult is it to start a conversation about family planning with your spouse/partner – is it very difficult, somewhat difficult, or easy?<br><br>***Yaya wahalar fara maganar tsarin iyali/ tazara tsakanin haihuwa/tazara tsakanin yara da mijinki/ abokin zamanki – da wahala sosai, da 'yar wahala ko da sauki?***	VERY DIFFICULT ………………………….…..1<br>SOMEWHAT DIFFICULT ……………………..….2<br>EASY…………………………………………….…..3	
Q708	Do you intend to talk to your spouse/partner about family planning in the next three months?<br><br>***Shin kina da niyyar magana da mijinki / abokin zamanki akan tsarin iyali/tazara tsakanin haihuwa/tazara tsakanin yara a cikin watanni ukku (3) nan gaba?***	YES ……………………………………………..…1<br>NO………………………………………………….2<br>DON'T KNOW…………………………………….8	

Q709	Does your husband/partner approve or disapprove of couples using a contraceptive method to avoid pregnancy/child birth spacing?<br><br>***Shin mijinki/ abokin zamanki zai iya amincewa ko ba zai iya amincewa ba da ma'aurata suyi amfani da dabarar tsarin iyali/ /tazara tsakanin yara?***	APPROVE…. . . . . ……….….. 1<br>DISAPPROVE. . . . .…..……. 2<br>DON'T KNOW……………………………………8	
Q710	Besides yourself (or your husband/partner) who else influences the decision whether you use a method of birth spacing or not?<br><br>***Banda ke kan ki (ko mijinki/abokin zamanki), ra'ayin wanene zai shafi kudurin da zaki yi game da amfani da dabarar tsari iyali/ tazarar haihuwa ko rashin yin hakan?***<br><br>CIRCLE ALL MENTIONED.	MOTHER……………….. ....A<br>MOTHER-IN-LAW …………....…………..………B<br>SISTER(S)……………………………………….C<br>SISTER(S)-IN-LAW……………………….…….D<br>GRANDMOTHER…………………….............E<br>FRIEND(S)……………………………………....F<br>HEALTH WORKER...G<br>COMMUNITY LEADER....................................H<br>RELIGIOUS LEADER……………………….….I<br>AUNT……...…………………………………..….J<br>OTHER RELATIVES…………………………...K<br><br>OTHER ____________________________ X<br>(SPECIFY)<br>NO ONE……………………...…..………….….Y	
Q711	If you wanted to use a method of family planning, would you need anyone's permission?<br><br>***Idan kina son ki yi amfani da dabarar tsarin iyali, kina bukatar daukar izini daga wani/wata?***	YES . . . .….…………….1<br>NO . . . .……………....2 →<br>DON'T KNOW………….………………………...8 →	 **Q713**<br>**Q713**
Q712	From whom would you need permission?<br><br>***A wurin wa zaki nemi izini?***<br><br>CIRCLE ALL MENTIONED.	HUSBAND/PARTNER……………………..…A<br>FATHER.. B<br>MOTHER………….. ....C<br>FATHER-IN-LAW...D<br>MOTHER-IN-LAW …………....…………….E<br>SISTER(S)……………………………….….F<br>SISTER(S)-IN-LAW………………………...G<br>OTHER RELATIVE…………………….….H<br><br>OTHER __________________________X<br>(SPECIFY)	
Q713	Do you think that government officials should speak publicly about family planning/child birth spacing?<br><br>***A ganin ki, ya kamata ma áikatan gwamnati su yi magana a wajen taro akan dabarun tsarin iyali/ tazara tsakanin haihuwa da haihuwa?***	YES……………………………………………..…1<br>NO……………………………………….…...…2<br>DON'T KNOW …………………………..……8	
Q714	In the past year, have you heard or seen a Local Government Official speaking publicly **against** family planning/ child birth spacing?<br><br>***A cikin shekara daya da ta shige,kin ji ko kin ga wani jami'in Karamar Hukuma na magana a bainar jama'a kan rashin goyon bayan dabarun tsarin iyali/ tazara tsakanin haihuwa da haihuwa?***	YES………………………………………………1<br>NO…………………………………………….…2<br>DON'T REMEMBER …………………………..8	

Q715	In the past year, have you heard or seen a Local Government Official speaking publicly **in favour** of family planning/ child birth spacing?<br><br>***A cikin shekara daya da ta shige,Kin ji ko kin ga wani jami'in Karamar Hukuma na magana a bainar jama'a kan goyon bayan dabarun tsarin iyali/ tazara tsakanin haihuwa da haihuwa?***	YES……………………………………..……1<br>NO…………………………………………….2<br>DON'T REMEMBER …………………………8	
Q716	Do you think that religious leaders should speak publicly about family planning/child birth spacing?<br><br>***A ganin ki shuwagabannin addini na iya magana a wajen taron jama' a kan mahimmancin tsarin iyali/tazara tsakanin haihuwa?***	YES . . . . ….…….…1<br>NO . . . . ….…….…2<br>DON'T KNOW……………………………………8	
Q717	In the past year, have you heard or seen a religious leader speaking publicly **<u>against</u>** family planning/child birth spacing?<br><br>***A cikin shekarar da ta shige kin taba jin ko ganin wani shugaban addini na magana a wajen taron jama' a kan rashin mahimmancin tsarin iyali/tazara tsakanin haihuwa/tazara tsakanin yara?***	YES……………………………………….…1<br>NO…………………………………………..…2<br>DON'T REMEMBER …………………………8	
Q718	In the past year, have you heard or seen a religious leader speaking publicly **<u>in favour of</u>** family planning/child birth spacing?<br><br>***A cikin shekarar da ta shige kin taba jin ko ganin wani shugaban addini na magana a wajen taron jama' a kan goyon bayan tsarin iyali/tazara tsakanin haihuwa/tazara tsakanin yara?***	YES…………………………………………1<br>NO………………………………………..…2<br>DON'T REMEMBER ………………………...8	
Q719	How many of your close friends and relatives do you think use family planning: none, some, most, or all?<br><br>***Kawayanki da 'yanwuwanki na kusa da ke, guda nawa ne su ke amfani da dabarar tsarin iyali/tazara tsakanin haihuw/tazara tsakanin yara: ba ko daya, kadan daga cikin su, mafiya yawa daga cikin su, ko gaba ki dayansu?***	NONE……………………………………1<br>SOME…………………………….……2<br>MOST………………………………….…3<br>ALL……………………………….……4<br>DON'T KNOW………………………..….8	
Q719a	How many unmarried girls in your community do you think are sexually active: none, some, most, all?<br><br>**'Yan mata nawa ne a wannan al'umma ki ke tsammaninnsun kai misalin kwanciya da maza: Ba kowa, Wasu, Mafi yawa, ko dukan su?**	NONE………………………………………. 1<br>SOME……………………………………… 2<br>MOST……………………………………… 3<br>ALL………………………………………… 4<br>DON'T KNOW…………………………….. 8	
Q719b	Among unmarried girls who are sexually active in your community, how many do you think are using contraception: none, some, most, or all?<br>**A cikin 'Yan matan da basu yi aure ba a cikin wannan al'ummar, kamar su nawa kike tsammanin suke amfani da hanyoyin tsarin iyali. Ba kowa, Wasu, Mafi yawa, ko dukan su?**	NONE………………………………………. 1<br>SOME……………………………………… 2<br>MOST……………………………………… 3<br>ALL………………………………………… 4<br>DON'T KNOW…………………………….. 8	
Q720	Do you think there are some people within this community who will call you bad names or avoid your company if they knew that you were using a family planning/contraceptive method?<br><br>***Shin kina ganin cewa da akwai wasu mutane a cikin wannan al'uma da za su kira ki da sunaye mara sa kyau ko kuma baza su so su yi ma'amala dake ba in sun san kina amfani da dabarar tsarin iyali/tazara tsakanin haihuwa/tazara tsakanin yara?***	YES . . . . ………….…..1<br>NO . . . . …………..….2<br>DON'T KNOW……..……………..……8	

Q721	Do you think there are some people within this community who will praise, encourage, or talk favorably about you if they knew that you were using a family planning/contraceptive method?<br><br>***Shin kina ganin cewa da akwai wasu mutane a cikin wannan al'uma wanda zasu yabeki ko su baki goyon baya ko suyi magana mai kyau akan ki , in sun san kina amfani da dabarar tsarin iyali/tazara tsakanin haihuwa/tazara tsakanin yara?***	YES . . . . ……………..1<br>NO . . . . ……………..2<br>DON'T KNOW……....…………………..…8	

Please tell me how you would agree or disagree with the following statements. Do you strongly agree, agree, disagree or strongly disagree?<br><br>***Don Allah Ina son ki gaya mini ko zaki yarda ko ba zaki yarda ba da wadannan maganganun.***		**Strongly Agree**<br><br>***Kin yarda sosai***	**Agree**<br><br>**Kin yarda kawai**	**Disagree**<br><br>**Ba ki yarda ba kawai**	**Strongly Disagree**<br><br>**Ba ki yarda ba sosai**
Q722	You could start a conversation with your partner about family planning/child birth spacing.<br><br>***Za ki iya fara magana da mijinki/abokin zamanki akan tsarin iyali /tazara tsakanin haihuwa.***	4	3	2	1
Q723	You could convince your spouse/partner that you should use a method of family planning/child birth spacing.<br><br>***Za ki iya shawo kan mijinki/abokin zaman ki don ki fara amfani da dabarar tsarin iyali / tazara tsakanin haihuwa.***	4	3	2	1
Q724	You could get to a place where family planning / birth spacing / child spacing methods are offered if you decided to use one.<br><br>***Zaki iya zuwa wurin da ake bayar da dabarun tsarin iyali/tazara tsakanin haihuwa da haihuwa idan kina son ki yi amfani da shi.***	4	3	2	1
Q725	You could obtain a family planning/child birth spacing if you decided to use one.<br><br>***Za ki iya samun dabarar tsarin iyali /tazara tsakanin haihuwa/tazara tsakanin yara idan kina son ki yi amfani da shi.***	4	3	2	1
Q726	You could use a method of family planning/ child birth spacing even if your partner doesn't want you to.<br><br>***Za ki iya yin amfani da dabarar tsarin iyali /tazara tsakanin haihuwa/tazara tsakanin yara ko mijinki/abokin zamanki bai amince ba.***	4	3	2	1
Q727	You could use a method of family planning/child birth spacing/ even if none of your friends or neighbors uses one.<br><br>***Za ki iya yin amfani da dabarar tsarin iyali /tazara tsakanin haihuwa/tazara tsakanin yara ko da kawayen ki ko makwaftan ki basu amfani da shi.***	4	3	2	1
Q728	You could use a method of family planning/child birth spacing/ even if your religious leader did not think you should use one.<br><br>***Za ki iya yin amfani da dabarar tsarin iyali /tazara tsakanin haihuwa/tazara tsakanin yara ko da wani shugaban addinin yana ganin cewa bai kamata ki yi amfani dashi ba.***	4	3	2	1

Q729	You could continue to use a family planning/child birth spacing method even if you experience some side effects.<br><br>***Za ki iya cigaba da amfani da dabarar tsarin iyali/tazara tsakanin haihuwa/tazara tsakanin yara ko da kin samu wasu matsaloli lokacin da ki ke amfani da dabarun.***	4	3	2	1

INSTRUCTIONS*:* ASK QUESTION Q730, THEN QUESTION Q731, Q732 AND Q733, FOR EACH PERSON LISTED IN THE LEFT COLUMN BEFORE MOVING ON TO THE NEXT PERSON.

	Q730. In the past one year, have you talked about family planning with __________?<br><br>**A shekara daya da ta shige, kin yi maganar tsarin kayyade iyali da---------------------?**	Q731. How important is this person's opinion to you with regard to family planning/birth spacing issues?<br><br>***Kamar yaya ki ka dauki muhimmancin ra'ayin wanan mutumin kan tsarin kayyade iyali da tazarar haihuwa?***<br><br>***(Ya na da muhimmanci ne sosai, muhimmanci kawai ko babu muhimmanci gaba daya)***	Q732. Do you think that this person would support your using a method of family planning?<br><br>***Ki na tunanin wannan mutumin zai goyi bayanki kan tsarin kayyade iyali?***	Q733. Is this person's approval necessary for you to use family planning?<br><br>***Yanke hukuncin wannan mutumin yana da muhimmnci kan kiyi amfani da tsarin kayyade iyali?***
a) Your mother<br>**Mahaifiyar ki**	YES............. 1<br>NO.............. 2<br>NA………… 7→(**b**)	NOT AT ALL…….. 1<br>SOMEWHAT……… 2<br>VERY …………… 3	YES.................. 1<br>NO.................... 2<br>DK.................... 8	YES……… 1<br>NO………. 2
b) Mother-in-law<br>***Uwar mijin ki***	YES............. 1<br>NO.............. 2<br>NA………… 7→(**c**)	NOT AT ALL…….. 1<br>SOMEWHAT……… 2<br>VERY …………… 3	YES.................. 1<br>NO.................... 2<br>DK.................... 8	YES……… 1<br>NO………. 2
c) Sister-in-law<br>**Ya/kanwan miji ki**	YES............. 1<br>NO.............. 2<br>NA………… 7→(**d**)	NOT AT ALL…….. 1<br>SOMEWHAT……… 2<br>VERY …………… 3	YES.................. 1<br>NO.................... 2<br>DK.................... 8	YES……… 1<br>NO………. 2
d) Father-in-law<br>**Mahaifin mijin ki**	YES............. 1<br>NO.............. 2<br>NA………… 7→(**e**)	NOT AT ALL…….. 1<br>SOMEWHAT……… 2<br>VERY …………… 3	YES.................. 1<br>NO.................... 2<br>DK.................... 8	YES……… 1<br>NO………. 2
e) Members of your religious community?<br>**Al'ummar addinki** ?	YES............. 1<br>NO.............. 2<br>NA………… 7→**(f)**	NOT AT ALL…….. 1<br>SOMEWHAT……… 2<br>VERY …………… 3	YES.................. 1<br>NO.................... 2<br>DK.................... 8	YES……… 1<br>NO………. 2
f) Best friend?<br><br>***Babbar kawar ki*** ?	YES............. 1<br>NO.............. 2<br>NA………… 7→**Q733a**	NOT AT ALL…….. 1<br>SOMEWHAT……… 2<br>VERY …………… 3	YES.................. 1<br>NO.................... 2<br>DK.................... 8	YES……… 1<br>NO………. 2

SECTION 7a: SOCIAL NORMS			
**Qno**	**Questions and filters**	**Coding categories**	**Skip to**
Now I am going to read you scenarios about three different women and I would like to know what you think will happen in the situations.<br>***Yanzu zan karanta maki wasu misalai gameda mata kashi ukku, kuma ina son in san abinda kike tunani zai faru ga wacce.***			
The first scenario is about a young woman and her boyfriend.<br>**Misalin farko gameda. Budurwa da sauyayi**			
Q734	A 16-year old girl is dating her boyfriend who is 17-years old. They decide to have sex. What do you think most people would say about them having sex?<br><br>**Yarinya ce 'yar shekara 16 da suke nema da saurayin ta, wanda shi kuma 'dan shekara 17 ne. Sun amince da suyi jima'i.**<br>**Me kike tsammanin mutane zasu ce gameda yin jima'in su?**	DEFINITELY SHOULD NOT HAVE SEX………………….1<br>PROBABLY SHOULD NOT HAVE SEX…………………..2<br>MAYBE SHOULD NOT HAVE SEX……………………….3<br>PROBABLY SHOULD HAVE SEX ………………….4<br>DEFINITELY SHOULD HAVE SEX ………………..5	
Q735	The boy and girl have been having sex. The girl learns that her friend recently got pregnant, and now the girl is worried that she will get pregnant as well. She's heard about modern contraception and is considering using it. Do you think most people in the girl's community would say she should definitely not use contraception, probably not use contraception, maybe use contraception, probably use contraception, or definitely use contraception?<br><br>**Yarinya ce da saurayin suna yin jima'i da junan su. Yarinya ta ji cewa kawarta ta samu juna biyu, kuma ita ma tana cikin damuwa da cewa ita ma zata iya daukar ciki. Ta ji maganar hanyar tsarin iyali ta zamani kuma tana son yin amfani da ita.**<br>**Kina tunanin akasarin mutanen da ke al'ummar zasu ce:Lallai kada tayi amfani da hanyar tsarin iyali,kila kada tayi amfani da hanyar tsarin iyali,kila wa kala tayi amfani da hanyar tsarin iyali, maiyiwa tayi amfani da hanyar tsarin iyali ko lallai ya dace da tayi amfani da hanyar tsarin iyali?.**	DEFINITELY NOT USE CONTRACEPTION………………….1<br>PROBABLY NOT USE CONTRACEPTION……………………..2<br>MAYBE USE CONTRACEPTION……………………………….3<br>PROBABLY USE CONTRACEPTION………………………….4<br>DEFINITELY USE CONTRACEPTION…………………………..5	
Q736	Do you think most people in the girl's situation would definitely not use contraception, probably not use contraception, maybe use contraception, probably use contraception, or definitely use contraception?<br><br>**Shin kina jin mafi yawan mutanen da suka shiga irin halin da yarinya ta shiga lallai zasu yi amfani da hanyar tsarin iyali,Kila baza su yi amfani da hanyar tsarin iyali ba,kila wa kala zasu yi amfani da hanyar tsarin iyali, maiyiwa baza su yi amfani da hanyar tsarin iyali ba ko lallai ya dace dasuyi amfani da hanyar tsarin iyali?.**	DEFINITELY NOT USE CONTRACEPTION………………….1<br>PROBABLY NOT USE CONTRACEPTION……………………..2<br>MAYBE USE CONTRACEPTION……………………………….3<br>PROBABLY USE CONTRACEPTION………………………….4<br>DEFINITELY USE CONTRACEPTION…………………………..5	
Q737	The girl's friend is also 16 and is married and doesn't want to get pregnant with her husband just yet. Do you think most people in their community would say the friend should definitely not use contraception, probably not use contraception, maybe use contraception, probably use contraception, or definitely use contraception?<br><br>**Kawar yarinya ma shekarun ta 16 kuma tanada aure.Bata bukatar samun ciki da mijin ta yanzu. Kina tunanin cewa yawancin mutanen dake al'ummar zasu ce**<br>**lallai kada tayi yi amfani da hanyar tsarin iyali,Kila ta yi amfani da hanyar tsarin iyali ,kila wa kala zata iya yin amfani da hanyar tsarin iyali, maiyiwa ta iya yin amfani da hanyar tsarin iyali ko lallai ya dace da tayi amfani da hanyar tsarin iyali?.**	DEFINITELY NOT USE CONTRACEPTION………………….1<br>PROBABLY NOT USE CONTRACEPTION……………………..2<br>MAYBE USE CONTRACEPTION……………………………….3<br>PROBABLY USE CONTRACEPTION………………………….4<br>DEFINITELY USE CONTRACEPTION…………………………..5	

The second scenario is about a young woman and her family.: **Misali na biyu gameda Budurwa and iyayenta.**			
Q738	There is a 21 year old woman who has a daughter that is 6 months old. She wants to wait at least another year until she gets pregnant again. Do you think most people would say that it is okay for her to take steps to delay her next pregnancy?<br><br>**Akwai wata yar shekaru 21 ce kuma tana da 'ya yar wata 6. Tana bukatar ta zauna har shekara daya kamin ta sake daukar ciki.**<br>**Kina tunanin mafi yawan mutanen zasu ce tayi daidai idan ta dauki matakin domin jinkirta daukar cikin?**	DEFINITELY NOT ……………………………………………1<br>PROBABLY NOT ……………………………… ……………2<br>MAYBE ………………………………………………………..3<br>PROBABLY ……………………………………………………4<br>DEFINITELY ……………………………………………………5	
Q739	The woman would like to delay her next pregnancy. She has never used contraception before. She recently heard about contraceptives that protect against pregnancy. Do you think most people in her community would say she should definitely not use modern contraception, probably not use contraception, maybe use contraception, probably use contraception, or definitely use contraception?<br><br>**Mace tana bukatar jinkirta daukar juna biyu na gaba. Ba ta taba amfani da hanyar tsarin iyali ta zamani ba. Taji magana gameda tsarin iyali dake bada kariya ga daukar ciki.**<br>**Kina tunanin cewa yawancin mutanen dake al'ummar macen zasu ce lallai kada tayi yi amfani da hanyar tsarin iyali,Kila kada ta yi amfani da hanyar tsarin iyali ,kila wa kala zata iya yin amfani da hanyar tsarin iyali, maiyiwa ta iya yin amfani da hanyar tsarin iyali ko lallai ya dace da tayi amfani da hanyar tsarin iyali?.**	DEFINITELY NOT USE CONTRACEPTION…………………1<br>PROBABLY NOT USE CONTRACEPTION……………………..2<br>MAYBE USE CONTRACEPTION………………………………3<br>PROBABLY USE CONTRACEPTION…………………………4<br>DEFINITELY USE CONTRACEPTION…………………………..5	
Q740	The woman talks to her husband about wanting to delay their next child for at least another year. He does not want her to use contraception because he wants to have another baby soon. Do you think most people in her community would say she should definitely not use contraception, probably not use contraception, maybe use contraception, probably use contraception, or definitely use contraception without his support?<br><br>**Mace tayi magana da mijin ta gameda bukatar jinkirta haihuwa ta gaba akalla shekara daya. Maigidan baya bukatar da tayi amfani da hanyar tsarin iyali domin yana bukatar samun wata haihuwar da wuri.**<br>**Kina tunanin cewa yawancin mutanen dake al'ummar macen zasu ce lallai kada tayi yi amfani da hanyar tsarin iyali,Kila kada ta yi amfani da hanyar tsarin iyali ,kila wa kala zata iya yin amfani da hanyar tsarin iyali, maiyiwa ta iya yin amfani da hanyar tsarin iyali ko lallai ya dace da tayi amfani da hanyar tsarin iyali?.**	DEFINITELY NOT ……………………………………………1<br>PROBABLY NOT ……………………………… ……………2<br>MAYBE ………………………………………………………..3<br>PROBABLY ……………………………………………………4<br>DEFINITELY ……………………………………………………5	
Q741	Do you think most people in her situation would definitely not use contraception, probably not use contraception, maybe use contraception, probably use contraception, or definitely use contraception?<br><br>**Shin kina tunanin cewa yawacin mutanen da suka shiga halin da macen ta shiga: lallai suyi amfani da hanyar tsarin iyali,kila su yi amfani da hanyar tsarin iyali ,kila wa kala suyi amfani da hanyar tsarin iyali, maiyiwa zasu iya yin amfani da hanyar tsarin iyali ko lallai ya dace da suyi amfani da hanyar tsarin iyali?.**	DEFINITELY NOT USE CONTRACEPTION………………1<br>PROBABLY NOT USE CONTRACEPTION……………………..2<br>MAYBE USE CONTRACEPTION………………………………3<br>PROBABLY USE CONTRACEPTION………………………4<br>DEFINITELY USE CONTRACEPTION…………………………..5	

Q742	Members of the woman's religious group found out she was using contraception when her husband wanted another baby. Do you think most people in her religious community will strongly disagree, disagree, be neutral about, support, or strongly support her decision?<br><br>**Al'ummar addinin macen sun gano cewa tana amfani da hanyar tsarin iyali ta zamani lokacin da mijin ta yake bukatar haihuwa.**<br>**Kina tsammanin yawacin jama'an al'umman addinin macen zasu ki amincewa sosai, zasu kyi amincewa, ko zasu nuna halin ko in kula, zasu nuna goyon baya, ko zasu goyi bayan shawarar da ta dauka?**	STRONGLY DISAGREE WITH HER DECISION…………..1<br>DISAGREE WITH HER DECISION ……………………..…..2<br>BE NEUTRAL ABOUT HER DECISION…………..…………..3<br>SUPPORT HER DECISION………………………….…..4<br>STRONGLY SUPPORT HER DECISION …………………..5	
The third scenario is about a woman and her family.**: Misali na ukku gameda Macen da iyayenta**			
Q743	There is a 28 year old woman and she and her husband have 4 children, 2 girls and 2 boys, between the ages of 1 and 8. She doesn't want more children. Do you think most people would say that it is okay for her to take steps to prevent pregnancy?<br><br>**Akwai kuma yar shekara 28 ce kuma mijin ta na da 'ya'ya 4, 2 mata, 2 maza. 'yan shekaru tsakanin 1 da 8.**<br>**Kina tunanin cewa mafi yawan mutane zasu ce yayi daidai idan ta dau matakan hana daukar ciki?**	DEFINITELY NOT ……………………………………………1<br>PROBABLY NOT ……………….…………… ………..…2<br>MAYBE …………………………..……………………..3<br>PROBABLY ……………….……………………….……4<br>DEFINITELY ……………………………………………….5	
Q744	The woman would like to prevent another pregnancy. She has heard about modern contraception but has never used it. Do you think most people in her community would say she should definitely not use contraception, probably not use contraception, maybe use contraception, probably use contraception, or definitely use contraception<br><br>**Mace na bukatar rashin daukar wani cikin. ba ta taba jin maganar hanyar tsarin iyali ta zamani ba kuma bata taba amfani da ita ba.**<br>**Kina tunanin cewa mafi yawan mutanen dake al'ummar su macen zasu ce lallai tayi amfani da hanyar tsarin iyali,Kila ta yi amfani da hanyar tsarin iyali ,kila wa kala tayi amfani da hanyar tsarin iyali, maiyiwa zata iya yin amfani da hanyar tsarin iyali ko lallai ya dace da tayi amfani da hanyar tsarin iyali?.**	DEFINITELY NOT USE CONTRACEPTION……………….1<br>PROBABLY NOT USE CONTRACEPTION……………………..2<br>MAYBE USE CONTRACEPTION……………………………3<br>PROBABLY USE CONTRACEPTION…………………………4<br>DEFINITELY USE CONTRACEPTION…………………………..5	
Q745	Do you think that most people in your community believe she should talk to her husband about her concerns about having more children?<br><br>**Shin kina tunanin cewa mafi yawan mutane a wannan al'ummar sun yadda da macen tayi ma mijin ta magana gameda halin karin samun haihuwa?**	DEFINITELY NOT ……………………………………………1<br>PROBABLY NOT …………………………… …………….2<br>MAYBE ………………………………………………..………..3<br>PROBABLY ………………………………………….……4<br>DEFINITELY ……………………………………………….5	
Q746	The woman goes to the provider to purchase contraception. The provider learns that her husband doesn't know that she's seeking contraception. Do you think most providers in this situation would definitely not, probably not, maybe, probably, or definitely give her contraception?<br>**Mace taje wajen Jami'in tsarin iyali domin sayen maganin tsarin iyali. Jami'in tsarin iyali yaji cewa mijin ta bai san cewa tana neman maganin tsarin iyali ba.**<br>**Kina tunanin cewa mafi yawan jami'an tsarin iyali dake cikin wannan halin lallai baza su saida ba, kila baza su saida ba, maiyiwa su saida mata, kila wa kala su saida ko kuma lallai zasu bata maganin tsarin iyali?.**	DEFINITELY NOT GIVE HER CONTRACEPTION……………1<br>PROBABLY NOT GIVE HER CONTRACEPTION…………….2<br>MAYBE GIVE HER CONTRACEPTION……………………..…3<br>PROBABLY GIVE HER CONTRACEPTION…………………..4<br>DEFINITELY GIVE HER CONTRACEPTION…………………5	

SECTION 8: GENDER INEQUITY MEASURES			
**Qno**	**Questions and filters**	**Coding categories**	**Skip to**
Now I would like to ask you some questions regarding your partner and how you and your partner make household decisions. Please remember to be as honest as possible and that your responses will remain confidential; that is, no one will see your answers.<br><br>***Yanzu ina son in tambayeki gameda mijinki/abokin zamanki, kan yadda ke da mijinki/abokin zamanki kuke yanke shawara bisa ga al'amurran da suka shafi gida. Ina son ki baiyana mani gaskiya kuma duk amsar da ki ka bani zasu kasance cikin sirri, babu wanda zai ga amsar da ki ka bayar.***			
Q801	CHECK Q510: CURRENTLY MARRIED<br><br>CURRENTLY MARRIED OR LIVING TOGETHER OR REMARRIED (Q510=1 OR 2 OR 3) ☐ ↓	NOT IN A UNION (Q510=4 OR 5 OR 6 OR 7) ☐ →	**Q811**
Q802	How old was your partner on his last birthday?<br><br>***Shekarar mijinki/abokin zamanki nawa ne cikakku?***	AGE IN COMPLETED YEARS……………[__|__]	
Q803	Did your husband/partner ever attend school?<br><br>***Shin ko mijinki/abokin zamanki ya ta ba zuwa makaranta?***	YES . . . . ……………………………. 1<br>NO . . . . ……………………………. 2 →	 **Q806**
Q804	What was the highest level of school he attended: quranic, primary, secondary, or higher?<br><br>***Menene zurfin ilmin sa a karatun:***<br>***A Makarantar kur'ani, firamare, sakandare, ko gaba da sakandare?***	QURANIC ONLY……………………………………… 0 →<br>PRIMARY…………………………………………… 1<br>JUNIOR SECONDARY (JSS)……..……………… 2<br>SENIOR SECONDARY (SSS)……………………. 3<br>HIGHER……………………………………………….. 4 }<br>DON'T KNOW…………………………………………. 8 } →	**Q806**<br><br><br><br>**Q806**
Q805	What is the highest (class/year) he completed **<u>at that level?</u>**<br><br>***Menene aji /shekara mafi girma da ya kammala a wannan gurbin?***<br><br>RECORD "00" IF NO COMPLETED YEARS	CLASS/YEAR……………………….… [__|__]<br><br>DON'T KNOW………………………………98	
Q806	What is your partner's occupation, that is, what kind of work does he mainly do when he is/was working?<br><br>***Menene sana'ar mijinki/abokin zamanki, watau wanne irin aiki ya ke yi ko yayi, lokacin da yake yin aiki?***	HEALTH SERVICE PROVIDER<br>& RELATED WORKERS ………………………………….. 01<br>PROFESSIONAL, TECHNICAL, ADMINISTRATIVE,<br>MANAGERIAL & RELATED WORKERS ……………….. 02<br>SALES & RELATED WORKERS ……………………….… 03<br>SERVICE, INSTALLATIONS, MAINTENANCE<br>& REPAIR WORKERS ……………………………….… 04<br>AGRICULTURAL, ANIMAL HUSBANDRY, FORESTRY<br>WORKERS, FISHERMEN & HUNTERS …………….… 05<br>TRANSPORTATION, PRODUCTION,<br>CONSTRUCTION & EXTRACTION WORKERS ……..… 06<br>STUDENT ONLY (NOT EMPLOYED) ………………….. 07<br>NOT EMPLOYED (NOT STUDENT) ……………………. 08<br>OTHER ______________________________ 96<br>(SPECIFY)	
Q807	Is your husband/partner currently working?<br><br>***Shin mijinki ko abokin zamanki na aiki yanzu?***	YES . . . .…………..……… 1 →<br>NO . . . .…………..……… 2<br>DON'T KNOW……...………………………………….. 8	**Q809**
Q808	What has he been doing for most of the time over the last 12 months?<br><br>***Wanne irin aiki ya fi yi a koda yaushe cikin watanni sha biyu (12) da suka shige?***	NOTHING/IDLE………………………………………….. 01 }<br>LOOKING FOR WORK………………………………… 02 }<br>GOING TO SCHOOL/STUDYING…………………….. 03 }<br>UNABLE TO WORK DUE TO ILLNESS/DISABILITY.. 04 }<br>RETIRED…………………………………………………. 05 }<br><br>HOUSEHOLD/FARM WORK……………………..….. 06<br>OTHER (SPECIFY)_________________________ 96	 <br>**Q811**

Q809	Does he usually work throughout the year, seasonally, or only once in a while?<br><br>***Shin yana aiki ne kusan ko wane lokaci a cikin shekara, aikin rani ko lokaci-lokaci?***	THROUGHOUT THE YEAR . . . . ………… 1<br>SEASONALLY/PART OF THE YEAR. . …………….. 2<br>ONCE IN A WHILE . . . . ………… 3	
Q810	Does he usually earn cash for this work?<br><br>***Shin yana samun kudi idan yayi aikin?***	YES . . . . ……………………………… 1<br>NO . . . . ………………………………. 2<br>DON'T KNOW…………………………………………… 8	
Q811	What is your occupation, that is, what kind of work do you mainly do?<br><br>***Menene sana'ar ki, watau wanne irin aiki ki ka fi yi?***	HEALTH SERVICE PROVIDER<br>& RELATED WORKERS ………………………………….. 01<br>PROFESSIONAL, TECHNICAL, ADMINISTRATIVE,<br>MANAGERIAL & RELATED WORKERS ……………….. 02<br>SALES & RELATED WORKERS ………………………… 03<br>SERVICE, INSTALLATIONS, MAINTENANCE<br>& REPAIR WORKERS ……………………………………. 04<br>AGRICULTURAL, ANIMAL HUSBANDRY, FORESTRY<br>WORKERS, FISHERMEN & HUNTERS ………………… 05<br>TRANSPORTATION, PRODUCTION,<br>CONSTRUCTION & EXTRACTION WORKERS ………. 06<br>STUDENT ONLY (NOT EMPLOYED) ………………….. 07<br>NOT EMPLOYED (NOT STUDENT) ……………………. 08<br>OTHER ……………………………………………………… 96<br>(SPECIFY) ________________________________	
Q812	As you know, some women take up jobs for which they are paid in cash or kind. Others sell things, have a small business or work on the family farm or in the family business.<br>In the last seven days, have **you** done any of these things or any other work?<br><br>***Kamar yadda ki ka sani, wasu matan na yin aikin da ake biyan su kudi ko kayan kudi. Wasu kuma suna kasuwanci ko yin aikin noma ko harkar kasuwancin iyali,***<br>***A cikin satin da ya shige, kin yi irin wannan aikin ko wani aiki?***	YES . . . . ……………………………… 1 →<br>NO . . . . ………………………………. 2	**Q814**
Q813	Have you done any work in the last 12 months?<br><br>***Kin yi wani aiki a cikin watanni sha biyu (12) da suka shige?***	YES . . . . ……………………………… 1<br>NO . . . . ………………………………. 2 →	**Q817**
Q814	Do you do this work for a member of your family, for someone else, or are you self-employed?<br><br>***Ki na yin wannan aikin ne ma iyalinki ko wani mutum ko kuma kina yin aikin kanki ne?***	FOR FAMILY MEMBER . . . . ………….. 1<br>FOR SOMEONE ELSE . . . . ……………….. 2<br>SELF-EMPLOYED………………………................... 3	
Q815	Do you usually work throughout the year, or do you work seasonally, or only once in a while?<br><br>***Ki na aiki ne kowanne lokaci cikin shekara, ko kina aikin rani/damina ne ko jifa-jifa****?*	THROUGHOUT THE YEAR . . . . ………. 1<br>SEASONALLY/PART OF THE YEAR . .. . ………… 2<br>ONCE IN A WHILE . . . . ………. 3	
Q816	Are you paid in cash or kind for this work, or are you not paid at all?<br><br>***Ana biyan ki kudi ne ko kayan kudi ko kuma sam ba'a biyanki?***	IN CASH ONLY…………………………………………… 1<br>IN CASH AND IN KIND………………….................. 2<br>IN KIND ONLY…………………………………………. 3<br>NOT PAID………………………………………………. 4	
Q817	CHECK Q510: CURRENTLY MARRIED<br><br>CURRENTLY MARRIED OR LIVING TOGETHER OR REMARRIED (Q510=1 OR 2 OR 3) ☐ ↓	NOT IN A UNION ☐ →<br>(Q510=4 OR 5 OR 6 OR 7)	**Q823**

Q818	CHECK Q813 AND Q816:<br><br>IF SHE IS CURRENTLY WORKING AND EARNS CASH (Q816=1 OR 2) ☐ ↓<br><br>IF SHE DOESN'T WORK OR DOES NOT EARN CASH (Q813=2 OR Q816=3 OR 4) ☐ →	Q820

Q819	Who decides how the money that you earn will be used: mainly you, mainly your partner, or you and your partner jointly?<br><br>***Wanene yake yanke shawarar yadda za ki kashe kudin da kika samu,ke da kan ki, mijinki/abokin zamanki kadai ko ke da abokin zamanki?***	RESPONDENT . . . . 1<br>PARTNER . . . . 2<br>RESPONDENT AND PARTNER JOINTLY. ……..……. 3<br><br>OTHER ________________________________ 6<br>(SPECIFY)	

Q820	CHECK Q807 AND Q810:<br><br>IF HE IS CURRENTLY WORKING AND EARNS CASH (Q810=1) ☐ ↓<br><br>IF HE DOESN'T WORK OR DOES NOT EARN CASH (Q807=2 OR 8; OR Q810=2 OR 8) ☐ →	Q822

Q821	Who decides how the money that your partner earns will be used: Mainly you, mainly your partner, or you and your partner jointly?<br><br>***Wanene ke yanke shawarar yadda mijinki/abokin zamanki ya ke kashe kudin da ya samu?***<br>***Ke da kan ki , mijinki/abokin zamanki, , ko ke da mijinki/abokin zaman*ki ke yanke shawarar?**	RESPONDENT . . . . ……….…. 1<br>PARTNER . . . . ………... 2<br>RESPONDENT AND PARTNER JOINTLY. …………. 3<br><br>OTHER ____________________________ 6<br>(SPECIFY)	

		YES	NO
Q822	Sometimes in a marriage or a relationship, a man prohibits his wife from doing certain things. Does your husband prohibit you from:<br><br>***Wani lokaci a dangantakar zaman aure, miji yakan hana mata yin wasu abubuwa. Shin ko mijinki yana hanaki yin wadannan:***		
	a. Working outside the home?<br>**Aiki wajen gida?**	1	2
	b. Having visits from people?<br>**Ziyarar wasu mutane**	1	2
	C.Visiting your friends?<br>**Ziyarar kawaye?**	1	2
	d. Visiting your family?<br>**Ziyarar Iyalin ki?**	1	2
	e. Using contraceptives?<br>**Amfani da dabarar tsarin iyali?**	1	2
	f. Using a mobile phone?<br>**Amfani da wayar salula**	1	2
Q823	Do you have any money of your own that you alone can decide how to use?<br><br>***Kina da kudin kanki wanda ke da kanki za ki yi shawarar yadda za kiyi amfani da su?***	YES . . . . 1<br>NO 2	

		HUSBAND<br>**MIJI NE**	WIFE<br>**MATAN NE**	BOTH EQUALLY<br>**DUK KANSU NE**	DK/DEPENDS<br>**BA KI SANI BA / YADANGANTA**
Q824	In a couple, who do you think should have the greater say in each of the following decisions: the husband, the wife, or both equally:<br><br>***A ma'arauta, wanene ki ke tunanin yake da tacewa kan kowanne daga cikin wadannan shawarwarin: miji ne, mata ne, dukansu ne, baki sani ba ko ya danganta***				
	a) Making large household purchases?<br>**Babbar sayayya a gida?**	1	2	3	8
	b) Making small daily household purchases?	1	2	3	8

	**Sayayyar yau da kullum ta gida?**<br>c. Deciding when to visit family, friends, or relatives?<br>**Yanke shawarar lokacin da za'a ziyarci iyali, kawaye/abokai ko 'yanuwa?**	1	2	3	8
	d. Deciding when and where to seek medical care for your own health?<br>***Yanke shawarar lokaci da wurin da za'a nemi kulawar kiwon lafiya?***	1	2	3	8

Q825	Sometimes a man is annoyed or angered by things that his wife does. In your opinion, is a man justified in hitting or beating his wife in the following situations?<br><br>***Wani lokaci ran miji yakan baci ko yayi hushi dalilin abubuwan da matar sa ta ke yi, A ra'ayinki, ya kamata miji ya bugi matarsa a wadannan yanayin?***	YES	NO	DON'T KNOW
	a. If she goes out without telling him?<br>**Idan ta fita ba tareda ta shaida ma sa ba**?	1	2	8
	b. If she neglects the house or the children?<br>**Idan ba ta kula da gida ko yara ba?**	1	2	8
	c. If she argues with him?<br>**Idan tayi gaddama da shi?**	1	2	8
	d. If she refuses to have sex with him?<br>***Idan ta kyi yin jima'i da shi?***	1	2	8
	e. If she cooks the food improperly?<br>***Idan ba tayi girkin abinci dakyau ba?***	1	2	8
	f. If he suspects her of being unfaithful?<br>***Idan yana zargin ta da bin maza a waje?***	1	2	8
	g. If she refuses to have another child?<br>**Idan ta kyi yadda da ta kara haihuwa?**			

Now I am going to read some statements to you about relationships and children. For each statement, please tell me your opinion as to whether you strongly agree, agree, disagree, or strongly disagree with it.

Yanzu ina son in karanta maki wasu maganganu gameda yanayin dangantaka da 'ya'ya. Ko wacce magana, ki shaida mani ra'ayinki kan ko kin yadda sosai, kin yadda kawai ko baki yadda ba, ko kuma ba ki yadda da maganar ba sosai?

		Strongly Agree<br><br>Kin yarda sosai	Agree<br><br>Kin yarda kawai	Disagree<br><br>Ba ki yarda ba kawai	Strongly disagree<br><br>Baki yarda ba sosai
Q826	The husband should be the one to decide whether the couple should use a family planning/birth spacing/child spacing method<br><br>***Miji shine wanda zai yanke shawarar ko ma'arauta suyi amfani da hanyar kayyade iyali/tazarar haihuwa***	4	3	2	1
Q827	Couples who practice family planning have a better quality of life than those who do not.<br><br>***Ma'auratan da suke amfani da hanyar kayyade iyali suna da kyakkyawar rayuwa fiye da wadanda basa yi***	4	3	2	1
Q828	Husbands and wives should discuss family planning.<br><br>***Miji da mata su rika tattaunawa kan hanyar kayyade iyali tare.***	4	3	2	1
Q829	Men should not allow their wives to use family planning.<br><br>***Maza kada su bar matan su suyi amfani da hanyar kayyade tsarin iyali***	4	3	2	1

Q830	A woman who uses family planning without her husband's knowledge should be punished.<br><br>***Duk matar da tayi amfani da hanyar kayyade tsarin iyali ba tare da sanin mijinta ba, a hukunta ta***	4	3	2	1
Q831	A woman who has no children is not completed/fulfilled.<br><br>***Duk macen da ba ta da 'ya'ya ba ta cika mace ba***	4	3	2	1
Q832	A man who has no children is not completed/fulfilled.<br><br>***Duk namijin da ba ya da 'ya'ya bai cika namiji ba***	4	3	2	1
Q833	It is good to have many children because one is not sure who among them will survive to care for the parents at old age.<br><br>***Yana dakyau idan aka haihu da yawa tun da mutum baya da tabbacin wanda zai kula dashi idan ya tsufa***	4	3	2	1
Q834	The number of children a couple will have is for God only to decide.<br><br>**Adadin 'ya'ya da ma'aurata zasu haifa daga Allah yake**	4	3	2	1
Q835	A woman should continue bearing children until she has at least one son.<br><br>***Mace ta cigaba da haihuwa har sai ta samu 'da namiji***	4	3	2	1
Q836	A woman should continue bearing children until she has at least one daughter.<br><br>***Mace ta cigaba da haihuwa har sai ta samu 'ya mace***	4	3	2	1

Qno	SECTION 9: MEDIA EXPOSURE<br>Questions and filters	Coding categories	Skip to
Q901	Now I would like to talk to you about your information needs and where you get information regarding health issues.<br><br>***Yanzu ina son in yi maki tambaya game da yadda ki ke bukatar samun labarai da kuma yadda ki ke samun labaran da suka shafi harkar kiwon lafiya?***<br><br>What are your main sources for receiving health information?<br>***Wadanne hanyoyi ne ki ke samun labaran kiwon lafiya?***<br><br>PROBE SEPARATELY FOR:<br>A. Media sources: **Ta hanyar jaridun da ake bugawa ko wanda ake sauraro**<br><br>**B.** Health personnel sources: **Ta hanyar Jami'an kiwon lafiya**<br><br>C Community sources: **Ta hanyar al'umma**<br><br>D Interpersonal sources: **Ta mu'amulla da jama'a**<br><br>PROBE: Any other source? **Akwai wata hanyar kuma?**<br><br>(FOR EACH CATEGORY)<br><br>CIRCLE ALL MENTIONED	**Media Sources**<br>RADIO .... AA<br>TV .... AB<br>VIDEOS .... AC<br>NEWSPAPERS .... AD<br>MAGAZINES/BOOKS .... AE<br>FLYERS/LEAFLETS/ POSTERS/<br>STICKERS.......... AF<br>BILL BOARDS.......... AG<br>WALL PAINTING.......... AH<br>FACE BOOK.......... AI<br>INTERNET.......... AJ<br>E-MAIL.......... AK<br>SMS/CELLPHONE/TEXT MESSAGE.......... AL<br><br>**Health Personnel Sources**<br>CLINICAL OFFICER/DOCTOR .... BA<br>NURSE/MIDWIFE.......... BB<br>COMMUNITY HEALTH WORKER.......... BC<br>PHARMACY/PHARMACIST.......... BD<br>PMV/CHEMIST.......... BE<br>HOSPITAL.......... BF<br>CLINIC .... BG<br>TBA.......... BH<br>HERBALIST/TRADITIONAL HEALER.......... BI<br><br>**Community Sources**<br>CINEMA/MOBILE CINEMA.......... CA<br>VIDEO SHOPS/VIEWING CENTER.......... CB<br>SOCIAL/COMMUNITY HALLS.......... CC<br>COMMUNITY OUTREACH EVENTS<br>(THEATRE, PUPPETS,<br>ROAD SHOWS, ETC).......... CD<br>CHANGE TO LIFE EVENTS<br>(WEDDING, NAMING CEREMONY,<br>HOUSEWARMING) .... CE<br>PEER EDUCATION.......... CF<br>SCHOOL.......... CG<br>NGOS.......... CH<br>FBOS/CHURCH/MOSQUES.......... CI<br>COMMUNITY<br>MEETINGS/ASSOCIATIONS.......... CJ<br>WOMEN'S/MEN'S GROUPS.......... CK<br>SOCIAL MOBILIZER/MOBILIZERS .... CL<br><br>**Interpersonal Sources**<br>PARENTS. .... DA<br>IN-LAWS.......... DB<br>SPOUSE/PARTNER.......... DC<br>SIBLINGS.......... DD<br>SISTER/BROTHER IN LAWS.......... DE<br>FRIENDS/NEIGHBORS .... DF<br>OTHER RELATIVES.......... DG<br>CHILDREN .... DH<br><br>**OTHER SOURCES: ___________________** XX<br><br>NONE.......... YY<br>DON'T KNOW.......... ZZ	
Q902	Do you read newspaper(s)?<br>***Ki na karanta jarida/ jaridu?***	YES .... 1<br>NO 2	
Q903	Do you read magazine(s)?<br><br>***Ki na karanta mujalla/ mujallu?***	YES .... 1<br>NO 2	

Q904	CHECK: Q902 & 903: READ NEWSPAPERS AND/OR MAGAZINES<br>IF YES TO EITHER ☐ ↓	IF NO TO BOTH ☐ →	Q907
Q905	Have you read any articles on family planning/child birth spacing in newspapers/magazines in the past three months?<br><br>***Kin ta ba karanta wani sharhi kan tsarin kayyade iyali/ tazarar haihuwa a jaridu ko mujallu a watanni ukku (3) da suka shige?***	YES .... 1<br>NO .... 2 →	Q907
Q906	What information did you read in the newspapers/magazines about family planning/ birth spacing?<br><br>***Wanne labarai ki ka karanta a jaridu/mujallu kan dabarun tsarin iyali /tazara tsakanin haihuwa?***<br><br>MULTIPLE RESPONSES - CIRCLE ALL MENTIONED.<br><br>IF RESPONDENT SAYS "PILL", PROBE FURTHER TO ESTABLISH IF THEY MEAN THE "DAILY PILL" OR THE "EMERGENCY PILL"	**METHODS**<br>DAILY PILLS .... A<br>IUD .... B<br>MALE CONDOMS .... C<br>FEMALE CONDOMS ………………………………D<br>INJECTABLES…………………………………… E<br>IMPLANTS………………………………………… F<br>EC/MORNING AFTER PILL/ POSTINOR 2 ....G<br>FEMALE STERILIZATION ....H<br>MALE STERILZATION ....I<br>STANDARD DAYS METHOD (SDM)<br>/CYCLE BEADS ....J<br><br>**ISSUE**<br>AGE AT MARRIAGE……………………………… K<br>DELAYING FIRST BIRTH………………………… L<br>DELAYING AGE AT FIRST SEX/ABSTINENCE…M<br>SPACING BETWEEN BIRTHS……………………..N<br>LIMITING FAMILY SIZE…………………. ………O<br>GOVT STATEMENTS REGARDING FP………... P<br>SPOUSE/PARTNER COMMUNICATION<br>(TALK TO YOUR PARTNER) …………. ………. Q<br>GO FOR FAMILY PLANNING …………………….. R<br>PROMOTING BENEFITS OF FP………………….. S<br>PROVIDERS STATEMENTS REGARDING FP …..T<br>RELIGIOUS LEADERS STATEMENTS<br>REGARDING FP ………………………………………U<br>TALK TO A HEALTH PROVIDER<br>ABOUT FP ……………………………………………..V<br><br>OTHERS (Specify)_________________________X<br>DON'T REMEMBER …………………………………..Z	
Q907	Do you listen to the radio?<br>***Ki na sauraren rediyo?***	YES .... 1<br>NO…………………………………………………2 →	Q912
Q908	How many days in a week do you listen to radio?<br>***Kwanaki nawa a sati ki ke sauraren rediyo?***	NUMBER OF DAYS IN A WEEK ☐<br><br>LESS THAN WEEKLY/NOT REGULARLY 8	
Q909	On average how long in total do you listen to radio on a normal day?<br>***A takaice tsawon lokaci nawa a yini ki ke jin rediyo?***	HOURS PER DAY…………………1 [__|__]<br>OR<br>MINUTES PER DAY………………2 [__|__]	
Q910	Have you heard any family planning/ child birth spacing information on the radio in the past three months?<br><br>***Kin taba jin wani bayani a rediyo game da dabarun tsarin iyali /tazara tsakanin haihuwa a cikin watanni ukku (3) da suka shige?***	YES.............................……………………….1<br>NO.................................………......................2 →	Q912

Q911	What information did you hear on the radio about family planning/birth spacing?<br><br>**Wanne labarai ki ka ji a rediyo kan *dabarun tsarin iyali /tazara tsakanin haihuwa?***<br><br>MULTIPLE RESPONSES - CIRCLE ALL MENTIONED.<br><br>IF RESPONDENT SAYS "PILL", PROBE FURTHER TO ESTABLISH IF THEY MEAN THE "DAILY PILL" OR THE "EMERGENCY PILL"	**METHODS**<br>DAILY PILLS .... A<br>IUD .... B<br>MALE CONDOMS .... C<br>FEMALE CONDOMS ……………………………D<br>INJECTABLES…………………………………… E<br>IMPLANTS…………………………………………… F<br>EC/MORNING AFTER PILL/ POSTINOR 2 ....G<br>FEMALE STERILIZATION .... H<br>MALE STERILZATION ....I<br>STANDARD DAYS METHOD (SDM)<br>/CYCLE BEADS ....J<br><br>**ISSUE**<br>AGE AT MARRIAGE…………………………………K<br>DELAYING FIRST BIRTH………………………….. L<br>DELAYING AGE AT FIRST SEX/ABSTINENCE….M<br>SPACING BETWEEN BIRTHS……………………..N<br>LIMITING FAMILY SIZE…………………… ……….O<br>GOVT STATEMENTS REGARDING FP……….......P<br>SPOUSE/PARTNER COMMUNICATION<br>(TALK TO YOUR PARTNER) ……………. …………Q<br>GO FOR FAMILY PLANNING ……………………… R<br>PROMOTING BENEFITS OF FP…………………… S<br>PROVIDERS STATEMENTS REGARDING FP …..T<br>RELIGIOUS LEADERS STATEMENTS<br>REGARDING FP ………………………………………U<br>TALK TO A HEALTH PROVIDER<br>ABOUT FP ……………………………………………..V<br><br>OTHERS (Specify)__________________________X<br>DON'T REMEMBER …………………………………Z		
Q912	In your opinion, how acceptable or not acceptable are the following topics on radio?<br><br>***A ra'ayinki, menene amincewarki ko rashin amincewarki kan wadannan al'amuran a rediyo?***	ACCEPTABLE	UNACCEPTABLE	
	a. Family planning/birth spacing*:*<br>***Dabarun tsarin iyali /tazara tsakanin haihuwa***	1	2	
	b. HIV/AIDS:<br>***Ciwon sida/Kanjamau***	1	2	
	c. Maternal health (antenatal care, delivery services, postpartum care) *:*<br>**Kiwon lafiyar mata masu ciki (Kulawar daukar ciki,kulawar haihuwa da kulawar bayan haihuwa)**	1	2	
	d. Child health (immunizations, disease prevention, nutrition)<br>**Kiwon lafiyar yara (Allurar riga kafi, riga kafin cututtuka, abinci mai gina jiki)**	1	2	
	e. Reproductive health (STIs, infertility problems)<br>***Kiwon lafiya da ya shafi haihuwa(Cututtukan ciwon sanyi da matsalolin rashin haihuwa)***	1	2	

Q913	**ASK THE FOLLOWING QUESTION BASED ON CITY/STATE:**<br><br>**ILORIN:**<br>Have you ever heard about a radio program called "Ireti Eda"?<br>***Kin ta ba sauraro wani shirin rediyo da ake kira "Ireti Eda"?***<br><br>**KADUNA/JOS/PLATEAU STATE:**<br>Have you ever heard about a radio program called "Komai Nisan Jifa"?<br>***Kin ta ba sauraron wani shiri a gidan rediyo " komai nisan jifa''***<br><br>**PROBE:** "You may have heard this program played on the radio or on a CD or through a radio listener's group"<br>**PROBE: Maiyuwa kin ji wannan shirin a rediyo ko "CD" ko wurin kungiyar masu sauraron rediyo**	YES……………………………………………..…1<br><br>NO………………………………………………...2 →<br><br>DON'T KNOW .... …8 →	 <br>**Q918**<br><br>**Q918**
Q914	What was the program about?<br><br>***Menene shirin ya kumsa ?***<br><br>CIRCLE ALL MENTIONED	AGE AT MARRIAGE……………………………………A<br>DELAYING FIRST BIRTH……………………………...B<br>USE/GO FOR FAMILY PLANNING...………………..C<br>DELAYING AGE AT FIRST SEX……………………...D<br>ABSTINENCE…………………………………………...E<br>SPACING BETWEEN BIRTHS……………………….F<br>LIMITING FAMILY SIZE……………………………...G<br>TALK TO HEALTH PROVIDER ABOUT FP…………H<br>TALK TO SPOUSE/PARTNER ABOUT FP…………..I<br>PLAN YOUR FAMILY ………………………………… J<br>GET IT TOGETHER……………………………………K<br><br>OTHERS (Specify)_________________________X<br>DON'T KNOW .... …….Z	
Q915	Have you ever listened to this radio program?<br><br>***Kin ta ba sauraron wannan shirin a rediyo?***	YES……………………………………………………1<br>NO……………………………………………………….2 →<br>DON'T KNOW………………………………………....8 →<br>DON'T LISTEN TO THE RADIO……………………7 →	 **Q917**<br>**Q917**<br>**Q917**
Q916	How often did you listen to this program – every week, once or twice a month, or less than once a month?<br><br>***Kamar sau nawa kike sauraron wannan shirin- kowanne mako, a kalla sau daya ko biyu a wata, ko a kalla sau daya a wata?***	EVERY WEEK…………………………………………1<br>ALMOST EVERY WEEK……………………………..2<br>ONCE OR TWICE A MONTH………………………..3<br>LESS THAN ONCE A MONTH………………………4<br>USED TO LISTEN BUT DON'T ANYMORE……….5<br>ONLY LISTENED ONCE……………….…………..6	
Q917	Have you ever attended a meeting where this program was played or discussed?<br><br>***Kin ta ba halartar wani taron da a ka sa wannan shirin ko ake tattaunawa akai?***	YES……………………………………………………1<br>NO……………………………………………………2<br>DON'T KNOW .... …..8	
Q918	Have you heard a radio jingle or spot with people talking about family planning or child spacing during a naming ceremony?<br><br>***Kin ta ba jin wani sako a rediyo ko dandalin jama'a da ake magana akan tazara tsakanin haifuwa/tsarin Iyari a lokacin taron suna?***	YES……………………………………………………1<br>NO……………………………………………………2 →<br>DON'T KNOW .... …..8 →	 **Q920**<br>**Q920**

Q919	What were the key messages of this radio spot or jingle?<br><br>***Mennene muhimmin abinda sakon rediyon ko shirin ke dauke dashi?***	AGE AT MARRIAGE……………………………………A<br>DELAYING FIRST BIRTH……………………………..B<br>USE/GO FOR FAMILY PLANNING...…………………..C<br>DELAYING AGE AT FIRST SEX……………………..D<br>ABSTINENCE…………………………………………..E<br>SPACING BETWEEN BIRTHS………………………..F<br>LIMITING FAMILY SIZE………………………………G<br>TALK TO HEALTH PROVIDER ABOUT FP…………H<br>TALK TO SPOUSE/PARTNER ABOUT FP…………..I<br>PLAN YOUR FAMILY ………………………………… J<br><br>OTHERS (Specify)__________________________X<br>DON'T KNOW .... …….Z	
Q920	Have you heard a radio jingle or spot with people talking about family planning or child spacing in a hair dressing salon/ barbing salon?<br><br>***Kin ta ba jin wani sako a radiyo ko shirin da mutane su ke magana kan tazara tsakanin haifuwa/tsarin iyali a wurin gyaran gashi (saloon) ko kitso ko kuma gun askin maza?***	YES……………………………………………………..1<br>NO………………………………………………………2 →<br>DON'T KNOW .... …...8 →	 **Q922**<br>**Q922**
Q921	What were the key messages of this radio spot or jingle?<br><br>***Mennene muhimmin abinda sakon rediyon ko shirin ke dauke dashi?***	AGE AT MARRIAGE……………………………………A<br>DELAYING FIRST BIRTH……………………………..B<br>USE/GO FOR FAMILY PLANNING...…………………..C<br>DELAYING AGE AT FIRST SEX……………………..D<br>ABSTINENCE…………………………………………..E<br>SPACING BETWEEN BIRTHS………………………..F<br>LIMITING FAMILY SIZE………………………………G<br>TALK TO HEALTH PROVIDER ABOUT FP…………H<br>TALK TO SPOUSE/PARTNER ABOUT FP…………..I<br>PLAN YOUR FAMILY ………………………………… J<br><br>OTHERS (Specify)__________________________X<br>DON'T KNOW .... …….Z	
Q922	Have you heard a radio jingle or spot with a family planning service provider answering questions about FP or talking to a couple?<br><br>***Kin ta ba jin wani sako a rediyo ko shirin da ma'aikaciyar kiwon lafiya ke amsa tambayyoyi akan tazara tsakanin haifuwa/tsarin iyali ko kuma tsakanin ma 'aurata?***	YES……………………………………………………..1<br>NO………………………………………………………2 →<br>DON'T KNOW .... …...8 →	 **Q924**<br>**Q924**
Q923	What were the key messages of this radio spot or jingle?<br><br>***Menene muhimmin abinda sakon rediyon ko shirin ke dauke dashi?***	AGE AT MARRIAGE……………………………………A<br>DELAYING FIRST BIRTH……………………………..B<br>USE/GO FOR FAMILY PLANNING...…………………..C<br>DELAYING AGE AT FIRST SEX……………………..D<br>ABSTINENCE…………………………………………..E<br>SPACING BETWEEN BIRTHS………………………..F<br>LIMITING FAMILY SIZE………………………………G<br>TALK TO HEALTH PROVIDER ABOUT FP…………H<br>TALK TO SPOUSE/PARTNER ABOUT FP…………..I<br>PLAN YOUR FAMILY ………………………………… J<br><br>OTHERS (Specify)__________________________X<br>DON'T KNOW .... …….Z	
Q924	Have you heard a radio jingle or spot with a couple talking about FP?<br><br>***Kin ta ba jin wani sako a rediyo ko shirin da ma'aurata ke magana kan tazara tsakanin haifuwa/tsarin iyali?***	YES……………………………………………………..1<br>NO………………………………………………………2 →<br>DON'T KNOW .... …...8 →	 **Q926**<br>**Q926**

Q925	What were the key messages of this radio spot or jingle?<br><br>***Menene muhimmin abinda sakon ko shirin rediyon ke dauke dashi?***	AGE AT MARRIAGE……………………………………A<br>DELAYING FIRST BIRTH……………………………..B<br>USE/GO FOR FAMILY PLANNING...………………...C<br>DELAYING AGE AT FIRST SEX……………………...D<br>ABSTINENCE…………………………………………..E<br>SPACING BETWEEN BIRTHS………………………..F<br>LIMITING FAMILY SIZE……………………………….G<br>TALK TO HEALTH PROVIDER ABOUT FP…………H<br>TALK TO SPOUSE/PARTNER ABOUT FP…………..I<br>PLAN YOUR FAMILY ………………………………… J<br><br>OTHERS (Specify)_________________________X<br>DON'T KNOW ....…….Z	
Q926	Have you heard a woman sharing her experience supporting the use of FP (Testimonial) on radio?<br><br>***Kin taba jin magana a rediyo da wata mace ke bada shaida gameda gamsuwarta da kuma goyon bayan ta kan tazara tsakanin haihuwa/tsarin iyali?***	YES……………………………………………………..1<br>NO……………………………………………………..2 →<br>DON'T KNOW .... …...8 →	 **Q928**<br>**Q928**
Q927	What were the key messages of this radio spot or jingle?<br><br>***Menene muhimmin abinda sakon ko shirin rediyon ke dauke dashi?***	AGE AT MARRIAGE……………………………………A<br>DELAYING FIRST BIRTH……………………………..B<br>USE/GO FOR FAMILY PLANNING...………………...C<br>DELAYING AGE AT FIRST SEX……………………...D<br>ABSTINENCE…………………………………………..E<br>SPACING BETWEEN BIRTHS………………………..F<br>LIMITING FAMILY SIZE……………………………….G<br>TALK TO HEALTH PROVIDER ABOUT FP…………H<br>TALK TO SPOUSE/PARTNER ABOUT FP…………..I<br>PLAN YOUR FAMILY ………………………………… J<br><br>OTHERS (Specify)_________________________X<br>DON'T KNOW ....…….Z	
Q928	Do you watch television?<br><br>***Ki na kallon talabijin?***	YES .... 1<br><br>NO .... 2 →	**Q933**
Q929	How many days in a week do you watch television?<br><br>***Kwanaki nawa a sati ki ke kallon talabijin?***	NUMBER OF DAYS PER WEEK [ ]<br><br>NOT REGULARLY……………………….….8	
Q930	On average how long in total do you watch television on a normal day?<br><br>***A kalla kamar tsawon wanne lokaci kike kallon talabijin a rana?***	HOURS PER DAY…………………1 [__|__]<br><br>OR<br><br>MINUTES PER DAY………………2 [__|__|	
Q931	Have you seen any family planning/child birth spacing programs/ information on the TV in the past three months?<br><br>***Shin ko kin ga shirye- shirye / bayanai game da dabarar tsarin iyali /tazara tsakanin haihuwa da haihuwa a cikin watanni ukku (3) da suka shige a talabijin?***	YES.............................……….…………….1<br><br>NO.............................……………………..……..2 →	**Q933**

Q932	What information did you see on the TV about family planning/birth spacing in the last three month?<br><br>**Wanne labarai ki ka gani a talebijin gameda *dabarar tsarin iyali /tazara tsakanin haihuwa?***<br><br>MULTIPLE RESPONSES - CIRCLE ALL MENTIONED.<br><br>IF RESPONDENT SAYS "PILL", PROBE FURTHER TO ESTABLISH IF THEY MEAN THE "DAILY PILL" OR THE "EMERGENCY PILL"	**METHODS**<br>DAILY PILLS .... A<br>IUD .... B<br>MALE CONDOMS.. C<br>FEMALE CONDOMS ……………………………….D<br>INJECTABLES…………………………………… E<br>IMPLANTS………………………………………… F<br>EC/MORNING AFTER PILL/ POSTINOR 2......... G<br>FEMALE STERILIZATION .... H<br>MALE STERILZATION...I<br>STANDARD DAYS METHOD (SDM)<br>/CYCLE BEADS .... J<br><br>**ISSUE**<br>AGE AT MARRIAGE…………………………….. K<br>DELAYING FIRST BIRTH………………………. L<br>DELAYING AGE AT FIRST SEX/ABSTINENCE…M<br>SPACING BETWEEN BIRTHS……………….…….N<br>LIMITING FAMILY SIZE……………………. ………O<br>GOVT STATEMENTS REGARDING FP……….. P<br>SPOUSE/PARTNER COMMUNICATION<br>(TALK TO YOUR PARTNER) …………. ………. Q<br>GO FOR FAMILY PLANNING …………………….. R<br>PROMOTING BENEFITS OF FP………………….. S<br>PROVIDERS STATEMENTS REGARDING FP …..T<br>RELIGIOUS LEADERS STATEMENTS<br>REGARDING FP ………………………………………U<br>TALK TO A HEALTH PROVIDER<br>ABOUT FP ……………………………………………..V<br><br>OTHERS (Specify)________________________X<br><br>DON'T REMEMBER ………………………………….Z	
Q933	Have you seen a television jingle or spot that shows people talking about family planning or child spacing during a naming ceremony?<br><br>***Kin ta ba ganin wani shirin talebijin ko wani dandali da yake nuna jama'a na magana gameda dabarar tsarin iyali /tazara tsakanin haihuwa lokacin bikin zana sunan jariri?***	YES…………………………………………………….1<br>NO……………………………………………………….2 →<br>DON'T KNOW...8 →	 **Q935**<br>**Q935**
Q934	What were the key messages of this television spot or jingle?<br><br>***Menene muhimman sakonni da wannan shirin talebijin yake bayarwa?***	AGE AT MARRIAGE……………………………………A<br>DELAYING FIRST BIRTH……………………………...B<br>USE/GO FOR FAMILY PLANNING..…………………..C<br>DELAYING AGE AT FIRST SEX……………………...D<br>ABSTINENCE…………..………………………………...E<br>SPACING BETWEEN BIRTHS……………………….F<br>LIMITING FAMILY SIZE………………………………G<br>TALK TO HEALTH PROVIDER ABOUT FP…………H<br>TALK TO SPOUSE/PARTNER ABOUT FP…………..I<br>PLAN YOUR FAMILY …………………………………. J<br>GET IT TOGETHER……………………………………K<br><br>OTHERS (Specify)_________________________X<br>DON'T KNOW..Z	
Q935	Have you seen a television jingle or spot that shows people talking about family planning or child spacing in a hair dressing salon/ barbing salon?<br><br>***Kin ta ba ganin wani shirin talebijin ko wani dandali da yake nuna jama'a na magana gameda dabarar tsarin iyali /tazara tsakanin haihuwa a wajen gyaran gashi ko kitso ko kuma gun askin maza?***	YES…………………………………………………….1<br>NO……………………………………………………….2 →<br>DON'T KNOW...8 →	 **Q937**<br>**Q937**

Q936	What were the key messages of this television spot or jingle?<br><br>***Menene muhimman sakonnin da wannan shirin talebijin yake bayarwa?***	AGE AT MARRIAGE……………………………………A<br>DELAYING FIRST BIRTH……………………………..B<br>USE/GO FOR FAMILY PLANNING..…………………..C<br>DELAYING AGE AT FIRST SEX……………………...D<br>ABSTINENCE…………………………………………...E<br>SPACING BETWEEN BIRTHS………………………..F<br>LIMITING FAMILY SIZE……………………………….G<br>TALK TO HEALTH PROVIDER ABOUT FP…………H<br>TALK TO SPOUSE/PARTNER ABOUT FP…………..I<br>PLAN YOUR FAMILY ………………………………… J<br>GET IT TOGETHER…………………………………….K<br><br>OTHERS (Specify)____________________________X<br>DON'T KNOW..Z	
Q937	Have you seen a television jingle or spot that shows a family planning service provider answering questions about FP or talking to a couple?<br><br>***Kin ta ba ganin wani shirin talebijin ko wani dandali da yake nuna jami'in kayyade tsarin iyali na amsa tambayoyi gameda dabarar tsarin iyali ko ya/ta na magana da ma'aurauta?***	YES……………………………………………………1<br>NO………………………………………………………2 →<br>DON'T KNOW...8 →	 **Q939**<br>**Q939**
Q938	What were the key messages of this television spot or jingle?<br><br>***Menene muhimman sakonni da wannan shirin talebijin yake bayarwa?***	AGE AT MARRIAGE……………………………………A<br>DELAYING FIRST BIRTH……………………………..B<br>USE/GO FOR FAMILY PLANNING..…………………..C<br>DELAYING AGE AT FIRST SEX……………………...D<br>ABSTINENCE…………………………………………...E<br>SPACING BETWEEN BIRTHS………………………..F<br>LIMITING FAMILY SIZE……………………………….G<br>TALK TO HEALTH PROVIDER ABOUT FP…………H<br>TALK TO SPOUSE/PARTNER ABOUT FP…………..I<br>PLAN YOUR FAMILY ………………………………… J<br>GET IT TOGETHER…………………………………….K<br><br>OTHERS (Specify)____________________________X<br>DON'T KNOW..Z	
Q939	Have you seen a television jingle or spot that shows a couple talking about FP?<br><br>***Kin ta ba ganin wani shirin talebijin ko wani dandali da yake nuna ma'aurata na magana gameda kayyade tsarin iyali?***	YES……………………………………………………1<br>NO………………………………………………………2 →<br>DON'T KNOW...8 →	 **Q941**<br>**Q941**
Q940	What were the key messages of this television spot or jingle?<br><br>***Menene muhimman sakonnin da wannan shirin talebijin yake bayarwa?***	AGE AT MARRIAGE……………………………………A<br>DELAYING FIRST BIRTH……………………………..B<br>USE/GO FOR FAMILY PLANNING..…………………..C<br>DELAYING AGE AT FIRST SEX……………………...D<br>ABSTINENCE…………………………………………...E<br>SPACING BETWEEN BIRTHS………………………..F<br>LIMITING FAMILY SIZE……………………………….G<br>TALK TO HEALTH PROVIDER ABOUT FP…………H<br>TALK TO SPOUSE/PARTNER ABOUT FP…………..I<br>PLAN YOUR FAMILY ………………………………… J<br>GET IT TOGETHER…………………………………….K<br><br>OTHERS (Specify)____________________________X<br>DON'T KNOW..Z	
Q941	Have you seen a woman on television sharing her experience supporting the use of FP (Testimonial)?<br><br>***Kin ta ba ganin mata a talebijin ta na baiyana ma jama'a goyon bayanta kan amfanin kayyade tsarin iyali (Shaida)?***	YES……………………………………………………1<br>NO………………………………………………………2 →<br>DON'T KNOW...8 →	 **Q943**<br>**Q943**

Q942	What were the key messages of this television spot or jingle?<br><br>***Menene muhimman sakonni da wannan shirin talebijin yake bayarwa?***	AGE AT MARRIAGE……………………………………A<br>DELAYING FIRST BIRTH……………………………..B<br>USE/GO FOR FAMILY PLANNING..………………..C<br>DELAYING AGE AT FIRST SEX……………………..D<br>ABSTINENCE…………………………………………..E<br>SPACING BETWEEN BIRTHS………………………..F<br>LIMITING FAMILY SIZE………………………………G<br>TALK TO HEALTH PROVIDER ABOUT FP…………H<br>TALK TO SPOUSE/PARTNER ABOUT FP…………..I<br>PLAN YOUR FAMILY ………………………………… J<br>GET IT TOGETHER……………………………………K<br><br>OTHERS (Specify)___________________________X<br>DON'T KNOW………………………………………………Z	
Q943	In the last year, did you see a program or drama series on the television called Newman Street?<br><br>***A cikin shekarar da ta shige, ko kin ga wani shirin wasan kwaikwayo a talebijin mai suna "Newman Street"***	YES……………………………………………………1<br>NO……………………………………………………2 →<br>DON'T KNOW……………………………………………8 →	 **Q950**<br>**Q950**
Q944	In the last three months, approximately how many episodes of Newman Street did you see?<br><br>***A cikin watanni ukku(3) da suka shige, a kalla shirin wasan kwaikwayon " Newman Street" nawa kika kalla?***	NONE…………………………………………………0<br>ONE OR TWO EPISODES…………………………1<br>THREE - FIVE EPISODES…………………………2<br>SIX OR MORE EPISODES…………………………3<br>NOT SURE/CAN'T REMEMBER ……………………8	
Q945	What were the main issues or topics that Newman Street covered?<br><br>***Wadanne irin abubuwa ko darussa shirin "Newman Street" ya kumsa?***<br><br>MULTIPLE RESPONSES POSSIBLE<br><br>PROBE: ANYTHING ELSE?	CHILD HEALTH…………………………………………A<br>ECONOMIC HARDSHIPS……………………………B<br>MALARIA…………………………………………………C<br>FAMILY PLANNING……………………………………D<br>FAKE DRUGS……………………………………………E<br>OTHERS (Specify)___________________________X<br>DON'T KNOW……………………………………………Z	
Q946	What were the main messages that you got from the episodes of Newman Street that you watched? That is, what did you learn from the episodes?<br><br>***Wadanne muhimman sakonni kika samu daga shirin "Newman Street" da kika kalla? Watau me kika koya daga wannan shirin na "Newman Street"?***<br><br>MULTIPLE RESPONSES POSSIBLE<br><br>PROBE: ANYTHING ELSE?	BENEFITS OF FAMILY PLANNING…………………A<br>METHODS OF FAMILY PLANNING……………………B<br>TALK TO YOUR SPOUSE ABOUT FAMILY PLANNING ……………………………………………C<br>WHERE TO GO FOR FAMILY PLANNING …………D<br>BENEFITS OF ADEQUATELY SPACED BIRTHS..E<br>BENEFITS OF LIMITING BIRTHS…………………F<br>TALK TO YOUR HEALTH PROVIDER ABOUT FAMILY PLANNING ……………………………G<br>TALK TO YOUR FRIENDS ABOUT FAMILY PLANNING ……………………………………………H<br>FAMILY PLANNING IS SAFE ………………………I<br>THERE IS A CONTRACEPTIVE METHOD THAT IS BEST FOR YOU……………………………………J<br>TALK TO YOUR RELATIONS ABOUT FAMILY PLANNING ……………………………………………K<br>FAMILY PLANNING PROVIDERS ARE COMPETENT TO PROVIDE INFORMATION AND SERVICES ………………………………………………………L<br>FAMILY PLANNING PROVIDERS WILL ANSWER ANY QUESTIONS YOU HAVE ABOUT SIDE EFFECTS ……………………………………………M<br>OTHERS (Specify)___________________________X<br>DON'T KNOW/CAN'T REMEMBER……………………Z	
Q947	Would you say that Newman Street is entertaining, educative, both entertaining and educative, or neither entertaining nor educative?<br><br>***Shin zaki iya cewa shirin "Newman Street" yana kawatarwa, ilmantarwa,ko kuma kawatarwa da ilmantarwa ko kuma babu wata kawatarwa da ilmantarwa?***	ENTERTAINING…………………………………………1<br>EDUCATIVE………………………………………………2<br>ENTERTAINING AND EDUCATIVE…………………3<br>NEITHER ENTERTAINING NOR EDUCATIVE……….4	
Q948	In the last year, did you discuss Newman Street with anyone?<br><br>***A cikin shekarar data shige, ko kin tattauna da wani ko wata gameda da shirin talabijin na " Newman Street"?***	YES……………………………………………………1<br>NO……………………………………………………2 →<br>DON'T KNOW……………………………………………8 →	 **Q950**<br>**Q950**

Q949	If so, with whom did you discuss?<br><br>***Idan kin tattauna da wani, da wanene kuka tattauna?***	PARTNER……………………………………………..A<br>FRIEND…………………………………………………..B<br>MOTHER…………………………………………………C<br>MOTHER-IN-LAW………………………………………D<br>SISTER-IN-LAW…………………………………………E<br>FATHER-IN-LAW………………………………………..F<br>MEMBERS OF RELIGIOUS COMMUNITY…………..G<br>Other (specify)______________________________X		
Q950	In your opinion, how acceptable or not acceptable are the following topics on TV?<br>***A ra'ayinki, menene amincewarki ko rashin amincewarki kan wadannan al'amuran a Talebijin?***	**ACCEPTABLE**	**UNACCEPTABLE**	
	a. Family planning/birth spacing : ***Dabarun tsarin iyali /tazara tsakanin haihuwa***	1	2	
	b. HIV/AIDS: ***Ciwon Sida/Kanjamau***	1	2	
	c. Maternal health (antenatal care, delivery services, postpartum care) :<br>**Kiwon lafiyar mata masu ciki (Kulawar daukar ciki,kulawar haihuwa da kulawar bayan haihuwa**	1	2	
	d. Child health (immunizations, disease prevention, nutrition) :<br>**Kiwon lafiyar yara (Allurar raga kafi, riga kafin cututtuka, abinci mai gina jiki)**	1	2	
	e. Reproductive health (STIs, infertility problems) :<br>***Kiwon lafiya da shafi haihuwa (Cututtukan ciwon sanyi da matsalolin rashin haihuwa)***	1	2	
Q951	Do you go to video shows/viewing centers?<br>***Ki na zuwa wajen kallon bidiyo ko wajen da jama'a ke taruwa domin kallo?***	YES…………………………………1<br>NO……………………………………2 →		**Q953**
Q952	How **<u>often</u>** do you go to video shows?<br>***Kamar sau nawa kike zuwa wajen kallon bidiyo?***<br><br>SINGLE RESPONSE	MORE THAN ONCE PER WEEK………..1<br>EVERY WEEK ……………………………..2<br>A COUPLE OF TIMES A MONTH ………..3<br>AT LEAST ONCE A MONTH ……………4<br>AT LEAST ONCE A YEAR ………………5<br>RARELY……………………………………6		

Q953	Do you own a mobile phone that is mainly for your own use?<br>***Shin kina da wayar salular da ke kadai kike amfani da ita?***	YES……………………………………………1<br>NO………………………………………………2 →	**Q955**
Q954	In the past six months, have you received a family planning message on your mobile phone?<br><br>**Kin sami wani sakon kayyade tsarin iyali/tazarar haihuwa a wayar salular ki a watanni shidda (6) da suka wuce?**	YES……………………………………………1<br>NO………………………………………………2<br>DON'T KNOW…………………………………8	
Q955	Have you ever had a conversation about family planning over the phone?<br><br>***Kin taba tattaunawa a kan tsarin iyali a waya?***	YES…………………………………………….. 1<br>NO……………………………………………… 2 →	**Q957**
Q956	Was the person (or people) you talked to about family planning over the phone in this city, in another city, in a rural area or in another country?<br><br>***Mutumin (ko mutanen) da ki ka yi magana dasu kan tsarin iyali ta wayar a wannan birni ne, ko wani birnin, a karkara ko wata kasar suke?***<br>CIRCLE ALL MENTIONED	THIS CITY………………………………..A<br>ANOTHER CITY…………………………B<br>RURAL AREA……………………………C<br>ANOTHER COUNTRY…………………..D<br>DON'T KNOW……………………………Z	

Q957	In the past year, have you heard or seen the phrase "Get it Together"<br><br>***A cikin shekara daya da ta shige, kin ta ba ji ko ganin wannan kalmar*** "Get it Together" -***"Mu hade tare"***	YES……………………………………………..….1<br>NO…………………………………………..………..2 →<br>DON'T KNOW…..……………………….....………8 →	 **Q960a**<br>**Q960a**
Q958	What does this phrase mean to you?<br><br>***Me wannan kalmar ta ke nufi?***<br><br>CIRCLE ALL MENTIONED	AGE AT MARRIAGE……………………………………A<br>DELAYING FIRST BIRTH……………………………..B<br>USE/GO FOR FAMILY PLANNING..…………………..C<br>DELAYING AGE AT FIRST SEX……………………...D<br>ABSTINENCE…………………………………………..E<br>SPACING BETWEEN BIRTHS………………………..F<br>LIMITING FAMILY SIZE………………………………G<br>TALK TO HEALTH PROVIDER ABOUT FP…………H<br>TALK TO SPOUSE/PARTNER ABOUT FP…………..I<br>PLAN YOUR FAMILY ………………………………… J<br>GET IT TOGETHER……………………………………K<br><br>OTHERS (Specify)_________________________X<br>DON'T KNOW .... …….Z	
Q959	Where did you see or hear this phrase?<br><br><br>***A ina ki ka gani ko ki ka ji wannnan kalmar?***<br><br><br><br><br><br>CIRCLE ALL MENTIONED	RADIO DRAMA PROGRAM..…………..……….....…..A<br>RADIO JINGLE/SPOT…………………………………..B<br>TELEVISION……………………………..………………C<br>NEWSPAPER ……………………………………………..D<br>POSTER …………………………………………………E<br>UMBRELLA………………………………………..…….F<br>SHOPPING BAG…………………………………………G<br>TSHIRT………. ……………………………………….....H<br>LEAFLET/STICKERS…………………………………..I<br>BADGE/BUTTON……………………………………….J<br>CAPS……………………………………………………K<br>WRISTBAND……………………………………………L<br>ON A SIGN AT A HEALTH FACILITY……………….M<br>ON A SIGN AT A PHARMACY / CHEMIST…………N<br>AT A ROADSHOW / RALLY…………………………..O<br>AT A PARADE…………………………………………P<br>BILLBOARD……………………………………………..Q<br>PENS……………………………………………………..R<br><br>OTHER (Specify)___________________________X<br>DON'T KNOW……………………………………….…Z	

Q960	In the past year, have you attended any meetings about family planning/child birth spacing that were led by someone wearing a T-shirt with this phrase?<br><br>***A cikin shekara daya da ta wuce, kin ta ba halartar taro gameda tsarin iyali/tazarar haihuwa da aka kaddamar wadda wani yake sanye da riga mai dauke da wannan kalmar**?*	YES……………………………………………………….1<br>NO………………………………………………………..2<br>DON'T KNOW……………………………………………8	
Q960a	In the past year, have you attended any meetings about family planning/child birth spacing that were led by someone wearing a T-shirt with the phrase "Una don hear"?<br><br>***A cikin shekara daya da ta wuce, kin ta ba halartar taro gameda tsarin iyali/tazarar haihuwa a inda wanda ke kaddamar da taron yake sanye da riga wanda aka rubuta wannan taken da wannan kalmar* "Una don hear" ma'ana (Ko kun ji)*?***	YES…………………………………………1<br><br>NO…………………………………………..2 →<br><br>DON'T KNOW……………………………8 →	 <br>**Q961**<br><br>**Q961**
Q960b	What does this phrase mean to you?<br><br>CIRCLE ALL MENTIONED<br><br>***Me wannan kalmar ta ke nufi?***	AGE AT MARRIAGE……………………………………A<br>DELAYING FIRST BIRTH……………………………..B<br>USE/GO FOR FAMILY PLANNING...…………………..C<br>DELAYING AGE AT FIRST SEX……………………...D<br>ABSTINENCE…………………………………………...E<br>SPACING BETWEEN BIRTHS………………………..F<br>LIMITING FAMILY SIZE………………………………G<br>TALK TO HEALTH PROVIDER ABOUT FP…………H<br>TALK TO SPOUSE/PARTNER ABOUT FP…………..I<br>PLAN YOUR FAMILY ………………………………… J<br>GET IT TOGETHER…………………………………….K<br>OTHERS (Specify)_________________________X<br>DON'T KNOW .... …….Z	
Q961	In the past year, have you seen/heard the phrase "Know, Talk, Go."<br><br>***A cikin shekara daya da ta wuce, Kin ta ba jin wannan kalmar* "Know, Talk, Go." - *"ki sani, ki yi maganar, Je ki"***	YES……………………………………………………1<br>NO……………………………………………………...2 →<br><br>DON'T KNOW…………………………………………8 →	 **Q964**<br><br>**Q964**
Q962	What does this phrase mean to you?<br><br><br>***Me wannan kalmar ta ke nufi?***<br><br><br><br><br>CIRCLE ALL MENTIONED	AGE AT MARRIAGE……………………………………A<br>DELAYING FIRST BIRTH……………………………..B<br>USE/GO FOR FAMILY PLANNING...…………………..C<br>DELAYING AGE AT FIRST SEX……………………...D<br>ABSTINENCE…………………………………………...E<br>SPACING BETWEEN BIRTHS………………………..F<br>LIMITING FAMILY SIZE………………………………G<br>TALK TO HEALTH PROVIDER ABOUT FP…………H<br>TALK TO SPOUSE/PARTNER ABOUT FP…………..I<br>PLAN YOUR FAMILY ………………………………… J<br>GET IT TOGETHER…………………………………….K<br><br>OTHERS (Specify)_________________________X<br>DON'T KNOW...…….Z	
Q963	Where did you see or hear this phrase?<br><br><br>***A ina ki ka gani ko ki ka ji wannnan kalmar?***<br><br><br><br><br>CIRCLE ALL MENTIONED	RADIO DRAMA PROGRAM………………………….A<br>RADIO JINGLE/SPOT ………………………………..B<br>TELEVISION……………………………………………C<br>NEWSPAPER ………………………………………….D<br>POSTER ……………………………………………….E<br>UMBRELLA………………………………………………F<br>SHOPPING BAG………………………………………G<br>TSHIRT………. ………………………………………...H<br>LEAFLET/STICKERS………………………………….I<br>BADGE/BUTTON……………………………………….J<br>CAPS…………………………………………………….K<br>WRISTBAND……………………………………………L<br>ON A SIGN AT A HEALTH FACILITY…………………M<br>ON A SIGN AT A PHARMACY / CHEMIST…………N<br>AT A ROADSHOW / RALLY…………………………O<br>AT A PARADE…………………………………………P<br>BILLBOARD…………………………………………….Q<br>PENS……………………………………………………..R<br><br>OTHER (Specify)___________________________X<br>DON'T KNOW…………………………………………….Z	

Q964	In the past year, have you seen/heard the phrase ""No dulling"?<br><br>***A cikin shekara daya da ta wuce, kin ta ba jin wannan kalmar ""No dulling"? ma'ana "Rashin walwala"***	YES……………………………………………………..1<br>NO……………………………………………………….2 →<br>DON'T KNOW………………………………………8 →	 **Q967**<br>**Q967**
Q965	What does this phrase mean to you?<br><br><br>***Me wannan kalmar ta ke nufi?***<br><br><br>CIRCLE ALL MENTIONED	AGE AT MARRIAGE……………………………………A<br>DELAYING FIRST BIRTH……………………………..B<br>USE/GO FOR FAMILY PLANNING...………………...C<br>DELAYING AGE AT FIRST SEX……………………...D<br>ABSTINENCE…………………………………………...E<br>SPACING BETWEEN BIRTHS……………………….F<br>LIMITING FAMILY SIZE……………………………….G<br>TALK TO HEALTH PROVIDER ABOUT FP…………H<br>TALK TO SPOUSE/PARTNER ABOUT FP…………..I<br>PLAN YOUR FAMILY ………………………………… J<br>GET IT TOGETHER……………………………………K<br><br>OTHERS (Specify)_________________________X<br>DON'T KNOW..Z	
Q966	Where did you see or hear this phrase?<br><br>***A ina ki ka gani ko ki ka ji wannnan kalmar?***<br><br><br><br><br><br><br><br><br><br><br>CIRCLE ALL MENTIONED	RADIO DRAMA PROGRAM..…………..………..…….A<br>RADIO JINGLE/SPOT …..………………….…..………..B<br>TELEVISION……………………………….……………C<br>NEWSPAPER ………………………………………..…..D<br>POSTER ……………………………….………….……E<br>UMBRELLA……………………………………………….F<br>SHOPPING BAG…………………………………………G<br>TSHIRT………. ………………………………………....H<br>LEAFLET/STICKERS…………………………………..I<br>BADGE/BUTTON………………………………………J<br>CAPS…………………………………………………….K<br>WRISTBAND……………………………………………L<br>ON A SIGN AT A HEALTH FACILITY………………….M<br>ON A SIGN AT A PHARMACY / CHEMIST…………N<br>AT A ROADSHOW / RALLY…………………………..O<br>AT A PARADE…………………………………………P<br>BILLBOARD……………………………………………..Q<br>PENS……………………………………………………..R<br><br>OTHER (Specify)___________________________X<br>DON'T KNOW………………………………………..…Z	
Q967	Do you understand Yoruba language?<br><br>***Kina jin harshen Yarbanci?***	 YES……………………………………………………1<br>NO……………………………………………………...2 →	 <br>**Q977**
Q968	In the past year, have you heard the phrase "Se o jasi" – (meaning "Are you into it?" or Are you part of it")?<br><br>***A shekara daya da ta shige, kin ji wannan kalmar "Se o jasi" –(ma'ana " Kina cikinta"? Ko kina da alaka da ita"***	YES…………………………………………………1<br>NO……………………………………………………..2 →<br><br>DON'T KNOW………………………………………8 →	 **Q971**<br><br>**Q971**
Q969	What does this phrase mean to you?<br><br>***Menene wannan kalmar ta ke nufi?***<br><br><br>[IF RESPONDENT ONLY GIVES THE COMMON DEFINITION OF THE PHRASE, PROBE TO SEE IF THERE IS ANY OTHER MEANING.<br><br>CIRCLE ALL MENTIONED	AGE AT MARRIAGE……………………………………A<br>DELAYING FIRST BIRTH……………………………..B<br>USE/GO FOR FAMILY PLANNING...………………...C<br>DELAYING AGE AT FIRST SEX……………………...D<br>ABSTINENCE…………………………………………...E<br>SPACING BETWEEN BIRTHS……………………….F<br>LIMITING FAMILY SIZE……………………………….G<br>TALK TO HEALTH PROVIDER ABOUT FP…………H<br>TALK TO SPOUSE/PARTNER ABOUT FP…………..I<br>PLAN YOUR FAMILY ………………………………… J<br>GET IT TOGETHER……………………………………K<br><br>OTHERS (Specify)_________________________X<br>DON'T KNOW..Z	
